# Human Embryonic Stem Cell Lines and Their Use in International Research

**DOI:** 10.1002/stem.286

**Published:** 2010-02

**Authors:** Peter Löser, Jacqueline Schirm, Anke Guhr, Anna M Wobus, Andreas Kurtz

**Affiliations:** a Robert Koch InstituteBerlin, Germany; b Leibniz Institute of Plant Genetics and Crop Plant ResearchGatersleben, Germany; c Berlin Brandenburg Center for Regenerative Therapies and European Human Embryonic Stem Cell Registry (hESCreg)Charité, Berlin, Germany

**Keywords:** Embryonic stem cells, Pluripotent stem cells, hESC Lines, hESC Research, hESC Usage, Human induced pluripotent stem cells, hiPSCs

## Abstract

Research in human pluripotent stem cells, including human embryonic stem cells (hESC) and human induced pluripotent stem cells (hiPSC), is one of the most dynamic research fields. Despite the high public attention, especially for hESC research, there is only scattered information on the number of hESC lines and the degree, dynamics, and diversification of their use on a global level. In this study we present data on the current number of publicly disclosed hESC lines, on the extent and impact of experimental work involving hESCs, and on the use of specific hESC lines in international research. The results are based on the evaluation of nearly 1,000 research papers published by the end of 2008, which describe experimental work on hESCs, and of a comprehensive database of published hESC lines. The average impact of hESC research papers is high at 7.422, with a predominance of research output by the United States. Of at least 1,071 original hESC lines derived up to November 2009 at 87 institutions in 24 countries, only a fraction is thoroughly characterized. Our data show the global predominance of a few hESC lines in research, but also reveal remarkable country-specific differences. Comparison of hESC and hiPSC application did not show a diminished role for hESC research, but rather revealed that, up to this time, both fields continue to expand, exist independently, and partially overlap. Stem Cells 2010;28:240–246

## INTRODUCTION

Research with human embryonic stem cell (hESC) lines has attracted increasing attention over the last decade because these cells have the capability to proliferate indefinitely and to differentiate into any cell type of the body. On the other hand, there has been much controversy about using hESCs due to their origin from early human embryos, which resulted in a wide panel of different national legislations on human ESC research [1]. In a preceding report, we have determined the number of hESC cell lines and quantified the extent of experimental research involving hESCs [2], but since then hESC research has further intensified and spread into many more countries. The release of new National Institutes of Health (NIH) guidelines on research involving hESCs in the United States (http://stemcells.nih.gov/policy/2009guidelines.htm) has evoked much interest in the question of how many cell lines are available worldwide. However, to date there is no current information on how many hESC lines exist worldwide, and literature data on this question are based on estimates and are somewhat divergent [3,4]. Although there are now several registries that contain partially overlapping data sets on a multitude of hESC lines [5–7], the level of characterization of the cell lines listed in these registries is highly variable and, most importantly, the extent of their use in international research can not be assessed. Moreover, there are no data that elucidate whether there is a preferential use of certain cell lines in different countries. We have determined the number of hESC lines and analyzed the scale of hESC research on a global level, including the use of specific hESC lines. We found remarkable differences between countries. Our studies are based on the analysis of a databank of hESC lines that was previously described [2,8] and has been updated regularly up to November 2009, as well as on the evaluation of nearly 1,000 research papers describing experimental work on hESCs up to the end of 2008.

## Materials and Methods

The methods for extracting papers on experimental work on hESCs and human induced pluripotent stem cells (hiPSCs) from the PubMed database are given in the supporting information. Methods used to determine impact factors, citation frequencies, international collaboration, and NIH funding of hESC work as well as the sources from which information on hESC lines was received are provided as supporting information.

## RESULTS

### Number of hESC Lines

We first determined the number of hESC lines reported up to November 24, 2009, in the scientific literature as well as in stem cell registries, on web pages or in press releases. According to our data, publicly available information exists on at least 1,071 original hESC lines that were derived at 87 institutions in 24 countries (Table 1). For 694 of these cell lines (64.8%), either derivation has been described in English-language peer-reviewed journals or these cell lines have been used in experimental work published in such journals. For 590 cell lines, at least some characterization data were reported in published articles. Thus, the number of hESC lines reported in the scientific literature has markedly increased since 2005, when only 171 of 414 known hESC lines were reported in the scientific literature (2). hESC lines not published in scientific journals to date comprise, for example, cell lines derived recently (e.g., 20 hESC lines from France and 17 lines from Denmark listed in hESCreg, the hESC registry of the European Union, as well as 68 lines in the United States, announced recently by the NIH) and many cell lines derived at the Reproductive Genetics Institute in Chicago and distributed by Stemride International Limited. While characterization data for the former will hopefully be published in the near term, only little information is publicly available for many of the hESC lines from the Reproductive Genetics Institute. Moreover, a large fraction of the hESC lines derived before August 2001 and originally registered at the NIH has not been described in the literature and is not publicly available for research so far.

**Table 1:** Overview of hESC lines publicly reported up to November 2009

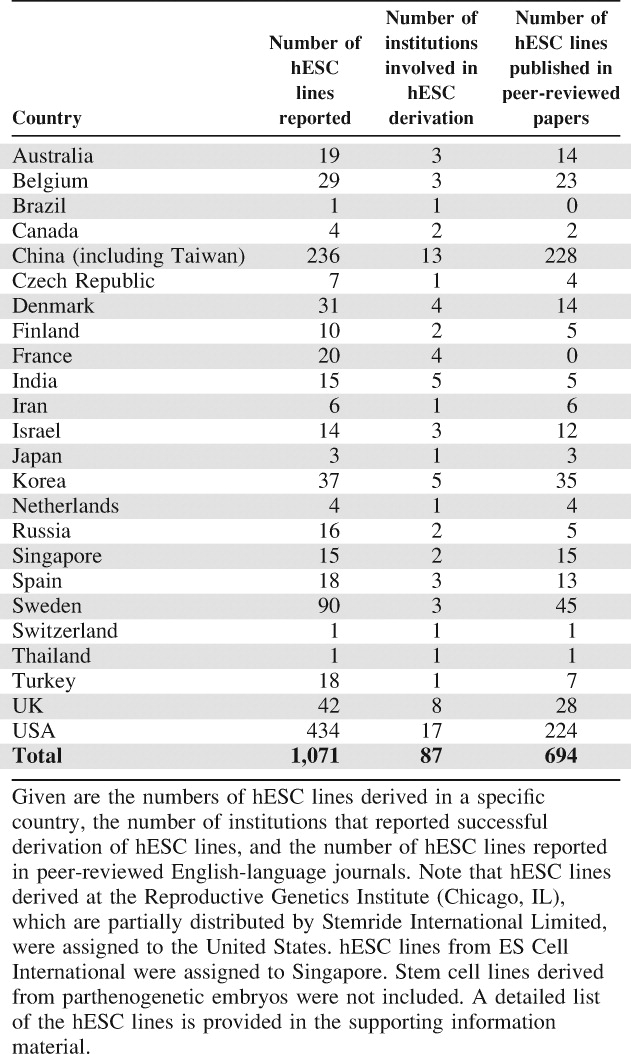

When analyzing characteristics and specificities in derivation modalities of hESC lines, two aspects should be noted. First, among hESC lines publicly reported, at least 116 (10.8%) harbor genetic modifications linked to 33 inheritable human diseases (Table 2). These cell lines were usually derived from embryos examined by preimplantation genetic diagnosis. By the end of 2005, only 27 of the 414 publicly known hESC lines (6.5%) harbored genetic alterations linked to an inheritable human disease [2]. Thus, there has been a considerable increase in this fraction of hESC lines over the past 4 years. When compared to recently published figures [9], the number of reported “diseased” hESC lines even doubled. This highlights the outstanding interest in this type of hESC lines, because they might be a valuable tool for studying the pathomechanisms of genetically inheritable diseases [10,11]. Second, although xeno-free derivation of hESC lines was considered to be a crucial prerequisite for later clinical use [12], only a few hESC lines were produced without any exposure to animal-derived compounds or in accordance with standards of good manufacturing practice. However, many cell lines were derived on human feeder cells, without performing immune surgery or under conditions free of animal-derived serum. A detailed list of the hESC lines reported here is provided as supporting information. In addition, there is a multitude of hESC sublines, some of which have been applied frequently, such as the sublines H9.2 of H9 (WiCell Research Institute, Inc., Madison, WI, http://www.wicell.org), ENVY of HES-3 (ESI, ES Cell International Pte Ltd, Singapore, http://www.escellinternational.com), or SA002.5 of Sahlgrenska 002 (Cellartis AB, Göteburg, Sweden, http://www.cellartis.com), which were not considered separately in the present analysis.

**Table 2:** Overview of hESC lines with genetic disorders linked to inheritable human diseases as determined by November 2009

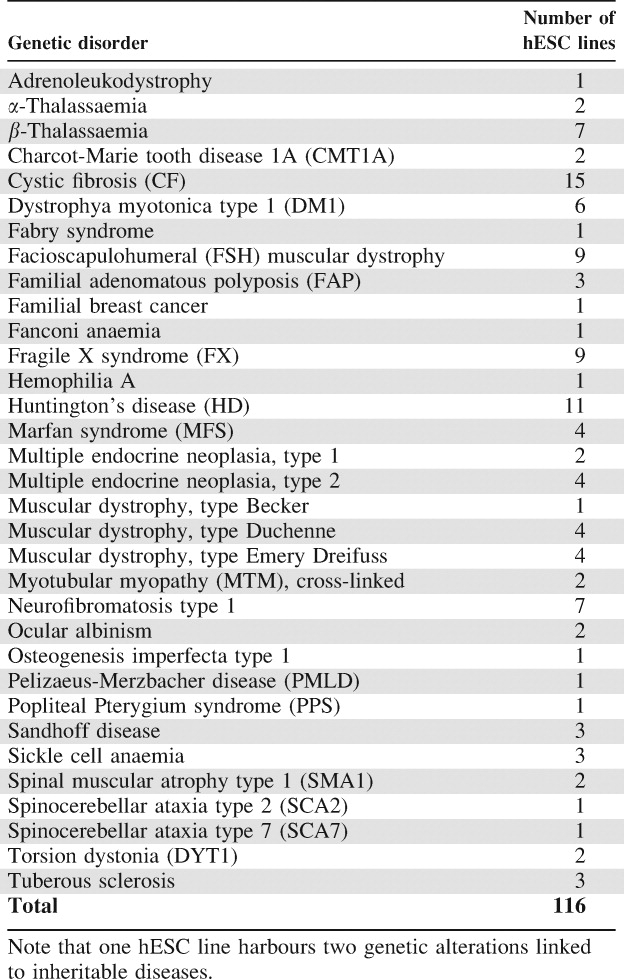

### Number and Impact of Publications Regarding Experimental Work Involving hESC

**Figure 1:**
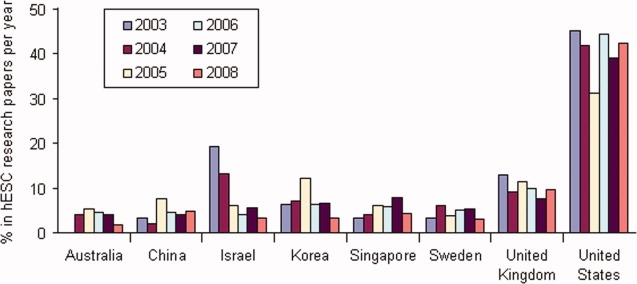
Share of published hESC research from selected countries in the total number of research papers published 2003–2008 worldwide in English language journals.

Next, we analyzed the number and impact of scientific papers published by the end of 2008 and describing results of hESC derivation and experimental work involving hESCs. We performed a broad search of the PubMed database for papers describing hESC work according to the method described earlier (for details, see also supporting information methods), which resulted in nearly 6,000 initial hits. These results were manually evaluated to exclude those papers in which hESCs were not experimentally used (e.g., comments, reviews, news, editorials, work on mouse ES cells, papers on ethical or political aspects of hESC research, etc.). We further excluded articles that summarized previously described methods and protocols, as well as papers in which only hESC-derived materials (e.g., DNA, RNA, or protein extracts) were used. Work involving stem cell lines derived from parthenogenetic embryos was also excluded from the analysis. Finally, we identified a total of 989 hESC research papers from 27 countries published between 1998 and 2008 in English-language peer-reviewed journals (a complete list of papers is provided in the supporting information). As shown in Table 3, most of these 989 research papers originated from groups in the United States (41.1%), followed by the United Kingdom (9.5%), Israel (7.2%), Korea (6.2%), and Singapore (5.6%). The leading position of US research in the hESC field has remained nearly unchanged as approximately 40% of publications in this field were from groups based in the United States over the last 6 years, although the total extent of this research increased dramatically worldwide (Fig. 1).

**Table 3:** Number of hESC research papers published 1998–2008

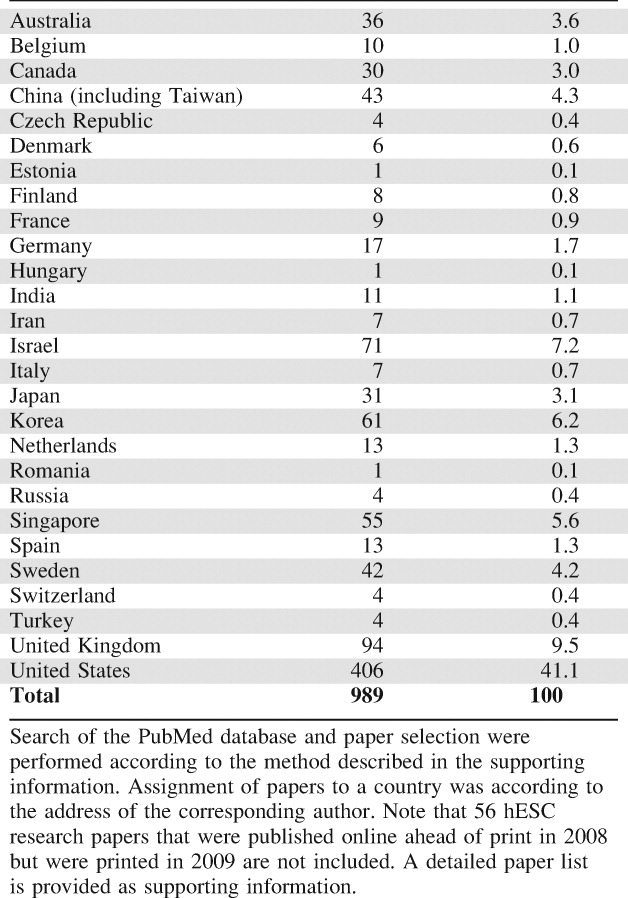

Because the number of papers does not necessarily reflect the impact of research, we also determined the Institute for Science Information citation impact factor for journals that published those 989 papers. The weighted overall average 5-year impact factor (2004–2008) for journals that had published experimental hESC work was 7.422, indicating the outstanding interest in hESC research. However, hESC work from US-based groups was published in journals with a higher average impact factor (9.123). The same is true for work published by Canadian groups (10.769), while work from Chinese, Korean, and Swedish groups were published in less influential journals (average 5-year impact factors of 4.041, 4.425, and 4.768, respectively). Because the impact factor of a journal does not necessarily reflect the impact or quality of individual papers published in this journal [13], we also determined citation frequencies for said 989 papers (supporting information). According to these data, hESC papers published between 2000 and 2007 were cited on average 14.9 times by studies published in 2008. However, studies from US and Canadian groups were cited more often (averages of 19.5 and 17.4 citations in 2008, respectively), while work from Chinese, Korean, and Swedish groups were cited less frequently (6.7, 7.1, and 8.1 times in 2008, respectively). Moreover, among the 20 hESC research papers cited most frequently in the scientific literature in 2008, fifteen (75%) were from groups in the United States, and US groups were involved in two others (supporting information). These findings extend and confirm our own earlier results on the leadership of US research in the hESC field [2,8].

hiPSCs show many, if not all, characteristics of hESCs, but production of hiPSC lines does not require the destruction of human embryos. Additionally, potential clinical use of autologous hiPSCs might not be hindered by immunological problems inherent to allogeneic hESC-derived cells. Since the first description of hiPSCs in 2007, it was speculated that research using hiPSCs may replace hESC research in the near term, and that hESCs would only be needed for comparison in the future. Some researchers involved in cloning and hESC research even claimed to concentrate solely on hiPSC research in the future [14]. Therefore, we considered to what extent research involving hiPSCs and hESCs would overlap and whether there were indications that hESC research would decline. In a first step toward investigating this question, we analyzed the number of papers describing experimental work involving hiPSCs by applying the same criteria as for experimental hESC studies and using the search string described in the supporting information. Of 16 hiPSC research papers extracted by our search of 2008 publications, 14 (87.5%) also reported application of hESCs or hESC-derived materials. In 2009, the number of experimental hiPSC studies published in English-language peer-reviewed journals dramatically increased to 66 (preliminary data for January 1 to September 30, 2009). Of these studies, 47 (71.2%) also involved application of hESCs or hESC-derived materials. The number of experimental hESC studies for the same period was 342 (preliminary data for January 1 to September 30, 2009) showing a further expansion of international hESC research in 2009. These findings allow for the conclusion that, at least in 2008 and 2009, both research fields existed independently, were further extended, and partially overlap. Moreover, the vast majority of studies on hESCs published in 2009 did not involve hiPSCs but, conversely, research on hiPSCs partially involved the application of hESCs or hESC-derived materials.

### Use of hESC Lines in International Research

**Figure 2:**
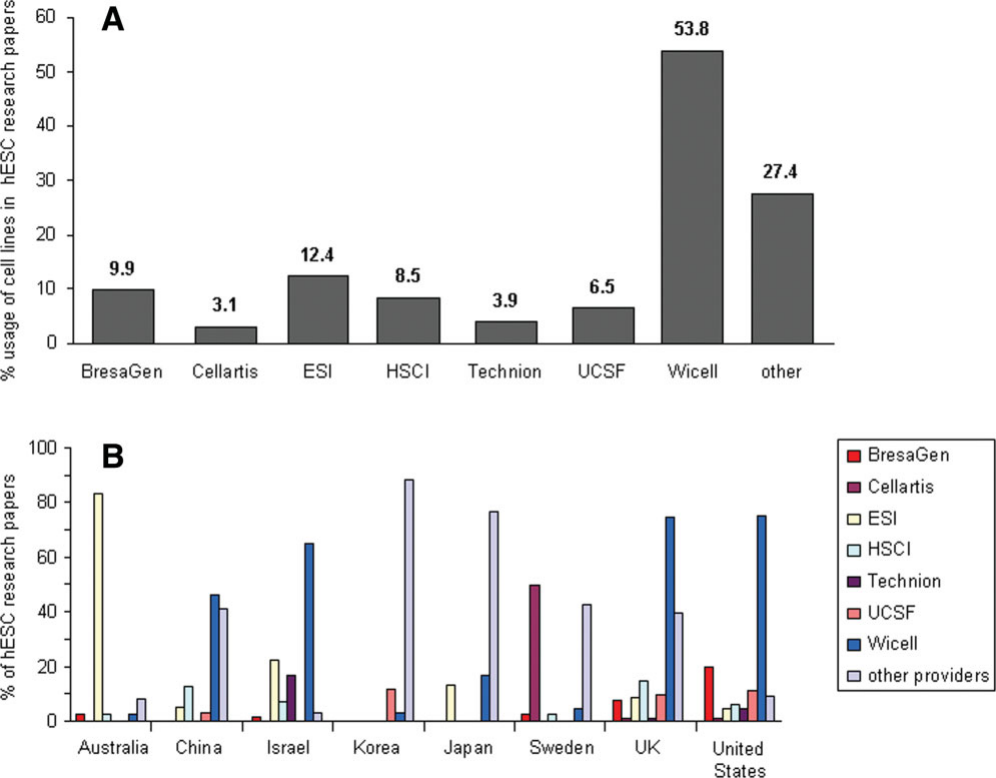
Use of hESC lines of selected providers in international research and in published research from selected countries. **A:** The share of cell lines from a specific provider in the cell lines used overall in hESC research papers published 1998–2008. BresaGen, BresaGen, Inc., Athens, Georgia; Cellartis, Cellartis AB, Göteburg, Sweden; ESI, ES Cell International Pte Ltd, Singapore; HSCI, Harvard Stem Cell Institute, Massachusetts; Technion, Israel Institute of Technology, Haifa, Israel; UCSF, University of California San Francisco, California; WiCell, WiCell Research Institute, Madison, Wisconsin. Cell lines from other providers contain hESC lines from 48 providers such as Karolinska Institute, Stockholm, Sweden (3.4%); Seoul National University, Seoul, Korea (3.0%); Kyoto University, Japan (2.4%); University of Newcastle, United Kingdom (1.8%); and University of Sheffield, United Kingdom (1.3%). Note that in many studies more than one hESC line was used. **B:** The percentage of hESC papers from a specific country produced with cell lines of the given providers. Cell lines from providers other than those specifically named were grouped in “other providers.”

Finally, we were interested to determine the use of hESC lines in international research. A complete and detailed list on the hESC lines used in scientific studies investigated here is provided as supporting information. Of 989 papers, 964 contained information on the specific hESC lines used. As shown in Figure 2A, most international hESC research published between 1998 and 2008 was performed with hESC lines provided by WiCell, which were derived in 1998 by Thomson and colleagues [15], or derivatives thereof. In 53.8% of all hESC studies published by end of 2008, at least one WiCell line or a derivative of such a line was used either without (36.3%) or with (17.5%) cell lines from other producers. Cell lines from other producers were less frequently used. However, there are significant country-specific differences in the use of hESC cell lines from certain providers (Fig. 2B). Whereas 75% of US-based research was performed with at least one WiCell line, only a small percentage of papers originating from Australia, Korea, or Sweden (which together account for more than 14% of hESC research papers) was based on these hESC lines (1.8%, 3.3%, and 4.8%, respectively). More than 80% of hESC research published by Australian groups was performed with hESC lines of ESI. Similarly, work reported by Swedish groups was mainly performed with hESC lines from Cellartis AB and from another Swedish provider, Karolinska Institute. Korean groups nearly exclusively used hESC lines provided by Korean producers. In Japan, Singapore, and China, 16.7%, 40%, and 46.7% of reported research was exclusively or partially performed with WiCell lines, respectively (Fig. 2B), whereas a large portion of hESC work was performed with hESC lines derived in the respective country. For example, 23 of 31 hESC research papers from Japanese groups reported work performed with hESC lines provided by Kyoto University. These lines have not been used outside Japan for legal reasons.

In contrast, all hESC research reported from Germany by the end of 2008 was performed involving hESC lines from WiCell (data not shown). Although hESC research is strictly regulated in Germany by the Stem Cell Act, it was permitted to use any of the hESC lines originally registered at the NIH. Therefore, it seems unlikely that the preferential use of just the WiCell lines is only due to a restrictive stem cell research policy. This view is supported by the fact that a high percentage of hESC work was also based on WiCell lines in countries with a highly permissive hESC research policy, such as Great Britain and Canada (74.5% and 90%, respectively).

Moreover, we wished to determine whether the wide use of WiCell lines could be a direct consequence of a possible interdependence of international hESC research, which could lead to an indirect transmission of US stem cell research policies to collaborative research in other countries. We therefore performed an analysis of authors' affiliations in the 989 research papers investigated here. This analysis revealed that, even in those countries with predominant use of WiCell lines, only a relatively low percentage of hESC papers evolved from direct collaboration with US groups (Canada, 20%; Israel, 13.1%; United Kingdom, 10.6%). Similarly, only 22% of hESC work published by US groups was the result of a direct collaboration with groups outside the United States.

Of all hESC lines reported in the literature by the end of 2008, H9 and H1 (including their derivatives) were the most frequently used (36.7% and 30.8% of published studies, respectively) (Table 4). In published research from US groups, these two cell lines were used even more often (52.6% and 47.4% of hESC publications, respectively). We therefore wondered whether the preferential use of these two cell lines over other NIH-approved cell lines might be a consequence of preferential NIH funding. This seems not to be the case. According to information from the 989 papers investigated here, 69.1% and 70.9% of studies performed with hESC lines H1 and H9 in the US had NIH or other governmental funding. Similarly, publications based on work with two other NIH approved cell lines frequently used in the United States (BG01 and UC06) were funded by the NIH to a comparable degree (72.8% and 81.5%, respectively).

**Table 4:** Most frequently used hESC lines

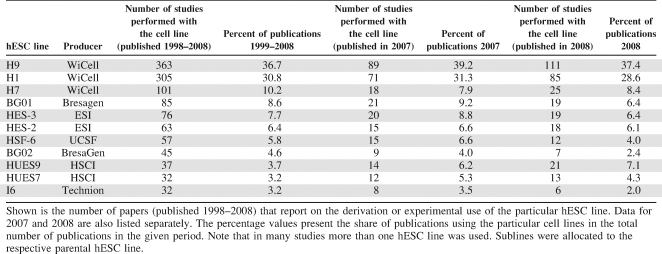

On the other hand, our data show that non-WiCell lines have also been widely used. For example, the Harvard Stem Cell Institute (HSCI) line HUES9, which has been available for only 5 years, was used in more than 7% of experimental hESC work published in 2008. Overall, cell lines from HSCI have been used in 12.3% and 12.9% of published work from 15 countries in 2007 and 2008, respectively, indicating diversification of cell lines used in international hESC research.

## DISCUSSION

In this study, we have analyzed the scale and impact of hESC research on a global level, including the use of hESC lines. We found evidence for at least 1,071 original hESC lines, a large portion of which has been reported in the scientific literature so far. However, despite this multitude of hESC lines, only cell lines from a few providers have been extensively used in published international research, with remarkable differences between countries.

Although we found evidence for more than 1,000 supposed hESC lines, the number of available and well-characterized lines is probably considerably lower. It even remains an open question whether the nearly 700 cell lines reported in the scientific literature have all the characteristics of human pluripotent cell lines and are available for research. For example, characterization data in scientific articles are often only presented exemplarily for individual cell lines, whereas derivation of multiple cell lines was reported. Furthermore, more than 400 hESC lines were described or used in only a single study with no follow-up studies so far. On the other hand, our list contains several as yet unpublished hESC lines with characteristics of true human pluripotent stem cells. For example, there are 14 hESC lines that were not published in the scientific literature until the end of 2008, but were accepted by the United Kingdom stem cell bank, which requires a specific level of characterization. Similarly, 68 hESC lines derived at institutions in the United States and not reported in the literature so far have been announced for NIH registration between September and November 2009. It is most likely that these cell lines will also have characteristics of truly pluripotent cells. Moreover, several companies, such as Cellartis, Novocell, or Advanced Cell Technology, Inc. with extensive experience in hESC research have reported the existence of hESC lines for which no characterization data have yet been published in the scientific literature. Some cell lines from Russia and China have obviously been well-characterized but only published in Russian or Chinese language journals, respectively. Therefore, it remains difficult to determine the number of well-characterized and available hESC lines. While the International Stem Cell Initiative characterized 59 hESC lines from 17 countries and was able to derive common and robust molecular patterns as a step toward the standardization of these cells [3], the actual level of characterization for most hESC lines is highly variable, often incomplete in accessible registries, and thus of limited value for comparison.

We also analyzed the extent and impact of international research involving hESCs. By 2008, the number of hESC research papers had more than tripled compared to the period from 1998 to 2005 [2]. In the same period, the average impact factor of journals in which original hESC work was published increased from 6.03 to 7.422. Thus, the high average impact factor of hESC research is probably not a mere consequence of the novelty of the hESC field but rather reflects a continuous and spreading interest in this type of research. Our data also underscore the leading position of the United States in hESC research with respect to quantity and quality of this research, which remained unchanged over the last decade. Other studies found an increasing productivity gap in US hESC research and an underperformance of US research in the field, respectively [16,17]. The reasons for the differing assessments may be partially due to a different data pool used. For example, Levine [17] included in his analysis all journal articles citing the first hESC paper by Thomson et al. [15] and therefore might have included articles that do not report original hESC work but rather summaries of or reflections on work with hESC cells. On the other hand, many more recent hESC research papers do not cite Thomson et al. [8]. In contrast, we have extensively analyzed all articles extracted by our analysis and even excluded papers describing previously reported work in articles published in rather methodical journals such as *Nature Protocols*, *Stem Cell Protocols*, or *Methods in Molecular Biology*.

Our data further reveal the preferential use of only a few cell lines in international hESC research until the end of 2008. We found a pronounced predominance of cell lines provided by the WiCell Research Institute, which was even stronger in US-based studies. However, our data also uncover regional differences in the use of hESC lines. The reasons for this phenomenon are not clear, and explanations remain speculative. For example, the low usage of WiCell lines in Australia, Korea, or Sweden might be due to the fact that these countries had their own hESC lines available at the start of international hESC research in the early years of this decade. On the other hand, the high percentage in use of WiCell lines in work from Great Britain and Canada may be explained by the early need of cells for hESC work in these countries before the later availability of domestic stem cell lines. The preferential use of WiCell lines in hESC studies from Israel (64.8% of research papers despite the early availability of their own hESC lines) might be due to the involvement of Israeli scientists in the derivation of the first WiCell lines and the establishment of well-suited sublines of H9 in Israel. However, a considerable portion of studies from Israel did not contain information on the specific cell lines used.

Importantly, we did not find proof for a direct correlation between the nature of a country's stem cell research policy (restrictive versus permissive) and the use of certain hESC lines nor for an indirect transmission of US stem cell policies to other regions due to highly collaborative research. Thus, other factors, such as early publicity by the provider or by early users, distribution policy, or the grade of characterization and documentation of a cell line (which increases with the frequency of its use), might contribute to the decision for using a specific cell line.

While this manuscript was in preparation, Scott et al. published a study on the use of human embryonic stem cell lines [18]. The authors found a strong predominance of only two hESC lines, WiCell H1 and H9, in experimental hESC research. This finding was based on the number of requests for certain hESC lines to the NIH Stem Cell Bank and to the HSCI, and on the evaluation of 534 publications for which hESC lines were used. Unfortunately, it is not obvious from this study whether it was focused on the US situation only or also included hESC work from other countries. Moreover, it is not clear what literature data pool was used for this analysis. Although our data, in principle, confirm the finding on the predominance of WiCell lines, there are also major differences to those reported by Scott et al. For example, these authors found that 83.3% and 60.9% of all hESC studies were based on the H9 and H1 cell lines, respectively, whereas we found evidence for application of these cell lines and their derivatives in 36.7% and 30.8% of hESC studies worldwide, respectively. This discrepancy is most likely due to a more comprehensive data set used in our study. Whether the US stem cell policies and resulting NIH funding restrictions are responsible for the preferred use of just two WiCell lines still remains an open question.

In summary, our data provide a detailed picture on the status of international hESC research based on a systematic analysis of the entire peer-reviewed literature. The persistent dominance of the WiCell lines H1 and H9 in research also points to a potential weakness with respect to universal applicability of the results, standardization, and harmonization in the field. The collection and dissemination of a wide range of data, including negative data, for as many hESC lines as possible (for example, by international registries such as hESCreg, the European Human Embryonic Stem Cell Registry) may be one option for the diversification of hESC use. International registries may then provide the answer to researchers as to whether it makes a difference which cell line to use, or to choose the best cell line for their research or application.
