# Supplementary material for: Human Embryonic Stem Cell Lines and Their Use in International Research
Source: Stem Cells. 2010 Feb;28(2):240–6. doi: 10.1002/stem.286 (PMC2952289; doi:10.1002/stem.286)
Supplement: Supplementary file 1 [file stem0028-0240-SD1.doc]

**Supplementary Methods**

Determination of publications reporting work on hESCs or hiPSCs

*The strings for searching the PubMed database (*[*http://www.ncbi.nlm.nih.gov/sites/entrez?db=PubMed*](http://www.ncbi.nlm.nih.gov/sites/entrez?db=PubMed)*) for papers on hESC work were the same as published previously (2):*

*human embryonic stem cell*[All Fields] AND (stem cells[MeSH Terms] OR stem cell transplantation[MeSH Terms]) AND Year[Publication Date] AND english[Language]*

*human es cell*[All Fields] AND (stem cells[MeSH Terms] OR stem cell transplantation[MeSH Terms]) AND Year[Publication Date] AND english[Language]*

*hesc[All Fields] AND (stem cells[MeSH Terms] OR stem cell transplantation[MeSH Terms]) AND Year[Publication Date] AND english[Language]*

*hes cell*[All Fields] AND (stem cells[MeSH Terms] OR stem cell transplantation[MeSH Terms]) AND Year[Publication Date] AND english[Language]*

*human pluripotent stem cell*[All Fields] AND (stem cells[MeSH Terms] OR stem cell transplantation[MeSH Terms]) AND Year[Publication Date] AND english[Language]*

*(embryonic stem cell*[All Fields] OR es cell*[All Fields]) AND (stem cells[MeSH Terms] OR stem cell transplantation[MeSH Terms]) AND Year[Publication Date] AND english[Language] AND (humans[MeSH Terms] OR human*[All Fields])*

*pluripoten*[All Fields] AND (stem cells[MeSH Terms] OR stem cell transplantation[MeSH Terms] OR stem cell*[All Fields]) AND Year[Publication Date] AND english[Language] AND (humans[MeSH Terms] OR human*[All Fields])*

*Year[Publication Date] AND (human embryonic stem cell*[All Fields] OR human es cell*[All Fields] OR hesc[All Fields] OR hes cell*[All Fields] OR human pluripotent stem cell*[All Fields]) AND english[Language]*

Searches for years until 2004 were performed in October 2005, search for the other years were performed by mid of the following year. In case of printed journals, the appearance date of the print edition was considered as relevant. Papers published ahead of print in 2008 but were not available in print by end of 2008 were not included.

Search of the PubMed database for papers on experimental work on human induced pluripotent stem cells (hiPSCs) was performed on September 30^th^ 2009 using the following string:

*(induced pluripotent stem[All Fields] OR (ips cell[All Fields] OR ips cells[All Fields]) OR iPSC[All Fields] OR iPSCs[All Fields]) AND Year[PublicationDate] AND english[Language] NOT review[PublicationType] NOT editorial[PublicationType] NOT comment[PublicationType] NOT inhibitory postsynaptic current*[All Fields]*

Hits were manually evaluated to select for papers that indeed report novel experimental work on hESCs and hiPSCs, respectively.

Determination of average Impact Factors

In 2009, of 229 Journals that have published experimental work on hESCs 9 have not been judged by the Institute for Scientific Information (ISI) affecting 28 papers extracted by our search method. These papers were not included in the following analyses of impact factors. The Five Year Impact Factor for a respective journal that had published experimental hESC research papers was multiplied by the number of papers that had appeared in this journal. The results were added and divided by the total paper numbers to obtain the average Impact Factors.

Determination of citation frequencies

All hESC papers extracted by our analysis were checked for their overall citation frequency as well as for citation frequency in 2008 using the Scopus database (<http://www.scopus.com/home.url>). Analysis was performed by July 2009.

Determination of international cooperation in hESC work

Determination of Authors Affiliation was performed by analysis of the respective papers. Papers with author’s affiliations in a single country were regarded as national studies. Papers produced by authors form at least two different countries were regarded as international cooperation.

Determination of NIH or other governmental funding of hESC work

Acknowledgements and other relevant sections of papers were screened for funding information. Studies produced under involvement of authors with an affiliation at a NIH institute were regarded as NIH funded.

Determination of hESC line numbers

Information on hESC lines reported by 2005 was based on data published previously (2). Information on novel hESC lines were collected from the scientific literature identified by the method described above. Furthermore, the following registries and stem cell banks were scanned for novel hESC lines:

- hESC registry of the National Institutes of Health (NIH) (<http://stemcells.nih.gov/research/registry/>)
- hESC registry of the International Society of Stem Cell Research (ISSCR) (<http://www.isscr.org/science/sclines.htm>)
- hESC registry of the International Stem Cell Forum (ISCF) (<http://www.stemcellforum.org/isci_project/the_registry.cfm>)
- hESC registry of the European Union (hESC^reg^) (<http://www.hescreg.eu/>)
- hESC registry of the Stem Cell Community (<http://www.stemcellcommunity.org/>)
- International Stem Cell Registry of the University of Massachusetts (UMass) Medical School (<http://www.umassmed.edu/scri/>)
- National Stem Cell Bank (NSCB) at WiCell Institute (<http://www.nationalstemcellbank.org/>)
- The Stem Cell Bank of the UK Medical Research Council (MRC) (<http://www.ukstemcellbank.org.uk/>)
- The Singapore Stem Cell Bank (SSCB) of the Singapore Stem Cell Consortium (SSCC) (<http://www.sscc.a-star.edu.sg/stemCellBank.php>)
- StemRide internationa hES Cell Bank der Firma (<http://www.stemride.com/Stem_Cell_Bank.htm>)

Public information on novel hESCs released in the press was obtained by continuously examining (for more than five years) Google News Alerts containing the term *stem cell* or *stem cells*.
