# Supplementary material for: Human Embryonic Stem Cell Lines and Their Use in International Research
Source: Stem Cells. 2010 Feb;28(2):240–6. doi: 10.1002/stem.286 (PMC2952289; doi:10.1002/stem.286)
Supplement: Supplementary file 2 [file stem0028-0240-SD2.doc]

| **Country** | **Number of Publications**  **(2000-2007 )** | **Average Journal Impact Factor** | **Citations in 2008** | **Average Citation Frequency in 2008** |
| --- | --- | --- | --- | --- |
| **Australia** | **32** | **6,952** | **377** | **11.8** |
| **Canada** | **16** | **10,769** | **278** | **17.4** |
| **China** | **26** | **4,041** | **172** | **6.6** |
| **Germany** | **12** | **6,406** | **111** | **9.2** |
| **Israel** | **61** | **6,631** | **1192** | **19.5** |
| **Japan** | **16** | **7,498** | **182** | **11.4** |
| **Korea** | **51** | **4,425** | **363** | **7.1** |
| **Singapore** | **42** | **5,538** | **438** | **10.4** |
| **Sweden** | **39** | **4,768** | **314** | **8.1** |
| **UK** | **65** | **7,161** | **969** | **14.9** |
| **USA** | **278** | **9,123** | **5426** | **19.5** |
| **Total (all countries)** | **696** | **7.422** | **10373** | **14.9** |

**Supplementary Table 1** Impact of hESC research from selected countries. Given are the numbers of articles reporting original hESC work published between 2000 and 2007, the average 5 year (2004-2008) ISI impact factor of journals that published said articles, and their absolute and average citation frequencies. To prevent an undue influence on the figures of an article frequently cited outside the experimental stem cell field, pioneering work on hESCs (Thomson et al., Science, 282:1145-1147, 1998) was excluded from the analysis. Pioneering work on hiPSCs in which hESCs or hESC-derived materials were used solely for comparison (Takahashi et al., Cell 131:861-872, 2007, and Yu et al., Science 318:1917-1920, 2007) was also omitted from this analysis for the same reason.
