# Supplementary material for: Human Embryonic Stem Cell Lines and Their Use in International Research
Source: Stem Cells. 2010 Feb;28(2):240–6. doi: 10.1002/stem.286 (PMC2952289; doi:10.1002/stem.286)
Supplement: Supplementary file 3 [file stem0028-0240-SD3.doc]

| **Scientific paper** | **Times Cited (2008)** | **Times Cited**  **(1998 to 2008)** | **Country of Corresponding Author** |
| --- | --- | --- | --- |
| Thomson, J. A. et al. *Science* 282, 1145 (1998)* | 530 | 2870 | United States |
| Boyer, L. A. et al. *Cell* 122, 947 (2005) | 225 | 513 | United States |
| Lee, T. I. et al. *Cell* 125, 301-313 (2006)* | 160 | 357 | United States |
| Reubinoff, B. E. et al., *Nat. Biotechnol.* 18, 399 (2000)* | 155 | 1002 | Australia |
| Sato, N. et al., *Nat. Med.* 10, 55 (2004)* | 122 | 426 | United States |
| Guenther, M. G. et al., *Cell* 130, 77 (2007) | 122 | 141 | United States |
| Xu, C. H. et al., *Nat. Biotechnol.* 19, 971 (2001) | 101 | 539 | United States |
| D'Amour, K. A. et al. *Nat. Biotechnol.* 24, 1392 (2006) | 97 | 154 | United States |
| Kehat, I. et al., *J. Clin. Invest.* 108, 407 (2001) | 94 | 570 | Israel |
| Zhang, S. C. et al. *Nat. Biotechnol.* 19, 1129 (2001)* | 83 | 504 | United States |
| Draper, J. S. et al. *Nat. Biotechnol.* 22, 53 ( 2004)* | 80 | 298 | United Kingdom |
| Ludwig, T. E. et al. *Nat. Biotechnol.* 24, 185 (2006) | 80 | 177 | United States |
| Cowan, C. A. et al. *N. Engl. J. Med.* 350, 1353 (2004) | 79 | 300 | United States |
| Cowan, C. A. et al. *Science* 309, 369 (2005) | 79 | 207 | United States |
| Amit, M. et al. *Dev. Biol.* 227, 271 (2000)* | 74 | 496 | Israel |
| Reubinoff, B. E. et al., *Nat. Biotechnol.* 19, 1134 (2001)* | 74 | 413 | Israel |
| Xu, R. H. et al., *Nat. Meth.* 2, 185 (2005) | 74 | 262 | United States |
| D'Amour, K. A. et al., *Nat. Biotechnol.* 23, 1534 (2005) | 74 | 162 | United States |
| Laflamme, M. A. et al., *Nat. Biotechnol.* 25, 1015 (2007) | 72 | 79 | United States |
| Perrier, A. L. et al., *Proc. Natl. Acad. Sci. USA* 101, 12543 (2004) | 69 | 211 | United States |

**Supplementary Table 2** hESC research papers cited most frequently in the scientific literature in 2008. Citation frequencies are also given for the period 1998-2008. Analysis was performed using the Scopus data base in July 2008. Papers on hiPSCs in which hESCs or hESC derived materials were used solely for comparison were not rated.

* Scientists from at least two countries were involved in the study.
