# Supplementary material for: Human Embryonic Stem Cell Lines and Their Use in International Research
Source: Stem Cells. 2010 Feb;28(2):240–6. doi: 10.1002/stem.286 (PMC2952289; doi:10.1002/stem.286)
Supplement: Supplementary file 4 [file stem0028-0240-SD4.pdf]

Overview on Publicly Reported hESC Lines

Arranged by Country of Origin.

AUSTRALIA

Provider: Prince of Wales Hospital, Sidney, & Illawarra Area Health Service, NSW, Australia

hESC line: **Endeavour-1 (E1)**

special features: derived in serum free medium
derived on human feeder cells

published in a peer-reviewed journal: yes

characterization data reported in a peer-reviewed journal: yes

reference: Sidhu, K. S. et al. Stem Cells Dev 17, 41-51 (2008)

---

hESC line: **Endeavour-2**

special features:

published in a peer-reviewed journal: no

characterization data reported in a peer-reviewed journal: no

reference: European Union hESC registry (http://www.hescreg.eu/)

Provider: Sidney IVF Ltd.

hESC line: **SIVF01**

special features:

published in a peer-reviewed journal: no

characterization data reported in a peer-reviewed journal: no

reference: European Union hESC registry (http://www.hescreg.eu/)

---

hESC line: **SIVF02**

special features:

published in a peer-reviewed journal: no

characterization data reported in a peer-reviewed journal: no

reference: European Union hESC registry (http://www.hescreg.eu/)

---

hESC line: **SIVF03**

special features: abnormal karyotype
derived on human feeder cells

published in a peer-reviewed journal:	yes
characterization data reported in a peer-reviewed journal:	yes
reference:	Peura, T. T. et al., Theriogenology 67, 32-42 (2007)<br>Peura, T. et al., Cloning Stem Cells 10, 203-216 (2008)

hESC line:	**SIVF04**
special features:	derived on human feeder cells
published in a peer-reviewed journal:	yes
characterization data reported in a peer-reviewed journal:	yes
reference:	Peura, T. T. et al., Theriogenology 67, 32-42 (2007)<br>Peura, T. et al., Cloning Stem Cells 10, 203-216 (2008)

hESC line:	**SIVF05**
special features:	derived on human feeder cells
published in a peer-reviewed journal:	yes
characterization data reported in a peer-reviewed journal:	yes
reference:	Peura, T. T. et al., Theriogenology 67, 32-42 (2007)<br>Peura, T. et al., Cloning Stem Cells 10, 203-216 (2008)

hESC line:	**SIVF06**
special features:	derived on human feeder cells
published in a peer-reviewed journal:	yes
characterization data reported in a peer-reviewed journal:	yes
reference:	Peura, T. T. et al., Theriogenology 67, 32-42 (2007)<br>Peura, T. et al., Cloning Stem Cells 10, 203-216 (2008)

hESC line:	**SIVF07**
special features:	derived on human feeder cells
published in a peer-reviewed journal:	yes
characterization data reported in a peer-reviewed journal:	yes
reference:	Peura, T. T. et al., Theriogenology 67, 32-42 (2007)<br>Peura, T. et al., Cloning Stem Cells 10, 203-216 (2008)

hESC line:	**SIVF08**
special features:	derived on human feeder cells

published in a peer-reviewed journal:	yes
characterization data reported in a peer-reviewed journal:	yes
reference:	Peura, T. T. et al., Theriogenology 67, 32-42 (2007)<br>Peura, T. et al., Cloning Stem Cells 10, 203-216 (2008)

hESC line:	**SIVF09**
special features:	abnormal karyotype<br>derived on human feeder cells
published in a peer-reviewed journal:	yes
characterization data reported in a peer-reviewed journal:	yes
reference:	Peura, T. T. et al., Theriogenology 67, 32-42 (2007)<br>Peura, T. et al., Cloning Stem Cells 10, 203-216 (2008)

hESC line:	**SIVF10**
special features:	derived on human feeder cells
published in a peer-reviewed journal:	yes
characterization data reported in a peer-reviewed journal:	yes
reference:	Peura, T. T. et al., Theriogenology 67, 32-42 (2007)<br>Peura, T. et al., Cloning Stem Cells 10, 203-216 (2008)

hESC line:	**SIVF11**
special features:	abnormal karyotype<br>derived on human feeder cells
published in a peer-reviewed journal:	yes
characterization data reported in a peer-reviewed journal:	yes
reference:	Peura, T. T. et al., Theriogenology 67, 32-42 (2007)<br>Peura, T. et al., Cloning Stem Cells 10, 203-216 (2008)

hESC line:	**SIVF12**
special features:	derived on human feeder cells
published in a peer-reviewed journal:	yes
characterization data reported in a peer-reviewed journal:	yes
reference:	Peura, T. et al., Cloning Stem Cells 10, 203-216 (2008)

hESC line:	**SIVF13**
special features:	derived on human feeder cells

published in a peer-reviewed journal:	yes
characterization data reported in a peer-reviewed journal:	yes
reference:	Peura, T. et al., Cloning Stem Cells 10, 203-216 (2008)

Provider: Stem Cell Sciences Ltd, Australia und Australian Stem Cell Centre (ASCC)

hESC line:	**MEL-1**
special features:	
published in a peer-reviewed journal:	yes
characterization data reported in a peer-reviewed journal:	yes
reference:	Press Conference Bio 2005, Philadelphia (http://www2.prnewswire.com/cgi-bin/micro_stories.pl?ACCT=638464&TICK=BIO05AIC&STORY=/www/story/06-19-2005/0003916442&EDATE=Jun+19,+2005)<br>Adewumi, O. et al. Nat Biotechnol 25, 803-816 (2007)

hESC line:	**MEL-2**
special features:	
published in a peer-reviewed journal:	yes
characterization data reported in a peer-reviewed journal:	yes
reference:	Press Conference Bio 2005, Philadelphia (http://www2.prnewswire.com/cgi-bin/micro_stories.pl?ACCT=638464&TICK=BIO05AIC&STORY=/www/story/06-19-2005/0003916442&EDATE=Jun+19,+2005)<br>Adewumi, O. et al. Nat Biotechnol 25, 803-816 (2007)

hESC line:	**MEL-3**
special features:	
published in a peer-reviewed journal:	no
characterization data reported in a peer-reviewed journal:	no
reference:	European Union hESC registry (http://www.hescreg.eu/)

hESC line:	**MEL-4**
special features:	

published in a peer-reviewed journal:	no
characterization data reported in a peer-reviewed journal:	no
reference:	European Union hESC registry (http://www.hescreg.eu/)

BELGIUM

Provider: Université Libre de Bruxelles, Belgium

hESC line:	**ERA1**
special features:	derived by whole embryo culture<br>derived in serum free medium<br>derived on human feeder cells
published in a peer-reviewed journal:	yes
characterization data reported in a peer-reviewed journal:	yes
reference:	Deleu, S. et al. Reprod Biomed Online 18, 704-716 (2009)

hESC line:	**ERA2**
special features:	derived by whole embryo culture<br>derived in serum free medium<br>derived on human feeder cells
published in a peer-reviewed journal:	yes
characterization data reported in a peer-reviewed journal:	yes
reference:	Deleu, S. et al. Reprod Biomed Online 18, 704-716 (2009)

hESC line:	**ERA3**
special features:	derived by whole embryo culture<br>derived in serum free medium<br>derived on human feeder cells
published in a peer-reviewed journal:	yes
characterization data reported in a peer-reviewed journal:	yes
reference:	Deleu, S. et al. Reprod Biomed Online 18, 704-716 (2009)

hESC line:	**ERAMUC1**
special features:	derived by whole embryo culture<br>derived in serum free medium<br>derived on human feeder cells<br>genetic disorder: Cystic fibrosis (CF)

published in a peer-reviewed journal:	yes
characterization data reported in a peer-reviewed journal:	yes
reference:	Deleu, S. et al. Reprod Biomed Online 18, 704-716 (2009)

hESC line:	**ERAMUC2**
special features:	derived in serum free medium<br>derived on human feeder cells<br>genetic disorder: Cystic fibrosis (CF)
published in a peer-reviewed journal:	yes
characterization data reported in a peer-reviewed journal:	yes
reference:	Deleu, S. et al. Reprod Biomed Online 18, 704-716 (2009)

Provider: Vrije Universiteit Brussel

hESC line:	**DEMI**
special features:	
published in a peer-reviewed journal:	no
characterization data reported in a peer-reviewed journal:	no
reference:	Arrêté du 19 août 2005 portant autorisation d'importation de cellules souches embryonnaires humaines (http://www.dsi.cnrs.fr/bo/2005/10-05/textessig-bo1005.htm)

hESC line:	**IGOR**
special features:	
published in a peer-reviewed journal:	no
characterization data reported in a peer-reviewed journal:	no
reference:	Arrêté du 19 août 2005 portant autorisation d'importation de cellules souches embryonnaires humaines (http://www.dsi.cnrs.fr/bo/2005/10-05/textessig-bo1005.htm)

hESC line:	**VUB01**
special features:	
published in a peer-reviewed journal:	yes
characterization data reported in a peer-reviewed journal:	yes
reference:	Mateizel, I. et al. Hum. Reprod.. 21, 503-511 (2006)

hESC line: **VUB02**

special features: derived in serum free medium

published in a peer-reviewed journal: yes

characterization data reported in a peer-reviewed journal: yes

reference: Mateizel, I. et al. Hum. Reprod.. 21, 503-511 (2006)

---

hESC line: **VUB03_DM1**

special features: derived in serum free medium
genetic disorder: Dystrophya myotonica type 1 (DM1)

published in a peer-reviewed journal: yes

characterization data reported in a peer-reviewed journal: yes

reference: Sermon, K. D. et al. Hum Reprod 20 (suppl 1), i5-i7 (2005)
Mateizel, I. et al. Hum. Reprod.. 21, 503-511 (2006)

---

hESC line: **VUB04_CF**

special features: derived in serum free medium
genetic disorder: Cystic fibrosis (CF)

published in a peer-reviewed journal: yes

characterization data reported in a peer-reviewed journal: yes

reference: Sermon, K. D. et al. Hum Reprod 20 (suppl 1), i5-i7 (2005)
Mateizel, I. et al. Hum. Reprod.. 21, 503-511 (2006)

---

hESC line: **VUB05_HD**

special features: derived in serum free medium
genetic disorder: Huntington's desease (HD)

published in a peer-reviewed journal: yes

characterization data reported in a peer-reviewed journal: yes

reference: Sermon, K. D. et al. Hum Reprod 20 (suppl 1), i5-i7 (2005)
Mateizel, I. et al. Hum. Reprod.. 21, 503-511 (2006)

---

hESC line: **VUB06**

special features: derived in serum free medium

published in a peer-reviewed journal:	yes
characterization data reported in a peer-reviewed journal:	yes
reference:	Spits, C. et al. Nat Biotechnol 26, 1361-1363 (2008)

hESC line:	**VUB07**
special features:	derived in serum free medium
published in a peer-reviewed journal:	yes
characterization data reported in a peer-reviewed journal:	yes
reference:	Spits, C. et al. Nat Biotechnol 26, 1361-1363 (2008)

hESC line:	**VUB08_MFS**
special features:	derived in serum free medium<br>genetic disorder: Marfan syndrome (MFS)
published in a peer-reviewed journal:	yes
characterization data reported in a peer-reviewed journal:	yes
reference:	Spits, C. et al. Nat Biotechnol 26, 1361-1363 (2008)

hESC line:	**VUB09_FSHD**
special features:	derived in serum free medium<br>genetic disorder: Facio Scapulo Humeral (FSH) muscular dystrophy
published in a peer-reviewed journal:	yes
characterization data reported in a peer-reviewed journal:	yes
reference:	Spits, C. et al. Nat Biotechnol 26, 1361-1363 (2008)

hESC line:	**VUB10_SCA7**
special features:	derived in serum free medium<br>genetic disorder: Spinocerebellar Ataxia Type 7 (SCA7)
published in a peer-reviewed journal:	no
characterization data reported in a peer-reviewed journal:	no
reference:	European Union hESC registry (http://www.hescreg.eu/)

hESC line:	**VUB11_FXS**
special features:	derived in serum free medium<br>genetic disorder: Fragile X syndrome (FX)

published in a peer-reviewed journal:	yes
characterization data reported in a peer-reviewed journal:	yes
reference:	Spits, C. et al. Nat Biotechnol 26, 1361-1363 (2008)

---

hESC line:	**VUB13_FXS**
special features:	derived in serum free medium<br>genetic disorder: Fragile X syndrome (FX)
published in a peer-reviewed journal:	yes
characterization data reported in a peer-reviewed journal:	yes
reference:	Spits, C. et al. Nat Biotechnol 26, 1361-1363 (2008)

---

hESC line:	**VUB15**
special features:	derived in serum free medium
published in a peer-reviewed journal:	yes
characterization data reported in a peer-reviewed journal:	yes
reference:	Spits, C. et al. Nat Biotechnol 26, 1361-1363 (2008)

---

hESC line:	**VUB17**
special features:	derived in serum free medium
published in a peer-reviewed journal:	yes
characterization data reported in a peer-reviewed journal:	yes
reference:	Spits, C. et al. Nat Biotechnol 26, 1361-1363 (2008)

---

hESC line:	**VUB19_DM1**
special features:	derived in serum free medium<br>genetic disorder: Dystrophya myotonica type 1 (DM1)
published in a peer-reviewed journal:	yes
characterization data reported in a peer-reviewed journal:	yes
reference:	Spits, C. et al. Nat Biotechnol 26, 1361-1363 (2008)

---

hESC line:	**VUB20_CMT1A**
special features:	abnormal karyotype<br>derived in serum free medium<br>genetic disorder: Charcot-Marie-Tooth-Disease 1A (CMT1A)

published in a peer-reviewed journal:	yes
characterization data reported in a peer-reviewed journal:	yes
reference:	Spits, C. et al. Nat Biotechnol 26, 1361-1363 (2008)

hESC line:	**VUB22_CF**
special features:	genetic disorder: Cystic fibrosis (CF)
published in a peer-reviewed journal:	no
characterization data reported in a peer-reviewed journal:	no
reference:	European Union hESC registry (http://www.hescreg.eu/)

hESC line:	**VUB23_OI**
special features:	genetic disorder: Osteogenesis imperfecta type 1
published in a peer-reviewed journal:	no
characterization data reported in a peer-reviewed journal:	no
reference:	European Union hESC registry (http://www.hescreg.eu/)

hESC line:	**VUB24_DM1**
special features:	derived in serum free medium<br>genetic disorder: Dystrophya myotonica type 1 (DM1)
published in a peer-reviewed journal:	yes
characterization data reported in a peer-reviewed journal:	yes
reference:	Spits, C. et al. Nat Biotechnol 26, 1361-1363 (2008)

hESC line:	**VUB26 (VUB26_QUATRO)**
special features:	abnormal karyotype<br>derived from research embryo<br>derived from single blastomere<br>derived in serum free medium
published in a peer-reviewed journal:	yes
characterization data reported in a peer-reviewed journal:	yes
reference:	Spits, C. et al. Nat Biotechnol 26, 1361-1363 (2008)<br>Geens, M. et al. Human Reprod, published online ahead of print July 24th 2009

hESC line:	**VUB27_Patru**
special features:	derived from research embryo<br>derived from single blastomere<br>derived in serum free medium
published in a peer-reviewed journal:	yes
characterization data reported in a peer-reviewed journal:	yes
reference:	Geens, M. et al. Human Reprod, published online ahead of print July 24th 2009

hESC line:	**VUB28_HD_MFS**
special features:	genetic disorder: Huntington's desease (HD)<br>genetic disorder: Marfan syndrome (MFS)
published in a peer-reviewed journal:	no
characterization data reported in a peer-reviewed journal:	no
reference:	European Union hESC registry (http://www.hescreg.eu/)

BRAZIL

Provider: Laboratório Nacional de Células-Tronco Embrionárias, Sao Paulo, Brazil

hESC line:	**BR-1**
special features:	
published in a peer-reviewed journal:	no
characterization data reported in a peer-reviewed journal:	no
reference:	UMA-Stem Cell Registry (http://www.umassmed.edu/iscr/index.aspx)

CANADA

Provider: Mount Sinai Hospital, Toronto

hESC line:	**CA1**
special features:	
published in a peer-reviewed journal:	yes
characterization data reported in a peer-reviewed journal:	yes
reference:	Press Release of the University of Toronto, 2005/06/10 (http://www.news.utoronto.ca/bin6/050610-1439.asp)<br>Adewumi, O. et al. Nat Biotechnol 25, 803-816 (2007)

hESC line:	**CA2**
special features:	

published in a peer-reviewed journal: yes

characterization data reported in a peer-reviewed journal: yes

reference: Press Release of the University of Toronto, 2005/06/10 (http://www.news.utoronto.ca/bin6/050610-1439.asp)
Adewumi, O. et al. Nat Biotechnol 25, 803-816 (2007)

Provider: University of Calgery

hESC line: **CC1**

special features:

published in a peer-reviewed journal: no

characterization data reported in a peer-reviewed journal: no

reference: UK Stem Cell Bank approved Cell Lines (http://www.mrc.ac.uk/consumption/groups/public/documents/content/mrc003259.pdf)

---

hESC line: **CC2**

special features:

published in a peer-reviewed journal: no

characterization data reported in a peer-reviewed journal: no

reference: UK Stem Cell Bank approved Cell Lines (http://www.mrc.ac.uk/consumption/groups/public/documents/content/mrc003259.pdf)

CHINA

Provider: Central South University, Changsha, China

hESC line: **chHES10**

special features: derived in serum free medium
derived on human feeder cells
mechanical ICM isolation

published in a peer-reviewed journal: yes

characterization data reported in a peer-reviewed journal: yes

reference: Lin, G. et al. Cell Stem Cell 2009 5, 461-465 (2009)

---

hESC line: **chHES100**

special features: derived in serum free medium
derived on human feeder cells
mechanical ICM isolation

published in a peer-reviewed journal:	yes
characterization data reported in a peer-reviewed journal:	yes
reference:	Lin, G. et al. Cell Stem Cell 2009 5, 461-465 (2009)

---

hESC line:	**chHES101**
special features:	derived in serum free medium<br>derived on human feeder cells<br>mechanical ICM isolation
published in a peer-reviewed journal:	yes
characterization data reported in a peer-reviewed journal:	yes
reference:	Lin, G. et al. Cell Stem Cell 2009 5, 461-465 (2009)

---

hESC line:	**chHES102**
special features:	derived in serum free medium<br>derived on human feeder cells<br>mechanical ICM isolation
published in a peer-reviewed journal:	yes
characterization data reported in a peer-reviewed journal:	yes
reference:	Lin, G. et al. Cell Stem Cell 2009 5, 461-465 (2009)

---

hESC line:	**chHES103**
special features:	derived in serum free medium<br>derived on human feeder cells<br>mechanical ICM isolation
published in a peer-reviewed journal:	yes
characterization data reported in a peer-reviewed journal:	yes
reference:	Lin, G. et al. Cell Stem Cell 2009 5, 461-465 (2009)

---

hESC line:	**chHES104**
special features:	derived in serum free medium<br>derived on human feeder cells<br>mechanical ICM isolation

published in a peer-reviewed journal:	yes
characterization data reported in a peer-reviewed journal:	yes
reference:	Lin, G. et al. Cell Stem Cell 2009 5, 461-465 (2009)

hESC line:	**chHES105**
special features:	abnormal karyotype<br>derived in serum free medium<br>derived on human feeder cells<br>mechanical ICM isolation
published in a peer-reviewed journal:	yes
characterization data reported in a peer-reviewed journal:	yes
reference:	Lin, G. et al. Cell Stem Cell 2009 5, 461-465 (2009)

hESC line:	**chHES106**
special features:	derived in serum free medium<br>derived on human feeder cells<br>mechanical ICM isolation
published in a peer-reviewed journal:	yes
characterization data reported in a peer-reviewed journal:	yes
reference:	Lin, G. et al. Cell Stem Cell 2009 5, 461-465 (2009)

hESC line:	**chHES107**
special features:	abnormal karyotype<br>derived in serum free medium<br>derived on human feeder cells<br>mechanical ICM isolation
published in a peer-reviewed journal:	yes
characterization data reported in a peer-reviewed journal:	yes
reference:	Lin, G. et al. Cell Stem Cell 2009 5, 461-465 (2009)

hESC line:	**chHES108**
special features:	derived in serum free medium<br>derived on human feeder cells<br>mechanical ICM isolation

published in a peer-reviewed journal: yes

characterization data reported in a peer-reviewed journal: yes

reference: Lin, G. et al. Cell Stem Cell 2009 5, 461-465 (2009)

---

hESC line: **chHES109**

special features: abnormal karyotype
derived in serum free medium
derived on human feeder cells
mechanical ICM isolation

published in a peer-reviewed journal: yes

characterization data reported in a peer-reviewed journal: yes

reference: Lin, G. et al. Cell Stem Cell 2009 5, 461-465 (2009)

---

hESC line: **chHES11**

special features: derived in serum free medium
derived on human feeder cells
mechanical ICM isolation

published in a peer-reviewed journal: yes

characterization data reported in a peer-reviewed journal: yes

reference: Lin, G. et al. Cell Stem Cell 2009 5, 461-465 (2009)

---

hESC line: **chHES110**

special features: derived in serum free medium
derived on human feeder cells
mechanical ICM isolation

published in a peer-reviewed journal: yes

characterization data reported in a peer-reviewed journal: yes

reference: Lin, G. et al. Cell Stem Cell 2009 5, 461-465 (2009)

---

hESC line: **chHES111**

special features: derived in serum free medium
derived on human feeder cells
mechanical ICM isolation

published in a peer-reviewed journal:	yes
characterization data reported in a peer-reviewed journal:	yes
reference:	Lin, G. et al. Cell Stem Cell 2009 5, 461-465 (2009)

---

hESC line:	**chHES112**
special features:	derived in serum free medium<br>derived on human feeder cells<br>mechanical ICM isolation
published in a peer-reviewed journal:	yes
characterization data reported in a peer-reviewed journal:	yes
reference:	Lin, G. et al. Cell Stem Cell 2009 5, 461-465 (2009)

---

hESC line:	**chHES113**
special features:	derived in serum free medium<br>derived on human feeder cells<br>mechanical ICM isolation
published in a peer-reviewed journal:	yes
characterization data reported in a peer-reviewed journal:	yes
reference:	Lin, G. et al. Cell Stem Cell 2009 5, 461-465 (2009)

---

hESC line:	**chHES114**
special features:	derived in serum free medium<br>derived on human feeder cells<br>mechanical ICM isolation
published in a peer-reviewed journal:	yes
characterization data reported in a peer-reviewed journal:	yes
reference:	Lin, G. et al. Cell Stem Cell 2009 5, 461-465 (2009)

---

hESC line:	**chHES115**
special features:	derived in serum free medium<br>derived on human feeder cells<br>mechanical ICM isolation

published in a peer-reviewed journal:	yes
characterization data reported in a peer-reviewed journal:	yes
reference:	Lin, G. et al. Cell Stem Cell 2009 5, 461-465 (2009)

hESC line:	**chHES116**
special features:	derived in serum free medium<br>derived on human feeder cells<br>mechanical ICM isolation
published in a peer-reviewed journal:	yes
characterization data reported in a peer-reviewed journal:	yes
reference:	Lin, G. et al. Cell Stem Cell 2009 5, 461-465 (2009)

hESC line:	**chHES117**
special features:	derived in serum free medium<br>derived on human feeder cells<br>mechanical ICM isolation
published in a peer-reviewed journal:	yes
characterization data reported in a peer-reviewed journal:	yes
reference:	Lin, G. et al. Cell Stem Cell 2009 5, 461-465 (2009)

hESC line:	**chHES118**
special features:	derived in serum free medium<br>derived on human feeder cells<br>mechanical ICM isolation
published in a peer-reviewed journal:	yes
characterization data reported in a peer-reviewed journal:	yes
reference:	Lin, G. et al. Cell Stem Cell 2009 5, 461-465 (2009)

hESC line:	**chHES119**
special features:	abnormal karyotype<br>derived in serum free medium<br>derived on human feeder cells<br>mechanical ICM isolation

published in a peer-reviewed journal:	yes
characterization data reported in a peer-reviewed journal:	yes
reference:	Lin, G. et al. Cell Stem Cell 2009 5, 461-465 (2009)

hESC line:	**chHES12**
special features:	derived in serum free medium<br>derived on human feeder cells<br>mechanical ICM isolation
published in a peer-reviewed journal:	yes
characterization data reported in a peer-reviewed journal:	yes
reference:	Lin, G. et al. Cell Stem Cell 2009 5, 461-465 (2009)

hESC line:	**chHES120**
special features:	derived in serum free medium<br>derived on human feeder cells<br>mechanical ICM isolation
published in a peer-reviewed journal:	yes
characterization data reported in a peer-reviewed journal:	yes
reference:	Lin, G. et al. Cell Stem Cell 2009 5, 461-465 (2009)

hESC line:	**chHES121**
special features:	derived in serum free medium<br>derived on human feeder cells<br>mechanical ICM isolation
published in a peer-reviewed journal:	yes
characterization data reported in a peer-reviewed journal:	yes
reference:	Lin, G. et al. Cell Stem Cell 2009 5, 461-465 (2009)

hESC line:	**chHES122**
special features:	abnormal karyotype<br>derived in serum free medium<br>derived on human feeder cells<br>mechanical ICM isolation

published in a peer-reviewed journal:	yes
characterization data reported in a peer-reviewed journal:	yes
reference:	Lin, G. et al. Cell Stem Cell 2009 5, 461-465 (2009)

---

hESC line:	**chHES123**
special features:	abnormal karyotype<br>derived in serum free medium<br>derived on human feeder cells<br>mechanical ICM isolation
published in a peer-reviewed journal:	yes
characterization data reported in a peer-reviewed journal:	yes
reference:	Lin, G. et al. Cell Stem Cell 2009 5, 461-465 (2009)

---

hESC line:	**chHES124**
special features:	derived in serum free medium<br>derived on human feeder cells<br>mechanical ICM isolation
published in a peer-reviewed journal:	yes
characterization data reported in a peer-reviewed journal:	yes
reference:	Lin, G. et al. Cell Stem Cell 2009 5, 461-465 (2009)

---

hESC line:	**chHES125**
special features:	derived in serum free medium<br>derived on human feeder cells<br>mechanical ICM isolation
published in a peer-reviewed journal:	yes
characterization data reported in a peer-reviewed journal:	yes
reference:	Lin, G. et al. Cell Stem Cell 2009 5, 461-465 (2009)

---

hESC line:	**chHES126**
special features:	derived in serum free medium<br>derived on human feeder cells<br>mechanical ICM isolation

published in a peer-reviewed journal:	yes
characterization data reported in a peer-reviewed journal:	yes
reference:	Lin, G. et al. Cell Stem Cell 2009 5, 461-465 (2009)

---

hESC line:	**chHES127**
special features:	derived in serum free medium<br>derived on human feeder cells<br>mechanical ICM isolation
published in a peer-reviewed journal:	yes
characterization data reported in a peer-reviewed journal:	yes
reference:	Lin, G. et al. Cell Stem Cell 2009 5, 461-465 (2009)

---

hESC line:	**chHES128**
special features:	derived in serum free medium<br>derived on human feeder cells<br>mechanical ICM isolation
published in a peer-reviewed journal:	yes
characterization data reported in a peer-reviewed journal:	yes
reference:	Lin, G. et al. Cell Stem Cell 2009 5, 461-465 (2009)

---

hESC line:	**chHES129**
special features:	derived in serum free medium<br>derived on human feeder cells<br>mechanical ICM isolation
published in a peer-reviewed journal:	yes
characterization data reported in a peer-reviewed journal:	yes
reference:	Lin, G. et al. Cell Stem Cell 2009 5, 461-465 (2009)

---

hESC line:	**chHES13**
special features:	derived in serum free medium<br>derived on human feeder cells<br>mechanical ICM isolation

published in a peer-reviewed journal:	yes
characterization data reported in a peer-reviewed journal:	yes
reference:	Lin, G. et al. Cell Stem Cell 2009 5, 461-465 (2009)

---

hESC line:	**chHES130**
special features:	derived in serum free medium<br>derived on human feeder cells<br>mechanical ICM isolation
published in a peer-reviewed journal:	yes
characterization data reported in a peer-reviewed journal:	yes
reference:	Lin, G. et al. Cell Stem Cell 2009 5, 461-465 (2009)

---

hESC line:	**chHES131**
special features:	derived in serum free medium<br>derived on human feeder cells<br>mechanical ICM isolation
published in a peer-reviewed journal:	yes
characterization data reported in a peer-reviewed journal:	yes
reference:	Lin, G. et al. Cell Stem Cell 2009 5, 461-465 (2009)

---

hESC line:	**chHES132**
special features:	derived in serum free medium<br>derived on human feeder cells<br>mechanical ICM isolation
published in a peer-reviewed journal:	yes
characterization data reported in a peer-reviewed journal:	yes
reference:	Lin, G. et al. Cell Stem Cell 2009 5, 461-465 (2009)

---

hESC line:	**chHES134**
special features:	derived in serum free medium<br>derived on human feeder cells<br>mechanical ICM isolation

published in a peer-reviewed journal:	yes
characterization data reported in a peer-reviewed journal:	yes
reference:	Lin, G. et al. Cell Stem Cell 2009 5, 461-465 (2009)

---

hESC line:	**chHES136**
special features:	abnormal karyotype<br>derived in serum free medium<br>derived on human feeder cells<br>mechanical ICM isolation
published in a peer-reviewed journal:	yes
characterization data reported in a peer-reviewed journal:	yes
reference:	Lin, G. et al. Cell Stem Cell 2009 5, 461-465 (2009)

---

hESC line:	**chHES137**
special features:	derived in serum free medium<br>derived on human feeder cells<br>mechanical ICM isolation
published in a peer-reviewed journal:	yes
characterization data reported in a peer-reviewed journal:	yes
reference:	Lin, G. et al. Cell Stem Cell 2009 5, 461-465 (2009)

---

hESC line:	**chHES138**
special features:	derived in serum free medium<br>derived on human feeder cells<br>mechanical ICM isolation
published in a peer-reviewed journal:	yes
characterization data reported in a peer-reviewed journal:	yes
reference:	Lin, G. et al. Cell Stem Cell 2009 5, 461-465 (2009)

---

hESC line:	**chHES139**
special features:	derived in serum free medium<br>derived on human feeder cells<br>mechanical ICM isolation

published in a peer-reviewed journal:	yes
characterization data reported in a peer-reviewed journal:	yes
reference:	Lin, G. et al. Cell Stem Cell 2009 5, 461-465 (2009)

hESC line:	**chHES14**
special features:	abnormal karyotype<br>derived in serum free medium<br>derived on human feeder cells<br>mechanical ICM isolation
published in a peer-reviewed journal:	yes
characterization data reported in a peer-reviewed journal:	yes
reference:	Lin, G. et al. Cell Stem Cell 2009 5, 461-465 (2009)

hESC line:	**chHES140**
special features:	derived in serum free medium<br>derived on human feeder cells<br>mechanical ICM isolation
published in a peer-reviewed journal:	yes
characterization data reported in a peer-reviewed journal:	yes
reference:	Lin, G. et al. Cell Stem Cell 2009 5, 461-465 (2009)

hESC line:	**chHES141**
special features:	derived in serum free medium<br>derived on human feeder cells<br>mechanical ICM isolation
published in a peer-reviewed journal:	yes
characterization data reported in a peer-reviewed journal:	yes
reference:	Lin, G. et al. Cell Stem Cell 2009 5, 461-465 (2009)

hESC line:	**chHES142**
special features:	derived in serum free medium<br>derived on human feeder cells<br>mechanical ICM isolation

published in a peer-reviewed journal:	yes
characterization data reported in a peer-reviewed journal:	yes
reference:	Lin, G. et al. Cell Stem Cell 2009 5, 461-465 (2009)

hESC line:	**chHES143**
special features:	derived in serum free medium<br>derived on human feeder cells<br>mechanical ICM isolation
published in a peer-reviewed journal:	yes
characterization data reported in a peer-reviewed journal:	yes
reference:	Lin, G. et al. Cell Stem Cell 2009 5, 461-465 (2009)

hESC line:	**chHES144**
special features:	derived in serum free medium<br>derived on human feeder cells<br>mechanical ICM isolation
published in a peer-reviewed journal:	yes
characterization data reported in a peer-reviewed journal:	yes
reference:	Lin, G. et al. Cell Stem Cell 2009 5, 461-465 (2009)

hESC line:	**chHES145**
special features:	abnormal karyotype<br>derived in serum free medium<br>derived on human feeder cells<br>mechanical ICM isolation
published in a peer-reviewed journal:	yes
characterization data reported in a peer-reviewed journal:	yes
reference:	Lin, G. et al. Cell Stem Cell 2009 5, 461-465 (2009)

published in a peer-reviewed journal:	yes
characterization data reported in a peer-reviewed journal:	yes
reference:	Lin, G. et al. Cell Stem Cell 2009 5, 461-465 (2009)

hESC line:	**chHES146**
special features:	abnormal karyotype<br>derived in serum free medium<br>derived on human feeder cells<br>mechanical ICM isolation
published in a peer-reviewed journal:	yes
characterization data reported in a peer-reviewed journal:	yes
reference:	Lin, G. et al. Cell Stem Cell 2009 5, 461-465 (2009)

hESC line:	**chHES147**
special features:	derived in serum free medium<br>derived on human feeder cells<br>mechanical ICM isolation
published in a peer-reviewed journal:	yes
characterization data reported in a peer-reviewed journal:	yes
reference:	Lin, G. et al. Cell Stem Cell 2009 5, 461-465 (2009)

hESC line:	**chHES148**
special features:	derived in serum free medium<br>derived on human feeder cells<br>mechanical ICM isolation
published in a peer-reviewed journal:	yes
characterization data reported in a peer-reviewed journal:	yes
reference:	Lin, G. et al. Cell Stem Cell 2009 5, 461-465 (2009)

hESC line:	**chHES149**
special features:	derived in serum free medium<br>derived on human feeder cells<br>mechanical ICM isolation
published in a peer-reviewed journal:	yes
characterization data reported in a peer-reviewed journal:	yes
reference:	Lin, G. et al. Cell Stem Cell 2009 5, 461-465 (2009)

hESC line:	**chHES15**
special features:	derived in serum free medium<br>derived on human feeder cells<br>mechanical ICM isolation

published in a peer-reviewed journal:	yes
characterization data reported in a peer-reviewed journal:	yes
reference:	Lin, G. et al. Cell Stem Cell 2009 5, 461-465 (2009)

hESC line:	**chHES150**
special features:	derived in serum free medium<br>derived on human feeder cells<br>mechanical ICM isolation
published in a peer-reviewed journal:	yes
characterization data reported in a peer-reviewed journal:	yes
reference:	Lin, G. et al. Cell Stem Cell 2009 5, 461-465 (2009)

hESC line:	**chHES151**
special features:	abnormal karyotype<br>derived in serum free medium<br>derived on human feeder cells<br>mechanical ICM isolation
published in a peer-reviewed journal:	yes
characterization data reported in a peer-reviewed journal:	yes
reference:	Lin, G. et al. Cell Stem Cell 2009 5, 461-465 (2009)

hESC line:	**chHES152**
special features:	abnormal karyotype<br>derived in serum free medium<br>derived on human feeder cells<br>mechanical ICM isolation
published in a peer-reviewed journal:	yes
characterization data reported in a peer-reviewed journal:	yes
reference:	Lin, G. et al. Cell Stem Cell 2009 5, 461-465 (2009)

hESC line:	**chHES153**
special features:	derived in serum free medium<br>derived on human feeder cells<br>mechanical ICM isolation

published in a peer-reviewed journal: yes

characterization data reported in a peer-reviewed journal: yes

reference: Lin, G. et al. Cell Stem Cell 2009 5, 461-465 (2009)

---

hESC line: **chHES156**

special features: derived in serum free medium
derived on human feeder cells
mechanical ICM isolation

published in a peer-reviewed journal: yes

characterization data reported in a peer-reviewed journal: yes

reference: Lin, G. et al. Cell Stem Cell 2009 5, 461-465 (2009)

---

hESC line: **chHES157**

special features: derived in serum free medium
derived on human feeder cells
mechanical ICM isolation

published in a peer-reviewed journal: yes

characterization data reported in a peer-reviewed journal: yes

reference: Lin, G. et al. Cell Stem Cell 2009 5, 461-465 (2009)

---

hESC line: **chHES158**

special features: abnormal karyotype
derived in serum free medium
derived on human feeder cells
mechanical ICM isolation

published in a peer-reviewed journal: yes

characterization data reported in a peer-reviewed journal: yes

reference: Lin, G. et al. Cell Stem Cell 2009 5, 461-465 (2009)

---

hESC line: **chHES16**

special features: derived in serum free medium
derived on human feeder cells
mechanical ICM isolation

published in a peer-reviewed journal:	yes
characterization data reported in a peer-reviewed journal:	yes
reference:	Lin, G. et al. Cell Stem Cell 2009 5, 461-465 (2009)

---

hESC line:	**chHES160**
special features:	derived in serum free medium<br>derived on human feeder cells<br>mechanical ICM isolation
published in a peer-reviewed journal:	yes
characterization data reported in a peer-reviewed journal:	yes
reference:	Lin, G. et al. Cell Stem Cell 2009 5, 461-465 (2009)

---

hESC line:	**chHES161**
special features:	derived in serum free medium<br>derived on human feeder cells<br>mechanical ICM isolation
published in a peer-reviewed journal:	yes
characterization data reported in a peer-reviewed journal:	yes
reference:	Lin, G. et al. Cell Stem Cell 2009 5, 461-465 (2009)

---

hESC line:	**chHES162**
special features:	derived in serum free medium<br>derived on human feeder cells<br>mechanical ICM isolation
published in a peer-reviewed journal:	yes
characterization data reported in a peer-reviewed journal:	yes
reference:	Lin, G. et al. Cell Stem Cell 2009 5, 461-465 (2009)

---

hESC line:	**chHES163**
special features:	derived in serum free medium<br>derived on human feeder cells<br>mechanical ICM isolation

published in a peer-reviewed journal:	yes
characterization data reported in a peer-reviewed journal:	yes
reference:	Lin, G. et al. Cell Stem Cell 2009 5, 461-465 (2009)

---

hESC line:	**chHES164**
special features:	abnormal karyotype<br>derived in serum free medium<br>derived on human feeder cells<br>mechanical ICM isolation
published in a peer-reviewed journal:	yes
characterization data reported in a peer-reviewed journal:	yes
reference:	Lin, G. et al. Cell Stem Cell 2009 5, 461-465 (2009)

---

hESC line:	**chHES165**
special features:	abnormal karyotype<br>derived in serum free medium<br>derived on human feeder cells<br>mechanical ICM isolation
published in a peer-reviewed journal:	yes
characterization data reported in a peer-reviewed journal:	yes
reference:	Lin, G. et al. Cell Stem Cell 2009 5, 461-465 (2009)

---

hESC line:	**chHES166**
special features:	derived in serum free medium<br>derived on human feeder cells<br>mechanical ICM isolation
published in a peer-reviewed journal:	yes
characterization data reported in a peer-reviewed journal:	yes
reference:	Lin, G. et al. Cell Stem Cell 2009 5, 461-465 (2009)

---

hESC line:	**chHES167**
special features:	derived in serum free medium<br>derived on human feeder cells<br>mechanical ICM isolation

published in a peer-reviewed journal:	yes
characterization data reported in a peer-reviewed journal:	yes
reference:	Lin, G. et al. Cell Stem Cell 2009 5, 461-465 (2009)

---

hESC line:	**chHES168**
special features:	derived in serum free medium<br>derived on human feeder cells<br>mechanical ICM isolation
published in a peer-reviewed journal:	yes
characterization data reported in a peer-reviewed journal:	yes
reference:	Lin, G. et al. Cell Stem Cell 2009 5, 461-465 (2009)

---

hESC line:	**chHES169**
special features:	derived in serum free medium<br>derived on human feeder cells<br>mechanical ICM isolation
published in a peer-reviewed journal:	yes
characterization data reported in a peer-reviewed journal:	yes
reference:	Lin, G. et al. Cell Stem Cell 2009 5, 461-465 (2009)

---

hESC line:	**chHES17**
special features:	derived in serum free medium<br>derived on human feeder cells<br>mechanical ICM isolation
published in a peer-reviewed journal:	yes
characterization data reported in a peer-reviewed journal:	yes
reference:	Lin, G. et al. Cell Stem Cell 2009 5, 461-465 (2009)

---

hESC line:	**cHHES170**
special features:	derived in serum free medium<br>derived on human feeder cells<br>mechanical ICM isolation

published in a peer-reviewed journal:	yes
characterization data reported in a peer-reviewed journal:	yes
reference:	Lin, G. et al. Cell Stem Cell 2009 5, 461-465 (2009)

hESC line:	**chHES171**
special features:	derived in serum free medium<br>derived on human feeder cells<br>mechanical ICM isolation
published in a peer-reviewed journal:	yes
characterization data reported in a peer-reviewed journal:	yes
reference:	Lin, G. et al. Cell Stem Cell 2009 5, 461-465 (2009)

hESC line:	**chHES172**
special features:	derived in serum free medium<br>derived on human feeder cells<br>mechanical ICM isolation
published in a peer-reviewed journal:	yes
characterization data reported in a peer-reviewed journal:	yes
reference:	Lin, G. et al. Cell Stem Cell 2009 5, 461-465 (2009)

hESC line:	**chHES173**
special features:	derived in serum free medium<br>derived on human feeder cells<br>mechanical ICM isolation
published in a peer-reviewed journal:	yes
characterization data reported in a peer-reviewed journal:	yes
reference:	Lin, G. et al. Cell Stem Cell 2009 5, 461-465 (2009)

hESC line:	**chHES174**
special features:	derived in serum free medium<br>derived on human feeder cells<br>mechanical ICM isolation

published in a peer-reviewed journal:	yes
characterization data reported in a peer-reviewed journal:	yes
reference:	Lin, G. et al. Cell Stem Cell 2009 5, 461-465 (2009)

hESC line:	**chHES175**
special features:	derived in serum free medium<br>derived on human feeder cells<br>mechanical ICM isolation
published in a peer-reviewed journal:	yes
characterization data reported in a peer-reviewed journal:	yes
reference:	Lin, G. et al. Cell Stem Cell 2009 5, 461-465 (2009)

hESC line:	**chHES176**
special features:	derived in serum free medium<br>derived on human feeder cells<br>mechanical ICM isolation
published in a peer-reviewed journal:	yes
characterization data reported in a peer-reviewed journal:	yes
reference:	Lin, G. et al. Cell Stem Cell 2009 5, 461-465 (2009)

hESC line:	**chHES177**
special features:	derived in serum free medium<br>derived on human feeder cells<br>mechanical ICM isolation
published in a peer-reviewed journal:	yes
characterization data reported in a peer-reviewed journal:	yes
reference:	Lin, G. et al. Cell Stem Cell 2009 5, 461-465 (2009)

hESC line:	**chHES178**
special features:	derived in serum free medium<br>derived on human feeder cells<br>mechanical ICM isolation

published in a peer-reviewed journal:	yes
characterization data reported in a peer-reviewed journal:	yes
reference:	Lin, G. et al. Cell Stem Cell 2009 5, 461-465 (2009)

hESC line:	**chHES179**
special features:	derived in serum free medium<br>derived on human feeder cells<br>mechanical ICM isolation
published in a peer-reviewed journal:	yes
characterization data reported in a peer-reviewed journal:	yes
reference:	Lin, G. et al. Cell Stem Cell 2009 5, 461-465 (2009)

hESC line:	**chHES18**
special features:	derived in serum free medium<br>derived on human feeder cells<br>mechanical ICM isolation
published in a peer-reviewed journal:	yes
characterization data reported in a peer-reviewed journal:	yes
reference:	Lin, G. et al. Cell Stem Cell 2009 5, 461-465 (2009)

hESC line:	**chHES180**
special features:	derived in serum free medium<br>derived on human feeder cells<br>mechanical ICM isolation
published in a peer-reviewed journal:	yes
characterization data reported in a peer-reviewed journal:	yes
reference:	Lin, G. et al. Cell Stem Cell 2009 5, 461-465 (2009)

hESC line:	**chHES181**
special features:	derived in serum free medium<br>derived on human feeder cells<br>mechanical ICM isolation

published in a peer-reviewed journal: yes

characterization data reported in a peer-reviewed journal: yes

reference: Lin, G. et al. Cell Stem Cell 2009 5, 461-465 (2009)

---

hESC line: **chHES182**

special features: derived in serum free medium
derived on human feeder cells
mechanical ICM isolation

published in a peer-reviewed journal: yes

characterization data reported in a peer-reviewed journal: yes

reference: Lin, G. et al. Cell Stem Cell 2009 5, 461-465 (2009)

---

hESC line: **chHES183**

special features: derived in serum free medium
derived on human feeder cells
mechanical ICM isolation

published in a peer-reviewed journal: yes

characterization data reported in a peer-reviewed journal: yes

reference: Lin, G. et al. Cell Stem Cell 2009 5, 461-465 (2009)

---

hESC line: **chHES184**

special features: derived in serum free medium
derived on human feeder cells
mechanical ICM isolation

published in a peer-reviewed journal: yes

characterization data reported in a peer-reviewed journal: yes

reference: Lin, G. et al. Cell Stem Cell 2009 5, 461-465 (2009)

---

hESC line: **chHES185**

special features: derived in serum free medium
derived on human feeder cells
mechanical ICM isolation

published in a peer-reviewed journal:	yes
characterization data reported in a peer-reviewed journal:	yes
reference:	Lin, G. et al. Cell Stem Cell 2009 5, 461-465 (2009)

hESC line:	**chHES186**
special features:	derived in serum free medium<br>derived on human feeder cells<br>mechanical ICM isolation
published in a peer-reviewed journal:	yes
characterization data reported in a peer-reviewed journal:	yes
reference:	Lin, G. et al. Cell Stem Cell 2009 5, 461-465 (2009)

hESC line:	**chHES187**
special features:	derived in serum free medium<br>derived on human feeder cells<br>mechanical ICM isolation
published in a peer-reviewed journal:	yes
characterization data reported in a peer-reviewed journal:	yes
reference:	Lin, G. et al. Cell Stem Cell 2009 5, 461-465 (2009)

hESC line:	**chHES188**
special features:	abnormal karyotype<br>derived in serum free medium<br>derived on human feeder cells<br>mechanical ICM isolation
published in a peer-reviewed journal:	yes
characterization data reported in a peer-reviewed journal:	yes
reference:	Lin, G. et al. Cell Stem Cell 2009 5, 461-465 (2009)

hESC line:	**chHES189**
special features:	derived in serum free medium<br>derived on human feeder cells<br>mechanical ICM isolation

published in a peer-reviewed journal:	yes
characterization data reported in a peer-reviewed journal:	yes
reference:	Lin, G. et al. Cell Stem Cell 2009 5, 461-465 (2009)

published in a peer-reviewed journal:	yes
characterization data reported in a peer-reviewed journal:	yes
reference:	Lin, G. et al. Cell Stem Cell 2009 5, 461-465 (2009)

hESC line:	**chHES19**
special features:	abnormal karyotype<br>derived in serum free medium<br>derived on human feeder cells<br>mechanical ICM isolation
published in a peer-reviewed journal:	yes
characterization data reported in a peer-reviewed journal:	yes
reference:	Lin, G. et al. Cell Stem Cell 2009 5, 461-465 (2009)

hESC line:	**chHES190**
special features:	derived in serum free medium<br>derived on human feeder cells<br>mechanical ICM isolation
published in a peer-reviewed journal:	yes
characterization data reported in a peer-reviewed journal:	yes
reference:	Lin, G. et al. Cell Stem Cell 2009 5, 461-465 (2009)

hESC line:	**chHES192**
special features:	derived in serum free medium<br>derived on human feeder cells<br>mechanical ICM isolation
published in a peer-reviewed journal:	yes
characterization data reported in a peer-reviewed journal:	yes
reference:	Lin, G. et al. Cell Stem Cell 2009 5, 461-465 (2009)

hESC line:	**chHES193**
special features:	derived in serum free medium<br>derived on human feeder cells<br>mechanical ICM isolation
published in a peer-reviewed journal:	yes
characterization data reported in a peer-reviewed journal:	yes
reference:	Lin, G. et al. Cell Stem Cell 2009 5, 461-465 (2009)

---

hESC line:	**chHES194**
special features:	derived in serum free medium<br>derived on human feeder cells<br>mechanical ICM isolation
published in a peer-reviewed journal:	yes
characterization data reported in a peer-reviewed journal:	yes
reference:	Lin, G. et al. Cell Stem Cell 2009 5, 461-465 (2009)

---

hESC line:	**chHES195**
special features:	derived in serum free medium<br>derived on human feeder cells<br>mechanical ICM isolation
published in a peer-reviewed journal:	yes
characterization data reported in a peer-reviewed journal:	yes
reference:	Lin, G. et al. Cell Stem Cell 2009 5, 461-465 (2009)

---

hESC line:	**chHES20**
special features:	derived in serum free medium<br>derived on human feeder cells<br>mechanical ICM isolation
published in a peer-reviewed journal:	yes
characterization data reported in a peer-reviewed journal:	yes
reference:	Zhou, J. et al. Stem Cells Dev 17, 737-749 (2008)

---

hESC line:	**chHES21**
special features:	derived in serum free medium<br>derived on human feeder cells<br>mechanical ICM isolation

published in a peer-reviewed journal:	yes
characterization data reported in a peer-reviewed journal:	yes
reference:	Lin, G. et al. Cell Stem Cell 2009 5, 461-465 (2009)

hESC line:	**chHES22**
special features:	derived in serum free medium<br>derived on human feeder cells<br>mechanical ICM isolation
published in a peer-reviewed journal:	yes
characterization data reported in a peer-reviewed journal:	yes
reference:	Zhou, J. et al. Stem Cells Dev 17, 737-749 (2008)

hESC line:	**chHES23**
special features:	derived in serum free medium<br>derived on human feeder cells<br>mechanical ICM isolation
published in a peer-reviewed journal:	yes
characterization data reported in a peer-reviewed journal:	yes
reference:	Lin, G. et al. Cell Stem Cell 2009 5, 461-465 (2009)

hESC line:	**chHES24**
special features:	abnormal karyotype<br>derived in serum free medium<br>derived on human feeder cells<br>mechanical ICM isolation
published in a peer-reviewed journal:	yes
characterization data reported in a peer-reviewed journal:	yes
reference:	Lin, G. et al. Cell Stem Cell 2009 5, 461-465 (2009)

hESC line:	**chHES25**
special features:	derived in serum free medium<br>derived on human feeder cells<br>mechanical ICM isolation

published in a peer-reviewed journal: yes

characterization data reported in a peer-reviewed journal: yes

reference: Lin, G. et al. Cell Stem Cell 2009 5, 461-465 (2009)

---

hESC line: **chHES26**

special features: derived in serum free medium
derived on human feeder cells
mechanical ICM isolation

published in a peer-reviewed journal: yes

characterization data reported in a peer-reviewed journal: yes

reference: Lin, G. et al. Cell Stem Cell 2009 5, 461-465 (2009)

---

hESC line: **chHES27**

special features: derived in serum free medium
derived on human feeder cells
mechanical ICM isolation

published in a peer-reviewed journal: yes

characterization data reported in a peer-reviewed journal: yes

reference: Lin, G. et al. Cell Stem Cell 2009 5, 461-465 (2009)

---

hESC line: **chHES28**

special features: derived in serum free medium
derived on human feeder cells
mechanical ICM isolation

published in a peer-reviewed journal: yes

characterization data reported in a peer-reviewed journal: yes

reference: Lin, G. et al. Cell Stem Cell 2009 5, 461-465 (2009)

---

hESC line: **chHES29**

special features: derived in serum free medium
derived on human feeder cells
mechanical ICM isolation

published in a peer-reviewed journal:	yes
characterization data reported in a peer-reviewed journal:	yes
reference:	Lin, G. et al. Cell Stem Cell 2009 5, 461-465 (2009)

hESC line:	**chHES3 (formerly chESC-3, H3)**
special features:	
published in a peer-reviewed journal:	yes
characterization data reported in a peer-reviewed journal:	yes
reference:	Wang, J. et al. Cell Biol Int 29, 654-661 (2005)<br>Yang, S. et al. Genes Chromosomes Cancer 47, 665-679 (2008)

hESC line:	**chHES30**
special features:	derived in serum free medium<br>derived on human feeder cells<br>mechanical ICM isolation
published in a peer-reviewed journal:	yes
characterization data reported in a peer-reviewed journal:	yes
reference:	Lin, G. et al. Cell Stem Cell 2009 5, 461-465 (2009)

hESC line:	**chHES31**
special features:	derived in serum free medium<br>derived on human feeder cells<br>mechanical ICM isolation
published in a peer-reviewed journal:	yes
characterization data reported in a peer-reviewed journal:	yes
reference:	Lin, G. et al. Cell Stem Cell 2009 5, 461-465 (2009)

hESC line:	**chHES32**
special features:	derived in serum free medium<br>derived on human feeder cells<br>mechanical ICM isolation
published in a peer-reviewed journal:	yes
characterization data reported in a peer-reviewed journal:	yes
reference:	Lin, G. et al. Cell Stem Cell 2009 5, 461-465 (2009)

hESC line:	**chHES33**
special features:	abnormal karyotype<br>derived in serum free medium<br>derived on human feeder cells<br>mechanical ICM isolation
published in a peer-reviewed journal:	yes
characterization data reported in a peer-reviewed journal:	yes
reference:	Lin, G. et al. Cell Stem Cell 2009 5, 461-465 (2009)

---

hESC line:	**chHES34**
special features:	derived in serum free medium<br>derived on human feeder cells<br>mechanical ICM isolation
published in a peer-reviewed journal:	yes
characterization data reported in a peer-reviewed journal:	yes
reference:	Lin, G. et al. Cell Stem Cell 2009 5, 461-465 (2009)

---

hESC line:	**chHES35**
special features:	derived in serum free medium<br>derived on human feeder cells<br>mechanical ICM isolation
published in a peer-reviewed journal:	yes
characterization data reported in a peer-reviewed journal:	yes
reference:	Lin, G. et al. Cell Stem Cell 2009 5, 461-465 (2009)

---

hESC line:	**chHES36**
special features:	derived in serum free medium<br>derived on human feeder cells<br>mechanical ICM isolation
published in a peer-reviewed journal:	yes
characterization data reported in a peer-reviewed journal:	yes
reference:	Lin, G. et al. Cell Stem Cell 2009 5, 461-465 (2009)

---

hESC line:	**chHES37**
special features:	abnormal karyotype<br>derived in serum free medium<br>derived on human feeder cells<br>mechanical ICM isolation
published in a peer-reviewed journal:	yes
characterization data reported in a peer-reviewed journal:	yes
reference:	Lin, G. et al. Cell Stem Cell 2009 5, 461-465 (2009)

---

hESC line:	**chHES38**
special features:	derived in serum free medium<br>derived on human feeder cells<br>mechanical ICM isolation
published in a peer-reviewed journal:	yes
characterization data reported in a peer-reviewed journal:	yes
reference:	Lin, G. et al. Cell Stem Cell 2009 5, 461-465 (2009)

---

hESC line:	**chHES39**
special features:	derived in serum free medium<br>derived on human feeder cells<br>mechanical ICM isolation
published in a peer-reviewed journal:	yes
characterization data reported in a peer-reviewed journal:	yes
reference:	Lin, G. et al. Cell Stem Cell 2009 5, 461-465 (2009)

---

hESC line:	**chHES40**
special features:	derived in serum free medium<br>derived on human feeder cells<br>mechanical ICM isolation
published in a peer-reviewed journal:	yes
characterization data reported in a peer-reviewed journal:	yes
reference:	Lin, G. et al. Cell Stem Cell 2009 5, 461-465 (2009)

---

hESC line:	**chHES41**
special features:	derived in serum free medium<br>derived on human feeder cells<br>mechanical ICM isolation

published in a peer-reviewed journal:	yes
characterization data reported in a peer-reviewed journal:	yes
reference:	Lin, G. et al. Cell Stem Cell 2009 5, 461-465 (2009)

hESC line:	**chHES42**
special features:	derived in serum free medium<br>derived on human feeder cells<br>mechanical ICM isolation
published in a peer-reviewed journal:	yes
characterization data reported in a peer-reviewed journal:	yes
reference:	Lin, G. et al. Cell Stem Cell 2009 5, 461-465 (2009)

hESC line:	**chHES43**
special features:	derived in serum free medium<br>derived on human feeder cells<br>mechanical ICM isolation
published in a peer-reviewed journal:	yes
characterization data reported in a peer-reviewed journal:	yes
reference:	Lin, G. et al. Cell Stem Cell 2009 5, 461-465 (2009)

hESC line:	**chHES44**
special features:	derived in serum free medium<br>derived on human feeder cells<br>mechanical ICM isolation
published in a peer-reviewed journal:	yes
characterization data reported in a peer-reviewed journal:	yes
reference:	Lin, G. et al. Cell Stem Cell 2009 5, 461-465 (2009)

hESC line:	**chHES45**
special features:	derived in serum free medium<br>derived on human feeder cells<br>mechanical ICM isolation

published in a peer-reviewed journal:	yes
characterization data reported in a peer-reviewed journal:	yes
reference:	Lin, G. et al. Cell Stem Cell 2009 5, 461-465 (2009)

hESC line:	**chHES46**
special features:	abnormal karyotype<br>derived in serum free medium<br>derived on human feeder cells<br>mechanical ICM isolation
published in a peer-reviewed journal:	yes
characterization data reported in a peer-reviewed journal:	yes
reference:	Lin, G. et al. Cell Stem Cell 2009 5, 461-465 (2009)

hESC line:	**chHES47**
special features:	derived in serum free medium<br>derived on human feeder cells<br>mechanical ICM isolation
published in a peer-reviewed journal:	yes
characterization data reported in a peer-reviewed journal:	yes
reference:	Lin, G. et al. Cell Stem Cell 2009 5, 461-465 (2009)

hESC line:	**chHES48**
special features:	abnormal karyotype<br>derived in serum free medium<br>derived on human feeder cells<br>mechanical ICM isolation
published in a peer-reviewed journal:	yes
characterization data reported in a peer-reviewed journal:	yes
reference:	Lin, G. et al. Cell Stem Cell 2009 5, 461-465 (2009)

hESC line:	**chHES49**
special features:	derived in serum free medium<br>derived on human feeder cells<br>mechanical ICM isolation

published in a peer-reviewed journal:	yes
characterization data reported in a peer-reviewed journal:	yes
reference:	Lin, G. et al. Cell Stem Cell 2009 5, 461-465 (2009)

hESC line:	**chHES50**
special features:	derived in serum free medium<br>derived on human feeder cells<br>mechanical ICM isolation
published in a peer-reviewed journal:	yes
characterization data reported in a peer-reviewed journal:	yes
reference:	Lin, G. et al. Cell Stem Cell 2009 5, 461-465 (2009)

hESC line:	**chHES51**
special features:	derived in serum free medium<br>derived on human feeder cells<br>mechanical ICM isolation
published in a peer-reviewed journal:	yes
characterization data reported in a peer-reviewed journal:	yes
reference:	Lin, G. et al. Cell Stem Cell 2009 5, 461-465 (2009)

hESC line:	**chHES52**
special features:	derived in serum free medium<br>derived on human feeder cells<br>mechanical ICM isolation
published in a peer-reviewed journal:	yes
characterization data reported in a peer-reviewed journal:	yes
reference:	Lin, G. et al. Cell Stem Cell 2009 5, 461-465 (2009)

hESC line:	**chHES53**
special features:	derived in serum free medium<br>derived on human feeder cells<br>mechanical ICM isolation

published in a peer-reviewed journal:	yes
characterization data reported in a peer-reviewed journal:	yes
reference:	Lin, G. et al. Cell Stem Cell 2009 5, 461-465 (2009)

---

hESC line:	**chHES54**
special features:	derived in serum free medium<br>derived on human feeder cells<br>mechanical ICM isolation
published in a peer-reviewed journal:	yes
characterization data reported in a peer-reviewed journal:	yes
reference:	Lin, G. et al. Cell Stem Cell 2009 5, 461-465 (2009)

---

hESC line:	**chHES55**
special features:	derived in serum free medium<br>derived on human feeder cells<br>mechanical ICM isolation
published in a peer-reviewed journal:	yes
characterization data reported in a peer-reviewed journal:	yes
reference:	Lin, G. et al. Cell Stem Cell 2009 5, 461-465 (2009)

---

hESC line:	**chHES56**
special features:	derived in serum free medium<br>derived on human feeder cells<br>mechanical ICM isolation
published in a peer-reviewed journal:	yes
characterization data reported in a peer-reviewed journal:	yes
reference:	Lin, G. et al. Cell Stem Cell 2009 5, 461-465 (2009)

---

hESC line:	**chHES57**
special features:	derived in serum free medium<br>derived on human feeder cells<br>mechanical ICM isolation

published in a peer-reviewed journal:	yes
characterization data reported in a peer-reviewed journal:	yes
reference:	Lin, G. et al. Cell Stem Cell 2009 5, 461-465 (2009)

---

hESC line:	**chHES58**
special features:	derived in serum free medium<br>derived on human feeder cells<br>mechanical ICM isolation
published in a peer-reviewed journal:	yes
characterization data reported in a peer-reviewed journal:	yes
reference:	Lin, G. et al. Cell Stem Cell 2009 5, 461-465 (2009)

---

hESC line:	**chHES59**
special features:	derived in serum free medium<br>derived on human feeder cells<br>mechanical ICM isolation
published in a peer-reviewed journal:	yes
characterization data reported in a peer-reviewed journal:	yes
reference:	Lin, G. et al. Cell Stem Cell 2009 5, 461-465 (2009)

---

hESC line:	**chHES60**
special features:	derived in serum free medium<br>derived on human feeder cells<br>mechanical ICM isolation
published in a peer-reviewed journal:	yes
characterization data reported in a peer-reviewed journal:	yes
reference:	Lin, G. et al. Cell Stem Cell 2009 5, 461-465 (2009)

---

hESC line:	**chHES61**
special features:	derived in serum free medium<br>derived on human feeder cells<br>mechanical ICM isolation

published in a peer-reviewed journal:	yes
characterization data reported in a peer-reviewed journal:	yes
reference:	Lin, G. et al. Cell Stem Cell 2009 5, 461-465 (2009)

hESC line:	**chHES62**
special features:	derived in serum free medium<br>derived on human feeder cells<br>mechanical ICM isolation
published in a peer-reviewed journal:	yes
characterization data reported in a peer-reviewed journal:	yes
reference:	Lin, G. et al. Cell Stem Cell 2009 5, 461-465 (2009)

hESC line:	**chHES63**
special features:	derived in serum free medium<br>derived on human feeder cells<br>mechanical ICM isolation
published in a peer-reviewed journal:	yes
characterization data reported in a peer-reviewed journal:	yes
reference:	Lin, G. et al. Cell Stem Cell 2009 5, 461-465 (2009)

hESC line:	**chHES64**
special features:	derived in serum free medium<br>derived on human feeder cells<br>mechanical ICM isolation
published in a peer-reviewed journal:	yes
characterization data reported in a peer-reviewed journal:	yes
reference:	Lin, G. et al. Cell Stem Cell 2009 5, 461-465 (2009)

hESC line:	**chHES65**
special features:	derived in serum free medium<br>derived on human feeder cells<br>mechanical ICM isolation

published in a peer-reviewed journal:	yes
characterization data reported in a peer-reviewed journal:	yes
reference:	Lin, G. et al. Cell Stem Cell 2009 5, 461-465 (2009)

---

hESC line:	**chHES66**
special features:	derived in serum free medium<br>derived on human feeder cells<br>mechanical ICM isolation
published in a peer-reviewed journal:	yes
characterization data reported in a peer-reviewed journal:	yes
reference:	Lin, G. et al. Cell Stem Cell 2009 5, 461-465 (2009)

---

hESC line:	**chHES67**
special features:	derived in serum free medium<br>derived on human feeder cells<br>mechanical ICM isolation
published in a peer-reviewed journal:	yes
characterization data reported in a peer-reviewed journal:	yes
reference:	Lin, G. et al. Cell Stem Cell 2009 5, 461-465 (2009)

---

hESC line:	**chHES68**
special features:	derived in serum free medium<br>derived on human feeder cells<br>mechanical ICM isolation
published in a peer-reviewed journal:	yes
characterization data reported in a peer-reviewed journal:	yes
reference:	Lin, G. et al. Cell Stem Cell 2009 5, 461-465 (2009)

---

hESC line:	**chHES69**
special features:	derived in serum free medium<br>derived on human feeder cells<br>mechanical ICM isolation

published in a peer-reviewed journal:	yes
characterization data reported in a peer-reviewed journal:	yes
reference:	Lin, G. et al. Cell Stem Cell 2009 5, 461-465 (2009)

hESC line:	**chHES70**
special features:	derived in serum free medium<br>derived on human feeder cells<br>mechanical ICM isolation
published in a peer-reviewed journal:	yes
characterization data reported in a peer-reviewed journal:	yes
reference:	Lin, G. et al. Cell Stem Cell 2009 5, 461-465 (2009)

hESC line:	**chHES71**
special features:	derived in serum free medium<br>derived on human feeder cells<br>mechanical ICM isolation
published in a peer-reviewed journal:	yes
characterization data reported in a peer-reviewed journal:	yes
reference:	Lin, G. et al. Cell Stem Cell 2009 5, 461-465 (2009)

hESC line:	**chHES72**
special features:	derived in serum free medium<br>derived on human feeder cells<br>mechanical ICM isolation
published in a peer-reviewed journal:	yes
characterization data reported in a peer-reviewed journal:	yes
reference:	Lin, G. et al. Cell Stem Cell 2009 5, 461-465 (2009)

hESC line:	**chHES73**
special features:	derived in serum free medium<br>derived on human feeder cells<br>mechanical ICM isolation

published in a peer-reviewed journal:	yes
characterization data reported in a peer-reviewed journal:	yes
reference:	Lin, G. et al. Cell Stem Cell 2009 5, 461-465 (2009)

hESC line:	**chHES74**
special features:	derived in serum free medium<br>derived on human feeder cells<br>mechanical ICM isolation
published in a peer-reviewed journal:	yes
characterization data reported in a peer-reviewed journal:	yes
reference:	Lin, G. et al. Cell Stem Cell 2009 5, 461-465 (2009)

hESC line:	**chHES75**
special features:	derived in serum free medium<br>derived on human feeder cells<br>mechanical ICM isolation
published in a peer-reviewed journal:	yes
characterization data reported in a peer-reviewed journal:	yes
reference:	Lin, G. et al. Cell Stem Cell 2009 5, 461-465 (2009)

hESC line:	**chHES76**
special features:	derived in serum free medium<br>derived on human feeder cells<br>mechanical ICM isolation
published in a peer-reviewed journal:	yes
characterization data reported in a peer-reviewed journal:	yes
reference:	Lin, G. et al. Cell Stem Cell 2009 5, 461-465 (2009)

hESC line:	**chHES77**
special features:	derived in serum free medium<br>derived on human feeder cells<br>mechanical ICM isolation

published in a peer-reviewed journal:	yes
characterization data reported in a peer-reviewed journal:	yes
reference:	Lin, G. et al. Cell Stem Cell 2009 5, 461-465 (2009)

hESC line:	**chHES78**
special features:	derived in serum free medium<br>derived on human feeder cells<br>mechanical ICM isolation
published in a peer-reviewed journal:	yes
characterization data reported in a peer-reviewed journal:	yes
reference:	Lin, G. et al. Cell Stem Cell 2009 5, 461-465 (2009)

hESC line:	**chHES79**
special features:	abnormal karyotype<br>derived in serum free medium<br>derived on human feeder cells<br>mechanical ICM isolation
published in a peer-reviewed journal:	yes
characterization data reported in a peer-reviewed journal:	yes
reference:	Lin, G. et al. Cell Stem Cell 2009 5, 461-465 (2009)

hESC line:	**chHES8**
special features:	derived in serum free medium<br>derived on human feeder cells<br>mechanical ICM isolation
published in a peer-reviewed journal:	yes
characterization data reported in a peer-reviewed journal:	yes
reference:	Zhou, J. et al. Stem Cells Dev 17, 737-749 (2008)

hESC line:	**chHES80**
special features:	derived in serum free medium<br>derived on human feeder cells<br>mechanical ICM isolation

published in a peer-reviewed journal:	yes
characterization data reported in a peer-reviewed journal:	yes
reference:	Lin, G. et al. Cell Stem Cell 2009 5, 461-465 (2009)

hESC line:	**chHES81**
special features:	derived in serum free medium<br>derived on human feeder cells<br>mechanical ICM isolation
published in a peer-reviewed journal:	yes
characterization data reported in a peer-reviewed journal:	yes
reference:	Lin, G. et al. Cell Stem Cell 2009 5, 461-465 (2009)

hESC line:	**chHES82**
special features:	abnormal karyotype<br>derived in serum free medium<br>derived on human feeder cells<br>mechanical ICM isolation
published in a peer-reviewed journal:	yes
characterization data reported in a peer-reviewed journal:	yes
reference:	Lin, G. et al. Cell Stem Cell 2009 5, 461-465 (2009)

hESC line:	**chHES83**
special features:	derived in serum free medium<br>derived on human feeder cells<br>mechanical ICM isolation
published in a peer-reviewed journal:	yes
characterization data reported in a peer-reviewed journal:	yes
reference:	Lin, G. et al. Cell Stem Cell 2009 5, 461-465 (2009)

hESC line:	**chHES84**
special features:	derived in serum free medium<br>derived on human feeder cells<br>mechanical ICM isolation

published in a peer-reviewed journal:	yes
characterization data reported in a peer-reviewed journal:	yes
reference:	Lin, G. et al. Cell Stem Cell 2009 5, 461-465 (2009)

---

hESC line:	**chHES85**
special features:	derived in serum free medium<br>derived on human feeder cells<br>mechanical ICM isolation
published in a peer-reviewed journal:	yes
characterization data reported in a peer-reviewed journal:	yes
reference:	Lin, G. et al. Cell Stem Cell 2009 5, 461-465 (2009)

---

hESC line:	**chHES86**
special features:	derived in serum free medium<br>derived on human feeder cells<br>mechanical ICM isolation
published in a peer-reviewed journal:	yes
characterization data reported in a peer-reviewed journal:	yes
reference:	Lin, G. et al. Cell Stem Cell 2009 5, 461-465 (2009)

---

hESC line:	**chHES87**
special features:	derived in serum free medium<br>derived on human feeder cells<br>mechanical ICM isolation
published in a peer-reviewed journal:	yes
characterization data reported in a peer-reviewed journal:	yes
reference:	Lin, G. et al. Cell Stem Cell 2009 5, 461-465 (2009)

---

hESC line:	**chHES88**
special features:	derived in serum free medium<br>derived on human feeder cells<br>mechanical ICM isolation

published in a peer-reviewed journal:	yes
characterization data reported in a peer-reviewed journal:	yes
reference:	Lin, G. et al. Cell Stem Cell 2009 5, 461-465 (2009)

hESC line:	**chHES89**
special features:	derived in serum free medium<br>derived on human feeder cells<br>mechanical ICM isolation
published in a peer-reviewed journal:	yes
characterization data reported in a peer-reviewed journal:	yes
reference:	Lin, G. et al. Cell Stem Cell 2009 5, 461-465 (2009)

hESC line:	**chHES9**
special features:	abnormal karyotype<br>derived in serum free medium<br>derived on human feeder cells<br>mechanical ICM isolation
published in a peer-reviewed journal:	yes
characterization data reported in a peer-reviewed journal:	yes
reference:	Lin, G. et al. Cell Stem Cell 2009 5, 461-465 (2009)

hESC line:	**chHES90**
special features:	derived in serum free medium<br>derived on human feeder cells<br>mechanical ICM isolation
published in a peer-reviewed journal:	yes
characterization data reported in a peer-reviewed journal:	yes
reference:	Lin, G. et al. Cell Stem Cell 2009 5, 461-465 (2009)

hESC line:	**chHES91**
special features:	abnormal karyotype<br>derived in serum free medium<br>derived on human feeder cells<br>mechanical ICM isolation

published in a peer-reviewed journal:	yes
characterization data reported in a peer-reviewed journal:	yes
reference:	Lin, G. et al. Cell Stem Cell 2009 5, 461-465 (2009)

hESC line:	**chHES92**
special features:	derived in serum free medium<br>derived on human feeder cells<br>mechanical ICM isolation
published in a peer-reviewed journal:	yes
characterization data reported in a peer-reviewed journal:	yes
reference:	Lin, G. et al. Cell Stem Cell 2009 5, 461-465 (2009)

hESC line:	**chHES94**
special features:	abnormal karyotype<br>derived in serum free medium<br>derived on human feeder cells<br>mechanical ICM isolation
published in a peer-reviewed journal:	yes
characterization data reported in a peer-reviewed journal:	yes
reference:	Lin, G. et al. Cell Stem Cell 2009 5, 461-465 (2009)

hESC line:	**chHES95**
special features:	derived in serum free medium<br>derived on human feeder cells<br>mechanical ICM isolation
published in a peer-reviewed journal:	yes
characterization data reported in a peer-reviewed journal:	yes
reference:	Lin, G. et al. Cell Stem Cell 2009 5, 461-465 (2009)

hESC line:	**chHES96**
special features:	abnormal karyotype<br>derived in serum free medium<br>derived on human feeder cells<br>mechanical ICM isolation

published in a peer-reviewed journal: yes

characterization data reported in a peer-reviewed journal: yes

reference: Lin, G. et al. Cell Stem Cell 2009 5, 461-465 (2009)

---

hESC line: **chHES97**

special features: derived in serum free medium
derived on human feeder cells
mechanical ICM isolation

published in a peer-reviewed journal: yes

characterization data reported in a peer-reviewed journal: yes

reference: Lin, G. et al. Cell Stem Cell 2009 5, 461-465 (2009)

---

hESC line: **chHES98**

special features: derived in serum free medium
derived on human feeder cells
mechanical ICM isolation

published in a peer-reviewed journal: yes

characterization data reported in a peer-reviewed journal: yes

reference: Lin, G. et al. Cell Stem Cell 2009 5, 461-465 (2009)

---

hESC line: **chHES99**

special features: derived in serum free medium
derived on human feeder cells
mechanical ICM isolation

published in a peer-reviewed journal: yes

characterization data reported in a peer-reviewed journal: yes

reference: Lin, G. et al. Cell Stem Cell 2009 5, 461-465 (2009)

Provider: China Medical University, Shenyang, China

hESC line: **n.n.**

special features: derived in serum free medium

published in a peer-reviewed journal: yes

characterization data reported in a peer-reviewed journal: yes

reference: Tan, J. C. et al. Neuroreport 19, 1451-1455 (2008)

**Provider: Drum Tower Hospital affiliates to the Nanjing University School of Medicine, Nanjin**	
hESC line:	**NJGLLhES1**
special features:	
published in a peer-reviewed journal:	no
characterization data reported in a peer-reviewed journal:	no
reference:	Sun, H. X. et al. Zhonghua nan ke xue14, 1083-1089 (2008) (Article in Chinnese)

**Provider: Huazhong University of Science and Technology, Wuhan, China**	
hESC line:	**TJMU1 (formerly hES-8)**
special features:	
published in a peer-reviewed journal:	yes
characterization data reported in a peer-reviewed journal:	yes
reference:	Chen, H. et al. Hum Reprod 20, 2201-2206 (2005)<br>Jiao, S. et al. J Huazhong Univ Sci Technolog Med Sci 29, 563-566 (2009)

hESC line:	**TJMU2 (formerly hES-18)**
special features:	
published in a peer-reviewed journal:	yes
characterization data reported in a peer-reviewed journal:	yes
reference:	Chen, H. et al. Hum Reprod 20, 2201-2206 (2005)<br>Jiao, S. et al. J Huazhong Univ Sci Technolog Med Sci 29, 563-566 (2009)

**Provider: Peking University Stem Cell Research Center, Beijing, China**	
hESC line:	**B4**
special features:	derived from research embryo
published in a peer-reviewed journal:	yes
characterization data reported in a peer-reviewed journal:	yes
reference:	Peng, H.-M. & Chen, G.-A. Hum Reprod 21, 217-222 (2006)

hESC line:	**B7**
special features:	derived from research embryo

published in a peer-reviewed journal:	yes
characterization data reported in a peer-reviewed journal:	yes
reference:	Peng, H.-M. & Chen, G.-A. Hum Reprod 21, 217-222 (2006)

hESC line:	**n.n**
special features:	
published in a peer-reviewed journal:	no
characterization data reported in a peer-reviewed journal:	no
reference:	Peng, H. M. & Chen G. A. Zhonghua Fu Chan Ke Za Zhi 40, 521-524 (2005)(Article in Chinese)

hESC line:	**n.n.**
special features:	
published in a peer-reviewed journal:	no
characterization data reported in a peer-reviewed journal:	no
reference:	Peng, H. M. & Chen G. A. Zhonghua Fu Chan Ke Za Zhi 40, 521-524 (2005)(Article in Chinese)

hESC line:	**PKU1**
special features:	derived from research embryo<br>derived in serum free medium
published in a peer-reviewed journal:	yes
characterization data reported in a peer-reviewed journal:	yes
reference:	Peng, H.-M. & Chen, G.-A. Hum Reprod 21, 217-222 (2006)

hESC line:	**PKU2**
special features:	derived in serum free medium
published in a peer-reviewed journal:	yes
characterization data reported in a peer-reviewed journal:	yes
reference:	Peng, H.-M. & Chen, G.-A. Hum Reprod 21, 217-222 (2006)

Provider: Shanghai JiaoTong University School of Medicine, Shanghai, China

hESC line:	**SHhes1**
special features:	derived by whole embryo culture<br>derived in serum free medium

published in a peer-reviewed journal: yes
characterization data reported in a peer-reviewed journal: yes
reference: Sun, B.W. et al. Hum Mol Genet 15, 65-75 (2006)

Provider: Shanghai Second Medical University, Shanghai, China

hESC line: **SH1**
special features:
published in a peer-reviewed journal: yes
characterization data reported in a peer-reviewed journal: no
reference: Wang, Q. et al. Stem Cells 23 1221-1227 (2005)

---

hESC line: **SH2**
special features:
published in a peer-reviewed journal: yes
characterization data reported in a peer-reviewed journal: no
reference: Wang, Q. et al. Stem Cells 23 1221-1227 (2005)

---

hESC line: **SH28**
special features: derived in serum free medium
mechanical ICM isolation
published in a peer-reviewed journal: yes
characterization data reported in a peer-reviewed journal: yes
reference: Fang, Z. F. et al. Cell Res 15, 394-400 (2005)

---

hESC line: **SH35**
special features: derived in serum free medium
mechanical ICM isolation
published in a peer-reviewed journal: yes
characterization data reported in a peer-reviewed journal: yes
reference: Fang, Z. F. et al. Cell Res 15, 394-400 (2005)

---

hESC line: **SH38**
special features: derived in serum free medium
mechanical ICM isolation

published in a peer-reviewed journal:	yes
characterization data reported in a peer-reviewed journal:	yes
reference:	Fang, Z. F. et al. Cell Res 15, 394-400 (2005)

hESC line:	**SH39**
special features:	derived in serum free medium<br>mechanical ICM isolation
published in a peer-reviewed journal:	yes
characterization data reported in a peer-reviewed journal:	yes
reference:	Fang, Z. F. et al. Cell Res 15, 394-400 (2005)

hESC line:	**SH4**
special features:	derived in serum free medium<br>mechanical ICM isolation
published in a peer-reviewed journal:	yes
characterization data reported in a peer-reviewed journal:	yes
reference:	Fang, Z. F. et al. Cell Res 15, 394-400 (2005)

hESC line:	**SH42**
special features:	derived in serum free medium<br>mechanical ICM isolation
published in a peer-reviewed journal:	yes
characterization data reported in a peer-reviewed journal:	yes
reference:	Fang, Z. F. et al. Cell Res 15, 394-400 (2005)

hESC line:	**SH7**
special features:	derived in serum free medium<br>derived on human feeder cells<br>mechanical ICM isolation
published in a peer-reviewed journal:	yes
characterization data reported in a peer-reviewed journal:	yes
reference:	Wang, Q. et al. Stem Cells 23 1221-1227 (2005)

Provider: State Key Laboratory of Reproductive Biology, Beijing, China

hESC line:	**hPES-1**
special features:	
published in a peer-reviewed journal:	no
characterization data reported in a peer-reviewed journal:	no
reference:	European Union hESC registry (http://www.hescreg.eu/)

hESC line:	**hPES-2**
special features:	
published in a peer-reviewed journal:	no
characterization data reported in a peer-reviewed journal:	no
reference:	European Union hESC registry (http://www.hescreg.eu/)

Provider: Sun Yat-sen University, Guangzhou, China

hESC line:	**alpha-EC-C**
special features:	genetic disorder: alpha-Thalassaemia (carrier)
published in a peer-reviewed journal:	yes
characterization data reported in a peer-reviewed journal:	no
reference:	Li, T. et al. Hum Reprod 23, 358-364 (2008)

hESC line:	**CHE1**
special features:	
published in a peer-reviewed journal:	no
characterization data reported in a peer-reviewed journal:	no
reference:	He, Z. et al. Zhonghua Yi Xue Za Zhi 82, 1314-1318 (2002) (article in Chinese)

hESC line:	**CHE2**
special features:	
published in a peer-reviewed journal:	no
characterization data reported in a peer-reviewed journal:	no
reference:	He, Z. et al. Zhonghua Yi Xue Za Zhi 82, 1314-1318 (2002) (article in Chinese)

hESC line:	**CHE3**
special features:	

published in a peer-reviewed journal:	no
characterization data reported in a peer-reviewed journal:	no
reference:	He, Z. et al. Zhonghua Yi Xue Za Zhi 82, 1314-1318 (2002) (article in Chinese)

hESC line:	**cHES-1**
special features:	derived from research embryo
published in a peer-reviewed journal:	yes
characterization data reported in a peer-reviewed journal:	yes
reference:	Li, T. et al. Chin Med J (Engl) 118, 116-122 (2005)

hESC line:	**SYSU-1**
special features:	
published in a peer-reviewed journal:	yes
characterization data reported in a peer-reviewed journal:	yes
reference:	Huang, G. et al. Chin Med J (Engl) 120, 589-594 (2007)

hESC line:	**SYSU-2**
special features:	
published in a peer-reviewed journal:	yes
characterization data reported in a peer-reviewed journal:	yes
reference:	Huang, G. et al. Chin Med J (Engl) 120, 589-594 (2007)

Provider: The Third Affiliated Hospital of Guangzhou Medical Collge, Guangzhou, China

hESC line:	**FY-3PN**
special features:	abnormal karyotype
published in a peer-reviewed journal:	yes
characterization data reported in a peer-reviewed journal:	yes
reference:	Sun, X. et al. Hum Reprod 23, 2185-2193 (2008)

hESC line:	**FY-hES-1**
special features:	

published in a peer-reviewed journal:	yes
characterization data reported in a peer-reviewed journal:	yes
reference:	Sun, X. et al. Hum Reprod 23, 2185-2193 (2008)

---

hESC line:	**FY-hES-10**
special features:	mechanical ICM isolation
published in a peer-reviewed journal:	yes
characterization data reported in a peer-reviewed journal:	yes
reference:	Liu, W. et al. J Genet Genomics 36, 229-239 (2009)

---

hESC line:	**FY-hES-11**
special features:	mechanical ICM isolation
published in a peer-reviewed journal:	yes
characterization data reported in a peer-reviewed journal:	yes
reference:	Liu, W. et al. J Genet Genomics 36, 229-239 (2009)

---

hESC line:	**FY-hES-3**
special features:	
published in a peer-reviewed journal:	yes
characterization data reported in a peer-reviewed journal:	yes
reference:	Sun, X. et al. Hum Reprod 23, 2185-2193 (2008)

---

hESC line:	**FY-hES-4**
special features:	
published in a peer-reviewed journal:	yes
characterization data reported in a peer-reviewed journal:	yes
reference:	Sun, X. et al. Hum Reprod 23, 2185-2193 (2008)

---

hESC line:	**FY-hES-5**
special features:	abnormal karyotype

published in a peer-reviewed journal: yes

characterization data reported in a peer-reviewed journal: yes

reference: Liu, W. et al. J Genet Genomics 36, 229-239 (2009)
Sun, X. et al. Hum Reprod 23, 2185-2193 (2008)

---

hESC line: **FY-hES-7**

special features:

published in a peer-reviewed journal: yes

characterization data reported in a peer-reviewed journal: yes

reference: Sun, X. et al. Hum Reprod 23, 2185-2193 (2008)

---

hESC line: **FY-hES-9**

special features:

published in a peer-reviewed journal: yes

characterization data reported in a peer-reviewed journal: yes

reference: Liu, W. et al. J Genet Genomics 36, 229-239 (2009)

Provider: The Third Affiliated Hosptial of Guangzhou Medical Collge, Guangzhou, China

hESC line: **FY-hES-8**

special features:

published in a peer-reviewed journal: yes

characterization data reported in a peer-reviewed journal: yes

reference: Sun, X. et al. Hum Reprod 23, 2185-2193 (2008)

CZECH REPUBLIC

Provider: Mendel University Brno

hESC line: **CCTL10**

special features:

published in a peer-reviewed journal: yes

characterization data reported in a peer-reviewed journal: no

reference: Dvorak, P. et al. Stem Cells 23, 1200-1211 (2005)

---

hESC line:	**CCTL12**
special features:	
published in a peer-reviewed journal:	yes
characterization data reported in a peer-reviewed journal:	yes
reference:	Dvorak, P. et al. Stem Cells 23, 1200-1211 (2005)

hESC line:	**CCTL13**
special features:	
published in a peer-reviewed journal:	no
characterization data reported in a peer-reviewed journal:	no
reference:	European Union hESC registry (http://www.hescreg.eu/)

hESC line:	**CCTL14**
special features:	
published in a peer-reviewed journal:	yes
characterization data reported in a peer-reviewed journal:	yes
reference:	Dvorak, P. et al. Stem Cells 23, 1200-1211 (2005)

hESC line:	**CCTL6**
special features:	
published in a peer-reviewed journal:	no
characterization data reported in a peer-reviewed journal:	no
reference:	European Union hESC registry (http://www.hescreg.eu/)

hESC line:	**CCTL8**
special features:	
published in a peer-reviewed journal:	no
characterization data reported in a peer-reviewed journal:	no
reference:	European Union hESC registry (http://www.hescreg.eu/)

hESC line:	**CCTL9**
special features:	

published in a peer-reviewed journal:	yes
characterization data reported in a peer-reviewed journal:	yes
reference:	Dvorak, P. et al. Stem Cells 23, 1200-1211 (2005)

DENMARK

Provider: Ciconia Aarhus Privathospital

hESC line:	**CSL1**
special features:	
published in a peer-reviewed journal:	no
characterization data reported in a peer-reviewed journal:	no
reference:	European Union hESC registry (http://www.hescreg.eu/)

hESC line:	**CSL2**
special features:	
published in a peer-reviewed journal:	no
characterization data reported in a peer-reviewed journal:	no
reference:	European Union hESC registry (http://www.hescreg.eu/)

hESC line:	**CSL3**
special features:	
published in a peer-reviewed journal:	no
characterization data reported in a peer-reviewed journal:	no
reference:	European Union hESC registry (http://www.hescreg.eu/)

hESC line:	**CSL4**
special features:	
published in a peer-reviewed journal:	no
characterization data reported in a peer-reviewed journal:	no
reference:	European Union hESC registry (http://www.hescreg.eu/)

Provider: Laboratory for Stem Cell Research, Aalborg University

hESC line:	**CLS1**
special features:	derived in serum free medium<br>derived on human feeder cells
published in a peer-reviewed journal:	yes
characterization data reported in a peer-reviewed journal:	yes
reference:	Lysdahl, H. et al. Reprod Biomed Online 12, 119-126 (2006)

hESC line:	**CLS2**
special features:	derived in serum free medium<br>derived on human feeder cells
published in a peer-reviewed journal:	yes
characterization data reported in a peer-reviewed journal:	yes
reference:	Lysdahl, H. et al. Reprod Biomed Online 12, 119-126 (2006)

hESC line:	**CLS3**
special features:	derived in serum free medium<br>derived on human feeder cells
published in a peer-reviewed journal:	yes
characterization data reported in a peer-reviewed journal:	yes
reference:	Lysdahl, H. et al. Reprod Biomed Online 12, 119-126 (2006)

hESC line:	**CLS4**
special features:	derived in serum free medium<br>derived on human feeder cells
published in a peer-reviewed journal:	yes
characterization data reported in a peer-reviewed journal:	yes
reference:	Lysdahl, H. et al. Reprod Biomed Online 12, 119-126 (2006)

Provider: University of Copenhagen

hESC line:	**LRB005**
special features:	

published in a peer-reviewed journal:	no
characterization data reported in a peer-reviewed journal:	no
reference:	European Union hESC registry (http://www.hescreg.eu/)

hESC line:	**LRB006**
special features:	
published in a peer-reviewed journal:	no
characterization data reported in a peer-reviewed journal:	no
reference:	European Union hESC registry (http://www.hescreg.eu/)

hESC line:	**LRB007**
special features:	
published in a peer-reviewed journal:	no
characterization data reported in a peer-reviewed journal:	no
reference:	European Union hESC registry (http://www.hescreg.eu/)

hESC line:	**LRB008**
special features:	
published in a peer-reviewed journal:	no
characterization data reported in a peer-reviewed journal:	no
reference:	European Union hESC registry (http://www.hescreg.eu/)

hESC line:	**LRB009**
special features:	
published in a peer-reviewed journal:	no
characterization data reported in a peer-reviewed journal:	no
reference:	European Union hESC registry (http://www.hescreg.eu/)

hESC line:	**LRB01**
special features:	derived in serum free medium

published in a peer-reviewed journal:	yes
characterization data reported in a peer-reviewed journal:	yes
reference:	Laursen, S. B. et al. Reprod Biomed Online 15, 89-98 (2007)

hESC line:	**LRB010**
special features:	
published in a peer-reviewed journal:	no
characterization data reported in a peer-reviewed journal:	no
reference:	European Union hESC registry (http://www.hescreg.eu/)

hESC line:	**LRB011**
special features:	
published in a peer-reviewed journal:	no
characterization data reported in a peer-reviewed journal:	no
reference:	European Union hESC registry (http://www.hescreg.eu/)

hESC line:	**LRB013**
special features:	
published in a peer-reviewed journal:	no
characterization data reported in a peer-reviewed journal:	no
reference:	European Union hESC registry (http://www.hescreg.eu/)

hESC line:	**LRB014**
special features:	
published in a peer-reviewed journal:	no
characterization data reported in a peer-reviewed journal:	no
reference:	European Union hESC registry (http://www.hescreg.eu/)

hESC line:	**LRB016**
special features:	

published in a peer-reviewed journal:	no
characterization data reported in a peer-reviewed journal:	no
reference:	European Union hESC registry (http://www.hescreg.eu/)

hESC line:	**LRB017**
special features:	
published in a peer-reviewed journal:	no
characterization data reported in a peer-reviewed journal:	no
reference:	European Union hESC registry (http://www.hescreg.eu/)

hESC line:	**LRB018**
special features:	
published in a peer-reviewed journal:	no
characterization data reported in a peer-reviewed journal:	no
reference:	European Union hESC registry (http://www.hescreg.eu/)

hESC line:	**LRB02**
special features:	derived in serum free medium
published in a peer-reviewed journal:	yes
characterization data reported in a peer-reviewed journal:	yes
reference:	Laursen, S. B. et al. Reprod Biomed Online 15, 89-98 (2007)

hESC line:	**LRB03**
special features:	derived in serum free medium
published in a peer-reviewed journal:	yes
characterization data reported in a peer-reviewed journal:	yes
reference:	Laursen, S. B. et al. Reprod Biomed Online 15, 89-98 (2007)

hESC line:	**LRB04**
special features:	derived in serum free medium

published in a peer-reviewed journal:	yes
characterization data reported in a peer-reviewed journal:	yes
reference:	Laursen, S. B. et al. Reprod Biomed Online 15, 89-98 (2007)

Provider: University of Southern Denmark

hESC line:	**KMEB1**
special features:	derived by whole embryo culture
published in a peer-reviewed journal:	yes
characterization data reported in a peer-reviewed journal:	yes
reference:	Prokhorova, T. A. et al. Stem Cells Dev, published online ahead of print April 7th, 2008

hESC line:	**KMEB2**
special features:	derived by whole embryo culture
published in a peer-reviewed journal:	yes
characterization data reported in a peer-reviewed journal:	yes
reference:	Prokhorova, T. A. et al. Stem Cells Dev, published online ahead of print April 7th, 2008

hESC line:	**KMEB3**
special features:	derived by whole embryo culture
published in a peer-reviewed journal:	yes
characterization data reported in a peer-reviewed journal:	yes
reference:	Prokhorova, T. A. et al. Stem Cells Dev, published online ahead of print April 7th, 2008

hESC line:	**KMEB4**
special features:	derived by whole embryo culture
published in a peer-reviewed journal:	yes
characterization data reported in a peer-reviewed journal:	yes
reference:	Prokhorova, T. A. et al. Stem Cells Dev, published online ahead of print April 7th, 2008

hESC line:	**KMEB5**
special features:	derived by whole embryo culture

published in a peer-reviewed journal:	yes
characterization data reported in a peer-reviewed journal:	yes
reference:	Prokhorova, T. A. et al. Stem Cells Dev, published online ahead of print April 7th, 2008

hESC line:	**Odense-3 (hESC-OD3)**
special features:	
published in a peer-reviewed journal:	yes
characterization data reported in a peer-reviewed journal:	yes
reference:	Prokhorova, T. A. et al. Stem Cells Dev, published online ahead of print April 7th, 2008

hESC line:	**Odense-4**
special features:	
published in a peer-reviewed journal:	no
characterization data reported in a peer-reviewed journal:	no
reference:	European Union hESC registry (http://www.hescreg.eu/)

FINLAND

Provider: University of Helsinki

hESC line:	**FES21**
special features:	
published in a peer-reviewed journal:	yes
characterization data reported in a peer-reviewed journal:	yes
reference:	Skottman, H. et al. Stem Cells 23, 1343-1356 (2005)<br>Mikkola, M. et al. BMC Dev Biol 6, 40 (2006)

hESC line:	**FES22**
special features:	
published in a peer-reviewed journal:	yes
characterization data reported in a peer-reviewed journal:	yes
reference:	Skottman, H. et al. Stem Cells 23, 1343-1356 (2005)<br>Mikkola, M. et al. BMC Dev Biol 6, 40 (2006)

hESC line:	**FES29**
special features:	derived on human feeder cells
published in a peer-reviewed journal:	yes
characterization data reported in a peer-reviewed journal:	yes
reference:	Skottman, H. et al. Stem Cells 23, 1343-1356 (2005)<br>Mikkola, M. et al. BMC Dev Biol 6, 40 (2006)

hESC line:	**FES30**
special features:	derived on human feeder cells
published in a peer-reviewed journal:	yes
characterization data reported in a peer-reviewed journal:	yes
reference:	Skottman, H. et al. Stem Cells 23, 1343-1356 (2005)<br>Mikkola, M. et al. BMC Dev Biol 6, 40 (2006)

hESC line:	**FES61**
special features:	derived in serum free medium<br>derived on human feeder cells<br>mechanical ICM isolation
published in a peer-reviewed journal:	yes
characterization data reported in a peer-reviewed journal:	yes
reference:	Mikkola, M. et al. BMC Dev Biol 6, 40 (2006)

hESC line:	**FES75**
special features:	
published in a peer-reviewed journal:	no
characterization data reported in a peer-reviewed journal:	no
reference:	European Union hESC registry (http://www.hescreg.eu/)

Provider: University of Tampere

hESC line:	**Regea 06/040**
special features:	
published in a peer-reviewed journal:	no
characterization data reported in a peer-reviewed journal:	no
reference:	European Union hESC registry (http://www.hescreg.eu/)

hESC line:	**Regea 07/027**
special features:	
published in a peer-reviewed journal:	no
characterization data reported in a peer-reviewed journal:	no
reference:	European Union hESC registry (http://www.hescreg.eu/)

hESC line:	**Regea 07/046**
special features:	
published in a peer-reviewed journal:	no
characterization data reported in a peer-reviewed journal:	no
reference:	European Union hESC registry (http://www.hescreg.eu/)

hESC line:	**Regea06/015**
special features:	
published in a peer-reviewed journal:	no
characterization data reported in a peer-reviewed journal:	no
reference:	European Union hESC registry (http://www.hescreg.eu/)

FRANCE

Provider: INSERM, Institut de génétique et de Biologie Moléculaire et Cellulaire, Illkirch, Fran

hESC line:	**STR-233-FRAXA**
special features:	genetic disorder: Fragile X syndrome (FX)
published in a peer-reviewed journal:	no
characterization data reported in a peer-reviewed journal:	no
reference:	European Union hESC registry (http://www.hescreg.eu/)

hESC line:	**STR-I-155-HD**
special features:	genetic disorder: Huntington's desease (HD)
published in a peer-reviewed journal:	no
characterization data reported in a peer-reviewed journal:	no
reference:	European Union hESC registry (http://www.hescreg.eu/)

hESC line:	**STR-I-171-GLA**
special features:	genetic disorder: Fabry Syndrome
published in a peer-reviewed journal:	no
characterization data reported in a peer-reviewed journal:	no
reference:	European Union hESC registry (http://www.hescreg.eu/)

---

hESC line:	**STR-I-189-FRAXA**
special features:	genetic disorder: Fragile X syndrome (FX)
published in a peer-reviewed journal:	no
characterization data reported in a peer-reviewed journal:	no
reference:	European Union hESC registry (http://www.hescreg.eu/)

---

hESC line:	**STR-I-203-CFTR**
special features:	genetic disorder: Cystic fibrosis (CF)
published in a peer-reviewed journal:	no
characterization data reported in a peer-reviewed journal:	no
reference:	European Union hESC registry (http://www.hescreg.eu/)

---

hESC line:	**STR-I-209-MEN2a**
special features:	genetic disorder: Multiple endocrine neoplasia, Type Iia
published in a peer-reviewed journal:	no
characterization data reported in a peer-reviewed journal:	no
reference:	European Union hESC registry (http://www.hescreg.eu/)

---

hESC line:	**STR-I-211-MEN2a**
special features:	genetic disorder: Multiple endocrine neoplasia, Type Iia
published in a peer-reviewed journal:	no
characterization data reported in a peer-reviewed journal:	no
reference:	European Union hESC registry (http://www.hescreg.eu/)

---

hESC line:	**STR-I-251-CFTR**
special features:	genetic disorder: Cystic fibrosis (CF)

published in a peer-reviewed journal:	no
characterization data reported in a peer-reviewed journal:	no
reference:	European Union hESC registry (http://www.hescreg.eu/)

hESC line:	**STR-I-263-Sca2**
special features:	genetic disorder: Spinocerebellar Ataxia Type 2 (SCA2)
published in a peer-reviewed journal:	no
characterization data reported in a peer-reviewed journal:	no
reference:	European Union hESC registry (http://www.hescreg.eu/)

hESC line:	**STR-I-271-MTMX**
special features:	genetic disorder: Myotubular myopathy (MTM), X linked
published in a peer-reviewed journal:	no
characterization data reported in a peer-reviewed journal:	no
reference:	European Union hESC registry (http://www.hescreg.eu/)

hESC line:	**STR-I-275-MTMX**
special features:	genetic disorder: Myotubular myopathy (MTM), X linked
published in a peer-reviewed journal:	no
characterization data reported in a peer-reviewed journal:	no
reference:	European Union hESC registry (http://www.hescreg.eu/)

hESC line:	**STR-I-301-MFS**
special features:	genetic disorder: Marfan syndrome (MFS)
published in a peer-reviewed journal:	no
characterization data reported in a peer-reviewed journal:	no
reference:	European Union hESC registry (http://www.hescreg.eu/)

hESC line:	**STR-I-305-APC**
special features:	genetic disorder: Familial adenomatous polyposis (FAP)

published in a peer-reviewed journal:	no
characterization data reported in a peer-reviewed journal:	no
reference:	European Union hESC registry (http://www.hescreg.eu/)

hESC line:	**STR-I-315-CMT1a**
special features:	genetic disorder: Charcot-Marie-Tooth-Disease 1A (CMT1A)
published in a peer-reviewed journal:	no
characterization data reported in a peer-reviewed journal:	no
reference:	European Union hESC registry (http://www.hescreg.eu/)

hESC line:	**STR-I-347-FRAXA**
special features:	genetic disorder: Fragile X syndrome (FX)
published in a peer-reviewed journal:	no
characterization data reported in a peer-reviewed journal:	no
reference:	European Union hESC registry (http://www.hescreg.eu/)

hESC line:	**STR-I-355-APC**
special features:	genetic disorder: Familial adenomatous polyposis (FAP)
published in a peer-reviewed journal:	no
characterization data reported in a peer-reviewed journal:	no
reference:	European Union hESC registry (http://www.hescreg.eu/)

hESC line:	**STR-I-359-APC**
special features:	genetic disorder: Familial adenomatous polyposis (FAP)
published in a peer-reviewed journal:	no
characterization data reported in a peer-reviewed journal:	no
reference:	European Union hESC registry (http://www.hescreg.eu/)

Provider: INSERM, Stem Cell and Brain Research Institute, Bron, France

hESC line:	**OSCAR**
special features:	

published in a peer-reviewed journal: no

characterization data reported in a peer-reviewed journal: no

reference: European Union hESC registry (http://www.hescreg.eu/)

Provider: Université Paris-Sud 11, France

hESC line: **CL01**

special features:

published in a peer-reviewed journal: no

characterization data reported in a peer-reviewed journal: no

reference: European Union hESC registry (http://www.hescreg.eu/)

Provider: University Hospital of Montpellier, France

hESC line: **HD90/FE07-142-L1**

special features: genetic disorder: Huntington's desease (HD)

published in a peer-reviewed journal: no

characterization data reported in a peer-reviewed journal: no

reference: European Union hESC registry (http://www.hescreg.eu/)

INDIA

Provider: Jawaharlal Nehru Centre for Advanced Scientific Research

hESC line: **BJNhem19**

special features:

published in a peer-reviewed journal: yes

characterization data reported in a peer-reviewed journal: yes

reference: Inamdar, M. S. et al., Stem Cells Dev 18, 423-433 (2009)

---

hESC line: **BJNhem20**

special features:

published in a peer-reviewed journal: yes

characterization data reported in a peer-reviewed journal: yes

reference: Inamdar, M. S. et al., Stem Cells Dev 18, 423-433 (2009)

Provider: National Centre for Biological Sciences/Tata Institute of Fundamental Research (In

hESC line:	**FCNCBS1**
special features:	
published in a peer-reviewed journal:	no
characterization data reported in a peer-reviewed journal:	no
reference:	NIH Human Embryonic Stem Cell Registry (http://grants.nih.gov/stem_cells/registry/current.htm)

hESC line:	**FCNCBS2**
special features:	
published in a peer-reviewed journal:	no
characterization data reported in a peer-reviewed journal:	no
reference:	NIH Human Embryonic Stem Cell Registry (http://grants.nih.gov/stem_cells/registry/current.htm)

hESC line:	**FCNCBS3**
special features:	
published in a peer-reviewed journal:	no
characterization data reported in a peer-reviewed journal:	no
reference:	NIH Human Embryonic Stem Cell Registry (http://grants.nih.gov/stem_cells/registry/current.htm)

Provider: National Institute for research in Reproductive health, Mumbai, India

hESC line:	**KIND1**
special features:	derived by whole embryo culture<br>derived in serum free medium<br>derived on human feeder cells
published in a peer-reviewed journal:	yes
characterization data reported in a peer-reviewed journal:	yes
reference:	Kumar, N. et al. Stem Cells Dev 18, 435-445 (2009)

hESC line:	**KIND2**
special features:	derived by whole embryo culture<br>derived in serum free medium<br>derived on human feeder cells

published in a peer-reviewed journal:	yes
characterization data reported in a peer-reviewed journal:	yes
reference:	Kumar, N. et al. Stem Cells Dev 18, 435-445 (2009)

Provider: Reliance Life Sciences

hESC line:	**ReliCellhES1**
special features:	derived by whole embryo culture
published in a peer-reviewed journal:	yes
characterization data reported in a peer-reviewed journal:	yes
reference:	Mandal, A. et al. Differentiation 74, 81-90 (2006)

hESC line:	**RLS ES 05**
special features:	
published in a peer-reviewed journal:	no
characterization data reported in a peer-reviewed journal:	no
reference:	NIH Human Embryonic Stem Cell Registry (http://grants.nih.gov/stem_cells/registry/current.htm)

hESC line:	**RLS ES 07**
special features:	
published in a peer-reviewed journal:	no
characterization data reported in a peer-reviewed journal:	no
reference:	NIH Human Embryonic Stem Cell Registry (http://grants.nih.gov/stem_cells/registry/current.htm)

hESC line:	**RLS ES 10**
special features:	
published in a peer-reviewed journal:	no
characterization data reported in a peer-reviewed journal:	no
reference:	NIH Human Embryonic Stem Cell Registry (http://grants.nih.gov/stem_cells/registry/current.htm)

hESC line:	**RLS ES 13**
special features:	

published in a peer-reviewed journal:	no
characterization data reported in a peer-reviewed journal:	no
reference:	NIH Human Embryonic Stem Cell Registry (http://grants.nih.gov/stem_cells/registry/current.htm)

hESC line:	**RLS ES 15**
special features:	
published in a peer-reviewed journal:	no
characterization data reported in a peer-reviewed journal:	no
reference:	NIH Human Embryonic Stem Cell Registry (http://grants.nih.gov/stem_cells/registry/current.htm)

hESC line:	**RLS ES 20**
special features:	
published in a peer-reviewed journal:	no
characterization data reported in a peer-reviewed journal:	no
reference:	NIH Human Embryonic Stem Cell Registry (http://grants.nih.gov/stem_cells/registry/current.htm)

hESC line:	**RLS ES 21**
special features:	
published in a peer-reviewed journal:	no
characterization data reported in a peer-reviewed journal:	no
reference:	NIH Human Embryonic Stem Cell Registry (http://grants.nih.gov/stem_cells/registry/current.htm)

IRAN

Provider: Royan Institute, Teheran

hESC line:	**Royan H1**
special features:	derived by whole embryo culture
published in a peer-reviewed journal:	yes
characterization data reported in a peer-reviewed journal:	yes
reference:	Baharvand, H. et al. Differentiation 72, 224-229 (2004)

hESC line:	**Royan H2**
special features:	

published in a peer-reviewed journal:	yes
characterization data reported in a peer-reviewed journal:	yes
reference:	Baharvand, H. et al. Dev Growth Differ 48, 117-128 (2006)

hESC line:	**Royan H3**
special features:	
published in a peer-reviewed journal:	yes
characterization data reported in a peer-reviewed journal:	yes
reference:	Baharvand, H. et al. Dev Growth Differ 48, 117-128 (2006)

hESC line:	**Royan H4**
special features:	
published in a peer-reviewed journal:	yes
characterization data reported in a peer-reviewed journal:	yes
reference:	Baharvand, H. et al. Dev Growth Differ 48, 117-128 (2006)

hESC line:	**Royan H5**
special features:	
published in a peer-reviewed journal:	yes
characterization data reported in a peer-reviewed journal:	yes
reference:	Baharvand, H. et al. Dev Growth Differ 48, 117-128 (2006)

hESC line:	**Royan H6**
special features:	
published in a peer-reviewed journal:	yes
characterization data reported in a peer-reviewed journal:	yes
reference:	Baharvand, H. et al. Dev Growth Differ 48, 117-128 (2006)

ISRAEL

Provider: Hadassah University Medical Center

hESC line:	**HAD 1**
special features:	derived in serum free medium<br>derived on human feeder cells<br>genetic disorder: Dystrophya myotonica type 1 (DM1)<br>laser-assisted ICM isolation
published in a peer-reviewed journal:	yes
characterization data reported in a peer-reviewed journal:	yes
reference:	Turetsky, T. et al. Hum Reprod 23, 46-53 (2008)

hESC line:	**HAD 2**
special features:	derived in serum free medium<br>derived on human feeder cells<br>genetic disorder: Cystic fibrosis (CF)<br>laser-assisted ICM isolation
published in a peer-reviewed journal:	yes
characterization data reported in a peer-reviewed journal:	yes
reference:	Turetsky, T. et al. Hum Reprod 23, 46-53 (2008)

hESC line:	**HAD 3**
special features:	derived in serum free medium<br>derived on human feeder cells<br>genetic disorder: Hemophilia A<br>laser-assisted ICM isolation
published in a peer-reviewed journal:	yes
characterization data reported in a peer-reviewed journal:	yes
reference:	Turetsky, T. et al. Hum Reprod 23, 46-53 (2008)

hESC line:	**HAD 4**
special features:	
published in a peer-reviewed journal:	no
characterization data reported in a peer-reviewed journal:	no
reference:	European Union hESC registry (http://www.hescreg.eu/)

hESC line:	**HAD 5**
special features:	derived in serum free medium<br>derived on human feeder cells<br>genetic disorder: Fragile X syndrome (FX)<br>laser-assisted ICM isolation
published in a peer-reviewed journal:	yes
characterization data reported in a peer-reviewed journal:	yes
reference:	Turetsky, T. et al. Hum Reprod 23, 46-53 (2008)

hESC line:	**HAD 6**
special features:	
published in a peer-reviewed journal:	no
characterization data reported in a peer-reviewed journal:	no
reference:	European Union hESC registry (http://www.hescreg.eu/)

Provider: Hebrew University, Jerusalem, Israel

hESC line:	**HEFX**
special features:	genetic disorder: Fragile X syndrome (FX)
published in a peer-reviewed journal:	yes
characterization data reported in a peer-reviewed journal:	yes
reference:	Eiges, R. et al. Cell Stem Cell 1, 568-577 (2007)

Provider: Technion-Israel Institute of Technology, Rambam Medical Center, Haifa

hESC line:	**H2B**
special features:	
published in a peer-reviewed journal:	yes
characterization data reported in a peer-reviewed journal:	no
reference:	Bauwens, C. L. et al. Stem Cells 26, 2300-2310 (2008)

hESC line:	**I3**
special features:	

published in a peer-reviewed journal:	yes
characterization data reported in a peer-reviewed journal:	yes
reference:	Amit, M. & J. Itskovitz-Eldor. J Anat 200, 225-232 (2002)<br>NIH Human Embryonic Stem Cell Registry (http://grants.nih.gov/stem_cells/registry/current.htm)

hESC line:	**I4**
special features:	
published in a peer-reviewed journal:	yes
characterization data reported in a peer-reviewed journal:	yes
reference:	Amit, M. & J. Itskovitz-Eldor. J Anat 200, 225-232 (2002)<br>NIH Human Embryonic Stem Cell Registry (http://grants.nih.gov/stem_cells/registry/current.htm)

hESC line:	**I6**
special features:	
published in a peer-reviewed journal:	yes
characterization data reported in a peer-reviewed journal:	yes
reference:	Amit, M. & J. Itskovitz-Eldor. J Anat 200, 225-232 (2002)<br>NIH Human Embryonic Stem Cell Registry (http://grants.nih.gov/stem_cells/registry/current.htm)

hESC line:	**I8**
special features:	
published in a peer-reviewed journal:	yes
characterization data reported in a peer-reviewed journal:	no
reference:	Amit, M. et al. Stem Cells 23, 761-771 (2005)

hESC line:	**I9**
special features:	derived by whole embryo culture
published in a peer-reviewed journal:	yes
characterization data reported in a peer-reviewed journal:	yes
reference:	Suss-Toby, E. et al. Hum Reprod 19, 670-675 (2004)

hESC line:	**J3**
special features:	

published in a peer-reviewed journal:	yes
characterization data reported in a peer-reviewed journal:	no
reference:	NIH Human Embryonic Stem Cell Registry (http://grants.nih.gov/stem_cells/registry/current.htm)

JAPAN

Provider: Department of Development and Differentiation, Institute for Frontier Medical Scien

hESC line:	**KhES-1**
special features:	derived in serum free medium
published in a peer-reviewed journal:	yes
characterization data reported in a peer-reviewed journal:	yes
reference:	Suemori, H. et al. Biochem Biophys Res Commun 345, 926-932 (2006)

hESC line:	**KhES-1 (subline 1)**
special features:	clonally derived derivative
published in a peer-reviewed journal:	
characterization data reported in a peer-reviewed journal:	derivative
reference:	Suzuki, K. et al. Proc Natl Acad Sci U S A 105, 13781-13786 (2008)

hESC line:	**KhES-2**
special features:	derived in serum free medium
published in a peer-reviewed journal:	yes
characterization data reported in a peer-reviewed journal:	yes
reference:	Suemori, H. et al. Biochem Biophys Res Commun 345, 926-932 (2006)

hESC line:	**KhES-3**
special features:	derived in serum free medium
published in a peer-reviewed journal:	yes
characterization data reported in a peer-reviewed journal:	yes
reference:	Suemori, H. et al. Biochem Biophys Res Commun 345, 926-932 (2006)

KOREA

Provider: Asan Medical Center - University of Ulsan, Seoul, Korea

hESC line: **AMC-hES1**

special features: derived in serum free medium

published in a peer-reviewed journal: yes

characterization data reported in a peer-reviewed journal: yes

reference: Lee, Y. J. et al. Int J Dev Biol 52, 43-53 (2008)

---

hESC line: **AMC-hES2**

special features: derived in serum free medium

published in a peer-reviewed journal: yes

characterization data reported in a peer-reviewed journal: yes

reference: Lee, Y. J. et al. Int J Dev Biol 52, 43-53 (2008)

Provider: Maria Infertility Hospital Medical Institute

hESC line: **MB01**

special features:

published in a peer-reviewed journal: yes

characterization data reported in a peer-reviewed journal: yes

reference: Park, S.-P. et al. Hum Reprod 19, 676-684 (2004)
NIH Human Embryonic Stem Cell Registry (http://grants.nih.gov/stem_cells/registry/current.htm)

---

hESC line: **MB02**

special features:

published in a peer-reviewed journal: yes

characterization data reported in a peer-reviewed journal: yes

reference: Park, S.-P. et al. Hum Reprod 19, 676-684 (2004)
NIH Human Embryonic Stem Cell Registry (http://grants.nih.gov/stem_cells/registry/current.htm)

---

hESC line: **MB03**

special features:

published in a peer-reviewed journal:	yes
characterization data reported in a peer-reviewed journal:	yes
reference:	Park, S.-P. et al. Hum Reprod 19, 676-684 (2004)<br>NIH Human Embryonic Stem Cell Registry (http://grants.nih.gov/stem_cells/registry/current.htm)

hESC line:	**MB04**
special features:	
published in a peer-reviewed journal:	yes
characterization data reported in a peer-reviewed journal:	yes
reference:	Park, S.-P. et al. Hum Reprod 19, 676-684 (2004)

hESC line:	**MB05**
special features:	
published in a peer-reviewed journal:	yes
characterization data reported in a peer-reviewed journal:	yes
reference:	Park, S.-P. et al. Hum Reprod 19, 676-684 (2004)

hESC line:	**MB06**
special features:	
published in a peer-reviewed journal:	yes
characterization data reported in a peer-reviewed journal:	yes
reference:	Park, S.-P. et al. Hum Reprod 19, 676-684 (2004)

hESC line:	**MB07**
special features:	
published in a peer-reviewed journal:	yes
characterization data reported in a peer-reviewed journal:	yes
reference:	Park, S.-P. et al. Hum Reprod 19, 676-684 (2004)

hESC line:	**MB08**
special features:	

published in a peer-reviewed journal:	yes
characterization data reported in a peer-reviewed journal:	yes
reference:	Park, S.-P. et al. Hum Reprod 19, 676-684 (2004)

hESC line:	**MB09**
special features:	
published in a peer-reviewed journal:	yes
characterization data reported in a peer-reviewed journal:	yes
reference:	Park, S.-P. et al. Hum Reprod 19, 676-684 (2004)

Provider: MizMedi Hospital, Seoul National University

hESC line:	**Miz-hES1**
special features:	
published in a peer-reviewed journal:	yes
characterization data reported in a peer-reviewed journal:	yes
reference:	Park, J. H. et al. Biol Reprod 69, 2007-2014 (2003)<br>NIH Human Embryonic Stem Cell Registry (http://grants.nih.gov/stem_cells/registry/current.htm)

hESC line:	**Miz-hES10**
special features:	derived in serum free medium
published in a peer-reviewed journal:	yes
characterization data reported in a peer-reviewed journal:	yes
reference:	Kim, S. J. et al. Mol Cells 19, 46-53 (2005)

hESC line:	**Miz-hES11**
special features:	derived in serum free medium
published in a peer-reviewed journal:	yes
characterization data reported in a peer-reviewed journal:	yes
reference:	Kim, S. J. et al. Mol Cells 19, 46-53 (2005)

hESC line:	**Miz-hES12**
special features:	derived in serum free medium

published in a peer-reviewed journal:	yes
characterization data reported in a peer-reviewed journal:	yes
reference:	Kim, S. J. et al. Mol Cells 19, 46-53 (2005)

hESC line:	**Miz-hES13**
special features:	abnormal karyotype<br>derived in serum free medium
published in a peer-reviewed journal:	yes
characterization data reported in a peer-reviewed journal:	yes
reference:	Kim, S. J. et al. Mol Cells 19, 46-53 (2005)

hESC line:	**Miz-hES14**
special features:	derived in serum free medium<br>derived on human feeder cells
published in a peer-reviewed journal:	yes
characterization data reported in a peer-reviewed journal:	yes
reference:	Lee, J. B. et al. Biol Reprod 72, 42-49 (2005)

hESC line:	**Miz-hES15**
special features:	derived in serum free medium<br>derived on human feeder cells
published in a peer-reviewed journal:	yes
characterization data reported in a peer-reviewed journal:	yes
reference:	Lee, J. B. et al. Biol Reprod 72, 42-49 (2005)

hESC line:	**Miz-hES2**
special features:	
published in a peer-reviewed journal:	yes
characterization data reported in a peer-reviewed journal:	yes
reference:	Park, J. H. et al. Biol Reprod 69, 2007-2014 (2003)

hESC line:	**Miz-hES3**
special features:	

published in a peer-reviewed journal:	yes
characterization data reported in a peer-reviewed journal:	yes
reference:	Park, J. H. et al. Biol Reprod 69, 2007-2014 (2003)

---

hESC line:	**Miz-hES4**
special features:	derived in serum free medium
published in a peer-reviewed journal:	yes
characterization data reported in a peer-reviewed journal:	yes
reference:	Kim, S. J. et al. Mol Cells 19, 46-53 (2005)

---

hESC line:	**Miz-hES5**
special features:	derived in serum free medium<br>identical to Miz-hES1
published in a peer-reviewed journal:	yes
characterization data reported in a peer-reviewed journal:	yes
reference:	Kim, S. J. et al. Mol Cells 19, 46-53 (2005)

---

hESC line:	**Miz-hES6**
special features:	derived in serum free medium
published in a peer-reviewed journal:	yes
characterization data reported in a peer-reviewed journal:	yes
reference:	Kim, S. J. et al. Mol Cells 19, 46-53 (2005)

---

hESC line:	**Miz-hES7**
special features:	derived in serum free medium
published in a peer-reviewed journal:	yes
characterization data reported in a peer-reviewed journal:	yes
reference:	Kim, S. J. et al. Mol Cells 19, 46-53 (2005)

---

hESC line:	**Miz-hES8**
special features:	derived in serum free medium

published in a peer-reviewed journal:	yes
characterization data reported in a peer-reviewed journal:	yes
reference:	Kim, S. J. et al. Mol Cells 19, 46-53 (2005)

hESC line:	**Miz-hES9**
special features:	derived in serum free medium<br>derived on human feeder cells
published in a peer-reviewed journal:	yes
characterization data reported in a peer-reviewed journal:	yes
reference:	Lee, J. B. et al. Biol Reprod 72, 42-49 (2005)

Provider: Pochon CHA University College of Medicine, Seoul

hESC line:	**CHAhES-1**
special features:	
published in a peer-reviewed journal:	no
characterization data reported in a peer-reviewed journal:	no
reference:	NIH Human Embryonic Stem Cell Registry (http://grants.nih.gov/stem_cells/registry/current.htm)

hESC line:	**CHAhES-2**
special features:	
published in a peer-reviewed journal:	no
characterization data reported in a peer-reviewed journal:	no
reference:	NIH Human Embryonic Stem Cell Registry (http://grants.nih.gov/stem_cells/registry/current.htm)

hESC line:	**CHAhES-3**
special features:	
published in a peer-reviewed journal:	yes
characterization data reported in a peer-reviewed journal:	no
reference:	Ahn, S.E. et al. Biochem Biophys Res Commun 340, 403-438 (2006)

hESC line:	**CHAhES-4**
special features:	

published in a peer-reviewed journal:	yes
characterization data reported in a peer-reviewed journal:	no
reference:	Kim, M. S. et al., Lab Chip 7, 513-515 (2007)

hESC line:	**CHAhES-6**
special features:	
published in a peer-reviewed journal:	yes
characterization data reported in a peer-reviewed journal:	no
reference:	Woo, D. G. et al. Biomaterials 30, 5631-5638 (2009)

Provider: Seoul National University

hESC line:	**SNUhES1**
special features:	derived in serum free medium
published in a peer-reviewed journal:	yes
characterization data reported in a peer-reviewed journal:	yes
reference:	Oh, S. K. et al. Stem Cells 23, 211-219 (2005)

hESC line:	**SNUhES11**
special features:	
published in a peer-reviewed journal:	yes
characterization data reported in a peer-reviewed journal:	no
reference:	Seol, H. W. et al. Chromosome Res 16, 1075-1084 (2008)

hESC line:	**SNuhES16**
special features:	
published in a peer-reviewed journal:	yes
characterization data reported in a peer-reviewed journal:	no
reference:	Cho, M. S. et al. Proc Natl Acad Sci U S A 105, 3392-3397 (2008)

hESC line:	**SNUhES2**
special features:	derived in serum free medium

published in a peer-reviewed journal:	yes
characterization data reported in a peer-reviewed journal:	yes
reference:	Oh, S. K. et al. Stem Cells 23, 211-219 (2005)

hESC line:	**SNUhES3**
special features:	derived by whole embryo culture<br>derived in serum free medium
published in a peer-reviewed journal:	yes
characterization data reported in a peer-reviewed journal:	yes
reference:	Oh, S. K. et al. Stem Cells 23, 211-219 (2005)

hESC line:	**SNUhES4**
special features:	
published in a peer-reviewed journal:	yes
characterization data reported in a peer-reviewed journal:	no
reference:	Kwon, Y.D. et al. Mol Ther 12, 28-32 (2005)

NETHERLANDS

Provider: Hubrecht Laboratory, Utrecht, The Netherlands

hESC line:	**hESC-NL1 (formerly NL-hESC1)**
special features:	mechanical ICM isolation
published in a peer-reviewed journal:	yes
characterization data reported in a peer-reviewed journal:	yes
reference:	van de Stolpe, A. et al. Reprod Biomed Online 11, 476–485 (2005)

hESC line:	**HESC-NL2 (formerly NL-hESC2)**
special features:	
published in a peer-reviewed journal:	yes
characterization data reported in a peer-reviewed journal:	no
reference:	European Union hESC registry (http://www.hescreg.eu/)

hESC line:	**HESC-NL3**
special features:	derived in serum free medium<br>derived on human feeder cells<br>mechanical ICM isolation
published in a peer-reviewed journal:	yes
characterization data reported in a peer-reviewed journal:	yes
reference:	Freund, C. et al. Stem Cells 26, 724 (2008)

hESC line:	**HESC-NL4**
special features:	derived in serum free medium<br>derived on human feeder cells<br>mechanical ICM isolation
published in a peer-reviewed journal:	yes
characterization data reported in a peer-reviewed journal:	yes
reference:	Freund, C. et al. Stem Cells 26, 724 (2008)

RUSSIA

Provider: Institute of Cytology of the Russian Academy of Sciences, St. Petersburg

hESC line:	**C612**
special features:	
published in a peer-reviewed journal:	no
characterization data reported in a peer-reviewed journal:	no
reference:	Kozhucharova, I. V. et al., Tsitologiia 51, 551-558 (2009) (article in Russian)

hESC line:	**C910**
special features:	
published in a peer-reviewed journal:	no
characterization data reported in a peer-reviewed journal:	no
reference:	Kozhucharova, I. V. et al., Tsitologiia 51, 551-558 (2009) (article in Russian)

hESC line:	**HESC-1**
special features:	

published in a peer-reviewed journal:	no
characterization data reported in a peer-reviewed journal:	no
reference:	Krylova, T.A., et al. Tsitologiia 45, 1172-1178 (2003) (article in Russian)

hESC line:	**HESC-2**
special features:	
published in a peer-reviewed journal:	no
characterization data reported in a peer-reviewed journal:	no
reference:	Krylova, T.A., et al. Tsitologiia 45, 1172-1178 (2003) (article in Russian)

hESC line:	**HESC-3**
special features:	
published in a peer-reviewed journal:	no
characterization data reported in a peer-reviewed journal:	no
reference:	Krylova, T.A., et al. Tsitologiia 45, 1172-1178 (2003) (article in Russian)

hESC line:	**HESC-4**
special features:	
published in a peer-reviewed journal:	no
characterization data reported in a peer-reviewed journal:	no
reference:	Krylova, T.A., et al. Tsitologiia 45, 1172-1178 (2003) (article in Russian)

hESC line:	**HESC-5**
special features:	
published in a peer-reviewed journal:	no
characterization data reported in a peer-reviewed journal:	no
reference:	Krylova, T.A., et al. Tsitologiia 47, 121-129 (2005) (article in Russian)

hESC line:	**SC1**
special features:	

published in a peer-reviewed journal:	no
characterization data reported in a peer-reviewed journal:	no
reference:	Krylova, T.A., et al. Tsitologiia 51, 565-576 (2009) (article in Russian)

hESC line:	**SC2**
special features:	
published in a peer-reviewed journal:	no
characterization data reported in a peer-reviewed journal:	no
reference:	Krylova, T.A., et al. Tsitologiia 51, 565-576 (2009) (article in Russian)

hESC line:	**SC3**
special features:	
published in a peer-reviewed journal:	no
characterization data reported in a peer-reviewed journal:	no
reference:	Krylova, T.A., et al. Tsitologiia 51, 565-576 (2009) (article in Russian)

hESC line:	**SC4**
special features:	
published in a peer-reviewed journal:	no
characterization data reported in a peer-reviewed journal:	no
reference:	Krylova, T.A., et al. Tsitologiia 51, 565-576 (2009) (article in Russian)

Provider: Russian Academy of Sciences, Moscow

hESC line:	**ESM01**
special features:	derived in serum free medium<br>derived without feeder cells
published in a peer-reviewed journal:	yes
characterization data reported in a peer-reviewed journal:	no
reference:	Lagarkova, M. A. et al. Cell Cycle 5, 416-420 (2006)

hESC line:	**ESM02**
special features:	

published in a peer-reviewed journal:	yes
characterization data reported in a peer-reviewed journal:	no
reference:	Lagarkova, M. A. et al. Cell Cycle 5, 416-420 (2006)

hESC line:	**ESM03**
special features:	
published in a peer-reviewed journal:	yes
characterization data reported in a peer-reviewed journal:	no
reference:	Lagarkova, M. A. et al. Cell Cycle 5, 416-420 (2006)

hESC line:	**ESM04**
special features:	
published in a peer-reviewed journal:	yes
characterization data reported in a peer-reviewed journal:	no
reference:	Prokhorovich, M. A. et al. Bull Exp Biol Med 144, 126-129 (2007)

hESC line:	**hESKM-5**
special features:	abnormal karyotype<br>derived in serum free medium<br>derived without feeder cells
published in a peer-reviewed journal:	yes
characterization data reported in a peer-reviewed journal:	yes
reference:	Eremeev, A. V. et al. Dokl Biol Sci 426, 293-295 (2009)

SINGAPORE

Provider: ES Cell International

hESC line:	**ESI-013**
special features:	abnormal karyotype<br>derived under GMP conditions<br>xeno-free derivation
published in a peer-reviewed journal:	yes
characterization data reported in a peer-reviewed journal:	yes
reference:	Crook, J. M. et al. Cell Stem Cell 1, 490-494 (2007)

hESC line:	**ESI-014**
special features:	derived under GMP conditions<br>xeno-free derivation
published in a peer-reviewed journal:	yes
characterization data reported in a peer-reviewed journal:	yes
reference:	Crook, J. M. et al. Cell Stem Cell 1, 490-494 (2007)

---

hESC line:	**ESI-017**
special features:	derived under GMP conditions
published in a peer-reviewed journal:	yes
characterization data reported in a peer-reviewed journal:	yes
reference:	Crook, J. M. et al. Cell Stem Cell 1, 490-494 (2007)

---

hESC line:	**ESI-027**
special features:	abnormal karyotype<br>derived under GMP conditions<br>xeno-free derivation
published in a peer-reviewed journal:	yes
characterization data reported in a peer-reviewed journal:	yes
reference:	Crook, J. M. et al. Cell Stem Cell 1, 490-494 (2007)

---

hESC line:	**ESI-035**
special features:	derived under GMP conditions<br>xeno-free derivation
published in a peer-reviewed journal:	yes
characterization data reported in a peer-reviewed journal:	yes
reference:	Crook, J. M. et al. Cell Stem Cell 1, 490-494 (2007)

---

hESC line:	**ESI-049**
special features:	derived under GMP conditions<br>xeno-free derivation

published in a peer-reviewed journal:	yes
characterization data reported in a peer-reviewed journal:	yes
reference:	Crook, J. M. et al. Cell Stem Cell 1, 490-494 (2007)

hESC line:	**ESI-051**
special features:	derived under GMP conditions<br>xeno-free derivation
published in a peer-reviewed journal:	yes
characterization data reported in a peer-reviewed journal:	yes
reference:	Crook, J. M. et al. Cell Stem Cell 1, 490-494 (2007)

hESC line:	**ESI-053**
special features:	derived under GMP conditions<br>xeno-free derivation
published in a peer-reviewed journal:	yes
characterization data reported in a peer-reviewed journal:	yes
reference:	Crook, J. M. et al. Cell Stem Cell 1, 490-494 (2007)

hESC line:	**HES-1**
special features:	
published in a peer-reviewed journal:	yes
characterization data reported in a peer-reviewed journal:	yes
reference:	Reubinoff, B. E. et al. Nat Biotechnol 18, 399-404 (2000)<br>NIH Human Embryonic Stem Cell Registry (http://grants.nih.gov/stem_cells/registry/current.htm)

hESC line:	**HES-2**
special features:	
published in a peer-reviewed journal:	yes
characterization data reported in a peer-reviewed journal:	yes
reference:	Reubinoff, B. E. et al. Nat Biotechnol 18, 399-404 (2000)<br>NIH Human Embryonic Stem Cell Registry (http://grants.nih.gov/stem_cells/registry/current.htm)

hESC line:	**HES-3**
special features:	
published in a peer-reviewed journal:	yes
characterization data reported in a peer-reviewed journal:	yes
reference:	NIH Human Embryonic Stem Cell Registry (http://grants.nih.gov/stem_cells/registry/current.htm)

hESC line:	**HES-4**
special features:	
published in a peer-reviewed journal:	yes
characterization data reported in a peer-reviewed journal:	yes
reference:	NIH Human Embryonic Stem Cell Registry (http://grants.nih.gov/stem_cells/registry/current.htm)

hESC line:	**HES-5**
special features:	
published in a peer-reviewed journal:	yes
characterization data reported in a peer-reviewed journal:	yes
reference:	NIH Human Embryonic Stem Cell Registry (http://grants.nih.gov/stem_cells/registry/current.htm)

hESC line:	**HES-6**
special features:	
published in a peer-reviewed journal:	yes
characterization data reported in a peer-reviewed journal:	yes
reference:	NIH Human Embryonic Stem Cell Registry (http://grants.nih.gov/stem_cells/registry/current.htm)

Provider: National University of Singapore, Kent Ridge

hESC line:	**n.n.**
special features:	derived in media containing human serum<br>derived on human feeder cells
published in a peer-reviewed journal:	yes
characterization data reported in a peer-reviewed journal:	yes
reference:	Richards, M. et al. Nat Biotechnol 20, 933-936 (2002)

SPAIN

Provider: Andalusian Stem Cell Bank, Granada, Spain

hESC line:	**AND-1**
special features:	derived by whole embryo culture<br>derived on human feeder cells<br>laser-assisted ICM isolation
published in a peer-reviewed journal:	yes
characterization data reported in a peer-reviewed journal:	yes
reference:	Cortes, J. L. et al. Hum Reprod 24, 1844-1851 (2009)

hESC line:	**AND-2**
special features:	derived by whole embryo culture<br>derived on human feeder cells
published in a peer-reviewed journal:	yes
characterization data reported in a peer-reviewed journal:	yes
reference:	Cortes, J. L. et al. Hum Reprod 24, 1844-1851 (2009)

hESC line:	**AND-3**
special features:	derived by whole embryo culture<br>derived on human feeder cells
published in a peer-reviewed journal:	yes
characterization data reported in a peer-reviewed journal:	yes
reference:	Cortes, J. L. et al. Hum Reprod 24, 1844-1851 (2009)

Provider: Center for Regenerative Medicine in Barcedlona, Spain

hESC line:	**ES[2]**
special features:	derived in serum free medium<br>derived on human feeder cells
published in a peer-reviewed journal:	yes
characterization data reported in a peer-reviewed journal:	yes
reference:	Raya, A. et al. Cold Spring Harb Symp Quant Biol 73, 127-135 (2008

hESC line:	**ES[3]**
special features:	derived in serum free medium<br>derived on human feeder cells

published in a peer-reviewed journal:	yes
characterization data reported in a peer-reviewed journal:	yes
reference:	Raya, A. et al. Cold Spring Harb Symp Quant Biol 73, 127-135 (2008

hESC line:	**ES[4]**
special features:	derived in serum free medium<br>derived on human feeder cells
published in a peer-reviewed journal:	yes
characterization data reported in a peer-reviewed journal:	yes
reference:	Raya, A. et al. Cold Spring Harb Symp Quant Biol 73, 127-135 (2008

hESC line:	**ES[5]**
special features:	derived in serum free medium<br>derived on human feeder cells
published in a peer-reviewed journal:	yes
characterization data reported in a peer-reviewed journal:	yes
reference:	Raya, A. et al. Cold Spring Harb Symp Quant Biol 73, 127-135 (2008

hESC line:	**ES[6]**
special features:	derived in serum free medium<br>derived on human feeder cells
published in a peer-reviewed journal:	yes
characterization data reported in a peer-reviewed journal:	yes
reference:	Raya, A. et al. Cold Spring Harb Symp Quant Biol 73, 127-135 (2008

Provider: Valencia Stem Cell Bank

hESC line:	**VAL-1**
special features:	derived by whole embryo culture<br>derived in serum free medium<br>derived on human feeder cells
published in a peer-reviewed journal:	yes
characterization data reported in a peer-reviewed journal:	yes
reference:	Simon, C. et al. Fertil Steril 83, 246-249 (2005)

hESC line:	**VAL-10B**
special features:	
published in a peer-reviewed journal:	no
characterization data reported in a peer-reviewed journal:	no
reference:	European Union hESC registry (http://www.hescreg.eu/)

---

hESC line:	**VAL-2**
special features:	derived by whole embryo culture<br>derived in serum free medium<br>derived on human feeder cells
published in a peer-reviewed journal:	yes
characterization data reported in a peer-reviewed journal:	yes
reference:	Simon, C. et al. Fertil Steril 83, 246-249 (2005)

---

hESC line:	**VAL-3**
special features:	derived by whole embryo culture<br>derived in serum free medium<br>derived on human feeder cells
published in a peer-reviewed journal:	yes
characterization data reported in a peer-reviewed journal:	yes
reference:	Valbuena, D. et al. Reprod Biomed Online 13, 875-886 (2006)

---

hESC line:	**VAL-4**
special features:	derived by whole embryo culture<br>derived in serum free medium<br>derived on human feeder cells
published in a peer-reviewed journal:	yes
characterization data reported in a peer-reviewed journal:	yes
reference:	Valbuena, D. et al. Reprod Biomed Online 13, 875-886 (2006)

---

hESC line:	**VAL-5**
special features:	derived by whole embryo culture<br>derived in serum free medium<br>derived on human feeder cells

published in a peer-reviewed journal:	yes
characterization data reported in a peer-reviewed journal:	yes
reference:	Valbuena, D. et al. Reprod Biomed Online 13, 875-886 (2006)

hESC line:	**Val-6M**
special features:	
published in a peer-reviewed journal:	no
characterization data reported in a peer-reviewed journal:	no
reference:	European Union hESC registry (http://www.hescreg.eu/)

hESC line:	**Val-7**
special features:	
published in a peer-reviewed journal:	no
characterization data reported in a peer-reviewed journal:	no
reference:	European Union hESC registry (http://www.hescreg.eu/)

hESC line:	**Val-8**
special features:	
published in a peer-reviewed journal:	no
characterization data reported in a peer-reviewed journal:	no
reference:	European Union hESC registry (http://www.hescreg.eu/)

hESC line:	**Val-9**
special features:	
published in a peer-reviewed journal:	no
characterization data reported in a peer-reviewed journal:	no
reference:	European Union hESC registry (http://www.hescreg.eu/)

SWEDEN

Provider: Cellartis AB

hESC line:	**AS034**
special features:	

published in a peer-reviewed journal:	yes
characterization data reported in a peer-reviewed journal:	yes
reference:	Heins, N. et al. Stem Cells 22, 367-376 (2004)

hESC line:	**AS038**
special features:	
published in a peer-reviewed journal:	yes
characterization data reported in a peer-reviewed journal:	yes
reference:	Heins, N. et al. Stem Cells 22, 367-376 (2004)

hESC line:	**AS079**
special features:	
published in a peer-reviewed journal:	no
characterization data reported in a peer-reviewed journal:	no
reference:	European Union hESC registry (http://www.hescreg.eu/)

hESC line:	**AS094 / SA094**
special features:	
published in a peer-reviewed journal:	yes
characterization data reported in a peer-reviewed journal:	no
reference:	hESC Registry of the Stem Cell Community (http://www.stemcellcommunity.org/)<br>Hansson, M. et al. Diabetes 53, 2603-2609 (2004)

hESC line:	**FC018**
special features:	abnormal karyotype
published in a peer-reviewed journal:	yes
characterization data reported in a peer-reviewed journal:	yes
reference:	Heins, N. et al. Stem Cells 22, 367-376 (2004)

hESC line:	**SA046**
special features:	

published in a peer-reviewed journal:	yes
characterization data reported in a peer-reviewed journal:	no
reference:	Molne, J. et al.Transplantation 86, 1407-1413 (2008)

hESC line:	**SA085**
special features:	
published in a peer-reviewed journal:	no
characterization data reported in a peer-reviewed journal:	no
reference:	hESC Registry of the Stem Cell Community (http://www.stemcellcommunity.org/)

hESC line:	**SA111**
special features:	
published in a peer-reviewed journal:	no
characterization data reported in a peer-reviewed journal:	no
reference:	European Union hESC registry (http://www.hescreg.eu/)

hESC line:	**SA121**
special features:	
published in a peer-reviewed journal:	yes
characterization data reported in a peer-reviewed journal:	yes
reference:	Heins, N. et al. Stem Cells 22, 367-376 (2004)

hESC line:	**SA142**
special features:	
published in a peer-reviewed journal:	no
characterization data reported in a peer-reviewed journal:	no
reference:	European Union hESC registry (http://www.hescreg.eu/)

hESC line:	**SA167**
special features:	

published in a peer-reviewed journal:	yes
characterization data reported in a peer-reviewed journal:	yes
reference:	Sjögren-Jansson, E. et al. Dev Dyn 233, 1304-1314 (2005)

hESC line:	**SA181**
special features:	
published in a peer-reviewed journal:	yes
characterization data reported in a peer-reviewed journal:	yes
reference:	Heins, N. et al. Stem Cells 22, 367-376 (2004)

hESC line:	**SA191**
special features:	
published in a peer-reviewed journal:	no
characterization data reported in a peer-reviewed journal:	no
reference:	European Union hESC registry (http://www.hescreg.eu/)

hESC line:	**SA196**
special features:	
published in a peer-reviewed journal:	no
characterization data reported in a peer-reviewed journal:	no
reference:	European Union hESC registry (http://www.hescreg.eu/)

hESC line:	**SA202**
special features:	
published in a peer-reviewed journal:	yes
characterization data reported in a peer-reviewed journal:	no
reference:	Noaksson, K. et al. Stem Cells 23, 1460-1467 (2005)

hESC line:	**SA203**
special features:	

published in a peer-reviewed journal:	no
characterization data reported in a peer-reviewed journal:	no
reference:	European Union hESC registry (http://www.hescreg.eu/)

hESC line:	**SA211**
special features:	
published in a peer-reviewed journal:	no
characterization data reported in a peer-reviewed journal:	no
reference:	hESC Registry of the Stem Cell Community (http://www.stemcellcommunity.org/)

hESC line:	**SA218**
special features:	
published in a peer-reviewed journal:	no
characterization data reported in a peer-reviewed journal:	no
reference:	hESC Registry of the Stem Cell Community (http://www.stemcellcommunity.org/)

hESC line:	**SA240**
special features:	
published in a peer-reviewed journal:	no
characterization data reported in a peer-reviewed journal:	no
reference:	European Union hESC registry (http://www.hescreg.eu/)

hESC line:	**SA279**
special features:	
published in a peer-reviewed journal:	no
characterization data reported in a peer-reviewed journal:	no
reference:	hESC Registry of the Stem Cell Community (http://www.stemcellcommunity.org/)

hESC line:	**SA348**
special features:	

published in a peer-reviewed journal:	yes
characterization data reported in a peer-reviewed journal:	no
reference:	Molne, J. et al.Transplantation 86, 1407-1413 (2008)

hESC line:	**SA352**
special features:	
published in a peer-reviewed journal:	no
characterization data reported in a peer-reviewed journal:	no
reference:	hESC Registry of the Stem Cell Community (http://www.stemcellcommunity.org/)

hESC line:	**SA399**
special features:	
published in a peer-reviewed journal:	no
characterization data reported in a peer-reviewed journal:	no
reference:	hESC Registry of the Stem Cell Community (http://www.stemcellcommunity.org/)

hESC line:	**SA461**
special features:	
published in a peer-reviewed journal:	yes
characterization data reported in a peer-reviewed journal:	yes
reference:	Darnfors, C. et al. Stem Cells 23, 483-488 (2005)

hESC line:	**SA502**
special features:	
published in a peer-reviewed journal:	no
characterization data reported in a peer-reviewed journal:	no
reference:	European Union hESC registry (http://www.hescreg.eu/)

hESC line:	**SA521**
special features:	

published in a peer-reviewed journal:	no
characterization data reported in a peer-reviewed journal:	no
reference:	European Union hESC registry (http://www.hescreg.eu/)

hESC line:	**SA540**
special features:	
published in a peer-reviewed journal:	no
characterization data reported in a peer-reviewed journal:	no
reference:	European Union hESC registry (http://www.hescreg.eu/)

hESC line:	**SA606**
special features:	
published in a peer-reviewed journal:	no
characterization data reported in a peer-reviewed journal:	no
reference:	European Union hESC registry (http://www.hescreg.eu/)

hESC line:	**SA611**
special features:	xeno-free derivation
published in a peer-reviewed journal:	yes
characterization data reported in a peer-reviewed journal:	yes
reference:	Ellerström, C. et al. Stem Cells 24, 2170-2176 (2006)

hESC line:	**Sahlgrenska 1 (SA001)**
special features:	xeno-free derivation
published in a peer-reviewed journal:	yes
characterization data reported in a peer-reviewed journal:	yes
reference:	Hansson, M. et al. Diabetes 53, 2603-2609 (2004)

hESC line:	**Sahlgrenska 2 (SA002)**
special features:	abnormal karyotype

published in a peer-reviewed journal: yes

characterization data reported in a peer-reviewed journal: yes

reference: Heins, N. et al. Stem Cells 22, 367-376 (2004)
NIH Human Embryonic Stem Cell Registry (http://grants.nih.gov/stem_cells/registry/current.htm)

---

hESC line: **Sahlgrenska 3**

special features:

published in a peer-reviewed journal: yes

characterization data reported in a peer-reviewed journal: no

reference: Tallheden, T. et al. Life Sci 79, 999-1006 (2006)

Provider: Göteburg University

hESC line: **Sahlgrenska 10**

special features:

published in a peer-reviewed journal: no

characterization data reported in a peer-reviewed journal: no

reference: NIH Human Embryonic Stem Cell Registry (http://grants.nih.gov/stem_cells/registry/current.htm)

---

hESC line: **Sahlgrenska 11**

special features:

published in a peer-reviewed journal: no

characterization data reported in a peer-reviewed journal: no

reference: NIH Human Embryonic Stem Cell Registry (http://grants.nih.gov/stem_cells/registry/current.htm)

---

hESC line: **Sahlgrenska 12**

special features:

published in a peer-reviewed journal: no

characterization data reported in a peer-reviewed journal: no

reference: NIH Human Embryonic Stem Cell Registry (http://grants.nih.gov/stem_cells/registry/current.htm)

---

hESC line: **Sahlgrenska 13**

special features:

published in a peer-reviewed journal:	no
characterization data reported in a peer-reviewed journal:	no
reference:	NIH Human Embryonic Stem Cell Registry (http://grants.nih.gov/stem_cells/registry/current.htm)

hESC line:	**Sahlgrenska 14**
special features:	
published in a peer-reviewed journal:	no
characterization data reported in a peer-reviewed journal:	no
reference:	NIH Human Embryonic Stem Cell Registry (http://grants.nih.gov/stem_cells/registry/current.htm)

hESC line:	**Sahlgrenska 15**
special features:	
published in a peer-reviewed journal:	no
characterization data reported in a peer-reviewed journal:	no
reference:	NIH Human Embryonic Stem Cell Registry (http://grants.nih.gov/stem_cells/registry/current.htm)

hESC line:	**Sahlgrenska 16**
special features:	
published in a peer-reviewed journal:	no
characterization data reported in a peer-reviewed journal:	no
reference:	NIH Human Embryonic Stem Cell Registry (http://grants.nih.gov/stem_cells/registry/current.htm)

hESC line:	**Sahlgrenska 17**
special features:	
published in a peer-reviewed journal:	no
characterization data reported in a peer-reviewed journal:	no
reference:	NIH Human Embryonic Stem Cell Registry (http://grants.nih.gov/stem_cells/registry/current.htm)

hESC line:	**Sahlgrenska 18**
special features:	

published in a peer-reviewed journal:	no
characterization data reported in a peer-reviewed journal:	no
reference:	NIH Human Embryonic Stem Cell Registry (http://grants.nih.gov/stem_cells/registry/current.htm)

hESC line:	**Sahlgrenska 19**
special features:	
published in a peer-reviewed journal:	no
characterization data reported in a peer-reviewed journal:	no
reference:	NIH Human Embryonic Stem Cell Registry (http://grants.nih.gov/stem_cells/registry/current.htm)

hESC line:	**Sahlgrenska 4**
special features:	
published in a peer-reviewed journal:	no
characterization data reported in a peer-reviewed journal:	no
reference:	NIH Human Embryonic Stem Cell Registry (http://grants.nih.gov/stem_cells/registry/current.htm)

hESC line:	**Sahlgrenska 5**
special features:	
published in a peer-reviewed journal:	no
characterization data reported in a peer-reviewed journal:	no
reference:	NIH Human Embryonic Stem Cell Registry (http://grants.nih.gov/stem_cells/registry/current.htm)

hESC line:	**Sahlgrenska 6**
special features:	
published in a peer-reviewed journal:	no
characterization data reported in a peer-reviewed journal:	no
reference:	NIH Human Embryonic Stem Cell Registry (http://grants.nih.gov/stem_cells/registry/current.htm)

hESC line:	**Sahlgrenska 7**
special features:	

published in a peer-reviewed journal:	no
characterization data reported in a peer-reviewed journal:	no
reference:	NIH Human Embryonic Stem Cell Registry (http://grants.nih.gov/stem_cells/registry/current.htm)

hESC line:	**Sahlgrenska 8**
special features:	
published in a peer-reviewed journal:	no
characterization data reported in a peer-reviewed journal:	no
reference:	NIH Human Embryonic Stem Cell Registry (http://grants.nih.gov/stem_cells/registry/current.htm)

hESC line:	**Sahlgrenska 9**
special features:	
published in a peer-reviewed journal:	no
characterization data reported in a peer-reviewed journal:	no
reference:	NIH Human Embryonic Stem Cell Registry (http://grants.nih.gov/stem_cells/registry/current.htm)

Provider: Karolinska Institut

hESC line:	**hICM40**
special features:	
published in a peer-reviewed journal:	no
characterization data reported in a peer-reviewed journal:	no
reference:	NIH Human Embryonic Stem Cell Registry (http://grants.nih.gov/stem_cells/registry/current.htm)

hESC line:	**hICM41**
special features:	
published in a peer-reviewed journal:	no
characterization data reported in a peer-reviewed journal:	no
reference:	NIH Human Embryonic Stem Cell Registry (http://grants.nih.gov/stem_cells/registry/current.htm)

hESC line:	**hICM42**
special features:	

published in a peer-reviewed journal:	no
characterization data reported in a peer-reviewed journal:	no
reference:	NIH Human Embryonic Stem Cell Registry (http://grants.nih.gov/stem_cells/registry/current.htm)

hESC line:	**hICM43**
special features:	
published in a peer-reviewed journal:	no
characterization data reported in a peer-reviewed journal:	no
reference:	NIH Human Embryonic Stem Cell Registry (http://grants.nih.gov/stem_cells/registry/current.htm)

hESC line:	**hICM8**
special features:	
published in a peer-reviewed journal:	no
characterization data reported in a peer-reviewed journal:	no
reference:	NIH Human Embryonic Stem Cell Registry (http://grants.nih.gov/stem_cells/registry/current.htm)

hESC line:	**hICM9**
special features:	
published in a peer-reviewed journal:	no
characterization data reported in a peer-reviewed journal:	no
reference:	NIH Human Embryonic Stem Cell Registry (http://grants.nih.gov/stem_cells/registry/current.htm)

hESC line:	**HS181**
special features:	derived on human feeder cells
published in a peer-reviewed journal:	yes
characterization data reported in a peer-reviewed journal:	yes
reference:	Hovatta, O. et al. Hum Reprod 18, 1404-1409 (2003)<br>Inzunza, J. et al. Mol Hum Reprod 10, 461-466 (2004)

hESC line:	**HS207**
special features:	derived on human feeder cells

published in a peer-reviewed journal:	yes
characterization data reported in a peer-reviewed journal:	yes
reference:	Hovatta, O. et al. Hum Reprod 18, 1404-1409 (2003)

hESC line:	**HS235**
special features:	derived on human feeder cells
published in a peer-reviewed journal:	yes
characterization data reported in a peer-reviewed journal:	yes
reference:	Inzunza, J. et al. Mol Hum Reprod 10, 461-466 (2004)

hESC line:	**HS237**
special features:	derived on human feeder cells
published in a peer-reviewed journal:	yes
characterization data reported in a peer-reviewed journal:	yes
reference:	Inzunza, J. et al. Mol Hum Reprod 10, 461-466 (2004)<br>Koivisto, H. et al. Reprod Biomed Online 9, 330-337 (2004)

hESC line:	**HS245**
special features:	derived in serum free medium<br>derived on human feeder cells
published in a peer-reviewed journal:	no
characterization data reported in a peer-reviewed journal:	no
reference:	O. Hovatta, Euroconference Paris 2005 (http://www.pasteur.fr/applications/euroconf/stemcells/hovatta.pdf)

hESC line:	**HS251**
special features:	derived in serum free medium<br>derived on human feeder cells
published in a peer-reviewed journal:	no
characterization data reported in a peer-reviewed journal:	no
reference:	O. Hovatta, Euroconference Paris 2005 (http://www.pasteur.fr/applications/euroconf/stemcells/hovatta.pdf)

hESC line:	**HS256**
special features:	derived in serum free medium<br>derived on human feeder cells

published in a peer-reviewed journal:	no
characterization data reported in a peer-reviewed journal:	no
reference:	O. Hovatta, Euroconference Paris 2005 (http://www.pasteur.fr/applications/euroconf/stemcells/hovatta.pdf)

hESC line:	**HS259**
special features:	derived in serum free medium<br>derived on human feeder cells
published in a peer-reviewed journal:	no
characterization data reported in a peer-reviewed journal:	no
reference:	O. Hovatta, Euroconference Paris 2005 (http://www.pasteur.fr/applications/euroconf/stemcells/hovatta.pdf)

hESC line:	**HS293**
special features:	derived in serum free medium<br>derived on human feeder cells
published in a peer-reviewed journal:	yes
characterization data reported in a peer-reviewed journal:	yes
reference:	Inzunza, J. et al. Stem Cells 23, 544-549 (2005)

hESC line:	**HS306**
special features:	derived in serum free medium<br>derived on human feeder cells
published in a peer-reviewed journal:	yes
characterization data reported in a peer-reviewed journal:	yes
reference:	Inzunza, J. et al. Stem Cells 23, 544-549 (2005)

hESC line:	**HS346**
special features:	derived in serum free medium<br>derived on human feeder cells
published in a peer-reviewed journal:	yes
characterization data reported in a peer-reviewed journal:	yes
reference:	Inzunza, J. et al. Stem Cells 23, 544-549 (2005)

hESC line:	**HS351**
special features:	derived in serum free medium<br>derived on human feeder cells
published in a peer-reviewed journal:	yes
characterization data reported in a peer-reviewed journal:	yes
reference:	Inzunza, J. et al. Stem Cells 23, 544-549 (2005)

hESC line:	**HS356**
special features:	derived in serum free medium<br>derived on human feeder cells
published in a peer-reviewed journal:	yes
characterization data reported in a peer-reviewed journal:	yes
reference:	Inzunza, J. et al. Stem Cells 23, 544-549 (2005)

hESC line:	**HS360**
special features:	derived in serum free medium<br>derived on human feeder cells
published in a peer-reviewed journal:	yes
characterization data reported in a peer-reviewed journal:	yes
reference:	Inzunza, J. et al. Stem Cells 23, 544-549 (2005)

hESC line:	**HS361**
special features:	derived in serum free medium<br>derived on human feeder cells
published in a peer-reviewed journal:	yes
characterization data reported in a peer-reviewed journal:	yes
reference:	Inzunza, J. et al. Stem Cells 23, 544-549 (2005)

hESC line:	**HS362**
special features:	derived in serum free medium<br>derived on human feeder cells

published in a peer-reviewed journal:	yes
characterization data reported in a peer-reviewed journal:	yes
reference:	Inzunza, J. et al. Stem Cells 23, 544-549 (2005)

hESC line:	**HS363**
special features:	derived in serum free medium<br>derived on human feeder cells
published in a peer-reviewed journal:	yes
characterization data reported in a peer-reviewed journal:	yes
reference:	Inzunza, J. et al. Stem Cells 23, 544-549 (2005)

hESC line:	**HS364**
special features:	derived in serum free medium<br>derived on human feeder cells
published in a peer-reviewed journal:	yes
characterization data reported in a peer-reviewed journal:	yes
reference:	Inzunza, J. et al. Stem Cells 23, 544-549 (2005)

hESC line:	**HS366**
special features:	derived in serum free medium<br>derived on human feeder cells
published in a peer-reviewed journal:	yes
characterization data reported in a peer-reviewed journal:	yes
reference:	Inzunza, J. et al. Stem Cells 23, 544-549 (2005)

hESC line:	**HS368**
special features:	derived in serum free medium<br>derived on human feeder cells
published in a peer-reviewed journal:	yes
characterization data reported in a peer-reviewed journal:	yes
reference:	Inzunza, J. et al. Stem Cells 23, 544-549 (2005)

hESC line:	**HS380**
special features:	derived in serum free medium<br>derived on human feeder cells
published in a peer-reviewed journal:	yes
characterization data reported in a peer-reviewed journal:	yes
reference:	Hovatta, O. Reprod Fertil Dev 18, 823-828 (2006)

---

hESC line:	**HS382**
special features:	derived in serum free medium<br>derived on human feeder cells
published in a peer-reviewed journal:	yes
characterization data reported in a peer-reviewed journal:	yes
reference:	Hovatta, O. Reprod Fertil Dev 18, 823-828 (2006)

---

hESC line:	**HS386**
special features:	derived in serum free medium<br>derived on human feeder cells
published in a peer-reviewed journal:	yes
characterization data reported in a peer-reviewed journal:	yes
reference:	Hovatta, O. Reprod Fertil Dev 18, 823-828 (2006)

---

hESC line:	**HS400**
special features:	derived in serum free medium<br>derived on human feeder cells
published in a peer-reviewed journal:	yes
characterization data reported in a peer-reviewed journal:	yes
reference:	Strom, S. et al. Hum Reprod 22, 3051-3058 (2007)

---

hESC line:	**HS401**
special features:	derived in serum free medium<br>derived on human feeder cells

published in a peer-reviewed journal:	yes
characterization data reported in a peer-reviewed journal:	yes
reference:	Hovatta, O. Reprod Fertil Dev 18, 823-828 (2006)

hESC line:	**HS402**
special features:	derived in serum free medium<br>derived on human feeder cells<br>mechanical ICM isolation
published in a peer-reviewed journal:	yes
characterization data reported in a peer-reviewed journal:	yes
reference:	Hovatta, O. Reprod Fertil Dev 18, 823-828 (2006)

hESC line:	**HS415**
special features:	derived in serum free medium<br>derived on human feeder cells<br>mechanical ICM isolation
published in a peer-reviewed journal:	yes
characterization data reported in a peer-reviewed journal:	yes
reference:	Strom, S. et al. Hum Reprod 22, 3051-3058 (2007)

hESC line:	**HS420**
special features:	derived in serum free medium<br>derived on human feeder cells<br>mechanical ICM isolation
published in a peer-reviewed journal:	yes
characterization data reported in a peer-reviewed journal:	yes
reference:	Strom, S. et al. Hum Reprod 22, 3051-3058 (2007)

hESC line:	**HS422**
special features:	derived in serum free medium<br>derived on human feeder cells<br>mechanical ICM isolation

published in a peer-reviewed journal:	yes
characterization data reported in a peer-reviewed journal:	yes
reference:	Strom, S. et al. Hum Reprod 22, 3051-3058 (2007)

---

hESC line:	**HS426**
special features:	derived in serum free medium<br>derived on human feeder cells<br>mechanical ICM isolation
published in a peer-reviewed journal:	yes
characterization data reported in a peer-reviewed journal:	yes
reference:	Strom, S. et al. Hum Reprod 22, 3051-3058 (2007)

---

hESC line:	**HS429**
special features:	derived in serum free medium<br>derived on human feeder cells<br>mechanical ICM isolation
published in a peer-reviewed journal:	yes
characterization data reported in a peer-reviewed journal:	yes
reference:	Strom, S. et al. Hum Reprod 22, 3051-3058 (2007)

---

hESC line:	**HS475**
special features:	derived in serum free medium<br>derived on human feeder cells<br>mechanical ICM isolation
published in a peer-reviewed journal:	yes
characterization data reported in a peer-reviewed journal:	no
reference:	Strom, S. et al. Hum Reprod 22, 3051-3058 (2007)

---

hESC line:	**HS480**
special features:	derived in serum free medium<br>derived on human feeder cells<br>mechanical ICM isolation

published in a peer-reviewed journal:	yes
characterization data reported in a peer-reviewed journal:	no
reference:	Strom, S. et al. Hum Reprod 22, 3051-3058 (2007)

hESC line:	**HS481**
special features:	derived in serum free medium<br>derived on human feeder cells<br>mechanical ICM isolation
published in a peer-reviewed journal:	yes
characterization data reported in a peer-reviewed journal:	no
reference:	Strom, S. et al. Hum Reprod 22, 3051-3058 (2007)

hESC line:	**HS491**
special features:	
published in a peer-reviewed journal:	no
characterization data reported in a peer-reviewed journal:	no
reference:	European Union hESC registry (http://www.hescreg.eu/)

hESC line:	**HS539**
special features:	
published in a peer-reviewed journal:	no
characterization data reported in a peer-reviewed journal:	no
reference:	European Union hESC registry (http://www.hescreg.eu/)

SWITZERLAND

Provider: Geneva University

hESC line:	**CH-ES1**
special features:	abnormal karyotype<br>mechanical ICM isolation
published in a peer-reviewed journal:	yes
characterization data reported in a peer-reviewed journal:	yes
reference:	Feki, A. et al. Swiss Med Wkly 138, 540-550 (2008)

TAIWAN, PROVINCE OF CHINA

Provider: China Medical University, Taichung, Taiwan

hESC line:	**TW1**
special features:	derived by whole embryo culture<br>derived in serum free medium
published in a peer-reviewed journal:	yes
characterization data reported in a peer-reviewed journal:	yes
reference:	Cheng, E. H. et al. Reprod Biomed Online 17, 436-444 (2008)

hESC line:	**TW2**
special features:	derived by whole embryo culture<br>derived in serum free medium
published in a peer-reviewed journal:	yes
characterization data reported in a peer-reviewed journal:	yes
reference:	Cheng, E. H. et al. Reprod Biomed Online 17, 436-444 (2008)

hESC line:	**TW3**
special features:	derived by whole embryo culture<br>derived in serum free medium
published in a peer-reviewed journal:	yes
characterization data reported in a peer-reviewed journal:	yes
reference:	Cheng, E. H. et al. Reprod Biomed Online 17, 436-444 (2008)

hESC line:	**TW4**
special features:	derived by whole embryo culture<br>derived in serum free medium
published in a peer-reviewed journal:	yes
characterization data reported in a peer-reviewed journal:	yes
reference:	Cheng, E. H. et al. Reprod Biomed Online 17, 436-444 (2008)

hESC line:	**TW5**
special features:	derived by whole embryo culture<br>derived in serum free medium

published in a peer-reviewed journal:	yes
characterization data reported in a peer-reviewed journal:	yes
reference:	Cheng, E. H. et al. Reprod Biomed Online 17, 436-444 (2008)

Provider: Kaohsiung Medical University, Taiwan

hESC line:	**T1**
special features:	derived in serum free medium
published in a peer-reviewed journal:	yes
characterization data reported in a peer-reviewed journal:	yes
reference:	Li, S. S. et al. Stem Cells Dev 15, 532-555 (2006)

hESC line:	**T2**
special features:	abnormal karyotype<br>derived in serum free medium
published in a peer-reviewed journal:	yes
characterization data reported in a peer-reviewed journal:	yes
reference:	Li, S. S. et al. Stem Cells Dev 15, 532-555 (2006)

hESC line:	**T3**
special features:	derived in serum free medium
published in a peer-reviewed journal:	yes
characterization data reported in a peer-reviewed journal:	yes
reference:	Li, S. S. et al. Stem Cells Dev 15, 532-555 (2006)

hESC line:	**T4**
special features:	derived in serum free medium
published in a peer-reviewed journal:	yes
characterization data reported in a peer-reviewed journal:	yes
reference:	Li, S. S. et al. Stem Cells Dev 15, 532-555 (2006)

hESC line:	**T5**
special features:	derived in serum free medium

published in a peer-reviewed journal:	yes
characterization data reported in a peer-reviewed journal:	yes
reference:	Li, S. S. et al. Stem Cells Dev 15, 532-555 (2006)

Provider: Taiwan National University

hESC line:	**NTU1**
special features:	
published in a peer-reviewed journal:	yes
characterization data reported in a peer-reviewed journal:	yes
reference:	Chen, H. F. et al. Hum Reprod 22, 567-577(2007)

hESC line:	**NTU2**
special features:	
published in a peer-reviewed journal:	yes
characterization data reported in a peer-reviewed journal:	yes
reference:	Chen, H. F. et al. Hum Reprod 22, 567-577(2007)

hESC line:	**NTU3**
special features:	
published in a peer-reviewed journal:	yes
characterization data reported in a peer-reviewed journal:	yes
reference:	Chen, H. F. et al. Hum Reprod 22, 567-577(2007)

THAILAND

Provider: Chulalonkorn University, Bangkok, Thailand

hESC line:	**n.n.**
special features:	derived in serum free medium<br>derived on human feeder cells<br>mechanical ICM isolation
published in a peer-reviewed journal:	yes
characterization data reported in a peer-reviewed journal:	no
reference:	Pruksananonda, K. et al. J Med Assoc Thai 92, 443-450 (2009)

TURKEY

Provider: Istanbul Memorial Hospital	
hESC line:	**MINE**
special features:	
published in a peer-reviewed journal:	yes
characterization data reported in a peer-reviewed journal:	yes
reference:	Findikli, N. et al. Reprod Biomed Online 10, p. 617-627 (2005)
hESC line:	**NS-10**
special features:	
published in a peer-reviewed journal:	no
characterization data reported in a peer-reviewed journal:	no
reference:	European Union hESC registry (http://www.hescreg.eu/)
hESC line:	**NS-3**
special features:	
published in a peer-reviewed journal:	yes
characterization data reported in a peer-reviewed journal:	yes
reference:	Findikli, N. et al. Reprod Biomed Online 10, p. 617-627 (2005)
hESC line:	**NS-4**
special features:	
published in a peer-reviewed journal:	yes
characterization data reported in a peer-reviewed journal:	yes
reference:	Findikli, N. et al. Reprod Biomed Online 10, p. 617-627 (2005)
hESC line:	**NS-5**
special features:	
published in a peer-reviewed journal:	yes
characterization data reported in a peer-reviewed journal:	yes
reference:	Findikli, N. et al. Reprod Biomed Online 10, p. 617-627 (2005)

hESC line:	**NS-6**
special features:	
published in a peer-reviewed journal:	yes
characterization data reported in a peer-reviewed journal:	yes
reference:	Findikli, N. et al. Reprod Biomed Online 10, p. 617-627 (2005)

---

hESC line:	**NS-7**
special features:	
published in a peer-reviewed journal:	yes
characterization data reported in a peer-reviewed journal:	yes
reference:	Findikli, N. et al. Reprod Biomed Online 10, p. 617-627 (2005)

---

hESC line:	**NS-8**
special features:	
published in a peer-reviewed journal:	yes
characterization data reported in a peer-reviewed journal:	yes
reference:	Findikli, N. et al. Reprod Biomed Online 10, p. 617-627 (2005)

---

hESC line:	**NS-9**
special features:	
published in a peer-reviewed journal:	no
characterization data reported in a peer-reviewed journal:	no
reference:	European Union hESC registry (http://www.hescreg.eu/)

---

hESC line:	**OZ**
special features:	
published in a peer-reviewed journal:	no
characterization data reported in a peer-reviewed journal:	no
reference:	European Union hESC registry (http://www.hescreg.eu/)

---

hESC line:	**OZ-1**
special features:	

published in a peer-reviewed journal:	no
characterization data reported in a peer-reviewed journal:	no
reference:	European Union hESC registry (http://www.hescreg.eu/)

hESC line:	**OZ-2**
special features:	
published in a peer-reviewed journal:	no
characterization data reported in a peer-reviewed journal:	no
reference:	European Union hESC registry (http://www.hescreg.eu/)

hESC line:	**OZ-3**
special features:	
published in a peer-reviewed journal:	no
characterization data reported in a peer-reviewed journal:	no
reference:	European Union hESC registry (http://www.hescreg.eu/)

hESC line:	**OZ-4**
special features:	
published in a peer-reviewed journal:	no
characterization data reported in a peer-reviewed journal:	no
reference:	European Union hESC registry (http://www.hescreg.eu/)

hESC line:	**OZ-5**
special features:	
published in a peer-reviewed journal:	no
characterization data reported in a peer-reviewed journal:	no
reference:	European Union hESC registry (http://www.hescreg.eu/)

hESC line:	**OZ-6**
special features:	

published in a peer-reviewed journal:	no
characterization data reported in a peer-reviewed journal:	no
reference:	European Union hESC registry (http://www.hescreg.eu/)

hESC line:	**OZ-7**
special features:	
published in a peer-reviewed journal:	no
characterization data reported in a peer-reviewed journal:	no
reference:	European Union hESC registry (http://www.hescreg.eu/)

hESC line:	**OZ-8**
special features:	genetic disorder: beta-Thalassaemia (affected)
published in a peer-reviewed journal:	no
characterization data reported in a peer-reviewed journal:	no
reference:	European Union hESC registry (http://www.hescreg.eu/)

UNITED KINGDOM

Provider: King's College London

hESC line:	**KCL-001 (formerly WT3)**
special features:	genetic disorder: not specified
published in a peer-reviewed journal:	yes
characterization data reported in a peer-reviewed journal:	yes
reference:	Pickering, S. J. et al. Reprod Biomed Online 7, 353-364 (2003)

hESC line:	**KCL-002 (formerly WT4)**
special features:	
published in a peer-reviewed journal:	yes
characterization data reported in a peer-reviewed journal:	no*
reference:	UK Stem Cell Bank (http://www.ukstemcellbank.org.uk/catalogue.html)

hESC line:	**KCL-003-CF1 (formerly CF-1)**
special features:	genetic disorder: Cystic fibrosis (CF)

published in a peer-reviewed journal:	yes
characterization data reported in a peer-reviewed journal:	yes
reference:	Pickering, S. J. et al. Reprod Biomed Online 10, 390-397 (2005)

hESC line:	**KCL-005 (formerly HD-1)**
special features:	genetic disorder: Huntington's desease (HD)
published in a peer-reviewed journal:	no
characterization data reported in a peer-reviewed journal:	no*
reference:	UK Stem Cell Bank (http://www.ukstemcellbank.org.uk/catalogue.html)

hESC line:	**KCL-008-HD2 (HD2)**
special features:	
published in a peer-reviewed journal:	no
characterization data reported in a peer-reviewed journal:	no*
reference:	UK Stem Cell Bank (http://www.ukstemcellbank.org.uk/catalogue.html)

hESC line:	**KCL-009-trans-1**
special features:	
published in a peer-reviewed journal:	no
characterization data reported in a peer-reviewed journal:	no*
reference:	UK Stem Cell Bank (http://www.ukstemcellbank.org.uk/catalogue.html)

Provider: Roslin Cells Ltd

hESC line:	**RC2**
special features:	
published in a peer-reviewed journal:	no
characterization data reported in a peer-reviewed journal:	no*
reference:	UK Stem Cell Bank (http://www.ukstemcellbank.org.uk/catalogue.html)

hESC line:	**RC3**
special features:	

published in a peer-reviewed journal:	no
characterization data reported in a peer-reviewed journal:	no*
reference:	UK Stem Cell Bank (http://www.ukstemcellbank.org.uk/catalogue.html)

hESC line:	**RC4**
special features:	
published in a peer-reviewed journal:	no
characterization data reported in a peer-reviewed journal:	no*
reference:	UK Stem Cell Bank (http://www.ukstemcellbank.org.uk/catalogue.html)

hESC line:	**RC5**
special features:	
published in a peer-reviewed journal:	no
characterization data reported in a peer-reviewed journal:	no*
reference:	UK Stem Cell Bank (http://www.ukstemcellbank.org.uk/catalogue.html)

Provider: Roslin Institute

hESC line:	**RCM 1**
special features:	derived by whole embryo culture<br>derived on human feeder cells
published in a peer-reviewed journal:	no
characterization data reported in a peer-reviewed journal:	no*
reference:	De Sousa, P. A. et al. Stem Cell Res, published online ahead of print February 7th, 2009

hESC line:	**RH1**
special features:	derived by whole embryo culture<br>derived in serum free medium<br>derived without feeder cells
published in a peer-reviewed journal:	yes
characterization data reported in a peer-reviewed journal:	yes
reference:	Fletcher, J.M. et al. Cloning Stem Cells 8, 319-334 (2006)

hESC line:	**RH3**
special features:	derived by whole embryo culture<br>xeno-free derivation
published in a peer-reviewed journal:	yes
characterization data reported in a peer-reviewed journal:	yes
reference:	Fletcher, J.M. et al. Cloning Stem Cells 8, 319-334 (2006)

hESC line:	**RH4**
special features:	derived by whole embryo culture<br>derived in serum free medium<br>derived on human feeder cells
published in a peer-reviewed journal:	yes
characterization data reported in a peer-reviewed journal:	yes
reference:	Fletcher, J.M. et al. Cloning Stem Cells 8, 319-334 (2006)

hESC line:	**RH5**
special features:	derived by whole embryo culture<br>derived in serum free medium<br>derived on human feeder cells
published in a peer-reviewed journal:	yes
characterization data reported in a peer-reviewed journal:	yes
reference:	Fletcher, J.M. et al. Cloning Stem Cells 8, 319-334 (2006)

hESC line:	**RH6**
special features:	derived by whole embryo culture<br>derived in serum free medium<br>derived on human feeder cells
published in a peer-reviewed journal:	yes
characterization data reported in a peer-reviewed journal:	yes
reference:	Fletcher, J.M. et al. Cloning Stem Cells 8, 319-334 (2006)

hESC line:	**RH7**
special features:	derived by whole embryo culture<br>derived in serum free medium<br>derived on human feeder cells

published in a peer-reviewed journal:	yes
characterization data reported in a peer-reviewed journal:	yes
reference:	Fletcher, J.M. et al. Cloning Stem Cells 8, 319-334 (2006)

Provider: University of Edinburgh & University of Oxford

hESC line:	**Edi-1**
special features:	
published in a peer-reviewed journal:	no
characterization data reported in a peer-reviewed journal:	no*
reference:	UK Stem Cell Bank (http://www.ukstemcellbank.org.uk/catalogue.html)

hESC line:	**Edi-2**
special features:	
published in a peer-reviewed journal:	no
characterization data reported in a peer-reviewed journal:	no*
reference:	UK Stem Cell Bank (http://www.ukstemcellbank.org.uk/catalogue.html)

hESC line:	**Edi-3**
special features:	
published in a peer-reviewed journal:	no
characterization data reported in a peer-reviewed journal:	no*
reference:	UK Stem Cell Bank (http://www.ukstemcellbank.org.uk/catalogue.html)

hESC line:	**Edi-4**
special features:	
published in a peer-reviewed journal:	no
characterization data reported in a peer-reviewed journal:	no*
reference:	UK Stem Cell Bank (http://www.ukstemcellbank.org.uk/catalogue.html)

Provider: University of Manchester

hESC line:	**Man-1**
special features:	derived in serum free medium<br>mechanical ICM isolation

published in a peer-reviewed journal:	yes
characterization data reported in a peer-reviewed journal:	no*
reference:	UK Stem Cell Bank (http://www.ukstemcellbank.org.uk/catalogue.html)

hESC line:	**Man-2**
special features:	
published in a peer-reviewed journal:	no
characterization data reported in a peer-reviewed journal:	no*
reference:	European Union hESC registry (http://www.hescreg.eu/)

Provider: University of Newcastle

hESC line:	**NCL2**
special features:	derived by whole embryo culture<br>derived in serum free medium
published in a peer-reviewed journal:	yes
characterization data reported in a peer-reviewed journal:	yes
reference:	Zhang, X. et al., Stem Cells 24, 2669-2976 (2006)

hESC line:	**NCL3**
special features:	derived in serum free medium
published in a peer-reviewed journal:	yes
characterization data reported in a peer-reviewed journal:	yes
reference:	Zhang, X. et al., Stem Cells 24, 2669-2976 (2006)

hESC line:	**NCL4**
special features:	derived in serum free medium
published in a peer-reviewed journal:	yes
characterization data reported in a peer-reviewed journal:	yes
reference:	Zhang, X. et al., Stem Cells 24, 2669-2976 (2006)

hESC line:	**NCL5**
special features:	derived in serum free medium

published in a peer-reviewed journal:	yes
characterization data reported in a peer-reviewed journal:	yes
reference:	Zhang, X. et al., Stem Cells 24, 2669-2976 (2006)

hESC line:	**NCL6**
special features:	derived by whole embryo culture<br>derived in serum free medium<br>derived on human feeder cells
published in a peer-reviewed journal:	yes
characterization data reported in a peer-reviewed journal:	yes
reference:	Zhang, X. et al., Stem Cells 24, 2669-2976 (2006)

hESC line:	**NCL7**
special features:	derived by whole embryo culture<br>derived in serum free medium
published in a peer-reviewed journal:	yes
characterization data reported in a peer-reviewed journal:	yes
reference:	Zhang, X. et al., Stem Cells 24, 2669-2976 (2006)

hESC line:	**NCL8**
special features:	derived by whole embryo culture<br>derived in serum free medium<br>derived on human feeder cells
published in a peer-reviewed journal:	yes
characterization data reported in a peer-reviewed journal:	yes
reference:	Zhang, X. et al., Stem Cells 24, 2669-2976 (2006)

hESC line:	**NCL9**
special features:	derived by whole embryo culture<br>derived in serum free medium
published in a peer-reviewed journal:	yes
characterization data reported in a peer-reviewed journal:	yes
reference:	Zhang, X. et al., Stem Cells 24, 2669-2976 (2006)

hESC line:	**NCL1 (formerly hES-NCL-1)**
special features:	
published in a peer-reviewed journal:	yes
characterization data reported in a peer-reviewed journal:	yes
reference:	Stojkovic, M. et al. Stem Cells 22, 790-797 (2004)

Provider: University of Nottingham

hESC line:	**NOTT1**
special features:	derived by whole embryo culture
published in a peer-reviewed journal:	yes
characterization data reported in a peer-reviewed journal:	yes
reference:	Burridge, P. W. et al. Stem Cells 25, 929-938 (2007)

hESC line:	**NOTT2**
special features:	derived by whole embryo culture
published in a peer-reviewed journal:	yes
characterization data reported in a peer-reviewed journal:	yes
reference:	Burridge, P. W. et al. Stem Cells 25, 929-938 (2007)

Provider: University of Sheffield & Axordia Ltd.

hESC line:	**Shef-1**
special features:	derived in serum free medium
published in a peer-reviewed journal:	yes
characterization data reported in a peer-reviewed journal:	yes
reference:	Press Release, Centre for Stem Cell Biology (CSCB), University of Sheffield, 2009/09/29 (http://www.cscb.shef.ac.uk/News/Article239.htm)<br>Draper, J. S. et al. Stem Cells Dev 13, 325-336 (2004)

hESC line:	**Shef-2**
special features:	

published in a peer-reviewed journal:	yes
characterization data reported in a peer-reviewed journal:	yes
reference:	Press Release, Centre for Stem Cell Biology (CSCB), University of Sheffield, 2009/09/29 (http://www.cscb.shef.ac.uk/News/Article239.htm)

---

hESC line:	**Shef-3**
special features:	
published in a peer-reviewed journal:	yes
characterization data reported in a peer-reviewed journal:	yes
reference:	Press Release, Centre for Stem Cell Biology (CSCB), University of Sheffield, 2009/09/29 (http://www.cscb.shef.ac.uk/News/Article239.htm)

---

hESC line:	**Shef-4**
special features:	
published in a peer-reviewed journal:	yes
characterization data reported in a peer-reviewed journal:	no*
reference:	Inniss, K. & Moore, H., Stem Cells Dev 15, 789-796 (2006)

---

hESC line:	**Shef-5**
special features:	
published in a peer-reviewed journal:	yes
characterization data reported in a peer-reviewed journal:	no*
reference:	Inniss, K. & Moore, H., Stem Cells Dev 15, 789-796 (2006)

---

hESC line:	**Shef-6**
special features:	
published in a peer-reviewed journal:	yes
characterization data reported in a peer-reviewed journal:	no*
reference:	Inniss, K. & Moore, H., Stem Cells Dev 15, 789-796 (2006)

---

hESC line:	**Shef-7**
special features:	

published in a peer-reviewed journal:	yes
characterization data reported in a peer-reviewed journal:	no*
reference:	UK Stem Cell Bank (http://www.ukstemcellbank.org.uk/catalogue.html)

hESC line:	**Shef-8**
special features:	
published in a peer-reviewed journal:	no
characterization data reported in a peer-reviewed journal:	no*
reference:	UK Stem Cell Bank (http://www.ukstemcellbank.org.uk/catalogue.html)

UNITED STATES

Provider: Advanced Cell Technology, Worcester, MA, USA

hESC line:	**MA01**
special features:	
published in a peer-reviewed journal:	yes
characterization data reported in a peer-reviewed journal:	no
reference:	Lund, R.D. et al. Cloning Stem Cells 8, 189-199 (2006)

hESC line:	**MA03**
special features:	
published in a peer-reviewed journal:	yes
characterization data reported in a peer-reviewed journal:	no
reference:	Lund, R.D. et al. Cloning Stem Cells 8, 189-199 (2006)

hESC line:	**MA04**
special features:	
published in a peer-reviewed journal:	yes
characterization data reported in a peer-reviewed journal:	no
reference:	Lund, R.D. et al. Cloning Stem Cells 8, 189-199 (2006)

hESC line:	**MA09**
special features:	

published in a peer-reviewed journal:	yes
characterization data reported in a peer-reviewed journal:	no
reference:	Lund, R.D. et al. Cloning Stem Cells 8, 189-199 (2006)

hESC line:	**MA133**
special features:	
published in a peer-reviewed journal:	yes
characterization data reported in a peer-reviewed journal:	no
reference:	UMA-Stem Cell Registry (http://www.umassmed.edu/iscr/index.aspx)

hESC line:	**MA135**
special features:	
published in a peer-reviewed journal:	no
characterization data reported in a peer-reviewed journal:	no
reference:	UMA-Stem Cell Registry (http://www.umassmed.edu/iscr/index.aspx)

hESC line:	**MA136**
special features:	
published in a peer-reviewed journal:	no
characterization data reported in a peer-reviewed journal:	no
reference:	UMA-Stem Cell Registry (http://www.umassmed.edu/iscr/index.aspx)

hESC line:	**MA137**
special features:	
published in a peer-reviewed journal:	no
characterization data reported in a peer-reviewed journal:	no
reference:	UMA-Stem Cell Registry (http://www.umassmed.edu/iscr/index.aspx)

hESC line:	**MA138**
special features:	

published in a peer-reviewed journal:	no
characterization data reported in a peer-reviewed journal:	no
reference:	UMA-Stem Cell Registry (http://www.umassmed.edu/iscr/index.aspx)

hESC line:	**MA139**
special features:	
published in a peer-reviewed journal:	no
characterization data reported in a peer-reviewed journal:	no
reference:	UMA-Stem Cell Registry (http://www.umassmed.edu/iscr/index.aspx)

hESC line:	**MA14 (formerly ACT-14)**
special features:	derived without feeder cells
published in a peer-reviewed journal:	yes
characterization data reported in a peer-reviewed journal:	yes
reference:	Klimanskaya, I. et al. Lancet 365, 1636-1641(2005)

hESC line:	**MA140**
special features:	
published in a peer-reviewed journal:	no
characterization data reported in a peer-reviewed journal:	no
reference:	UMA-Stem Cell Registry (http://www.umassmed.edu/iscr/index.aspx)

hESC line:	**MA141**
special features:	
published in a peer-reviewed journal:	no
characterization data reported in a peer-reviewed journal:	no
reference:	UMA-Stem Cell Registry (http://www.umassmed.edu/iscr/index.aspx)

hESC line:	**MA40**
special features:	

published in a peer-reviewed journal:	yes
characterization data reported in a peer-reviewed journal:	no
reference:	Lund, R.D. et al. Cloning Stem Cells 8, 189-199 (2006)

hESC line:	**MA42**
special features:	
published in a peer-reviewed journal:	no
characterization data reported in a peer-reviewed journal:	no
reference:	UMA-Stem Cell Registry (http://www.umassmed.edu/iscr/index.aspx)

hESC line:	**MA50**
special features:	
published in a peer-reviewed journal:	no
characterization data reported in a peer-reviewed journal:	no
reference:	UMA-Stem Cell Registry (http://www.umassmed.edu/iscr/index.aspx)

hESC line:	**MA99**
special features:	
published in a peer-reviewed journal:	yes
characterization data reported in a peer-reviewed journal:	no
reference:	UMA-Stem Cell Registry (http://www.umassmed.edu/iscr/index.aspx)

hESC line:	**MAJ1**
special features:	
published in a peer-reviewed journal:	yes
characterization data reported in a peer-reviewed journal:	no
reference:	Lund, R.D. et al. Cloning Stem Cells 8, 189-199 (2006)

hESC line:	**NED1 (MA126)**
special features:	derived from single blastomere

published in a peer-reviewed journal:	yes
characterization data reported in a peer-reviewed journal:	yes
reference:	Chung, Y. et al. Cell Stem Cell 2, 113-117 (2008)

hESC line:	**NED2 (MA127)**
special features:	derived from single blastomere
published in a peer-reviewed journal:	yes
characterization data reported in a peer-reviewed journal:	yes
reference:	Chung, Y. et al. Cell Stem Cell 2, 113-117 (2008)

hESC line:	**NED3 (MA128)**
special features:	derived from single blastomere
published in a peer-reviewed journal:	yes
characterization data reported in a peer-reviewed journal:	yes
reference:	Chung, Y. et al. Cell Stem Cell 2, 113-117 (2008)

hESC line:	**NED4 (MA129)**
special features:	derived from single blastomere
published in a peer-reviewed journal:	yes
characterization data reported in a peer-reviewed journal:	yes
reference:	Chung, Y. et al. Cell Stem Cell 2, 113-117 (2008)

hESC line:	**NED5**
special features:	derived from single blastomere
published in a peer-reviewed journal:	yes
characterization data reported in a peer-reviewed journal:	yes
reference:	Chung, Y. et al. Cell Stem Cell 2, 113-117 (2008)

Provider: BresaGen, Inc., Athens, Georgia

hESC line:	**BG01**
special features:	

published in a peer-reviewed journal:	yes
characterization data reported in a peer-reviewed journal:	yes
reference:	Mitalipova, M. et al. Stem Cells 21, 521-526 (2003)<br>NIH Human Embryonic Stem Cell Registry (http://grants.nih.gov/stem_cells/registry/current.htm)

hESC line:	**BG02**
special features:	
published in a peer-reviewed journal:	yes
characterization data reported in a peer-reviewed journal:	yes
reference:	Mitalipova, M. et al. Stem Cells 21, 521-526 (2003)<br>NIH Human Embryonic Stem Cell Registry (http://grants.nih.gov/stem_cells/registry/current.htm)

hESC line:	**BG03**
special features:	
published in a peer-reviewed journal:	yes
characterization data reported in a peer-reviewed journal:	yes
reference:	Mitalipova, M. et al. Stem Cells 21, 521-526 (2003)<br>NIH Human Embryonic Stem Cell Registry (http://grants.nih.gov/stem_cells/registry/current.htm)

hESC line:	**BG04**
special features:	
published in a peer-reviewed journal:	yes
characterization data reported in a peer-reviewed journal:	yes
reference:	Mitalipova, M. et al. Stem Cells 21, 521-526 (2003)<br>NIH Human Embryonic Stem Cell Registry (http://grants.nih.gov/stem_cells/registry/current.htm)

Provider: Children's Hospital Coorperation, Boston, MA

hESC line:	**CHB-1**
special features:	derived by whole embryo culture<br>derived in serum free medium

published in a peer-reviewed journal:	yes
characterization data reported in a peer-reviewed journal:	yes
reference:	Lerou, P. H. et al. Nat Protoc 3, 923-933 (2008)

hESC line:	**CHB-10**
special features:	derived by whole embryo culture<br>derived in serum free medium
published in a peer-reviewed journal:	yes
characterization data reported in a peer-reviewed journal:	yes
reference:	Lerou, P. H. et al. Nat Protoc 3, 923-933 (2008)

hESC line:	**CHB-11**
special features:	derived by whole embryo culture<br>derived in serum free medium
published in a peer-reviewed journal:	yes
characterization data reported in a peer-reviewed journal:	yes
reference:	Lerou, P. H. et al. Nat Protoc 3, 923-933 (2008)

hESC line:	**CHB-12**
special features:	derived by whole embryo culture<br>derived in serum free medium
published in a peer-reviewed journal:	yes
characterization data reported in a peer-reviewed journal:	yes
reference:	Lerou, P. H. et al. Nat Protoc 3, 923-933 (2008)

hESC line:	**CHB-13**
special features:	
published in a peer-reviewed journal:	no
characterization data reported in a peer-reviewed journal:	no
reference:	NIH Human Embryonic Stem Cell Registry (http://grants.nih.gov/stem_cells/registry/current.htm)

hESC line:	**CHB-14**
special features:	

published in a peer-reviewed journal:	no
characterization data reported in a peer-reviewed journal:	no
reference:	NIH Human Embryonic Stem Cell Registry (http://grants.nih.gov/stem_cells/registry/current.htm)

hESC line:	**CHB-15**
special features:	
published in a peer-reviewed journal:	no
characterization data reported in a peer-reviewed journal:	no
reference:	NIH Human Embryonic Stem Cell Registry (http://grants.nih.gov/stem_cells/registry/current.htm)

hESC line:	**CHB-2**
special features:	derived by whole embryo culture<br>derived in serum free medium
published in a peer-reviewed journal:	yes
characterization data reported in a peer-reviewed journal:	yes
reference:	Lerou, P. H. et al. Nat Protoc 3, 923-933 (2008)

hESC line:	**CHB-3**
special features:	derived by whole embryo culture<br>derived in serum free medium
published in a peer-reviewed journal:	yes
characterization data reported in a peer-reviewed journal:	yes
reference:	Lerou, P. H. et al. Nat Protoc 3, 923-933 (2008)

hESC line:	**CHB-4**
special features:	derived by whole embryo culture<br>derived in serum free medium
published in a peer-reviewed journal:	yes
characterization data reported in a peer-reviewed journal:	yes
reference:	Lerou, P. H. et al. Nat Protoc 3, 923-933 (2008)

hESC line:	**CHB-5**
special features:	derived by whole embryo culture<br>derived in serum free medium

published in a peer-reviewed journal:	yes
characterization data reported in a peer-reviewed journal:	yes
reference:	Lerou, P. H. et al. Nat Protoc 3, 923-933 (2008)

hESC line:	**CHB-6**
special features:	derived by whole embryo culture<br>derived in serum free medium
published in a peer-reviewed journal:	yes
characterization data reported in a peer-reviewed journal:	yes
reference:	Lerou, P. H. et al. Nat Protoc 3, 923-933 (2008)

hESC line:	**CHB-8**
special features:	derived by whole embryo culture<br>derived in serum free medium
published in a peer-reviewed journal:	yes
characterization data reported in a peer-reviewed journal:	yes
reference:	Lerou, P. H. et al. Nat Protoc 3, 923-933 (2008)

hESC line:	**CHB-9**
special features:	derived by whole embryo culture<br>derived in serum free medium
published in a peer-reviewed journal:	yes
characterization data reported in a peer-reviewed journal:	yes
reference:	Lerou, P. H. et al. Nat Protoc 3, 923-933 (2008)

Provider: Children's Memorial Hospital, Chicago, Illinois

hESC line:	**CM-1**
special features:	
published in a peer-reviewed journal:	no
characterization data reported in a peer-reviewed journal:	no
reference:	NIH Human Embryonic Stem Cell Registry (http://grants.nih.gov/stem_cells/registry/current.htm)

hESC line:	**CM-11**
special features:	

published in a peer-reviewed journal:	no
characterization data reported in a peer-reviewed journal:	no
reference:	NIH Human Embryonic Stem Cell Registry (http://grants.nih.gov/stem_cells/registry/current.htm)

hESC line:	**CM-12**
special features:	
published in a peer-reviewed journal:	no
characterization data reported in a peer-reviewed journal:	no
reference:	NIH Human Embryonic Stem Cell Registry (http://grants.nih.gov/stem_cells/registry/current.htm)

hESC line:	**CM-13**
special features:	
published in a peer-reviewed journal:	no
characterization data reported in a peer-reviewed journal:	no
reference:	NIH Human Embryonic Stem Cell Registry (http://grants.nih.gov/stem_cells/registry/current.htm)

hESC line:	**CM-14**
special features:	
published in a peer-reviewed journal:	no
characterization data reported in a peer-reviewed journal:	no
reference:	NIH Human Embryonic Stem Cell Registry (http://grants.nih.gov/stem_cells/registry/current.htm)

hESC line:	**CM-16**
special features:	
published in a peer-reviewed journal:	no
characterization data reported in a peer-reviewed journal:	no
reference:	NIH Human Embryonic Stem Cell Registry (http://grants.nih.gov/stem_cells/registry/current.htm)

hESC line:	**CM-2**
special features:	

published in a peer-reviewed journal:	no
characterization data reported in a peer-reviewed journal:	no
reference:	NIH Human Embryonic Stem Cell Registry (http://grants.nih.gov/stem_cells/registry/current.htm)

hESC line:	**CM-5**
special features:	
published in a peer-reviewed journal:	no
characterization data reported in a peer-reviewed journal:	no
reference:	NIH Human Embryonic Stem Cell Registry (http://grants.nih.gov/stem_cells/registry/current.htm)

hESC line:	**CM-6**
special features:	
published in a peer-reviewed journal:	no
characterization data reported in a peer-reviewed journal:	no
reference:	NIH Human Embryonic Stem Cell Registry (http://grants.nih.gov/stem_cells/registry/current.htm)

hESC line:	**CM-7**
special features:	
published in a peer-reviewed journal:	no
characterization data reported in a peer-reviewed journal:	no
reference:	NIH Human Embryonic Stem Cell Registry (http://grants.nih.gov/stem_cells/registry/current.htm)

hESC line:	**CM-8**
special features:	
published in a peer-reviewed journal:	no
characterization data reported in a peer-reviewed journal:	no
reference:	NIH Human Embryonic Stem Cell Registry (http://grants.nih.gov/stem_cells/registry/current.htm)

Provider: Cythera Inc., San Diego California

hESC line:	**CyT25**
special features:	

published in a peer-reviewed journal:	yes
characterization data reported in a peer-reviewed journal:	no
reference:	D'Amour, K. A. et al. Nat Biotechnol 23, 1534-1541 (2005)<br>Hoffman, L. M. et al. Stem Cells 23 1468-1478 (2005)

hESC line:	**hes-101**
special features:	
published in a peer-reviewed journal:	no
characterization data reported in a peer-reviewed journal:	no
reference:	NIH Human Embryonic Stem Cell Registry (http://grants.nih.gov/stem_cells/registry/current.htm)

hESC line:	**hes-1-2**
special features:	
published in a peer-reviewed journal:	no
characterization data reported in a peer-reviewed journal:	no
reference:	NIH Human Embryonic Stem Cell Registry (http://grants.nih.gov/stem_cells/registry/current.htm)

hESC line:	**hes-3-0**
special features:	
published in a peer-reviewed journal:	no
characterization data reported in a peer-reviewed journal:	no
reference:	NIH Human Embryonic Stem Cell Registry (http://grants.nih.gov/stem_cells/registry/current.htm)

hESC line:	**hes-4-0**
special features:	
published in a peer-reviewed journal:	no
characterization data reported in a peer-reviewed journal:	no
reference:	NIH Human Embryonic Stem Cell Registry (http://grants.nih.gov/stem_cells/registry/current.htm)

hESC line:	**hes-5-1**
special features:	

published in a peer-reviewed journal:	no
characterization data reported in a peer-reviewed journal:	no
reference:	NIH Human Embryonic Stem Cell Registry (http://grants.nih.gov/stem_cells/registry/current.htm)

hESC line:	**hes-8-1**
special features:	
published in a peer-reviewed journal:	no
characterization data reported in a peer-reviewed journal:	no
reference:	NIH Human Embryonic Stem Cell Registry (http://grants.nih.gov/stem_cells/registry/current.htm)

hESC line:	**hes-8-2**
special features:	
published in a peer-reviewed journal:	no
characterization data reported in a peer-reviewed journal:	no
reference:	NIH Human Embryonic Stem Cell Registry (http://grants.nih.gov/stem_cells/registry/current.htm)

hESC line:	**hes-9-1**
special features:	
published in a peer-reviewed journal:	no
characterization data reported in a peer-reviewed journal:	no
reference:	NIH Human Embryonic Stem Cell Registry (http://grants.nih.gov/stem_cells/registry/current.htm)

hESC line:	**hes-9-2**
special features:	
published in a peer-reviewed journal:	no
characterization data reported in a peer-reviewed journal:	no
reference:	NIH Human Embryonic Stem Cell Registry (http://grants.nih.gov/stem_cells/registry/current.htm)

Provider: Harvard University

hESC line:	**HUES PGD 1**
special features:	

published in a peer-reviewed journal:	no
characterization data reported in a peer-reviewed journal:	no
reference:	NIH Human Embryonic Stem Cell Registry (http://grants.nih.gov/stem_cells/registry/current.htm)

hESC line:	**HUES PGD 10**
special features:	
published in a peer-reviewed journal:	no
characterization data reported in a peer-reviewed journal:	no
reference:	NIH Human Embryonic Stem Cell Registry (http://grants.nih.gov/stem_cells/registry/current.htm)

hESC line:	**HUES PGD 11**
special features:	
published in a peer-reviewed journal:	no
characterization data reported in a peer-reviewed journal:	no
reference:	NIH Human Embryonic Stem Cell Registry (http://grants.nih.gov/stem_cells/registry/current.htm)

hESC line:	**HUES PGD 12**
special features:	
published in a peer-reviewed journal:	no
characterization data reported in a peer-reviewed journal:	no
reference:	NIH Human Embryonic Stem Cell Registry (http://grants.nih.gov/stem_cells/registry/current.htm)

hESC line:	**HUES PGD 2**
special features:	
published in a peer-reviewed journal:	no
characterization data reported in a peer-reviewed journal:	no
reference:	NIH Human Embryonic Stem Cell Registry (http://grants.nih.gov/stem_cells/registry/current.htm)

hESC line:	**HUES PGD 3**
special features:	

published in a peer-reviewed journal:	no
characterization data reported in a peer-reviewed journal:	no
reference:	NIH Human Embryonic Stem Cell Registry (http://grants.nih.gov/stem_cells/registry/current.htm)

hESC line:	**HUES PGD 4**
special features:	
published in a peer-reviewed journal:	no
characterization data reported in a peer-reviewed journal:	no
reference:	

hESC line:	**HUES PGD 5**
special features:	
published in a peer-reviewed journal:	no
characterization data reported in a peer-reviewed journal:	no
reference:	NIH Human Embryonic Stem Cell Registry (http://grants.nih.gov/stem_cells/registry/current.htm)

hESC line:	**HUES PGD 6**
special features:	
published in a peer-reviewed journal:	no
characterization data reported in a peer-reviewed journal:	no
reference:	NIH Human Embryonic Stem Cell Registry (http://grants.nih.gov/stem_cells/registry/current.htm)

hESC line:	**HUES PGD 7**
special features:	
published in a peer-reviewed journal:	no
characterization data reported in a peer-reviewed journal:	no
reference:	NIH Human Embryonic Stem Cell Registry (http://grants.nih.gov/stem_cells/registry/current.htm)

hESC line:	**HUES PGD 8**
special features:	

published in a peer-reviewed journal:	no
characterization data reported in a peer-reviewed journal:	no
reference:	NIH Human Embryonic Stem Cell Registry (http://grants.nih.gov/stem_cells/registry/current.htm)

hESC line:	**HUES PGD 9**
special features:	
published in a peer-reviewed journal:	no
characterization data reported in a peer-reviewed journal:	no
reference:	NIH Human Embryonic Stem Cell Registry (http://grants.nih.gov/stem_cells/registry/current.htm)

hESC line:	**HUES1**
special features:	derived in serum free medium
published in a peer-reviewed journal:	yes
characterization data reported in a peer-reviewed journal:	yes
reference:	Cowan, C. A. et al. N Engl J Med 350, 1353-1356 (2004)

hESC line:	**HUES10**
special features:	derived in serum free medium
published in a peer-reviewed journal:	yes
characterization data reported in a peer-reviewed journal:	yes
reference:	Cowan, C. A. et al. N Engl J Med 350, 1353-1356 (2004)

hESC line:	**HUES11**
special features:	derived in serum free medium
published in a peer-reviewed journal:	yes
characterization data reported in a peer-reviewed journal:	yes
reference:	Cowan, C. A. et al. N Engl J Med 350, 1353-1356 (2004)

hESC line:	**HUES12**
special features:	derived in serum free medium

published in a peer-reviewed journal:	yes
characterization data reported in a peer-reviewed journal:	yes
reference:	Cowan, C. A. et al. N Engl J Med 350, 1353-1356 (2004)

hESC line:	**HUES13**
special features:	derived in serum free medium
published in a peer-reviewed journal:	yes
characterization data reported in a peer-reviewed journal:	yes
reference:	Cowan, C. A. et al. N Engl J Med 350, 1353-1356 (2004)

hESC line:	**HUES14**
special features:	derived in serum free medium
published in a peer-reviewed journal:	yes
characterization data reported in a peer-reviewed journal:	yes
reference:	Cowan, C. A. et al. N Engl J Med 350, 1353-1356 (2004)

hESC line:	**HUES15**
special features:	derived in serum free medium
published in a peer-reviewed journal:	yes
characterization data reported in a peer-reviewed journal:	yes
reference:	Cowan, C. A. et al. N Engl J Med 350, 1353-1356 (2004)

hESC line:	**HUES16**
special features:	derived in serum free medium
published in a peer-reviewed journal:	yes
characterization data reported in a peer-reviewed journal:	yes
reference:	Cowan, C. A. et al. N Engl J Med 350, 1353-1356 (2004)

hESC line:	**HUES17**
special features:	derived in serum free medium

published in a peer-reviewed journal:	yes
characterization data reported in a peer-reviewed journal:	yes
reference:	Cowan, C. A. et al. N Engl J Med 350, 1353-1356 (2004)

hESC line:	**HUES18**
special features:	derived in serum free medium
published in a peer-reviewed journal:	yes
characterization data reported in a peer-reviewed journal:	yes
reference:	Chen, A. E. et al. Cell Stem Cell 4, 103-106 (2009)

hESC line:	**HUES19**
special features:	derived in serum free medium
published in a peer-reviewed journal:	yes
characterization data reported in a peer-reviewed journal:	yes
reference:	Chen, A. E. et al. Cell Stem Cell 4, 103-106 (2009)

hESC line:	**HUES2**
special features:	derived in serum free medium
published in a peer-reviewed journal:	yes
characterization data reported in a peer-reviewed journal:	yes
reference:	Cowan, C. A. et al. N Engl J Med 350, 1353-1356 (2004)

hESC line:	**HUES20**
special features:	derived in serum free medium
published in a peer-reviewed journal:	yes
characterization data reported in a peer-reviewed journal:	yes
reference:	Chen, A. E. et al. Cell Stem Cell 4, 103-106 (2009)

hESC line:	**HUES21**
special features:	derived in serum free medium

published in a peer-reviewed journal:	yes
characterization data reported in a peer-reviewed journal:	yes
reference:	Chen, A. E. et al. Cell Stem Cell 4, 103-106 (2009)

hESC line:	**HUES22**
special features:	derived in serum free medium
published in a peer-reviewed journal:	yes
characterization data reported in a peer-reviewed journal:	yes
reference:	Chen, A. E. et al. Cell Stem Cell 4, 103-106 (2009)

hESC line:	**HUES23**
special features:	abnormal karyotype<br>derived in serum free medium
published in a peer-reviewed journal:	yes
characterization data reported in a peer-reviewed journal:	yes
reference:	Chen, A. E. et al. Cell Stem Cell 4, 103-106 (2009)

hESC line:	**HUES24**
special features:	derived in serum free medium
published in a peer-reviewed journal:	yes
characterization data reported in a peer-reviewed journal:	yes
reference:	Chen, A. E. et al. Cell Stem Cell 4, 103-106 (2009)

hESC line:	**HUES25**
special features:	derived in serum free medium
published in a peer-reviewed journal:	yes
characterization data reported in a peer-reviewed journal:	yes
reference:	Chen, A. E. et al. Cell Stem Cell 4, 103-106 (2009)

hESC line:	**HUES26**
special features:	derived in serum free medium

published in a peer-reviewed journal:	yes
characterization data reported in a peer-reviewed journal:	yes
reference:	Chen, A. E. et al. Cell Stem Cell 4, 103-106 (2009)

hESC line:	**HUES27**
special features:	derived in serum free medium
published in a peer-reviewed journal:	yes
characterization data reported in a peer-reviewed journal:	yes
reference:	Chen, A. E. et al. Cell Stem Cell 4, 103-106 (2009)

hESC line:	**HUES28**
special features:	derived in serum free medium
published in a peer-reviewed journal:	yes
characterization data reported in a peer-reviewed journal:	yes
reference:	Chen, A. E. et al. Cell Stem Cell 4, 103-106 (2009)

hESC line:	**HUES29**
special features:	abnormal karyotype<br>derived in serum free medium
published in a peer-reviewed journal:	yes
characterization data reported in a peer-reviewed journal:	yes
reference:	Chen, A. E. et al. Cell Stem Cell 4, 103-106 (2009)

hESC line:	**HUES3**
special features:	derived in serum free medium
published in a peer-reviewed journal:	yes
characterization data reported in a peer-reviewed journal:	yes
reference:	Cowan, C. A. et al. N Engl J Med 350, 1353-1356 (2004)

hESC line:	**HUES30**
special features:	derived in serum free medium<br>laser-assisted ICM isolation

published in a peer-reviewed journal:	yes
characterization data reported in a peer-reviewed journal:	yes
reference:	Chen, A. E. et al. Cell Stem Cell 4, 103-106 (2009)

hESC line:	**HUES31**
special features:	derived in serum free medium<br>laser-assisted ICM isolation
published in a peer-reviewed journal:	yes
characterization data reported in a peer-reviewed journal:	yes
reference:	Chen, A. E. et al. Cell Stem Cell 4, 103-106 (2009)

hESC line:	**HUES32**
special features:	derived in serum free medium
published in a peer-reviewed journal:	yes
characterization data reported in a peer-reviewed journal:	yes
reference:	Chen, A. E. et al. Cell Stem Cell 4, 103-106 (2009)

hESC line:	**HUES33**
special features:	derived in serum free medium
published in a peer-reviewed journal:	yes
characterization data reported in a peer-reviewed journal:	yes
reference:	Chen, A. E. et al. Cell Stem Cell 4, 103-106 (2009)

hESC line:	**HUES34**
special features:	derived in serum free medium<br>laser-assisted ICM isolation
published in a peer-reviewed journal:	yes
characterization data reported in a peer-reviewed journal:	yes
reference:	Chen, A. E. et al. Cell Stem Cell 4, 103-106 (2009)

hESC line:	**HUES35**
special features:	derived in serum free medium<br>laser-assisted ICM isolation

published in a peer-reviewed journal:	yes
characterization data reported in a peer-reviewed journal:	yes
reference:	Chen, A. E. et al. Cell Stem Cell 4, 103-106 (2009)

hESC line:	**HUES36**
special features:	derived in serum free medium
published in a peer-reviewed journal:	yes
characterization data reported in a peer-reviewed journal:	yes
reference:	Chen, A. E. et al. Cell Stem Cell 4, 103-106 (2009)

hESC line:	**HUES37**
special features:	derived in serum free medium
published in a peer-reviewed journal:	yes
characterization data reported in a peer-reviewed journal:	yes
reference:	Chen, A. E. et al. Cell Stem Cell 4, 103-106 (2009)

hESC line:	**HUES38**
special features:	derived in serum free medium<br>laser-assisted ICM isolation
published in a peer-reviewed journal:	yes
characterization data reported in a peer-reviewed journal:	yes
reference:	Chen, A. E. et al. Cell Stem Cell 4, 103-106 (2009)

hESC line:	**HUES39**
special features:	derived in serum free medium<br>laser-assisted ICM isolation
published in a peer-reviewed journal:	yes
characterization data reported in a peer-reviewed journal:	yes
reference:	Chen, A. E. et al. Cell Stem Cell 4, 103-106 (2009)

hESC line:	**HUES4**
special features:	derived in serum free medium

published in a peer-reviewed journal:	yes
characterization data reported in a peer-reviewed journal:	yes
reference:	Cowan, C. A. et al. N Engl J Med 350, 1353-1356 (2004)

---

hESC line:	**HUES40**
special features:	derived in serum free medium<br>laser-assisted ICM isolation
published in a peer-reviewed journal:	yes
characterization data reported in a peer-reviewed journal:	yes
reference:	Chen, A. E. et al. Cell Stem Cell 4, 103-106 (2009)

---

hESC line:	**HUES41**
special features:	derived in serum free medium<br>laser-assisted ICM isolation
published in a peer-reviewed journal:	yes
characterization data reported in a peer-reviewed journal:	yes
reference:	Chen, A. E. et al. Cell Stem Cell 4, 103-106 (2009)

---

hESC line:	**HUES42**
special features:	derived in serum free medium<br>laser-assisted ICM isolation
published in a peer-reviewed journal:	yes
characterization data reported in a peer-reviewed journal:	yes
reference:	Chen, A. E. et al. Cell Stem Cell 4, 103-106 (2009)

---

hESC line:	**HUES43**
special features:	derived in serum free medium<br>laser-assisted ICM isolation
published in a peer-reviewed journal:	yes
characterization data reported in a peer-reviewed journal:	yes
reference:	Chen, A. E. et al. Cell Stem Cell 4, 103-106 (2009)

---

hESC line:	**HUES44**
special features:	

published in a peer-reviewed journal:	no
characterization data reported in a peer-reviewed journal:	no
reference:	NIH Human Embryonic Stem Cell Registry (http://grants.nih.gov/stem_cells/registry/current.htm)

hESC line:	**HUES45**
special features:	
published in a peer-reviewed journal:	no
characterization data reported in a peer-reviewed journal:	no
reference:	NIH Human Embryonic Stem Cell Registry (http://grants.nih.gov/stem_cells/registry/current.htm)

hESC line:	**HUES46**
special features:	derived in serum free medium
published in a peer-reviewed journal:	yes
characterization data reported in a peer-reviewed journal:	yes
reference:	Chen, A. E. et al. Cell Stem Cell 4, 103-106 (2009)

hESC line:	**HUES47**
special features:	
published in a peer-reviewed journal:	no
characterization data reported in a peer-reviewed journal:	no
reference:	NIH Human Embryonic Stem Cell Registry (http://grants.nih.gov/stem_cells/registry/current.htm)

hESC line:	**HUES48**
special features:	derived in serum free medium<br>laser-assisted ICM isolation
published in a peer-reviewed journal:	yes
characterization data reported in a peer-reviewed journal:	yes
reference:	Chen, A. E. et al. Cell Stem Cell 4, 103-106 (2009)

hESC line:	**HUES49**
special features:	derived in serum free medium<br>laser-assisted ICM isolation

published in a peer-reviewed journal:	yes
characterization data reported in a peer-reviewed journal:	yes
reference:	Chen, A. E. et al. Cell Stem Cell 4, 103-106 (2009)

hESC line:	**HUES5**
special features:	derived in serum free medium
published in a peer-reviewed journal:	yes
characterization data reported in a peer-reviewed journal:	yes
reference:	Cowan, C. A. et al. N Engl J Med 350, 1353-1356 (2004)

hESC line:	**HUES50**
special features:	abnormal karyotype<br>derived in serum free medium<br>laser-assisted ICM isolation
published in a peer-reviewed journal:	yes
characterization data reported in a peer-reviewed journal:	yes
reference:	Chen, A. E. et al. Cell Stem Cell 4, 103-106 (2009)

hESC line:	**HUES51**
special features:	derived in serum free medium<br>laser-assisted ICM isolation
published in a peer-reviewed journal:	yes
characterization data reported in a peer-reviewed journal:	yes
reference:	Chen, A. E. et al. Cell Stem Cell 4, 103-106 (2009)

hESC line:	**HUES52**
special features:	derived in serum free medium<br>laser-assisted ICM isolation
published in a peer-reviewed journal:	yes
characterization data reported in a peer-reviewed journal:	yes
reference:	Chen, A. E. et al. Cell Stem Cell 4, 103-106 (2009)

hESC line:	**HUES53**
special features:	derived in serum free medium<br>laser-assisted ICM isolation
published in a peer-reviewed journal:	yes
characterization data reported in a peer-reviewed journal:	yes
reference:	Chen, A. E. et al. Cell Stem Cell 4, 103-106 (2009)

hESC line:	**HUES54**
special features:	derived in serum free medium<br>laser-assisted ICM isolation
published in a peer-reviewed journal:	yes
characterization data reported in a peer-reviewed journal:	yes
reference:	Chen, A. E. et al. Cell Stem Cell 4, 103-106 (2009)

hESC line:	**HUES55**
special features:	derived in serum free medium<br>laser-assisted ICM isolation
published in a peer-reviewed journal:	yes
characterization data reported in a peer-reviewed journal:	yes
reference:	Chen, A. E. et al. Cell Stem Cell 4, 103-106 (2009)

hESC line:	**HUES56**
special features:	derived in serum free medium<br>laser-assisted ICM isolation
published in a peer-reviewed journal:	yes
characterization data reported in a peer-reviewed journal:	yes
reference:	Chen, A. E. et al. Cell Stem Cell 4, 103-106 (2009)

hESC line:	**HUES57**
special features:	derived in serum free medium<br>laser-assisted ICM isolation

published in a peer-reviewed journal:	yes
characterization data reported in a peer-reviewed journal:	yes
reference:	Chen, A. E. et al. Cell Stem Cell 4, 103-106 (2009)

hESC line:	**HUES58**
special features:	derived in serum free medium<br>laser-assisted ICM isolation
published in a peer-reviewed journal:	yes
characterization data reported in a peer-reviewed journal:	yes
reference:	Chen, A. E. et al. Cell Stem Cell 4, 103-106 (2009)

hESC line:	**HUES59**
special features:	derived in serum free medium<br>laser-assisted ICM isolation
published in a peer-reviewed journal:	yes
characterization data reported in a peer-reviewed journal:	yes
reference:	Chen, A. E. et al. Cell Stem Cell 4, 103-106 (2009)

hESC line:	**HUES6**
special features:	derived in serum free medium
published in a peer-reviewed journal:	yes
characterization data reported in a peer-reviewed journal:	yes
reference:	Cowan, C. A. et al. N Engl J Med 350, 1353-1356 (2004)

hESC line:	**HUES60**
special features:	derived in serum free medium<br>laser-assisted ICM isolation
published in a peer-reviewed journal:	yes
characterization data reported in a peer-reviewed journal:	yes
reference:	Chen, A. E. et al. Cell Stem Cell 4, 103-106 (2009)

hESC line:	**HUES61**
special features:	derived in serum free medium<br>laser-assisted ICM isolation

published in a peer-reviewed journal:	yes
characterization data reported in a peer-reviewed journal:	yes
reference:	Chen, A. E. et al. Cell Stem Cell 4, 103-106 (2009)

hESC line:	**HUES62**
special features:	derived in serum free medium<br>laser-assisted ICM isolation
published in a peer-reviewed journal:	yes
characterization data reported in a peer-reviewed journal:	yes
reference:	Chen, A. E. et al. Cell Stem Cell 4, 103-106 (2009)

hESC line:	**HUES63**
special features:	derived in serum free medium<br>laser-assisted ICM isolation
published in a peer-reviewed journal:	yes
characterization data reported in a peer-reviewed journal:	yes
reference:	Chen, A. E. et al. Cell Stem Cell 4, 103-106 (2009)

hESC line:	**HUES64**
special features:	derived in serum free medium<br>laser-assisted ICM isolation
published in a peer-reviewed journal:	yes
characterization data reported in a peer-reviewed journal:	yes
reference:	Chen, A. E. et al. Cell Stem Cell 4, 103-106 (2009)

hESC line:	**HUES65**
special features:	derived in serum free medium<br>laser-assisted ICM isolation
published in a peer-reviewed journal:	yes
characterization data reported in a peer-reviewed journal:	yes
reference:	Chen, A. E. et al. Cell Stem Cell 4, 103-106 (2009)

hESC line:	**HUES66**
special features:	derived in serum free medium<br>laser-assisted ICM isolation
published in a peer-reviewed journal:	yes
characterization data reported in a peer-reviewed journal:	yes
reference:	Chen, A. E. et al. Cell Stem Cell 4, 103-106 (2009)

hESC line:	**HUES67**
special features:	
published in a peer-reviewed journal:	no
characterization data reported in a peer-reviewed journal:	no
reference:	NIH Human Embryonic Stem Cell Registry (http://grants.nih.gov/stem_cells/registry/current.htm)

hESC line:	**HUES68**
special features:	
published in a peer-reviewed journal:	no
characterization data reported in a peer-reviewed journal:	no
reference:	NIH Human Embryonic Stem Cell Registry (http://grants.nih.gov/stem_cells/registry/current.htm)

hESC line:	**HUES69**
special features:	
published in a peer-reviewed journal:	no
characterization data reported in a peer-reviewed journal:	no
reference:	NIH Human Embryonic Stem Cell Registry (http://grants.nih.gov/stem_cells/registry/current.htm)

hESC line:	**HUES7**
special features:	derived in serum free medium
published in a peer-reviewed journal:	yes
characterization data reported in a peer-reviewed journal:	yes
reference:	Cowan, C. A. et al. N Engl J Med 350, 1353-1356 (2004)

hESC line:	**HUES70**
special features:	
published in a peer-reviewed journal:	no
characterization data reported in a peer-reviewed journal:	no
reference:	

hESC line:	**HUES8**
special features:	derived in serum free medium
published in a peer-reviewed journal:	yes
characterization data reported in a peer-reviewed journal:	yes
reference:	Cowan, C. A. et al. N Engl J Med 350, 1353-1356 (2004)

hESC line:	**HUES9**
special features:	derived in serum free medium
published in a peer-reviewed journal:	yes
characterization data reported in a peer-reviewed journal:	yes
reference:	Cowan, C. A. et al. N Engl J Med 350, 1353-1356 (2004)

Provider: International Stem Cell Research Institute, Los Angeles

hESC line:	**CSES1**
special features:	
published in a peer-reviewed journal:	yes
characterization data reported in a peer-reviewed journal:	yes
reference:	Lavon, N. et al. Stem Cells 26, 1874-1882 (2008)

hESC line:	**CSES2**
special features:	
published in a peer-reviewed journal:	yes
characterization data reported in a peer-reviewed journal:	yes
reference:	Lavon, N. et al. Stem Cells 26, 1874-1882 (2008)

hESC line:	**CSES3**
special features:	

published in a peer-reviewed journal:	yes
characterization data reported in a peer-reviewed journal:	yes
reference:	Lavon, N. et al. Stem Cells 26, 1874-1882 (2008)

hESC line:	**CSES4**
special features:	
published in a peer-reviewed journal:	yes
characterization data reported in a peer-reviewed journal:	yes
reference:	Lavon, N. et al. Stem Cells 26, 1874-1882 (2008)

hESC line:	**CSES5**
special features:	
published in a peer-reviewed journal:	yes
characterization data reported in a peer-reviewed journal:	yes
reference:	Lavon, N. et al. Stem Cells 26, 1874-1882 (2008)

hESC line:	**CSES6**
special features:	
published in a peer-reviewed journal:	yes
characterization data reported in a peer-reviewed journal:	yes
reference:	Lavon, N. et al. Stem Cells 26, 1874-1882 (2008)

hESC line:	**CSES7**
special features:	
published in a peer-reviewed journal:	yes
characterization data reported in a peer-reviewed journal:	yes
reference:	Lavon, N. et al. Stem Cells 26, 1874-1882 (2008)

Provider: Jones Institute for Reproductive Medicine, Eastern Virginia Medical School

hESC line:	**ES-76**
special features:	derived from research embryo

published in a peer-reviewed journal:	yes
characterization data reported in a peer-reviewed journal:	yes
reference:	Lanzendorf, S. E. et al. Fertil Steril 76, 132-137 (2001)

hESC line:	**ES-78-1**
special features:	derived from research embryo
published in a peer-reviewed journal:	yes
characterization data reported in a peer-reviewed journal:	no
reference:	Lanzendorf, S. E. et al. Fertil Steril 76, 132-137 (2001)

hESC line:	**ES-78-2**
special features:	derived from research embryo
published in a peer-reviewed journal:	yes
characterization data reported in a peer-reviewed journal:	no
reference:	Lanzendorf, S. E. et al. Fertil Steril 76, 132-137 (2001)

Provider: New York University School of Medicine

hESC line:	**NYUES1**
special features:	
published in a peer-reviewed journal:	no
characterization data reported in a peer-reviewed journal:	no
reference:	NIH Human Embryonic Stem Cell Registry (http://grants.nih.gov/stem_cells/registry/current.htm)

hESC line:	**NYUES2**
special features:	
published in a peer-reviewed journal:	no
characterization data reported in a peer-reviewed journal:	no
reference:	NIH Human Embryonic Stem Cell Registry (http://grants.nih.gov/stem_cells/registry/current.htm)

hESC line:	**NYUES3**
special features:	

published in a peer-reviewed journal:	no
characterization data reported in a peer-reviewed journal:	no
reference:	NIH Human Embryonic Stem Cell Registry (http://grants.nih.gov/stem_cells/registry/current.htm)

hESC line:	**NYUES4**
special features:	
published in a peer-reviewed journal:	no
characterization data reported in a peer-reviewed journal:	no
reference:	NIH Human Embryonic Stem Cell Registry (http://grants.nih.gov/stem_cells/registry/current.htm)

hESC line:	**NYUES5**
special features:	
published in a peer-reviewed journal:	no
characterization data reported in a peer-reviewed journal:	no
reference:	NIH Human Embryonic Stem Cell Registry (http://grants.nih.gov/stem_cells/registry/current.htm)

hESC line:	**NYUES6**
special features:	
published in a peer-reviewed journal:	no
characterization data reported in a peer-reviewed journal:	no
reference:	NIH Human Embryonic Stem Cell Registry (http://grants.nih.gov/stem_cells/registry/current.htm)

hESC line:	**NYUES7**
special features:	
published in a peer-reviewed journal:	no
characterization data reported in a peer-reviewed journal:	no
reference:	NIH Human Embryonic Stem Cell Registry (http://grants.nih.gov/stem_cells/registry/current.htm)

Provider: NovoCell Inc., San Diego, California, USA

hESC line:	**CyT203**
special features:	

published in a peer-reviewed journal:	yes
characterization data reported in a peer-reviewed journal:	no
reference:	D'Amour, K. A. et al. Nat Biotechnol 24, 1392-1401 (2006)

hESC line:	**CyT49**
special features:	derived under GMP conditions
published in a peer-reviewed journal:	yes
characterization data reported in a peer-reviewed journal:	no
reference:	D'Amour, K. A. et al. Nat Biotechnol 24, 1392-1401 (2006)

Provider: Reproductive Genetics Institute, Chicago, IL (partially distributed by SIL*)

hESC line:	**RG-148 (SI-148)**
special features:	genetic disorder: Dystrophya myotonica type 1 (DM1)
published in a peer-reviewed journal:	yes
characterization data reported in a peer-reviewed journal:	yes
reference:	Verlinsky, Y. et al. Reprod Biomed Online 10, 105-110 (2005)<br>NIH Human Embryonic Stem Cell Registry (http://grants.nih.gov/stem_cells/registry/current.htm)<br>Stemride International Stem Cell Bank (http://www.stemride.com/Stem_Cell_Bank.htm)

hESC line:	**RG-153 (SI-153)**
special features:	genetic disorder: Dystrophya myotonica type 1 (DM1)
published in a peer-reviewed journal:	yes
characterization data reported in a peer-reviewed journal:	yes
reference:	Verlinsky, Y. et al. Reprod Biomed Online 10, 105-110 (2005)<br>NIH Human Embryonic Stem Cell Registry (http://grants.nih.gov/stem_cells/registry/current.htm)<br>Stemride International Stem Cell Bank (http://www.stemride.com/Stem_Cell_Bank.htm)

hESC line:	**RG-170 (SI-170)**
special features:	genetic disorder: Muscular dystrophy, type Becker

* SIL, Stemride International Limited, UK

published in a peer-reviewed journal:	yes
characterization data reported in a peer-reviewed journal:	yes
reference:	Verlinsky, Y. et al. Reprod Biomed Online 10, 105-110 (2005)<br>NIH Human Embryonic Stem Cell Registry (http://grants.nih.gov/stem_cells/registry/current.htm)<br>Stemride International Stem Cell Bank (http://www.stemride.com/Stem_Cell_Bank.htm)

hESC line:	**RG-186 (SI-186)**
special features:	genetic disorder: Huntington's desease (HD)
published in a peer-reviewed journal:	yes
characterization data reported in a peer-reviewed journal:	yes
reference:	Verlinsky, Y. et al. Reprod Biomed Online 10, 105-110 (2005)<br>NIH Human Embryonic Stem Cell Registry (http://grants.nih.gov/stem_cells/registry/current.htm)<br>Stemride International Stem Cell Bank (http://www.stemride.com/Stem_Cell_Bank.htm)

hESC line:	**RG-194 (SI-194)**
special features:	genetic disorder: Huntington's desease (HD)
published in a peer-reviewed journal:	yes
characterization data reported in a peer-reviewed journal:	no
reference:	Verlinsky, Y. et al. Reprod Biomed Online 10, 105-110 (2005)<br>NIH Human Embryonic Stem Cell Registry (http://grants.nih.gov/stem_cells/registry/current.htm)<br>Stemride International Stem Cell Bank (http://www.stemride.com/Stem_Cell_Bank.htm)

hESC line:	**RG-222 (SI-222)**
special features:	
published in a peer-reviewed journal:	no
characterization data reported in a peer-reviewed journal:	no
reference:	NIH Human Embryonic Stem Cell Registry (http://grants.nih.gov/stem_cells/registry/current.htm)<br>Stemride International Stem Cell Bank (http://www.stemride.com/Stem_Cell_Bank.htm)

hESC line:	**RG-230 (SI-230)**
special features:	

published in a peer-reviewed journal:	no
characterization data reported in a peer-reviewed journal:	no
reference:	NIH Human Embryonic Stem Cell Registry (http://grants.nih.gov/stem_cells/registry/current.htm)<br>Stemride International Stem Cell Bank (http://www.stemride.com/Stem_Cell_Bank.htm)

hESC line:	**RG-233 (SI-233)**
special features:	genetic disorder: Sickle cell anaemia
published in a peer-reviewed journal:	yes
characterization data reported in a peer-reviewed journal:	no
reference:	NIH Human Embryonic Stem Cell Registry (http://grants.nih.gov/stem_cells/registry/current.htm)<br>Stemride International Stem Cell Bank (http://www.stemride.com/Stem_Cell_Bank.htm)<br>Verlinsky, Y. et al. Reprod Biomed Online 13, 547-550 (2006)

hESC line:	**RG-246 (SI-246)**
special features:	genetic disorder: Muscular dystrophy, type Emery Dreifuss
published in a peer-reviewed journal:	yes
characterization data reported in a peer-reviewed journal:	no
reference:	NIH Human Embryonic Stem Cell Registry (http://grants.nih.gov/stem_cells/registry/current.htm)<br>Stemride International Stem Cell Bank (http://www.stemride.com/Stem_Cell_Bank.htm)<br>Verlinsky, Y. et al. Reprod Biomed Online 13, 547-550 (2006)

hESC line:	**RG-249 (SI-249)**
special features:	
published in a peer-reviewed journal:	no
characterization data reported in a peer-reviewed journal:	no
reference:	Stemride International Stem Cell Bank (http://www.stemride.com/Stem_Cell_Bank.htm)

hESC line:	**RG-271 (SI-271)**
special features:	genetic disorder: Torsion dystonia (DYT1)

published in a peer-reviewed journal:	yes
characterization data reported in a peer-reviewed journal:	no
reference:	Stemride International Stem Cell Bank (http://www.stemride.com/Stem_Cell_Bank.htm)<br>Verlinsky, Y. et al. Reprod Biomed Online 13, 547-550 (2006)

hESC line:	**RG-283 (SI-283)**
special features:	genetic disorder: Muscular dystrophy, type Duchenne
published in a peer-reviewed journal:	yes
characterization data reported in a peer-reviewed journal:	no
reference:	Stemride International Stem Cell Bank (http://www.stemride.com/Stem_Cell_Bank.htm)<br>Verlinsky, Y. et al. Reprod Biomed Online 13, 547-550 (2006)

hESC line:	**RG-288 (SI-288)**
special features:	genetic disorder: Cystic fibrosis (CF)
published in a peer-reviewed journal:	no
characterization data reported in a peer-reviewed journal:	no
reference:	Stemride International Stem Cell Bank (http://www.stemride.com/Stem_Cell_Bank.htm)

hESC line:	**RG-289 (SI-289)**
special features:	genetic disorder: Cystic fibrosis (CF)
published in a peer-reviewed journal:	no
characterization data reported in a peer-reviewed journal:	no
reference:	NIH Human Embryonic Stem Cell Registry (http://grants.nih.gov/stem_cells/registry/current.htm)<br>Stemride Internatioinal Presentation "hESC Lines with Genetic and chromosomal disorders" , October 2008 (http://stemride.com/news.htm)

hESC line:	**RG-301 (SI-301)**
special features:	genetic disorder: Muscular dystrophy, type Duchenne

published in a peer-reviewed journal:	no
characterization data reported in a peer-reviewed journal:	no
reference:	NIH Human Embryonic Stem Cell Registry (http://grants.nih.gov/stem_cells/registry/current.htm)<br>Stemride International Stem Cell Bank (http://www.stemride.com/Stem_Cell_Bank.htm)

hESC line:	**RG-308**
special features:	
published in a peer-reviewed journal:	no
characterization data reported in a peer-reviewed journal:	no
reference:	NIH Human Embryonic Stem Cell Registry (http://grants.nih.gov/stem_cells/registry/current.htm)

hESC line:	**RG-313**
special features:	
published in a peer-reviewed journal:	no
characterization data reported in a peer-reviewed journal:	no
reference:	NIH Human Embryonic Stem Cell Registry (http://grants.nih.gov/stem_cells/registry/current.htm)

hESC line:	**RG-315 (SI-315)**
special features:	genetic disorder: Neurofibromatosis type 1 (affected)
published in a peer-reviewed journal:	no
characterization data reported in a peer-reviewed journal:	no
reference:	NIH Human Embryonic Stem Cell Registry (http://grants.nih.gov/stem_cells/registry/current.htm)<br>Stemride International Stem Cell Bank (http://www.stemride.com/Stem_Cell_Bank.htm)

hESC line:	**RG-316 (SI-316)**
special features:	genetic disorder: Tuberous sclerosis

published in a peer-reviewed journal:	no
characterization data reported in a peer-reviewed journal:	no
reference:	NIH Human Embryonic Stem Cell Registry (http://grants.nih.gov/stem_cells/registry/current.htm)<br>Stemride International Stem Cell Bank (http://www.stemride.com/Stem_Cell_Bank.htm)

hESC line:	**RG-320 (SI-320)**
special features:	genetic disorder: Tuberous sclerosis
published in a peer-reviewed journal:	no
characterization data reported in a peer-reviewed journal:	no
reference:	NIH Human Embryonic Stem Cell Registry (http://grants.nih.gov/stem_cells/registry/current.htm)<br>Stemride International Stem Cell Bank (http://www.stemride.com/Stem_Cell_Bank.htm)

hESC line:	**RG-326 (SI-326)**
special features:	genetic disorder: Popliteal Pterygium Syndrom (PPS)
published in a peer-reviewed journal:	no
characterization data reported in a peer-reviewed journal:	no
reference:	NIH Human Embryonic Stem Cell Registry (http://grants.nih.gov/stem_cells/registry/current.htm)<br>Stemride International Stem Cell Bank (http://www.stemride.com/Stem_Cell_Bank.htm)

hESC line:	**RG-328 (SI-328)**
special features:	genetic disorder: Facio Scapulo Humeral (FSH) muscular dystrophy
published in a peer-reviewed journal:	no
characterization data reported in a peer-reviewed journal:	no
reference:	NIH Human Embryonic Stem Cell Registry (http://grants.nih.gov/stem_cells/registry/current.htm)<br>Stemride International Stem Cell Bank (http://www.stemride.com/Stem_Cell_Bank.htm)

hESC line:	**RG-330 (SI-330)**
special features:	genetic disorder: Facio Scapulo Humeral (FSH) muscular dystrophy

published in a peer-reviewed journal:	no
characterization data reported in a peer-reviewed journal:	no
reference:	NIH Human Embryonic Stem Cell Registry (http://grants.nih.gov/stem_cells/registry/current.htm)<br>Stemride International Stem Cell Bank (http://www.stemride.com/Stem_Cell_Bank.htm)

hESC line:	**RG-333 (SI-333)**
special features:	genetic disorder: Facio Scapulo Humeral (FSH) muscular dystrophy
published in a peer-reviewed journal:	no
characterization data reported in a peer-reviewed journal:	no
reference:	NIH Human Embryonic Stem Cell Registry (http://grants.nih.gov/stem_cells/registry/current.htm)<br>Stemride International Stem Cell Bank (http://www.stemride.com/Stem_Cell_Bank.htm)

hESC line:	**RG-344 (SI-344)**
special features:	
published in a peer-reviewed journal:	yes
characterization data reported in a peer-reviewed journal:	no
reference:	Verlinsky, Y. et al. Reprod Biomed Online 18, 120-126 (2009)

hESC line:	**RG-356**
special features:	genetic disorder: alpha-Thalassaemia (affected)
published in a peer-reviewed journal:	no
characterization data reported in a peer-reviewed journal:	no
reference:	NIH Human Embryonic Stem Cell Registry (http://grants.nih.gov/stem_cells/registry/current.htm)

hESC line:	**RG-357**
special features:	genetic disorder: Muscular dystrophy, type Emery Dreifuss
published in a peer-reviewed journal:	no
characterization data reported in a peer-reviewed journal:	no
reference:	NIH Human Embryonic Stem Cell Registry (http://grants.nih.gov/stem_cells/registry/current.htm)

hESC line:	**RG-358**
special features:	genetic disorder: Muscular dystrophy, type Emery Dreifuss
published in a peer-reviewed journal:	no
characterization data reported in a peer-reviewed journal:	no
reference:	NIH Human Embryonic Stem Cell Registry (http://grants.nih.gov/stem_cells/registry/current.htm)

hESC line:	**RG-399 (SI-399)**
special features:	genetic disorder: Facio Scapulo Humeral (FSH) muscular dystrophy
published in a peer-reviewed journal:	no
characterization data reported in a peer-reviewed journal:	no
reference:	NIH Human Embryonic Stem Cell Registry (http://grants.nih.gov/stem_cells/registry/current.htm)<br>Stemride International Stem Cell Bank (http://www.stemride.com/Stem_Cell_Bank.htm)

hESC line:	**RG-401 (SI-401)**
special features:	genetic disorder: Facio Scapulo Humeral (FSH) muscular dystrophy
published in a peer-reviewed journal:	no
characterization data reported in a peer-reviewed journal:	no
reference:	NIH Human Embryonic Stem Cell Registry (http://grants.nih.gov/stem_cells/registry/current.htm)<br>Stemride International Stem Cell Bank (http://www.stemride.com/Stem_Cell_Bank.htm)

hESC line:	**RG-402 (SI-402)**
special features:	genetic disorder: Facio Scapulo Humeral (FSH) muscular dystrophy
published in a peer-reviewed journal:	no
characterization data reported in a peer-reviewed journal:	no
reference:	NIH Human Embryonic Stem Cell Registry (http://grants.nih.gov/stem_cells/registry/current.htm)<br>Stemride International Stem Cell Bank (http://www.stemride.com/Stem_Cell_Bank.htm)

hESC line:	**RG-403 (SI-403)**
special features:	genetic disorder: Facio Scapulo Humeral (FSH) muscular dystrophy

published in a peer-reviewed journal:	no
characterization data reported in a peer-reviewed journal:	no
reference:	NIH Human Embryonic Stem Cell Registry (http://grants.nih.gov/stem_cells/registry/current.htm)<br>Stemride International Stem Cell Bank (http://www.stemride.com/Stem_Cell_Bank.htm)

hESC line:	**RG-404 (SI-404)**
special features:	genetic disorder: Spinal muscular atrophy type 1 (SMA1)
published in a peer-reviewed journal:	no
characterization data reported in a peer-reviewed journal:	no
reference:	NIH Human Embryonic Stem Cell Registry (http://grants.nih.gov/stem_cells/registry/current.htm)<br>Stemride International Stem Cell Bank (http://www.stemride.com/Stem_Cell_Bank.htm)

hESC line:	**RG-406 (SI-406)**
special features:	genetic disorder: Torsion dystonia (DYT1)
published in a peer-reviewed journal:	no
characterization data reported in a peer-reviewed journal:	no
reference:	NIH Human Embryonic Stem Cell Registry (http://grants.nih.gov/stem_cells/registry/current.htm)<br>Stemride International Stem Cell Bank (http://www.stemride.com/Stem_Cell_Bank.htm)

hESC line:	**RG-413**
special features:	genetic disorder: Familial breast cancer (BRCA2)<br>genetic disorder: Multiple endocrine neoplasia type 1
published in a peer-reviewed journal:	no
characterization data reported in a peer-reviewed journal:	no
reference:	NIH Human Embryonic Stem Cell Registry (http://grants.nih.gov/stem_cells/registry/current.htm)

hESC line:	**RG-414**
special features:	genetic disorder: Multiple endocrine neoplasia type 1

published in a peer-reviewed journal:	no
characterization data reported in a peer-reviewed journal:	no
reference:	NIH Human Embryonic Stem Cell Registry (http://grants.nih.gov/stem_cells/registry/current.htm)

hESC line:	**RG-415**
special features:	genetic disorder: Multiple endocrine neoplasia type 2
published in a peer-reviewed journal:	no
characterization data reported in a peer-reviewed journal:	no
reference:	NIH Human Embryonic Stem Cell Registry (http://grants.nih.gov/stem_cells/registry/current.htm)

hESC line:	**RG-416**
special features:	genetic disorder: Cystic fibrosis (CF)
published in a peer-reviewed journal:	no
characterization data reported in a peer-reviewed journal:	no
reference:	NIH Human Embryonic Stem Cell Registry (http://grants.nih.gov/stem_cells/registry/current.htm)

hESC line:	**RG-417**
special features:	genetic disorder: Cystic fibrosis (CF)
published in a peer-reviewed journal:	no
characterization data reported in a peer-reviewed journal:	no
reference:	NIH Human Embryonic Stem Cell Registry (http://grants.nih.gov/stem_cells/registry/current.htm)

hESC line:	**RG-418**
special features:	genetic disorder: Hemoglobin Beta Locus Mutation (cd8+G / 619del)
published in a peer-reviewed journal:	no
characterization data reported in a peer-reviewed journal:	no
reference:	NIH Human Embryonic Stem Cell Registry (http://grants.nih.gov/stem_cells/registry/current.htm)

hESC line:	**RG-420**
special features:	genetic disorder: Hemoglobin Beta Locus Mutation (cd8+G / 619del)

published in a peer-reviewed journal:	no
characterization data reported in a peer-reviewed journal:	no
reference:	NIH Human Embryonic Stem Cell Registry (http://grants.nih.gov/stem_cells/registry/current.htm)

hESC line:	**RG-422**
special features:	genetic disorder: Cystic fibrosis (CF)
published in a peer-reviewed journal:	no
characterization data reported in a peer-reviewed journal:	no
reference:	NIH Human Embryonic Stem Cell Registry (http://grants.nih.gov/stem_cells/registry/current.htm)

hESC line:	**RG-423**
special features:	genetic disorder: Cystic fibrosis (CF)
published in a peer-reviewed journal:	no
characterization data reported in a peer-reviewed journal:	no
reference:	NIH Human Embryonic Stem Cell Registry (http://grants.nih.gov/stem_cells/registry/current.htm)

hESC line:	**RG-424**
special features:	genetic disorder: Multiple endocrine neoplasia type 2
published in a peer-reviewed journal:	no
characterization data reported in a peer-reviewed journal:	no
reference:	NIH Human Embryonic Stem Cell Registry (http://grants.nih.gov/stem_cells/registry/current.htm)

hESC line:	**RG-426**
special features:	genetic disorder: Pelizaeus-Merzbacher Disease (PMLD)
published in a peer-reviewed journal:	no
characterization data reported in a peer-reviewed journal:	no
reference:	NIH Human Embryonic Stem Cell Registry (http://grants.nih.gov/stem_cells/registry/current.htm)

hESC line:	**RG-428**
special features:	genetic disorder: Tuberous sclerosis

published in a peer-reviewed journal:	no
characterization data reported in a peer-reviewed journal:	no
reference:	NIH Human Embryonic Stem Cell Registry (http://grants.nih.gov/stem_cells/registry/current.htm)

hESC line:	**SI-100**
special features:	
published in a peer-reviewed journal:	no
characterization data reported in a peer-reviewed journal:	no
reference:	Stemride International Stem Cell Bank (http://www.stemride.com/Stem_Cell_Bank.htm)

hESC line:	**SI-101**
special features:	
published in a peer-reviewed journal:	no
characterization data reported in a peer-reviewed journal:	no
reference:	Stemride International Stem Cell Bank (http://www.stemride.com/Stem_Cell_Bank.htm)

hESC line:	**SI-102**
special features:	
published in a peer-reviewed journal:	no
characterization data reported in a peer-reviewed journal:	no
reference:	Stemride International Stem Cell Bank (http://www.stemride.com/Stem_Cell_Bank.htm)

hESC line:	**SI-103**
special features:	
published in a peer-reviewed journal:	no
characterization data reported in a peer-reviewed journal:	no
reference:	Stemride International Stem Cell Bank (http://www.stemride.com/Stem_Cell_Bank.htm)

hESC line:	**SI-104 (hESC-104)**
special features:	abnormal karyotype

published in a peer-reviewed journal:	yes
characterization data reported in a peer-reviewed journal:	no
reference:	Stemride International Stem Cell Bank (http://www.stemride.com/Stem_Cell_Bank.htm)<br>Verlinsky, Y. et al. Reprod Biomed Online 13, 547-550 (2006)

hESC line:	**SI-105**
special features:	
published in a peer-reviewed journal:	no
characterization data reported in a peer-reviewed journal:	no
reference:	Stemride International Stem Cell Bank (http://www.stemride.com/Stem_Cell_Bank.htm)

hESC line:	**SI-106**
special features:	
published in a peer-reviewed journal:	no
characterization data reported in a peer-reviewed journal:	no
reference:	Stemride International Stem Cell Bank (http://www.stemride.com/Stem_Cell_Bank.htm)

hESC line:	**SI-107**
special features:	
published in a peer-reviewed journal:	no
characterization data reported in a peer-reviewed journal:	no
reference:	Stemride International Stem Cell Bank (http://www.stemride.com/Stem_Cell_Bank.htm)

hESC line:	**SI-108**
special features:	
published in a peer-reviewed journal:	no
characterization data reported in a peer-reviewed journal:	no
reference:	Stemride International Stem Cell Bank (http://www.stemride.com/Stem_Cell_Bank.htm)

hESC line:	**SI-109**
special features:	

published in a peer-reviewed journal:	no
characterization data reported in a peer-reviewed journal:	no
reference:	Stemride International Stem Cell Bank (http://www.stemride.com/Stem_Cell_Bank.htm)

hESC line:	**SI-110**
special features:	
published in a peer-reviewed journal:	no
characterization data reported in a peer-reviewed journal:	no
reference:	Stemride International Stem Cell Bank (http://www.stemride.com/Stem_Cell_Bank.htm)

hESC line:	**SI-111**
special features:	
published in a peer-reviewed journal:	no
characterization data reported in a peer-reviewed journal:	no
reference:	Stemride International Stem Cell Bank (http://www.stemride.com/Stem_Cell_Bank.htm)

hESC line:	**SI-114**
special features:	
published in a peer-reviewed journal:	no
characterization data reported in a peer-reviewed journal:	no
reference:	Stemride International Stem Cell Bank (http://www.stemride.com/Stem_Cell_Bank.htm)

hESC line:	**SI-115**
special features:	
published in a peer-reviewed journal:	no
characterization data reported in a peer-reviewed journal:	no
reference:	Stemride International Stem Cell Bank (http://www.stemride.com/Stem_Cell_Bank.htm)

hESC line:	**SI-122**
special features:	

published in a peer-reviewed journal:	no
characterization data reported in a peer-reviewed journal:	no
reference:	Stemride International Stem Cell Bank (http://www.stemride.com/Stem_Cell_Bank.htm)

hESC line:	**SI-123**
special features:	
published in a peer-reviewed journal:	no
characterization data reported in a peer-reviewed journal:	no
reference:	Stemride International Stem Cell Bank (http://www.stemride.com/Stem_Cell_Bank.htm)

hESC line:	**SI-124**
special features:	
published in a peer-reviewed journal:	no
characterization data reported in a peer-reviewed journal:	no
reference:	Stemride International Stem Cell Bank (http://www.stemride.com/Stem_Cell_Bank.htm)

hESC line:	**SI-125**
special features:	genetic disorder: Fragile X syndrome (FX)
published in a peer-reviewed journal:	yes
characterization data reported in a peer-reviewed journal:	yes
reference:	Verlinsky, Y. et al. Reprod Biomed Online 10, 105-110 (2005)<br>Stemride International Stem Cell Bank (http://www.stemride.com/Stem_Cell_Bank.htm)

hESC line:	**SI-126**
special features:	
published in a peer-reviewed journal:	no
characterization data reported in a peer-reviewed journal:	no
reference:	Stemride International Stem Cell Bank (http://www.stemride.com/Stem_Cell_Bank.htm)

hESC line:	**SI-128**
special features:	genetic disorder: Fanconi anaemia (carrier)

published in a peer-reviewed journal:	yes
characterization data reported in a peer-reviewed journal:	yes
reference:	Verlinsky, Y. et al. Reprod Biomed Online 10, 105-110 (2005)<br>Stemride International Stem Cell Bank (http://www.stemride.com/Stem_Cell_Bank.htm)

hESC line:	**SI-130**
special features:	
published in a peer-reviewed journal:	no
characterization data reported in a peer-reviewed journal:	no
reference:	Stemride International Stem Cell Bank (http://www.stemride.com/Stem_Cell_Bank.htm)

hESC line:	**SI-131**
special features:	
published in a peer-reviewed journal:	no
characterization data reported in a peer-reviewed journal:	no
reference:	Stemride International Stem Cell Bank (http://www.stemride.com/Stem_Cell_Bank.htm)

hESC line:	**SI-132**
special features:	
published in a peer-reviewed journal:	no
characterization data reported in a peer-reviewed journal:	no
reference:	Stemride International Stem Cell Bank (http://www.stemride.com/Stem_Cell_Bank.htm)

hESC line:	**SI-133**
special features:	
published in a peer-reviewed journal:	no
characterization data reported in a peer-reviewed journal:	no
reference:	Stemride International Stem Cell Bank (http://www.stemride.com/Stem_Cell_Bank.htm)

hESC line:	**SI-134**
special features:	

published in a peer-reviewed journal:	no
characterization data reported in a peer-reviewed journal:	no
reference:	Stemride International Stem Cell Bank (http://www.stemride.com/Stem_Cell_Bank.htm)

hESC line:	**SI-135**
special features:	
published in a peer-reviewed journal:	no
characterization data reported in a peer-reviewed journal:	no
reference:	Stemride International Stem Cell Bank (http://www.stemride.com/Stem_Cell_Bank.htm)

hESC line:	**SI-137**
special features:	genetic disorder: Neurofibromatosis type 1 (affected)
published in a peer-reviewed journal:	yes
characterization data reported in a peer-reviewed journal:	yes
reference:	Verlinsky, Y. et al. Reprod Biomed Online 10, 105-110 (2005)<br>Stemride International Stem Cell Bank (http://www.stemride.com/Stem_Cell_Bank.htm)

hESC line:	**SI-138**
special features:	genetic disorder: Neurofibromatosis type 1 (affected)
published in a peer-reviewed journal:	yes
characterization data reported in a peer-reviewed journal:	yes
reference:	Verlinsky, Y. et al. Reprod Biomed Online 10, 105-110 (2005)<br>Stemride International Stem Cell Bank (http://www.stemride.com/Stem_Cell_Bank.htm)

hESC line:	**SI-139**
special features:	genetic disorder: Neurofibromatosis type 1 (affected)
published in a peer-reviewed journal:	yes
characterization data reported in a peer-reviewed journal:	yes
reference:	Verlinsky, Y. et al. Reprod Biomed Online 10, 105-110 (2005)<br>Stemride International Stem Cell Bank (http://www.stemride.com/Stem_Cell_Bank.htm)

hESC line:	**SI-140**
special features:	genetic disorder: Neurofibromatosis type 1 (affected)
published in a peer-reviewed journal:	yes
characterization data reported in a peer-reviewed journal:	yes
reference:	Verlinsky, Y. et al. Reprod Biomed Online 10, 105-110 (2005)<br>Stemride International Stem Cell Bank (http://www.stemride.com/Stem_Cell_Bank.htm)

hESC line:	**SI-141**
special features:	genetic disorder: Neurofibromatosis type 1 (affected)
published in a peer-reviewed journal:	yes
characterization data reported in a peer-reviewed journal:	yes
reference:	Verlinsky, Y. et al. Reprod Biomed Online 10, 105-110 (2005)<br>Stemride International Stem Cell Bank (http://www.stemride.com/Stem_Cell_Bank.htm)

hESC line:	**SI-144**
special features:	
published in a peer-reviewed journal:	no
characterization data reported in a peer-reviewed journal:	no
reference:	Stemride International Stem Cell Bank (http://www.stemride.com/Stem_Cell_Bank.htm)

hESC line:	**SI-145 (hESC-145)**
special features:	abnormal karyotype
published in a peer-reviewed journal:	yes
characterization data reported in a peer-reviewed journal:	no
reference:	Stemride International Stem Cell Bank (http://www.stemride.com/Stem_Cell_Bank.htm)<br>Verlinsky, Y. et al. Reprod Biomed Online 13, 547-550 (2006)

hESC line:	**SI-146**
special features:	

published in a peer-reviewed journal:	no
characterization data reported in a peer-reviewed journal:	no
reference:	Stemride International Stem Cell Bank (http://www.stemride.com/Stem_Cell_Bank.htm)

hESC line:	**SI-149**
special features:	
published in a peer-reviewed journal:	no
characterization data reported in a peer-reviewed journal:	no
reference:	Stemride International Stem Cell Bank (http://www.stemride.com/Stem_Cell_Bank.htm)

hESC line:	**SI-15**
special features:	established from morula stage embryo
published in a peer-reviewed journal:	yes
characterization data reported in a peer-reviewed journal:	yes
reference:	Strelchenko, N. et al. Reprod Biomed Online 9, 623-629 (2004)<br>Stemride International Stem Cell Bank (http://www.stemride.com/Stem_Cell_Bank.htm)

hESC line:	**SI-150**
special features:	
published in a peer-reviewed journal:	no
characterization data reported in a peer-reviewed journal:	no
reference:	Stemride International Stem Cell Bank (http://www.stemride.com/Stem_Cell_Bank.htm)

hESC line:	**SI-151**
special features:	
published in a peer-reviewed journal:	no
characterization data reported in a peer-reviewed journal:	no
reference:	Stemride International Stem Cell Bank (http://www.stemride.com/Stem_Cell_Bank.htm)

hESC line:	**SI-154**
special features:	genetic disorder: Marfan syndrome (MFS)

published in a peer-reviewed journal:	yes
characterization data reported in a peer-reviewed journal:	yes
reference:	Verlinsky, Y. et al. Reprod Biomed Online 10, 105-110 (2005)<br>Stemride International Stem Cell Bank (http://www.stemride.com/Stem_Cell_Bank.htm)

hESC line:	**SI-155 (SC-155)**
special features:	
published in a peer-reviewed journal:	yes
characterization data reported in a peer-reviewed journal:	no
reference:	Stemride International Stem Cell Bank (http://www.stemride.com/Stem_Cell_Bank.htm)<br>Verlinsky, Y. et al. Reprod Biomed Online 18, 120-126 (2009)

hESC line:	**SI-156**
special features:	
published in a peer-reviewed journal:	no
characterization data reported in a peer-reviewed journal:	no
reference:	Stemride International Stem Cell Bank (http://www.stemride.com/Stem_Cell_Bank.htm)

hESC line:	**SI-157**
special features:	
published in a peer-reviewed journal:	no
characterization data reported in a peer-reviewed journal:	no
reference:	Stemride International Stem Cell Bank (http://www.stemride.com/Stem_Cell_Bank.htm)

hESC line:	**SI-158**
special features:	genetic disorder: beta-Thalassaemia (carrier)
published in a peer-reviewed journal:	yes
characterization data reported in a peer-reviewed journal:	yes
reference:	Verlinsky, Y. et al. Reprod Biomed Online 10, 105-110 (2005)<br>Stemride International Stem Cell Bank (http://www.stemride.com/Stem_Cell_Bank.htm)

hESC line:	**SI-159 (SC-159)**
special features:	
published in a peer-reviewed journal:	yes
characterization data reported in a peer-reviewed journal:	no
reference:	Stemride International Stem Cell Bank (http://www.stemride.com/Stem_Cell_Bank.htm)<br>Verlinsky, Y. et al. Reprod Biomed Online 18, 120-126 (2009)

hESC line:	**SI-160 (SC-160)**
special features:	
published in a peer-reviewed journal:	yes
characterization data reported in a peer-reviewed journal:	no
reference:	Stemride International Stem Cell Bank (http://www.stemride.com/Stem_Cell_Bank.htm)<br>Verlinsky, Y. et al. Reprod Biomed Online 18, 120-126 (2009)

hESC line:	**SI-161**
special features:	
published in a peer-reviewed journal:	no
characterization data reported in a peer-reviewed journal:	no
reference:	Stemride International Stem Cell Bank (http://www.stemride.com/Stem_Cell_Bank.htm)

hESC line:	**SI-162**
special features:	
published in a peer-reviewed journal:	no
characterization data reported in a peer-reviewed journal:	no
reference:	Stemride International Stem Cell Bank (http://www.stemride.com/Stem_Cell_Bank.htm)

hESC line:	**SI-163**
special features:	
published in a peer-reviewed journal:	no
characterization data reported in a peer-reviewed journal:	no
reference:	Stemride International Stem Cell Bank (http://www.stemride.com/Stem_Cell_Bank.htm)

hESC line:	**SI-164**
special features:	genetic disorder: beta-Thalassaemia (affected)
published in a peer-reviewed journal:	yes
characterization data reported in a peer-reviewed journal:	yes
reference:	Verlinsky, Y. et al. Reprod Biomed Online 10, 105-110 (2005)<br>Stemride International Stem Cell Bank (http://www.stemride.com/Stem_Cell_Bank.htm)

hESC line:	**SI-165**
special features:	
published in a peer-reviewed journal:	no
characterization data reported in a peer-reviewed journal:	no
reference:	Stemride International Stem Cell Bank (http://www.stemride.com/Stem_Cell_Bank.htm)

hESC line:	**SI-167**
special features:	
published in a peer-reviewed journal:	no
characterization data reported in a peer-reviewed journal:	no
reference:	Stemride International Stem Cell Bank (http://www.stemride.com/Stem_Cell_Bank.htm)

hESC line:	**SI-168 (hESC-168)**
special features:	abnormal karyotype
published in a peer-reviewed journal:	yes
characterization data reported in a peer-reviewed journal:	no
reference:	Stemride International Stem Cell Bank (http://www.stemride.com/Stem_Cell_Bank.htm)<br>Verlinsky, Y. et al. Reprod Biomed Online 13, 547-550 (2006)

hESC line:	**SI-169**
special features:	
published in a peer-reviewed journal:	no
characterization data reported in a peer-reviewed journal:	no
reference:	Stemride International Stem Cell Bank (http://www.stemride.com/Stem_Cell_Bank.htm)

hESC line:	**SI-171**
special features:	
published in a peer-reviewed journal:	no
characterization data reported in a peer-reviewed journal:	no
reference:	Stemride International Stem Cell Bank (http://www.stemride.com/Stem_Cell_Bank.htm)

hESC line:	**SI-172**
special features:	
published in a peer-reviewed journal:	no
characterization data reported in a peer-reviewed journal:	no
reference:	Stemride International Stem Cell Bank (http://www.stemride.com/Stem_Cell_Bank.htm)

hESC line:	**SI-174**
special features:	
published in a peer-reviewed journal:	no
characterization data reported in a peer-reviewed journal:	no
reference:	Stemride International Stem Cell Bank (http://www.stemride.com/Stem_Cell_Bank.htm)

hESC line:	**SI-175**
special features:	
published in a peer-reviewed journal:	no
characterization data reported in a peer-reviewed journal:	no
reference:	Stemride International Stem Cell Bank (http://www.stemride.com/Stem_Cell_Bank.htm)

hESC line:	**SI-176**
special features:	
published in a peer-reviewed journal:	no
characterization data reported in a peer-reviewed journal:	no
reference:	Stemride International Stem Cell Bank (http://www.stemride.com/Stem_Cell_Bank.htm)

hESC line:	**SI-177**
special features:	

published in a peer-reviewed journal:	no
characterization data reported in a peer-reviewed journal:	no
reference:	Stemride International Stem Cell Bank (http://www.stemride.com/Stem_Cell_Bank.htm)

hESC line:	**SI-178**
special features:	
published in a peer-reviewed journal:	no
characterization data reported in a peer-reviewed journal:	no
reference:	Stemride International Stem Cell Bank (http://www.stemride.com/Stem_Cell_Bank.htm)

hESC line:	**SI-179**
special features:	
published in a peer-reviewed journal:	no
characterization data reported in a peer-reviewed journal:	no
reference:	Stemride International Stem Cell Bank (http://www.stemride.com/Stem_Cell_Bank.htm)

hESC line:	**SI-18**
special features:	established from morula stage embryo
published in a peer-reviewed journal:	yes
characterization data reported in a peer-reviewed journal:	yes
reference:	Strelchenko, N. et al. Reprod Biomed Online 9, 623-629 (2004)<br>Stemride International Stem Cell Bank (http://www.stemride.com/Stem_Cell_Bank.htm)

hESC line:	**SI-180**
special features:	genetic disorder: Muscular dystrophy, type Duchenne
published in a peer-reviewed journal:	yes
characterization data reported in a peer-reviewed journal:	yes
reference:	Verlinsky, Y. et al. Reprod Biomed Online 10, 105-110 (2005)<br>Stemride International Stem Cell Bank (http://www.stemride.com/Stem_Cell_Bank.htm)

hESC line:	**SI-182**
special features:	

published in a peer-reviewed journal:	no
characterization data reported in a peer-reviewed journal:	no
reference:	Stemride International Stem Cell Bank (http://www.stemride.com/Stem_Cell_Bank.htm)

hESC line:	**SI-183 (SC-183)**
special features:	
published in a peer-reviewed journal:	yes
characterization data reported in a peer-reviewed journal:	no
reference:	Stemride International Stem Cell Bank (http://www.stemride.com/Stem_Cell_Bank.htm)<br>Verlinsky, Y. et al. Reprod Biomed Online 18, 120-126 (2009)

hESC line:	**SI-184 (SC-184, hESC-184)**
special features:	abnormal karyotype
published in a peer-reviewed journal:	yes
characterization data reported in a peer-reviewed journal:	no
reference:	Stemride International Stem Cell Bank (http://www.stemride.com/Stem_Cell_Bank.htm)<br>Verlinsky, Y. et al. Reprod Biomed Online 18, 120-126 (2009)

hESC line:	**SI-185 (SC-185)**
special features:	
published in a peer-reviewed journal:	yes
characterization data reported in a peer-reviewed journal:	no
reference:	Stemride International Stem Cell Bank (http://www.stemride.com/Stem_Cell_Bank.htm)<br>Verlinsky, Y. et al. Reprod Biomed Online 18, 120-126 (2009)

hESC line:	**SI-187**
special features:	genetic disorder: Huntington's desease (HD)
published in a peer-reviewed journal:	yes
characterization data reported in a peer-reviewed journal:	yes
reference:	Verlinsky, Y. et al. Reprod Biomed Online 10, 105-110 (2005)<br>Stemride International Stem Cell Bank (http://www.stemride.com/Stem_Cell_Bank.htm)

hESC line:	**SI-188**
special features:	
published in a peer-reviewed journal:	no
characterization data reported in a peer-reviewed journal:	no
reference:	Stemride International Stem Cell Bank (http://www.stemride.com/Stem_Cell_Bank.htm)

hESC line:	**SI-189**
special features:	
published in a peer-reviewed journal:	no
characterization data reported in a peer-reviewed journal:	no
reference:	Stemride International Stem Cell Bank (http://www.stemride.com/Stem_Cell_Bank.htm)

hESC line:	**SI-191**
special features:	
published in a peer-reviewed journal:	no
characterization data reported in a peer-reviewed journal:	no
reference:	Stemride International Stem Cell Bank (http://www.stemride.com/Stem_Cell_Bank.htm)

hESC line:	**SI-192**
special features:	
published in a peer-reviewed journal:	no
characterization data reported in a peer-reviewed journal:	no
reference:	Stemride International Stem Cell Bank (http://www.stemride.com/Stem_Cell_Bank.htm)

hESC line:	**SI-193**
special features:	
published in a peer-reviewed journal:	no
characterization data reported in a peer-reviewed journal:	no
reference:	Stemride International Stem Cell Bank (http://www.stemride.com/Stem_Cell_Bank.htm)

hESC line:	**SI-195**
special features:	

published in a peer-reviewed journal:	no
characterization data reported in a peer-reviewed journal:	no
reference:	Stemride International Stem Cell Bank (http://www.stemride.com/Stem_Cell_Bank.htm)

hESC line:	**SI-196**
special features:	
published in a peer-reviewed journal:	no
characterization data reported in a peer-reviewed journal:	no
reference:	Stemride International Stem Cell Bank (http://www.stemride.com/Stem_Cell_Bank.htm)

hESC line:	**SI-197**
special features:	abnormal karyotype
published in a peer-reviewed journal:	yes
characterization data reported in a peer-reviewed journal:	no
reference:	Stemride International Stem Cell Bank (http://www.stemride.com/Stem_Cell_Bank.htm)<br>Verlinsky, Y. et al. Reprod Biomed Online 13, 547-550 (2006)

hESC line:	**SI-198**
special features:	
published in a peer-reviewed journal:	no
characterization data reported in a peer-reviewed journal:	no
reference:	Stemride International Stem Cell Bank (http://www.stemride.com/Stem_Cell_Bank.htm)

hESC line:	**SI-199 (SC-199)**
special features:	
published in a peer-reviewed journal:	yes
characterization data reported in a peer-reviewed journal:	no
reference:	Stemride International Stem Cell Bank (http://www.stemride.com/Stem_Cell_Bank.htm)<br>Verlinsky, Y. et al. Reprod Biomed Online 18, 120-126 (2009)

hESC line:	**SI-200**
special features:	

published in a peer-reviewed journal:	no
characterization data reported in a peer-reviewed journal:	no
reference:	Stemride International Stem Cell Bank (http://www.stemride.com/Stem_Cell_Bank.htm)

hESC line:	**SI-201**
special features:	genetic disorder: Adrenoleukodystrophy (affected)
published in a peer-reviewed journal:	yes
characterization data reported in a peer-reviewed journal:	yes
reference:	Verlinsky, Y. et al. Reprod Biomed Online 10, 105-110 (2005)<br>Stemride International Stem Cell Bank (http://www.stemride.com/Stem_Cell_Bank.htm)

hESC line:	**SI-202**
special features:	
published in a peer-reviewed journal:	no
characterization data reported in a peer-reviewed journal:	no
reference:	Stemride International Stem Cell Bank (http://www.stemride.com/Stem_Cell_Bank.htm)

hESC line:	**SI-203**
special features:	
published in a peer-reviewed journal:	no
characterization data reported in a peer-reviewed journal:	no
reference:	Stemride International Stem Cell Bank (http://www.stemride.com/Stem_Cell_Bank.htm)

hESC line:	**SI-204**
special features:	
published in a peer-reviewed journal:	no
characterization data reported in a peer-reviewed journal:	no
reference:	Stemride International Stem Cell Bank (http://www.stemride.com/Stem_Cell_Bank.htm)

hESC line:	**SI-205**
special features:	

published in a peer-reviewed journal:	no
characterization data reported in a peer-reviewed journal:	no
reference:	Stemride International Stem Cell Bank (http://www.stemride.com/Stem_Cell_Bank.htm)

hESC line:	**SI-206 (SC-206)**
special features:	
published in a peer-reviewed journal:	yes
characterization data reported in a peer-reviewed journal:	no
reference:	Stemride International Stem Cell Bank (http://www.stemride.com/Stem_Cell_Bank.htm)<br>Verlinsky, Y. et al. Reprod Biomed Online 18, 120-126 (2009)

hESC line:	**SI-208 (hESC-208)**
special features:	abnormal karyotype
published in a peer-reviewed journal:	yes
characterization data reported in a peer-reviewed journal:	no
reference:	Stemride International Stem Cell Bank (http://www.stemride.com/Stem_Cell_Bank.htm)<br>Verlinsky, Y. et al. Reprod Biomed Online 13, 547-550 (2006)

hESC line:	**SI-209 (SC-209)**
special features:	
published in a peer-reviewed journal:	yes
characterization data reported in a peer-reviewed journal:	no
reference:	Stemride International Stem Cell Bank (http://www.stemride.com/Stem_Cell_Bank.htm)<br>Verlinsky, Y. et al. Reprod Biomed Online 18, 120-126 (2009)

hESC line:	**SI-21**
special features:	established from morula stage embryo
published in a peer-reviewed journal:	yes
characterization data reported in a peer-reviewed journal:	yes
reference:	Strelchenko, N. et al. Reprod Biomed Online 9, 623-629 (2004)<br>Stemride International Stem Cell Bank (http://www.stemride.com/Stem_Cell_Bank.htm)

hESC line:	**SI-210**
special features:	
published in a peer-reviewed journal:	no
characterization data reported in a peer-reviewed journal:	no
reference:	Stemride International Stem Cell Bank (http://www.stemride.com/Stem_Cell_Bank.htm)

hESC line:	**SI-211**
special features:	
published in a peer-reviewed journal:	no
characterization data reported in a peer-reviewed journal:	no
reference:	Stemride International Stem Cell Bank (http://www.stemride.com/Stem_Cell_Bank.htm)

hESC line:	**SI-213**
special features:	genetic disorder: beta-Thalassaemia (carrier)
published in a peer-reviewed journal:	no
characterization data reported in a peer-reviewed journal:	no
reference:	Stemride International Stem Cell Bank (http://www.stemride.com/Stem_Cell_Bank.htm)<br>Verlinsky, Y. et al. Reprod Biomed Online 13, 547-550 (2006)

hESC line:	**SI-214**
special features:	genetic disorder: Fragile X syndrome (FX)
published in a peer-reviewed journal:	no
characterization data reported in a peer-reviewed journal:	no
reference:	Stemride International Stem Cell Bank (http://www.stemride.com/Stem_Cell_Bank.htm)<br>Verlinsky, Y. et al. Reprod Biomed Online 13, 547-550 (2006)

hESC line:	**SI-215**
special features:	
published in a peer-reviewed journal:	yes
characterization data reported in a peer-reviewed journal:	no
reference:	Stemride International Stem Cell Bank (http://www.stemride.com/Stem_Cell_Bank.htm)

hESC line:	**SI-216**
special features:	
published in a peer-reviewed journal:	no
characterization data reported in a peer-reviewed journal:	no
reference:	Stemride International Stem Cell Bank (http://www.stemride.com/Stem_Cell_Bank.htm)

hESC line:	**SI-217 (SC-217)**
special features:	
published in a peer-reviewed journal:	yes
characterization data reported in a peer-reviewed journal:	no
reference:	Stemride International Stem Cell Bank (http://www.stemride.com/Stem_Cell_Bank.htm)<br>Verlinsky, Y. et al. Reprod Biomed Online 18, 120-126 (2009)

hESC line:	**SI-218**
special features:	genetic disorder: Huntington's desease (HD)
published in a peer-reviewed journal:	yes
characterization data reported in a peer-reviewed journal:	no
reference:	Stemride International Stem Cell Bank (http://www.stemride.com/Stem_Cell_Bank.htm)<br>Verlinsky, Y. et al. Reprod Biomed Online 13, 547-550 (2006)

hESC line:	**SI-219**
special features:	
published in a peer-reviewed journal:	no
characterization data reported in a peer-reviewed journal:	no
reference:	Stemride International Stem Cell Bank (http://www.stemride.com/Stem_Cell_Bank.htm)

hESC line:	**SI-220 (SC-220)**
special features:	

published in a peer-reviewed journal:	yes
characterization data reported in a peer-reviewed journal:	no
reference:	Stemride International Stem Cell Bank (http://www.stemride.com/Stem_Cell_Bank.htm)<br>Verlinsky, Y. et al. Reprod Biomed Online 18, 120-126 (2009)

hESC line:	**SI-221 (SC-221)**
special features:	
published in a peer-reviewed journal:	yes
characterization data reported in a peer-reviewed journal:	no
reference:	Stemride International Stem Cell Bank (http://www.stemride.com/Stem_Cell_Bank.htm)<br>Verlinsky, Y. et al. Reprod Biomed Online 18, 120-126 (2009)

hESC line:	**SI-223**
special features:	
published in a peer-reviewed journal:	no
characterization data reported in a peer-reviewed journal:	no
reference:	Stemride International Stem Cell Bank (http://www.stemride.com/Stem_Cell_Bank.htm)

hESC line:	**SI-224**
special features:	
published in a peer-reviewed journal:	no
characterization data reported in a peer-reviewed journal:	no
reference:	Stemride International Stem Cell Bank (http://www.stemride.com/Stem_Cell_Bank.htm)

hESC line:	**SI-226**
special features:	
published in a peer-reviewed journal:	no
characterization data reported in a peer-reviewed journal:	no
reference:	Stemride International Stem Cell Bank (http://www.stemride.com/Stem_Cell_Bank.htm)

hESC line:	**SI-227**
special features:	

published in a peer-reviewed journal:	no
characterization data reported in a peer-reviewed journal:	no
reference:	Stemride International Stem Cell Bank (http://www.stemride.com/Stem_Cell_Bank.htm)

hESC line:	**SI-228**
special features:	
published in a peer-reviewed journal:	no
characterization data reported in a peer-reviewed journal:	no
reference:	Stemride International Stem Cell Bank (http://www.stemride.com/Stem_Cell_Bank.htm)

hESC line:	**SI-231**
special features:	
published in a peer-reviewed journal:	no
characterization data reported in a peer-reviewed journal:	no
reference:	Stemride International Stem Cell Bank (http://www.stemride.com/Stem_Cell_Bank.htm)

hESC line:	**SI-232**
special features:	
published in a peer-reviewed journal:	no
characterization data reported in a peer-reviewed journal:	no
reference:	Stemride International Stem Cell Bank (http://www.stemride.com/Stem_Cell_Bank.htm)

hESC line:	**SI-235**
special features:	genetic disorder: Neurofibromatosis type 1 (affected)
published in a peer-reviewed journal:	yes
characterization data reported in a peer-reviewed journal:	no
reference:	Stemride International Stem Cell Bank (http://www.stemride.com/Stem_Cell_Bank.htm)

hESC line:	**SI-236**
special features:	

published in a peer-reviewed journal:	no
characterization data reported in a peer-reviewed journal:	no
reference:	Stemride International Stem Cell Bank (http://www.stemride.com/Stem_Cell_Bank.htm)<br>Verlinsky, Y. et al. Reprod Biomed Online 13, 547-550 (2006)

hESC line:	**SI-237**
special features:	
published in a peer-reviewed journal:	no
characterization data reported in a peer-reviewed journal:	no
reference:	Stemride International Stem Cell Bank (http://www.stemride.com/Stem_Cell_Bank.htm)

hESC line:	**SI-238**
special features:	
published in a peer-reviewed journal:	no
characterization data reported in a peer-reviewed journal:	no
reference:	Stemride International Stem Cell Bank (http://www.stemride.com/Stem_Cell_Bank.htm)

hESC line:	**SI-239**
special features:	
published in a peer-reviewed journal:	no
characterization data reported in a peer-reviewed journal:	no
reference:	Stemride International Stem Cell Bank (http://www.stemride.com/Stem_Cell_Bank.htm)

hESC line:	**SI-24**
special features:	established from morula stage embryo
published in a peer-reviewed journal:	yes
characterization data reported in a peer-reviewed journal:	yes
reference:	Strelchenko, N. et al. Reprod Biomed Online 9, 623-629 (2004)<br>Stemride International Stem Cell Bank (http://www.stemride.com/Stem_Cell_Bank.htm)

hESC line:	**SI-240 (SC-240)**
special features:	

published in a peer-reviewed journal:	yes
characterization data reported in a peer-reviewed journal:	no
reference:	Stemride International Stem Cell Bank (http://www.stemride.com/Stem_Cell_Bank.htm)<br>Verlinsky, Y. et al. Reprod Biomed Online 18, 120-126 (2009)

hESC line:	**SI-241**
special features:	
published in a peer-reviewed journal:	no
characterization data reported in a peer-reviewed journal:	no
reference:	Stemride International Stem Cell Bank (http://www.stemride.com/Stem_Cell_Bank.htm)

hESC line:	**SI-242**
special features:	
published in a peer-reviewed journal:	no
characterization data reported in a peer-reviewed journal:	no
reference:	Stemride International Stem Cell Bank (http://www.stemride.com/Stem_Cell_Bank.htm)

hESC line:	**SI-243 (SC-243)**
special features:	
published in a peer-reviewed journal:	yes
characterization data reported in a peer-reviewed journal:	no
reference:	Stemride International Stem Cell Bank (http://www.stemride.com/Stem_Cell_Bank.htm)<br>Verlinsky, Y. et al. Reprod Biomed Online 18, 120-126 (2009)

hESC line:	**SI-244**
special features:	
published in a peer-reviewed journal:	no
characterization data reported in a peer-reviewed journal:	no
reference:	Stemride International Stem Cell Bank (http://www.stemride.com/Stem_Cell_Bank.htm)

hESC line:	**SI-245 (SC-245, hESC-245, RG-245)**
special features:	abnormal karyotype<br>genetic disorder: Muscular dystrophy, type Emery Dreifuss (carrier)
published in a peer-reviewed journal:	yes
characterization data reported in a peer-reviewed journal:	no
reference:	Stemride International Stem Cell Bank (http://www.stemride.com/Stem_Cell_Bank.htm)<br>Verlinsky, Y. et al. Reprod Biomed Online 13, 547-550 (2006)

hESC line:	**SI-247**
special features:	
published in a peer-reviewed journal:	no
characterization data reported in a peer-reviewed journal:	no
reference:	Stemride International Stem Cell Bank (http://www.stemride.com/Stem_Cell_Bank.htm)

hESC line:	**SI-248**
special features:	
published in a peer-reviewed journal:	no
characterization data reported in a peer-reviewed journal:	no
reference:	Stemride International Stem Cell Bank (http://www.stemride.com/Stem_Cell_Bank.htm)

hESC line:	**SI-250**
special features:	
published in a peer-reviewed journal:	no
characterization data reported in a peer-reviewed journal:	no
reference:	Stemride International Stem Cell Bank (http://www.stemride.com/Stem_Cell_Bank.htm)

hESC line:	**SI-251**
special features:	
published in a peer-reviewed journal:	no
characterization data reported in a peer-reviewed journal:	no
reference:	Stemride International Stem Cell Bank (http://www.stemride.com/Stem_Cell_Bank.htm)

hESC line:	**SI-252 (hESC-252)**
special features:	abnormal karyotype
published in a peer-reviewed journal:	yes
characterization data reported in a peer-reviewed journal:	no
reference:	Stemride International Stem Cell Bank (http://www.stemride.com/Stem_Cell_Bank.htm)<br>Verlinsky, Y. et al. Reprod Biomed Online 13, 547-550 (2006)

hESC line:	**SI-253 (SC-253)**
special features:	
published in a peer-reviewed journal:	yes
characterization data reported in a peer-reviewed journal:	no
reference:	Stemride International Stem Cell Bank (http://www.stemride.com/Stem_Cell_Bank.htm)<br>Verlinsky, Y. et al. Reprod Biomed Online 18, 120-126 (2009)

hESC line:	**SI-254**
special features:	genetic disorder: Sickle cell anaemia
published in a peer-reviewed journal:	no
characterization data reported in a peer-reviewed journal:	no
reference:	Stemride International Stem Cell Bank (http://www.stemride.com/Stem_Cell_Bank.htm)

hESC line:	**SI-255**
special features:	
published in a peer-reviewed journal:	no
characterization data reported in a peer-reviewed journal:	no
reference:	Stemride International Stem Cell Bank (http://www.stemride.com/Stem_Cell_Bank.htm)

hESC line:	**SI-256**
special features:	
published in a peer-reviewed journal:	no
characterization data reported in a peer-reviewed journal:	no
reference:	Stemride International Stem Cell Bank (http://www.stemride.com/Stem_Cell_Bank.htm)

hESC line:	**SI-257 (hESC-257)**
special features:	genetic disorder: Cystic fibrosis (CF)
published in a peer-reviewed journal:	yes
characterization data reported in a peer-reviewed journal:	no
reference:	Stemride International Stem Cell Bank (http://www.stemride.com/Stem_Cell_Bank.htm)<br>Verlinsky, Y. et al. Reprod Biomed Online 13, 547-550 (2006)

hESC line:	**SI-267 (SC-267)**
special features:	abnormal karyotype
published in a peer-reviewed journal:	yes
characterization data reported in a peer-reviewed journal:	no
reference:	Verlinsky, Y. et al. Reprod Biomed Online 18, 120-126 (2009)

hESC line:	**SI-269 (SC-269)**
special features:	
published in a peer-reviewed journal:	yes
characterization data reported in a peer-reviewed journal:	no
reference:	Verlinsky, Y. et al. Reprod Biomed Online 18, 120-126 (2009)

hESC line:	**SI-27**
special features:	established from morula stage embryo
published in a peer-reviewed journal:	yes
characterization data reported in a peer-reviewed journal:	yes
reference:	Strelchenko, N. et al. Reprod Biomed Online 9, 623-629 (2004)<br>Stemride International Stem Cell Bank (http://www.stemride.com/Stem_Cell_Bank.htm)

hESC line:	**SI-270 (SC-270)**
special features:	abnormal karyotype
published in a peer-reviewed journal:	yes
characterization data reported in a peer-reviewed journal:	no
reference:	Verlinsky, Y. et al. Reprod Biomed Online 18, 120-126 (2009)

hESC line:	**SI-274**
special features:	genetic disorder: Spinal muscular atrophy type 1 (SMA1)
published in a peer-reviewed journal:	yes
characterization data reported in a peer-reviewed journal:	no
reference:	Stemride International Stem Cell Bank (http://www.stemride.com/Stem_Cell_Bank.htm)<br>Verlinsky, Y. et al. Reprod Biomed Online 13, 547-550 (2006)

hESC line:	**SI-277**
special features:	genetic disorder: Sickle cell anaemia
published in a peer-reviewed journal:	yes
characterization data reported in a peer-reviewed journal:	no
reference:	Stemride International Stem Cell Bank (http://www.stemride.com/Stem_Cell_Bank.htm)<br>Verlinsky, Y. et al. Reprod Biomed Online 13, 547-550 (2006)

hESC line:	**SI-278 (hESC-278)**
special features:	abnormal karyotype
published in a peer-reviewed journal:	no
characterization data reported in a peer-reviewed journal:	no
reference:	Stemride Internatioinal Presentation "hESC Lines with Genetic and chromosomal disorders" , October 2008 (http://stemride.com/news.htm)

hESC line:	**SI-279**
special features:	genetic disorder: Ocular albinism
published in a peer-reviewed journal:	yes
characterization data reported in a peer-reviewed journal:	no
reference:	Stemride International Stem Cell Bank (http://www.stemride.com/Stem_Cell_Bank.htm)<br>Verlinsky, Y. et al. Reprod Biomed Online 13, 547-550 (2006)

hESC line:	**SI-28**
special features:	established from morula stage embryo

published in a peer-reviewed journal:	yes
characterization data reported in a peer-reviewed journal:	yes
reference:	Strelchenko, N. et al. Reprod Biomed Online 9, 623-629 (2004)<br>Stemride International Stem Cell Bank (http://www.stemride.com/Stem_Cell_Bank.htm)

hESC line:	**SI-284 (SC-284)**
special features:	
published in a peer-reviewed journal:	yes
characterization data reported in a peer-reviewed journal:	no
reference:	Verlinsky, Y. et al. Reprod Biomed Online 18, 120-126 (2009)

hESC line:	**SI-285 (SC-285)**
special features:	genetic disorder: Ocular albinism
published in a peer-reviewed journal:	yes
characterization data reported in a peer-reviewed journal:	no
reference:	Stemride International Stem Cell Bank (http://www.stemride.com/Stem_Cell_Bank.htm)<br>Verlinsky, Y. et al. Reprod Biomed Online 18, 120-126 (2009)

hESC line:	**SI-294 (SC-294)**
special features:	
published in a peer-reviewed journal:	yes
characterization data reported in a peer-reviewed journal:	no
reference:	Verlinsky, Y. et al. Reprod Biomed Online 18, 120-126 (2009)

hESC line:	**SI-295 (SC-295)**
special features:	
published in a peer-reviewed journal:	yes
characterization data reported in a peer-reviewed journal:	no
reference:	Verlinsky, Y. et al. Reprod Biomed Online 18, 120-126 (2009)

hESC line:	**SI-297 (SC-297)**
special features:	

published in a peer-reviewed journal:	yes
characterization data reported in a peer-reviewed journal:	no
reference:	Verlinsky, Y. et al. Reprod Biomed Online 18, 120-126 (2009)

hESC line:	**SI-300 (SC-300, hESC-300)**
special features:	abnormal karyotype
published in a peer-reviewed journal:	yes
characterization data reported in a peer-reviewed journal:	no
reference:	Verlinsky, Y. et al. Reprod Biomed Online 18, 120-126 (2009)

hESC line:	**SI-302 (SC-302, RG-302)**
special features:	genetic disorder: Muscular dystrophy, type Duchenne (carrirer)
published in a peer-reviewed journal:	yes
characterization data reported in a peer-reviewed journal:	no
reference:	Stemride International Stem Cell Bank (http://www.stemride.com/Stem_Cell_Bank.htm)<br>Verlinsky, Y. et al. Reprod Biomed Online 18, 120-126 (2009)

hESC line:	**SI-31**
special features:	established from morula stage embryo
published in a peer-reviewed journal:	yes
characterization data reported in a peer-reviewed journal:	yes
reference:	Strelchenko, N. et al. Reprod Biomed Online 9, 623-629 (2004)<br>Stemride International Stem Cell Bank (http://www.stemride.com/Stem_Cell_Bank.htm)

hESC line:	**SI-310 (hESC-310)**
special features:	abnormal karyotype
published in a peer-reviewed journal:	no
characterization data reported in a peer-reviewed journal:	no
reference:	Stemride Internatioinal Presentation "hESC Lines with Genetic and chromosomal disorders" , October 2008 (http://stemride.com/news.htm)

hESC line:	**SI-321 (SC-321)**
special features:	abnormal karyotype

published in a peer-reviewed journal:	yes
characterization data reported in a peer-reviewed journal:	no
reference:	Verlinsky, Y. et al. Reprod Biomed Online 18, 120-126 (2009)<br>Stemride Internatioinal Presentation "hESC Lines with Genetic and chromosomal disorders" , October 2008 (http://stemride.com/news.htm)

hESC line:	**SI-323**
special features:	genetic disorder: Sandhoff disease (affected)
published in a peer-reviewed journal:	no
characterization data reported in a peer-reviewed journal:	no
reference:	Stemride International Stem Cell Bank (http://www.stemride.com/Stem_Cell_Bank.htm)

hESC line:	**SI-324**
special features:	genetic disorder: Sandhoff disease (affected)
published in a peer-reviewed journal:	no
characterization data reported in a peer-reviewed journal:	no
reference:	Stemride International Stem Cell Bank (http://www.stemride.com/Stem_Cell_Bank.htm)

hESC line:	**SI-325 (SC-325)**
special features:	
published in a peer-reviewed journal:	yes
characterization data reported in a peer-reviewed journal:	no
reference:	Verlinsky, Y. et al. Reprod Biomed Online 18, 120-126 (2009)

hESC line:	**SI-327 (SC-327)**
special features:	
published in a peer-reviewed journal:	yes
characterization data reported in a peer-reviewed journal:	no
reference:	Verlinsky, Y. et al. Reprod Biomed Online 18, 120-126 (2009)

hESC line:	**SI-329**
special features:	genetic disorder: Facio Scapulo Humeral (FSH) muscular dystrophy

published in a peer-reviewed journal:	no
characterization data reported in a peer-reviewed journal:	no
reference:	Stemride International Stem Cell Bank (http://www.stemride.com/Stem_Cell_Bank.htm)

hESC line:	**SI-33**
special features:	established from morula stage embryo
published in a peer-reviewed journal:	yes
characterization data reported in a peer-reviewed journal:	yes
reference:	Strelchenko, N. et al. Reprod Biomed Online 9, 623-629 (2004)<br>Stemride International Stem Cell Bank (http://www.stemride.com/Stem_Cell_Bank.htm)

hESC line:	**SI-331 (SC-331, hESC-331)**
special features:	
published in a peer-reviewed journal:	yes
characterization data reported in a peer-reviewed journal:	no
reference:	Verlinsky, Y. et al. Reprod Biomed Online 18, 120-126 (2009)

hESC line:	**SI-334 (SC-334)**
special features:	
published in a peer-reviewed journal:	no
characterization data reported in a peer-reviewed journal:	no
reference:	Verlinsky, Y. et al. Reprod Biomed Online 18, 120-126 (2009)

hESC line:	**SI-335 (SC-335)**
special features:	
published in a peer-reviewed journal:	yes
characterization data reported in a peer-reviewed journal:	no
reference:	Verlinsky, Y. et al. Reprod Biomed Online 18, 120-126 (2009)

hESC line:	**SI-336 (SC-336)**
special features:	

published in a peer-reviewed journal:	yes
characterization data reported in a peer-reviewed journal:	no
reference:	Verlinsky, Y. et al. Reprod Biomed Online 18, 120-126 (2009)

hESC line:	**SI-337 (SC-337)**
special features:	
published in a peer-reviewed journal:	yes
characterization data reported in a peer-reviewed journal:	no
reference:	Verlinsky, Y. et al. Reprod Biomed Online 18, 120-126 (2009)

hESC line:	**SI-338 (SC-338)**
special features:	
published in a peer-reviewed journal:	yes
characterization data reported in a peer-reviewed journal:	no
reference:	Verlinsky, Y. et al. Reprod Biomed Online 18, 120-126 (2009)

hESC line:	**SI-339 (hESC-339)**
special features:	abnormal karyotype
published in a peer-reviewed journal:	no
characterization data reported in a peer-reviewed journal:	no
reference:	Stemride Internatioinal Presentation "hESC Lines with Genetic and chromosomal disorders" , October 2008 (http://stemride.com/news.htm)

hESC line:	**SI-340 (SC-340)**
special features:	
published in a peer-reviewed journal:	yes
characterization data reported in a peer-reviewed journal:	no
reference:	Verlinsky, Y. et al. Reprod Biomed Online 18, 120-126 (2009)

hESC line:	**SI-342 (SC-342)**
special features:	

published in a peer-reviewed journal:	yes
characterization data reported in a peer-reviewed journal:	no
reference:	Verlinsky, Y. et al. Reprod Biomed Online 18, 120-126 (2009)

hESC line:	**SI-343 (SC-343)**
special features:	
published in a peer-reviewed journal:	yes
characterization data reported in a peer-reviewed journal:	no
reference:	Verlinsky, Y. et al. Reprod Biomed Online 18, 120-126 (2009)

hESC line:	**SI-345 (SC-345)**
special features:	
published in a peer-reviewed journal:	yes
characterization data reported in a peer-reviewed journal:	no
reference:	Verlinsky, Y. et al. Reprod Biomed Online 10, 105-110 (2005)

hESC line:	**SI-354 (SC-354)**
special features:	
published in a peer-reviewed journal:	yes
characterization data reported in a peer-reviewed journal:	no
reference:	Verlinsky, Y. et al. Reprod Biomed Online 18, 120-126 (2009)

hESC line:	**SI-359 (hESC-359)**
special features:	abnormal karyotype
published in a peer-reviewed journal:	no
characterization data reported in a peer-reviewed journal:	no
reference:	Stemride Internatioinal Presentation "hESC Lines with Genetic and chromosomal disorders" , October 2008 (http://stemride.com/news.htm)

hESC line:	**SI-395**
special features:	genetic disorder: Sandhoff disease (affected)

published in a peer-reviewed journal:	no
characterization data reported in a peer-reviewed journal:	no
reference:	Stemride International Stem Cell Bank (http://www.stemride.com/Stem_Cell_Bank.htm)

hESC line:	**SI-396**
special features:	genetic disorder: Huntington's desease (HD)
published in a peer-reviewed journal:	no
characterization data reported in a peer-reviewed journal:	no
reference:	Stemride International Stem Cell Bank (http://www.stemride.com/Stem_Cell_Bank.htm)

hESC line:	**SI-397**
special features:	genetic disorder: Huntington's desease (HD)
published in a peer-reviewed journal:	no
characterization data reported in a peer-reviewed journal:	no
reference:	Stemride International Stem Cell Bank (http://www.stemride.com/Stem_Cell_Bank.htm)

hESC line:	**SI-405**
special features:	genetic disorder: beta-Thalassaemia (carrier)
published in a peer-reviewed journal:	no
characterization data reported in a peer-reviewed journal:	no
reference:	Stemride International Stem Cell Bank (http://www.stemride.com/Stem_Cell_Bank.htm)

hESC line:	**SI-53**
special features:	derived by whole embryo culture
published in a peer-reviewed journal:	yes
characterization data reported in a peer-reviewed journal:	yes
reference:	Strelchenko, N. et al. Reprod Biomed Online 9, 623-629 (2004)<br>Stemride International Stem Cell Bank (http://www.stemride.com/Stem_Cell_Bank.htm)

hESC line:	**SI-60**
special features:	derived by whole embryo culture

published in a peer-reviewed journal:	yes
characterization data reported in a peer-reviewed journal:	yes
reference:	Strelchenko, N. et al. Reprod Biomed Online 9, 623-629 (2004)<br>Stemride International Stem Cell Bank (http://www.stemride.com/Stem_Cell_Bank.htm)

hESC line:	**SI-62**
special features:	derived by whole embryo culture
published in a peer-reviewed journal:	yes
characterization data reported in a peer-reviewed journal:	yes
reference:	Strelchenko, N. et al. Reprod Biomed Online 9, 623-629 (2004)<br>Stemride International Stem Cell Bank (http://www.stemride.com/Stem_Cell_Bank.htm)

hESC line:	**SI-63**
special features:	derived by whole embryo culture
published in a peer-reviewed journal:	yes
characterization data reported in a peer-reviewed journal:	yes
reference:	Strelchenko, N. et al. Reprod Biomed Online 9, 623-629 (2004)<br>Stemride International Stem Cell Bank (http://www.stemride.com/Stem_Cell_Bank.htm)

hESC line:	**SI-79**
special features:	derived by whole embryo culture
published in a peer-reviewed journal:	yes
characterization data reported in a peer-reviewed journal:	yes
reference:	Strelchenko, N. et al. Reprod Biomed Online 9, 623-629 (2004)<br>Stemride International Stem Cell Bank (http://www.stemride.com/Stem_Cell_Bank.htm)

hESC line:	**SI-80**
special features:	derived by whole embryo culture

published in a peer-reviewed journal:	yes
characterization data reported in a peer-reviewed journal:	yes
reference:	Strelchenko, N. et al. Reprod Biomed Online 9, 623-629 (2004)<br>Stemride International Stem Cell Bank (http://www.stemride.com/Stem_Cell_Bank.htm)

hESC line:	**SI-81**
special features:	derived by whole embryo culture
published in a peer-reviewed journal:	yes
characterization data reported in a peer-reviewed journal:	yes
reference:	Strelchenko, N. et al. Reprod Biomed Online 9, 623-629 (2004)<br>Stemride International Stem Cell Bank (http://www.stemride.com/Stem_Cell_Bank.htm)

hESC line:	**SI-93**
special features:	
published in a peer-reviewed journal:	yes
characterization data reported in a peer-reviewed journal:	yes
reference:	Strelchenko, N. et al. Reprod Biomed Online 9, 623-629 (2004)<br>Stemride International Stem Cell Bank (http://www.stemride.com/Stem_Cell_Bank.htm)

hESC line:	**SI-94**
special features:	
published in a peer-reviewed journal:	yes
characterization data reported in a peer-reviewed journal:	yes
reference:	Strelchenko, N. et al. Reprod Biomed Online 9, 623-629 (2004)<br>Stemride International Stem Cell Bank (http://www.stemride.com/Stem_Cell_Bank.htm)

hESC line:	**SI-95**
special features:	

published in a peer-reviewed journal:	yes
characterization data reported in a peer-reviewed journal:	yes
reference:	Strelchenko, N. et al. Reprod Biomed Online 9, 623-629 (2004)<br>Stemride International Stem Cell Bank (http://www.stemride.com/Stem_Cell_Bank.htm)

hESC line:	**SI-96**
special features:	
published in a peer-reviewed journal:	yes
characterization data reported in a peer-reviewed journal:	yes
reference:	Strelchenko, N. et al. Reprod Biomed Online 9, 623-629 (2004)<br>Stemride International Stem Cell Bank (http://www.stemride.com/Stem_Cell_Bank.htm)

hESC line:	**SI-97**
special features:	
published in a peer-reviewed journal:	yes
characterization data reported in a peer-reviewed journal:	yes
reference:	Strelchenko, N. et al. Reprod Biomed Online 9, 623-629 (2004)<br>Stemride International Stem Cell Bank (http://www.stemride.com/Stem_Cell_Bank.htm)

hESC line:	**SI-98**
special features:	
published in a peer-reviewed journal:	no
characterization data reported in a peer-reviewed journal:	no
reference:	Stemride International Stem Cell Bank (http://www.stemride.com/Stem_Cell_Bank.htm)

hESC line:	**SI-99**
special features:	
published in a peer-reviewed journal:	no
characterization data reported in a peer-reviewed journal:	no
reference:	Stemride International Stem Cell Bank (http://www.stemride.com/Stem_Cell_Bank.htm)

Provider: Reprogenetics, LCC, San Francisco, CA

hESC line:	**RG7**
special features:	
published in a peer-reviewed journal:	no
characterization data reported in a peer-reviewed journal:	no
reference:	NIH Human Embryonic Stem Cell Registry (http://grants.nih.gov/stem_cells/registry/current.htm)

Provider: Rockefeller University

hESC line:	**RUES1**
special features:	derived in serum free medium
published in a peer-reviewed journal:	yes
characterization data reported in a peer-reviewed journal:	yes
reference:	James, D. et al Dev Biol 295, 90-102 (2006)

hESC line:	**RUES2**
special features:	
published in a peer-reviewed journal:	no
characterization data reported in a peer-reviewed journal:	no
reference:	Noggle, S. A. et al., Human Embryonic Stem Cells. The Practical Handbook, Wiley & Sons, 123-147 (2007)

hESC line:	**RUES3**
special features:	
published in a peer-reviewed journal:	no
characterization data reported in a peer-reviewed journal:	no
reference:	JDRF-Funded Research, Brivanlou Project (http://onlineapps.jdfcure.org/AbstractReport.cfm?grant_id=14798&abs_type=FPR)

Provider: StemLifeLine, Inc., San Carlos, California, USA

hESC line:	**R5.3**
special features:	derived by whole embryo culture<br>derived in serum free medium<br>derived on human feeder cells

published in a peer-reviewed journal:	yes
characterization data reported in a peer-reviewed journal:	yes
reference:	Ilic, D. et al. Stem Cells Dev, published online ahead of print June 1st 2009

hESC line:	**R5.5**
special features:	derived by whole embryo culture<br>derived in serum free medium<br>derived on human feeder cells
published in a peer-reviewed journal:	yes
characterization data reported in a peer-reviewed journal:	yes
reference:	Ilic, D. et al. Stem Cells Dev, published online ahead of print June 1st 2009

hESC line:	**W10-1A**
special features:	derived from single blastomere<br>derived on human feeder cells<br>mechanical ICM isolation
published in a peer-reviewed journal:	yes
characterization data reported in a peer-reviewed journal:	yes
reference:	Ilic, D. et al. Stem Cells Dev, published online ahead of print February 17th 2009

hESC line:	**W13-1C**
special features:	derived from single blastomere<br>derived on human feeder cells<br>mechanical ICM isolation
published in a peer-reviewed journal:	yes
characterization data reported in a peer-reviewed journal:	yes
reference:	Ilic, D. et al. Stem Cells Dev, published online ahead of print February 17th 2009

hESC line:	**W14-1A**
special features:	derived from single blastomere<br>derived on human feeder cells<br>mechanical ICM isolation

published in a peer-reviewed journal:	yes
characterization data reported in a peer-reviewed journal:	yes
reference:	Ilic, D. et al. Stem Cells Dev, published online ahead of print February 17th 2009

hESC line:	**W8-8A**
special features:	derived from single blastomere<br>derived on human feeder cells<br>mechanical ICM isolation
published in a peer-reviewed journal:	yes
characterization data reported in a peer-reviewed journal:	yes
reference:	Ilic, D. et al. Stem Cells Dev, published online ahead of print February 17th 2009

Provider: University of California, San Francisco

hESC line:	**HSF-1**
special features:	
published in a peer-reviewed journal:	yes
characterization data reported in a peer-reviewed journal:	yes
reference:	NIH Human Embryonic Stem Cell Registry (http://grants.nih.gov/stem_cells/registry/current.htm)

hESC line:	**HSF-10**
special features:	derived by whole embryo culture<br>derived in serum free medium<br>derived on human feeder cells
published in a peer-reviewed journal:	yes
characterization data reported in a peer-reviewed journal:	yes
reference:	Chavez, S. L. et al. Stem Cells Dev 17, 535-546 (2008)

hESC line:	**HSF-12**
special features:	derived by whole embryo culture<br>derived in serum free medium<br>derived on human feeder cells

published in a peer-reviewed journal:	yes
characterization data reported in a peer-reviewed journal:	yes
reference:	Chavez, S. L. et al. Stem Cells Dev 17, 535-546 (2008)

---

hESC line:	**HSF-13**
special features:	derived by whole embryo culture<br>derived in serum free medium<br>derived on human feeder cells
published in a peer-reviewed journal:	yes
characterization data reported in a peer-reviewed journal:	yes
reference:	Chavez, S. L. et al. Stem Cells Dev 17, 535-546 (2008)

---

hESC line:	**HSF-6**
special features:	
published in a peer-reviewed journal:	yes
characterization data reported in a peer-reviewed journal:	yes
reference:	NIH Human Embryonic Stem Cell Registry (http://grants.nih.gov/stem_cells/registry/current.htm)

---

hESC line:	**HSF-7**
special features:	derived by whole embryo culture<br>derived in serum free medium<br>derived on human feeder cells
published in a peer-reviewed journal:	yes
characterization data reported in a peer-reviewed journal:	yes
reference:	Chavez, S. L. et al. Stem Cells Dev 17, 535-546 (2008)

---

hESC line:	**HSF-8**
special features:	derived by whole embryo culture<br>derived in serum free medium<br>derived on human feeder cells
published in a peer-reviewed journal:	yes
characterization data reported in a peer-reviewed journal:	yes
reference:	Chavez, S. L. et al. Stem Cells Dev 17, 535-546 (2008)

---

hESC line:	**HSF-9**
special features:	derived by whole embryo culture<br>derived in serum free medium<br>derived on human feeder cells
published in a peer-reviewed journal:	yes
characterization data reported in a peer-reviewed journal:	yes
reference:	Chavez, S. L. et al. Stem Cells Dev 17, 535-546 (2008)

hESC line:	**UCSF-1**
special features:	derived by whole embryo culture<br>derived in serum free medium<br>derived on human feeder cells
published in a peer-reviewed journal:	yes
characterization data reported in a peer-reviewed journal:	yes
reference:	Genbacev, O. et al. Fertil Steril 83, 1517-1529 (2005)

hESC line:	**UCSF-2**
special features:	derived by whole embryo culture<br>derived in serum free medium<br>derived on human feeder cells
published in a peer-reviewed journal:	yes
characterization data reported in a peer-reviewed journal:	yes
reference:	Genbacev, O. et al. Fertil Steril 83, 1517-1529 (2005)

hESC line:	**UCSF-3**
special features:	
published in a peer-reviewed journal:	no
characterization data reported in a peer-reviewed journal:	no
reference:	NIH Human Embryonic Stem Cell Registry (http://grants.nih.gov/stem_cells/registry/current.htm)<br>California Institue of Regenerative Medicine - List of acceptably derived embryonic stem cell lines (http://www.cirm.ca.gov/CIRMCellLines)

hESC line:	**UCSF-4**
special features:	

published in a peer-reviewed journal:	no
characterization data reported in a peer-reviewed journal:	no
reference:	California Institue of Regenerative Medicine - List of acceptably derived embryonic stem cell lines (http://www.cirm.ca.gov/CIRMCellLines)

hESC line:	**UCSFB-1**
special features:	
published in a peer-reviewed journal:	no
characterization data reported in a peer-reviewed journal:	no
reference:	NIH Human Embryonic Stem Cell Registry (http://grants.nih.gov/stem_cells/registry/current.htm)

hESC line:	**UCSFB-10**
special features:	
published in a peer-reviewed journal:	no
characterization data reported in a peer-reviewed journal:	no
reference:	NIH Human Embryonic Stem Cell Registry (http://grants.nih.gov/stem_cells/registry/current.htm)

hESC line:	**UCSFB-2**
special features:	
published in a peer-reviewed journal:	no
characterization data reported in a peer-reviewed journal:	no
reference:	NIH Human Embryonic Stem Cell Registry (http://grants.nih.gov/stem_cells/registry/current.htm)

hESC line:	**UCSFB-3**
special features:	
published in a peer-reviewed journal:	no
characterization data reported in a peer-reviewed journal:	no
reference:	NIH Human Embryonic Stem Cell Registry (http://grants.nih.gov/stem_cells/registry/current.htm)

hESC line:	**UCSFB-4**
special features:	

published in a peer-reviewed journal:	no
characterization data reported in a peer-reviewed journal:	no
reference:	NIH Human Embryonic Stem Cell Registry (http://grants.nih.gov/stem_cells/registry/current.htm)

hESC line:	**UCSFB-5**
special features:	
published in a peer-reviewed journal:	no
characterization data reported in a peer-reviewed journal:	no
reference:	NIH Human Embryonic Stem Cell Registry (http://grants.nih.gov/stem_cells/registry/current.htm)

hESC line:	**UCSFB-6**
special features:	
published in a peer-reviewed journal:	no
characterization data reported in a peer-reviewed journal:	no
reference:	NIH Human Embryonic Stem Cell Registry (http://grants.nih.gov/stem_cells/registry/current.htm)

hESC line:	**UCSFB-7**
special features:	
published in a peer-reviewed journal:	no
characterization data reported in a peer-reviewed journal:	no
reference:	NIH Human Embryonic Stem Cell Registry (http://grants.nih.gov/stem_cells/registry/current.htm)

hESC line:	**UCSFB-8**
special features:	
published in a peer-reviewed journal:	no
characterization data reported in a peer-reviewed journal:	no
reference:	NIH Human Embryonic Stem Cell Registry (http://grants.nih.gov/stem_cells/registry/current.htm)

hESC line:	**UCSFB-9**
special features:	

published in a peer-reviewed journal:	no
characterization data reported in a peer-reviewed journal:	no
reference:	NIH Human Embryonic Stem Cell Registry (http://grants.nih.gov/stem_cells/registry/current.htm)

Provider: University of Connecticut Health Center

hESC line:	**CT1**
special features:	
published in a peer-reviewed journal:	no
characterization data reported in a peer-reviewed journal:	no
reference:	UMA-Stem Cell Registry (http://www.umassmed.edu/iscr/index.aspx)<br>University of Connecticut, Press release, 2009/01/28 (http://news.uconn.edu/2009/January/rel09008.html)

hESC line:	**CT2**
special features:	
published in a peer-reviewed journal:	no
characterization data reported in a peer-reviewed journal:	no
reference:	UMA-Stem Cell Registry (http://www.umassmed.edu/iscr/index.aspx)<br>University of Connecticut, Press release, 2009/01/28 (http://news.uconn.edu/2009/January/rel09008.html)

Provider: WiCell Research Institute

hESC line:	**H1**
special features:	
published in a peer-reviewed journal:	yes
characterization data reported in a peer-reviewed journal:	yes
reference:	Thomson, J. A. et al. Science 282, 1145-1147 (1998)<br>NIH Human Embryonic Stem Cell Registry (http://grants.nih.gov/stem_cells/registry/current.htm)

hESC line:	**H1.1**
special features:	

published in a peer-reviewed journal:	
characterization data reported in a peer-reviewed journal:	derivative
reference:	Odorico, J. S. et al. Stem Cells 19, 193-204 (2001)<br>Kaufman, D. S. et al. Proc Natl Acad Sci U S A 98, 10716-10721 (2001)

hESC line:	**H13**
special features:	
published in a peer-reviewed journal:	yes
characterization data reported in a peer-reviewed journal:	yes
reference:	Thomson, J. A. et al. Science 282, 1145-1147 (1998)<br>NIH Human Embryonic Stem Cell Registry (http://grants.nih.gov/stem_cells/registry/current.htm)

hESC line:	**H14**
special features:	
published in a peer-reviewed journal:	yes
characterization data reported in a peer-reviewed journal:	yes
reference:	Thomson, J. A. et al. Science 282, 1145-1147 (1998)<br>NIH Human Embryonic Stem Cell Registry (http://grants.nih.gov/stem_cells/registry/current.htm)

hESC line:	**H7**
special features:	
published in a peer-reviewed journal:	yes
characterization data reported in a peer-reviewed journal:	yes
reference:	Thomson, J. A. et al. Science 282, 1145-1147 (1998)<br>NIH Human Embryonic Stem Cell Registry (http://grants.nih.gov/stem_cells/registry/current.htm)

hESC line:	**H9**
special features:	

published in a peer-reviewed journal:	yes
characterization data reported in a peer-reviewed journal:	yes
reference:	Thomson, J. A. et al. Science 282, 1145-1147 (1998)<br>NIH Human Embryonic Stem Cell Registry (http://grants.nih.gov/stem_cells/registry/current.htm)

hESC line:	**WA15**
special features:	derived in animal-free defined media<br>derived without feeder cells
published in a peer-reviewed journal:	yes
characterization data reported in a peer-reviewed journal:	yes
reference:	Ludwig, T. E. et al. Nat Biotech 24, 185-187 (2006)

hESC line:	**WA16**
special features:	abnormal karyotype<br>derived in animal-free defined media<br>derived without feeder cells
published in a peer-reviewed journal:	yes
characterization data reported in a peer-reviewed journal:	yes
reference:	Ludwig, T. E. et al. Nat Biotech 24, 185-187 (2006)
