# Supplementary material for: Human Embryonic Stem Cell Lines and Their Use in International Research
Source: Stem Cells. 2010 Feb;28(2):240–6. doi: 10.1002/stem.286 (PMC2952289; doi:10.1002/stem.286)
Supplement: Supplementary file 5 [file stem0028-0240-SD5.pdf]

Research Papers Reporting Original hESC Work

1998 Thomson, J. A. et al., Science 282, 1145-1147 (1998)
http://dx.doi.org/10.1126/science.282.5391.1145

2000 Amit, M. et al., Dev Biol 227, 271-278 (2000)
http://dx.doi.org/10.1006/dbio.2000.9912

Itskovitz-Eldor, J. et al., Mol Med 6, 88-95 (2000)
http://www.molmed.org/content/2000/2_00.html

Reubinoff, B. E. et al., Nat Biotechnol 18, 399-404 (2000)
http://dx.doi.org/10.1038/74447

Schuldiner, M. et al., Proc Natl Acad Sci U S A 97, 11307-11312 (2000)
http://dx.doi.org/10.1073/pnas.97.21.11307

Tzukerman, M. et al., Mol Biol Cell 11, 4381-4391 (2000)
http://www.molbiolcell.org/cgi/content/abstract/11/12/4381

2001 Assady, S. et al., Diabetes 50, 1691-1697 (2001)
http://dx.doi.org/10.2337/diabetes.50.8.1691

Carpenter, M. K. et al., Exp Neurol 172, 383-397 (2001)
http://dx.doi.org/10.1006/exnr.2001.7832

Eiges, R. et al., Curr Biol 11, 514-518 (2001)
http://dx.doi.org/10.1016/S0960-9822(01)00144-0

Kaufman, D. S. et al., Proc Natl Acad Sci U S A 98, 10716-10721 (2001)
http://dx.doi.org/10.1073/pnas.191362598

Kehat, I. et al., J Clin Invest 108, 407-414 (2001)
http://dx.doi.org/10.1172/JCI200112131

Lanzendorf, S. E. et al., Fertil Steril 76, 132-137 (2001)
http://dx.doi.org/10.1016/S0015-0282(01)01825-8

Reubinoff, B. E. et al., Hum Reprod 16, 2187-2194 (2001)
http://humrep.oxfordjournals.org/cgi/content/abstract/16/10/2187

Reubinoff, B. E. et al., Nat Biotechnol 19, 1134-1140 (2001)
http://dx.doi.org/10.1038/nbt1201-1134

Schuldiner, M. et al., Brain Res 913, 201-205 (2001)
http://dx.doi.org/10.1016/S0006-8993(01)02776-7

Xu, C. H. et al., Nat Biotechnol 19, 971-974 (2001)
http://dx.doi.org/10.1038/nbt1001-971

Zhang, S. C. et al., Nat Biotechnol 19, 1129-1133 (2001)
http://dx.doi.org/10.1038/nbt1201-1129

2002 Amit, M. & Itskovitz-Eldor, J., J Anat 200, 225-232 (2002)
http://dx.doi.org/10.1186/1471-2121-10-44

Andrews, P. W., Philos Trans R Soc Lond B Biol Sci 357, 405-417 (2002)
http://dx.doi.org/10.1098/rstb.2002.1058

Cooper, S. et al., J Anat 200, 259-265 (2002)
http://dx.doi.org/10.1046/j.1469-7580.2002.00034.x

Draper, J. S. et al., J Anat 200, 249-258 (2002)
http://dx.doi.org/10.1046/j.1469-7580.2002.00030.x

Drukker, M. et al., Proc Natl Acad Sci U S A 99, 9864-9869 (2002)
http://dx.doi.org/10.1073/pnas.142298299

Goldstein, R. S. et al., Dev Dynam 225, 80-86 (2002)
http://dx.doi.org/10.1002/dvdy.10108

Henderson, J. K. et al., Stem Cells 20, 329-337 (2002)
http://dx.doi.org/10.1634/stemcells.20-4-329

Kehat, I. et al., Circ Res 91, 659-661 (2002)
http://dx.doi.org/10.1161/01.RES.0000039084.30342.9B

Levenberg, S. et al., Proc Natl Acad Sci U S A 99, 4391-4396 (2002)
http://dx.doi.org/10.1073/pnas.032074999

Mummery, C. et al., J Anat 200, 233-242 (2002)
http://dx.doi.org/10.1046/j.1469-7580.2002.00031.x

Pfeifer, A. et al., Proc Natl Acad Sci U S A 99, 2140-2145 (2002)
http://dx.doi.org/10.1073/pnas.251682798

Richards, M. et al., Nat Biotechnol 20, 933-936 (2002)
http://dx.doi.org/10.1038/nbt726

Sathananthan, H. et al., Reprod Biomed Online 4, 56-61 (2002)
http://www.rbmonline.com/4DCGI/Article/Detail?38%091%09=%20392%09

Xu, C. et al., Circ Res 91, 501-508 (2002)
http://dx.doi.org/10.1161/01.RES.0000035254.80718.91

Xu, R. H. et al., Nat Biotechnol 20, 1261-1264 (2002)
http://dx.doi.org/10.1038/nbt761

2003 Amit, M. et al., Biol Reprod 68, 2150-2156 (2003)
http://dx.doi.org/10.1095/biolreprod.102.012583

Carpenter, M. K. et al., Cloning Stem Cells 5, 79-88 (2003)
http://dx.doi.org/10.1089/153623003321512193

Chadwick, K. et al., Blood 102, 906-915 (2003)
http://dx.doi.org/10.1182/blood-2003-03-0832

2003 Chen, Y. et al., Cell Res 13, 251-263 (2003)
http://dx.doi.org/10.1038/sj.cr.7290170

Cheng, L. Z. et al., Stem Cells 21, 131-142 (2003)
http://dx.doi.org/10.1634/stemcells.21-2-131

Gerecht-Nir, S. et al., Lab Invest 83, 1811-1820 (2003)
http://dx.doi.org/10.1097/01.LAB.0000106502.41391.F0

Green, H. et al., Proc Natl Acad Sci U S A 100, 15625-15630 (2003)
http://dx.doi.org/10.1073/pnas.0307226100

Gropp, M. et al., Mol Ther 7, 281-287 (2003)
http://dx.doi.org/10.1016/S1525-0016(02)00047-3

He, J. Q. et al., Circ Res 93, 32-39 (2003)
http://dx.doi.org/10.1161/01.RES.0000080317.92718.99

Hovatta, O. et al., Hum Reprod 18, 1404-1409 (2003)
http://dx.doi.org/10.1093/humrep/deg290 [doi]

Levenberg, S. et al., Proc Natl Acad Sci U S A 100, 12741-12746 (2003)
http://dx.doi.org/10.1073/pnas.1735463100

Ma, Y. et al., Stem Cells 21, 111-117 (2003)
http://dx.doi.org/10.1634/stemcells.21-1-111

Mitalipova, M. et al., Stem Cells 21, 521-526 (2003)
http://dx.doi.org/10.1634/stemcells.21-5-521

Moore, F. L. et al., Proc Natl Acad Sci U S A 100, 538 (2003)
http://dx.doi.org/10.1073/pnas.0234478100

Mummery, C. et al., Circulation 107, 2733-2740 (2003)
http://dx.doi.org/10.1161/01.CIR.0000068356.38592.68

Park, J. H. et al., Biol Reprod 69, 2007-2014 (2003)
http://dx.doi.org/10.1095/biolreprod.103.017467

Park, S. et al., Neurosci Lett 353, 91-94 (2003)
http://dx.doi.org/10.1016/j.neulet.2003.08.082

Pickering, S. J. et al., Reprod Biomed Online 7, 353-364 (2003)
http://www.rbmonline.com/4DCGI/Article/Detail?38%091%09=%201074%09

Rajagopal, J. et al., Science 299, 363 (2003)
http://dx.doi.org/10.1126/science.1077838

Rambhatla, L. et al., Cell Transplant 12, 1-11 (2003)
http://www.ingentaconnect.com/content/cog/ct/2003/00000012/00000001/art00001

Richards, M. et al., Stem Cells 21, 546-556 (2003)
http://stemcells.alphamedpress.org/cgi/content/full/21/5/546

2003 Sato, N. et al., Dev Biol 260, 404-412 (2003)
http://dx.doi.org/10.1016/S0012-1606(03)00256-2

Schuldiner, M. et al., Stem Cells 21, 257-265 (2003)
http://stemcells.alphamedpress.org/cgi/content/abstract/21/3/257

Schulz, T. C. et al., BMC Neurosci 4, 27 (2003)
http://dx.doi.org/10.1186/1471-2202-4-27

Smith-Arica, J. R. et al., Cloning Stem Cells 5, 51-62 (2003)
http://dx.doi.org/10.1089/153623003321512166

Snir, M. et al., Am J Physiol Heart Circ Physiol 285, H2355 (2003)
http://dx.doi.org/10.1152/ajpheart.00020.2003

Sottile, V. et al., Cloning Stem Cells 5, 149-155 (2003)
http://dx.doi.org/10.1089/153623003322234759

Sperger, J. M. et al., Proc Natl Acad Sci U S A 100, 13350-13355 (2003)
http://dx.doi.org/10.1073/pnas.2235735100

Tzukerman, M. et al. Proc Natl Acad Sci U S A 100, 13507-13512 (2003)
http://dx.doi.org/10.1073/pnas.2235551100

Walsh, J. & Andrews, P. W., Apmis 111, 197-210 (2003)
http://dx.doi.org/10.1034/j.1600-0463.2003.1110124.x

Zwaka, T. P.& Thomson, J. A., Nat Biotechnol 21, 319-321 (2003)
http://dx.doi.org/10.1038/nbt788

2004 Abeyta, M. J. et al., Hum Mol Genet 13, 601-608 (2004)
http://dx.doi.org/10.1093/hmg/ddh068

Amit, M. et al., Biol Reprod 70, 837-845 (2004)
http://dx.doi.org/10.1095/biolreprod.103.021147

Anderson, D. G. et al., Nat Biotechnol 22, 863-866 (2004)
http://dx.doi.org/10.1038/nbt981

Anneren, C.& Cowan, C. A., J Biol Chem 279, 31590-31598 (2004)
http://dx.doi.org/10.1074/jbc.M403547200

Baharvand, H. et al., Differentiation 72, 224-229 (2004)
http://dx.doi.org/10.1111/j.1432-0436.2004.07205005.x

Ben-Hur, T. et al., Stem Cells 22, 1246-1255 (2004)
http://dx.doi.org/10.1634/stemcells.2004-0094

Besser, D., J Biol Chem 279, 45076-45084 (2004)
http://dx.doi.org/10.1074/jbc.M404979200

Bhattacharya, B. et al., Blood 103, 2956-2964 (2004)
http://dx.doi.org/10.1182/blood-2003-09-3314

2004 Bieberich, E. et al., J Cell Biol 167, 723-734 (2004)
http://dx.doi.org/10.1083/jcb.200405144

Bielby, R. C. et al., Tissue Eng 10, 1518-1525 (2004)
http://dx.doi.org/10.1089/ten.2004.10.1518

Brandenberger, R. et al., BMC Dev Biol 4, 10 (2004)
http://dx.doi.org/10.1186/1471-213X-4-10

Brandenberger, R. et al., Nat Biotechnol 22, 707-716 (2004)
http://dx.doi.org/10.1038/nbt971

Brimble, S. N. et al., Stem Cells Dev 13, 585-597 (2004)
http://dx.doi.org/10.1089/scd.2004.13.585

Buytaert-Hoefen, K. A. et al., Stem Cells 22, 669-674 (2004)
http://dx.doi.org/10.1634/stemcells.22-5-669

Buzzard, J. J. et al., Nat Biotechnol 22, 381-382 (2004)
http://dx.doi.org/10.1038/nbt0404-381

Calhoun, J. D. et al., Biochem Biophys Res Commun 323, 453-464 (2004)
http://dx.doi.org/10.1016/j.bbrc.2004.08.117

Carpenter, M. K. et al., Dev Dyn 229, 243-258 (2004)
http://dx.doi.org/10.1002/dvdy.10431

Cerdan, C., Rouleau, A., Bhatia, M., Blood 103, 2504-2512 (2004)
http://dx.doi.org/10.1182/blood-2003-07-2563

Choo, A. B. et al., Biotechnol Bioeng 88, 321-331 (2004)
http://dx.doi.org/10.1002/bit.20247

Clark, A. T. et al., Hum Mol Genet 13, 727-739 (2004)
http://dx.doi.org/10.1093/hmg/ddh088

Clark, A. T. et al., Stem Cells 22, 169-179 (2004)
http://dx.doi.org/10.1634/stemcells.22-2-169

Conley, B. J. et al., Fetal Diagn Ther 19, 218-223 (2004)
http://dx.doi.org/10.1159/000076701

Cowan, C. A. et al., N Engl J Med 350, 1353-1356 (2004)
http://dx.doi.org/10.1056/NEJMsr040330

Daheron, L. et al., Stem Cells 22, 770-778 (2004)
http://dx.doi.org/10.1634/stemcells.22-5-770

Dang, S. M. et al., Stem Cells 22, 275-282 (2004)
http://dx.doi.org/10.1634/stemcells.22-3-275

Dhara, S. K. & Benvenisty, N., Nucleic Acids Res 32, 3995-4002 (2004)
http://dx.doi.org/10.1093/nar/gkh746

2004 Draper, J. S. et al., Nat Biotechnol 22, 53-54 (2004)
http://dx.doi.org/10.1038/nbt922

Draper, J. S. et al., Stem Cells Dev 13, 325-336 (2004)
http://dx.doi.org/10.1089/1547328041797525

Dvash, T. et al., Hum Reprod 19, 2875-2883 (2004)
http://dx.doi.org/10.1093/humrep/deh529

Fujioka, T. et al., Int J Dev Biol 48, 1149-1154 (2004)
http://dx.doi.org/10.1387/ijdb.041852tf

Gerami-Naini, B. et al., Endocrinology 145, 1517-1524 (2004)
http://dx.doi.org/10.1210/en.2003-1241

Gerecht-Nir, S. et al., Biol Reprod 71, 2029-2036 (2004)
http://dx.doi.org/10.1095/biolreprod.104.031930

Gerecht-Nir, S. et al., Biotechnol Bioeng 86, 493-502 (2004)
http://dx.doi.org/10.1002/bit.20045

Gerecht-Nir, S. et al., Biotechnol Bioeng 88, 313-320 (2004)
http://dx.doi.org/10.1002/bit.20248

Gertow, K. et al., Stem Cells Dev 13, 421-435 (2004)
http://dx.doi.org/10.1089/1547328041797499

Ginis, I. et al., Dev Biol 269, 360-380 (2004)
http://dx.doi.org/10.1016/j.ydbio.2003.12.034

Hansson, M. et al., Diabetes 53, 2603-2609 (2004)
http://dx.doi.org/10.2337/diabetes.53.10.2603

Hay, D. C. et al., Stem Cells 22, 225-235 (2004)
http://dx.doi.org/10.1634/stemcells.22-2-225

Heins, N. et al., Stem Cells 22, 367-376 (2004)
http://stemcells.alphamedpress.org/cgi/content/abstract/22/3/367

Heng, B. C. et al., In Vitro Cell Dev Biol Anim 40, 255-257 (2004)
http://dx.doi.org/10.1290/0407052.1

Hong, S. H. et al., Mol Cells 18, 320-325 (2004)
http://molcells.inforang.com/article_pdf/Ksmcb/18/Ksmcb18-3-7.pdf

Humphrey, R. K. et al., Stem Cells 22, 522-530 (2004)
http://dx.doi.org/10.1634/stemcells.22-4-522

Huntriss, J. et al., Mol Reprod Dev 67, 323-336 (2004)
http://dx.doi.org/10.1002/mrd.20030

Imreh, M. P. et al., Stem Cells Dev 13, 337-343 (2004)
http://dx.doi.org/10.1089/1547328041797534

2004 Inzunza, J. et al., Mol Hum Reprod 10, 461-466 (2004)
http://dx.doi.org/10.1093/molehr/gah051

Ji, L. et a ., Biotechnol Bioeng 88, 299-312 (2004)
http://dx.doi.org/10.1002/bit.20243

Johkura, K. et al., J Anat 205, 247-255 (2004)
http://dx.doi.org/10.1111/j.0021-8782.2004.00336.x

Kehat, I. et al., Nat Biotechnol 22, 1282-1289 (2004)
http://dx.doi.org/10.1038/nbt1014

Klimanskaya, I. et al., Cloning Stem Cells 6, 217-245 (2004)
http://dx.doi.org/10.1089/clo.2004.6.217

Koivisto, H. et al., Reprod Biomed Online 9, 330-337 (2004)
http://www.rbmonline.com/4DCGI/Article/Detail?38%091%09=%201375%09

Lakshmipathy, U. et al., Stem Cells 22, 531-543 (2004)
http://stemcells.alphamedpress.org/cgi/content/full/22/4/531

Lavon, N. et al., Differentiation 72, 230 (2004)
http://dx.doi.org/10.1111/j.1432-0436.2004.07205002.x

Lee, J. B. et al., Reproduction 128, 727-735 (2004)
http://dx.doi.org/10.1530/rep.1.00415

Li, L. et al., Stem Cells 22, 448-456 (2004)
http://dx.doi.org/10.1634/stemcells.22-4-448

Liu, Y. P. et al., Stem Cells Dev 13, 636-645 (2004)
http://dx.doi.org/10.1089/scd.2004.13.636

Lu, S. J. et al., Blood 103, 4134-4141 (2004)
http://dx.doi.org/10.1182/blood-2003-10-3575

Magyar, J. et al., Cardiovasc Res 64, 477-487 (2004)
http://dx.doi.org/10.1016/j.cardiores.2004.07.014

Matin, M. M. et al., Stem Cells 22, 659-668 (2004)
http://dx.doi.org/10.1634/stemcells.22-5-659

Menendez, P. et al., Mol Ther 10, 1109-1120 (2004)
http://dx.doi.org/10.1016/j.ymthe.2004.08.016

Miura, T. et al., Stem Cells Dev 13, 694-715 (2004)
http://dx.doi.org/10.1089/scd.2004.13.694

Moore, F. L. et al., Genomics 83, 834-843 (2004)
http://dx.doi.org/10.1016/j.ygeno.2003.11.005

Nat, R., Hovatta, O., J Cell Mol Med 8, 570-571 (2004)
http://dx.doi.org/10.1111/j.1582-4934.2004.tb00482.x

2004 Orner, B. P. et al., J Am Chem Soc 126, 10808-10809 (2004)
http://dx.doi.org/10.1021/ja0474291

Park, J. H. et al., Mol Cells 17, 309-315 (2004)
http://molcells.inforang.com/article_pdf/Ksmcb/17/Ksmcb17-2-19.pdf

Park, S. et al., Neurosci Lett 359, 99-103 (2004)
http://dx.doi.org/10.1016/j.neulet.2004.01.073

Park, S. H. et al., Ultrastruct Pathol 28, 229-238 (2004)
http://dx.doi.org/10.1080/01913120490515595

Park, S. P. et al., Hum Reprod 19, 676-684 (2004)
http://dx.doi.org/10.1093/humrep/deh102

Pera, M. F. et al., J Cell Sci 117, 1269-1280 (2004)
http://dx.doi.org/10.1242/jcs.00970

Perrier, A. L. et al., Proc Natl Acad Sci U S A 101, 12543-12548 (2004)
http://dx.doi.org/10.1073/pnas.0404700101

Ponsaerts, P. et al., Cloning Stem Cells 6, 211-216 (2004)
http://dx.doi.org/10.1089/clo.2004.6.211

Rao, R. R. et al., Biotechnol Bioeng 88, 273-286 (2004)
http://dx.doi.org/10.1002/bit.20245

Reppel, M., et al., Cell Physiol Biochem 14, 187-196 (2004)
http://dx.doi.org/10.1159/000080326

Richards, M. et al., Stem Cells 22, 51-64 (2004)
http://dx.doi.org/10.1634/stemcells.22-1-51

Richards, M. et al., Stem Cells 22, 779-789 (2004)
http://dx.doi.org/10.1634/stemcells.22-5-779

Rosler, E. S. et al., Dev Dyn 229, 259-274 (2004)
http://dx.doi.org/10.1002/dvdy.10430

Satin, J. et al., J Physiol-London 559, 479-496 (2004)
http://dx.doi.org/10.1113/jphysiol.2004.068213

Sato, N. et al., Nat Med 10, 55-63 (2004)
http://dx.doi.org/10.1038/nm979

Schulz, T. C. et al., Stem Cells 22, 1218-1238 (2004)
http://dx.doi.org/10.1634/stemcells.2004-0114

Segev, H. et al., Stem Cells 22, 265-274 (2004)
http://dx.doi.org/10.1634/stemcells.22-3-265

Shirahashi, H. et al., Cell Transplant 13, 197-211 (2004)
http://www.ingentaconnect.com/content/cog/ct/2004/00000013/00000003/art00001

2004 Sjogren, A. et al., Reprod Biomed Online 9, 326-329 (2004)
http://www.rbmonline.com/4DCGI/Article/Detail?38%091%09=%201428%09

Stojkovic, M. et al., Stem Cells 22, 790-797 (2004)
http://dx.doi.org/10.1634/stemcells.22-5-790

Strelchenko, N. et al., Reprod Biomed Online 9, 623-629 (2004)
http://www.rbmonline.com/4DCGI/Article/Detail?38%091%09=%201558%09

Suh, M. R. et al., Dev Biol 270, 488-498 (2004)
http://dx.doi.org/10.1016/j.ydbio.2004.02.019

Suss-Toby, E. et al., Hum Reprod 19, 670-675 (2004)
http://dx.doi.org/10.1093/humrep/deh135

Tian, X. et al., Exp Hematol 32, 1000-1009 (2004)
http://dx.doi.org/10.1016/j.exphem.2004.06.013

Urbach, A.et al., Stem Cells 22, 635-641 (2004)
http://dx.doi.org/10.1634/stemcells.22-4-635

Vallier, L. et al., Dev Biol 275, 403-421 (2004)
http://dx.doi.org/10.1016/j.ydbio.2004.08.031

Vallier, L. et al., Stem Cells 22, 2-11 (2004)
http://dx.doi.org/10.1634/stemcells.22-1-2

Wang, L. et al., Immunity 21, 31-41 (2004)
http://dx.doi.org/10.1016/j.immuni.2004.06.006

Wong, R. C. B. et al., Stem Cells 22, 883-889 (2004)
http://dx.doi.org/10.1634/stemcells.22-6-883

Xie, C. et al., Biochem Biophys Res Commun 315, 581-588 (2004)
http://dx.doi.org/10.1016/j.bbrc.2004.01.089

Xu, C. et al., Stem Cells 22, 972-980 (2004)
http://dx.doi.org/10.1634/stemcells.22-6-972

Zeng, X. et al., Restor Neurol Neurosci 22, 421-428 (2004)
http://iospress.metapress.com/content/mrwlamy9p535vjar/

Zeng, X. et al., Stem Cells 22, 292-312 (2004)
http://dx.doi.org/10.1634/stemcells.22-3-292

Zeng, X. et al., Stem Cells 22, 925-940 (2004)
http://dx.doi.org/10.1634/stemcells.22-6-925

Zhan, X. et al., Lancet 364, 163-171 (2004)
http://dx.doi.org/10.1016/S0140-6736(04)16629-4

Zhou, C. Q. et al., Chin Med J (Engl) 117, 1050-1055 (2004)
http://www.cmj.org/Periodical/AbstractList.asp?titleid=LW7519

2005 Amit, M. et al., Stem Cells 23, 761-771 (2005)
http://dx.doi.org/10.1634/stemcells.2004-0046

Andrews, P. W. et al., Biochem Soc Trans 33, 1526-1530 (2005)
http://dx.doi.org/10.1042/BST20051526

Barberi, T. et al., PLoS Med 2, e161 (2005)
http://dx.doi.org/10.1371/journal.pmed.0020161

Beattie, G. M. et al., Stem Cells 23, 489-495 (2005)
http://dx.doi.org/10.1634/stemcells.2004-0279

Bhattacharya, B. et al., BMC Dev Biol, 5, 22 (2005)
http://dx.doi.org/10.1186/1471-213X-5-22

Boyer, L. A. et al., Cell 122, 947-956 (2005)
http://dx.doi.org/10.1016/j.cell.2005.08.020

Brolen, G. K. C. et al., Diabetes 54, 2867-2874 (2005)
http://dx.doi.org/10.2337/diabetes.54.10.2867

Cai, J. et al., BMC Dev Biol 5, 26 (2005)
http://dx.doi.org/10.1186/1471-213X-5-26

Cao, T. et al., Tissue Cell 37, 325-334 (2005)
http://dx.doi.org/10.1016/j.tice.2005.03.008

Cauffman, G. et al., Mol Hum Reprod 11, 405-411 (2005)
http://dx.doi.org/10.1093/molehr/gah167

Chen, H. et al., Hum Reprod 20, 2201-2206 (2005)
http://dx.doi.org/10.1093/humrep/dei010

Chew, J. L. et al., Mol Cell Biol 25, 6031-6046 (2005)
http://dx.doi.org/10.1128/MCB.25.14.6031-6046.2005

Costa, M. et al., Nat Methods 2, 259-260 (2005)
http://dx.doi.org/10.1038/NMETH748

Cowan, C. A. et al., Science 309, 369-373 (2005)
http://dx.doi.org/10.1126/science.1116447

D'Amour, K. A. et al., Nat Biotechnol 23, 1534-1541(2005)
http://dx.doi.org/10.1038/nbt1163

Darnfors, C. et al., Stem Cells 23, 483-488 (2005)
http://dx.doi.org/10.1634/stemcells.2004-0254

Dolnikov, K. et al., Ann N Y Acad Sci 1047, 66-75 (2005)
http://dx.doi.org/10.1196/annals.1341.006

Dravid, G. et al., Stem Cells 23, 1489-1501 (2005)
http://dx.doi.org/10.1634/stemcells.2005-0034

2005 Dvorak, P. et al., Stem Cells 23, 1200-1211 (2005)
http://dx.doi.org/10.1634/stemcells.2005-0303

Enver, T. et al., Hum Mol Genet 14, 3129-1340 (2005)
http://dx.doi.org/10.1093/hmg/ddi345

Ezashi, T. et al., Proc Natl Acad Sci U S A 102, 4783-4788 (2005)
http://dx.doi.org/10.1073/pnas.0501283102

Ezeh, U. I., et al., Cancer,104, 2255-2265 (2005)
http://dx.doi.org/10.1002/cncr.21432

Fang, Z. F. et al., Cell Res 15, 394-400 (2005)
http://dx.doi.org/10.1038/sj.cr.7290307

Faulkner, J.& Keirstead, H. S., Transpl Immunol 15, 131-142 (2005)
http://dx.doi.org/10.1016/j.trim.2005.09.007

Findikli, N. et al., Reprod Biomed Online 10, 617-627 (2005)
http://www.rbmonline.com/4DCGI/Article/Detail?38%091%09=%201658%09

Fong, W. J. et al., Bioprocess Biosyst Eng 27, 381-387 (2005)
http://dx.doi.org/10.1007/s00449-005-0421-5

Genbacev, O. et al., Fertil Steril 83, 1517-1529 (2005)
http://dx.doi.org/10.1016/j.fertnstert.2005.01.086

Gerecht-Nir, S. et al., Dev Dyn 232, 487-497 (2005)
http://dx.doi.org/10.1002/dvdy.20247

Gerrard, L. et al., Stem Cells 23, 1234-1241 (2005)
http://dx.doi.org/10.1634/stemcells.2005-0110

Gerrard, L. et al., Stem Cells 23, 124-133 (2005)
http://dx.doi.org/10.1634/stemcells.2004-0102

Goh, G. et al., Thromb Haemost 94, 728-737 (2005)
http://dx.doi.org/10.1160/TH05-04-0268

Golan-Mashiach, M. et al., Faseb J 19, 147-149 (2005)
http://dx.doi.org/10.1096/fj.04-2417fje

Ha, S. Y. et al., Hum Reprod 20, 1779-1785 (2005)
http://dx.doi.org/10.1093/humrep/deh854

Hatano, S. Y. et al., Mech Dev 122, 67-79 (2005)
http://dx.doi.org/10.1016/j.mod.2004.08.008

Heng, B. C. et al., Ann Clin Lab Sci 35, 459-462 (2005)
http://www.annclinlabsci.org/cgi/content/abstract/35/4/459

Heng, B. C. et al., Cell Biochem Funct 23, 141-146 (2005)
http://dx.doi.org/10.1002/cbf.1221

2005 Hoffman, L. M. et al., Stem Cells 23, 1468-1478 (2005)
http://dx.doi.org/10.1634/stemcells.2004-0371

Hyslop, L. et al., Stem Cells 23, 1035-1043 (2005)
http://dx.doi.org/10.1634/stemcells.2005-0080

Inzunza, J. et al., Stem Cells 23, 544-549 (2005)
http://dx.doi.org/10.1634/stemcells.2004-0201

Itsykson, P. et al., Mol Cell Neurosci 30, 24-36 (2005)
http://dx.doi.org/10.1016/j.mcn.2005.05.004

James, D. et al., Development 132, 1273-1282 (2005)
http://dx.doi.org/10.1242/dev.01706

Kameda, T. & Thomson, J. A., Stem Cells 23, 1535-1540 (2005)
http://dx.doi.org/10.1634/stemcells.2005-0054

Kang, H. B., et al., Stem Cells Dev 14, 395-401 (2005)
http://dx.doi.org/10.1089/scd.2005.14.395

Keirstead, H. S. et al., J Neurosci 25, 4694-4705 (2005)
http://dx.doi.org/10.1523/JNEUROSCI.0311-05.2005

Khoo, M. L., et al., Biol Reprod 73, 1147-1156 (2005)
http://dx.doi.org/10.1095/biolreprod.104.036673

Kim, H. S., et al., Stem Cells 23, 1228-1233 (2005)
http://dx.doi.org/10.1634/stemcells.2004-0296

Kim, J. H. et al., Exp Mol Med 37, 36-44 (2005)
http://www.e-emm.org/search_read.htm?page=36&year=2005&vol=37

Kim, S. J. et al., Mol Cells 19, 46-53 (2005)
http://molcells.inforang.com/article_pdf/Ksmcb/19/Ksmcb19-1-6.pdf

Kim, S. J., et al.,Yonsei Med J 46, 693-699 (2005)
http://ymj.or.kr/abstracts/viewArticle.asp?year=2005&month=10&page=693

Kim, S. K. et al., Stem Cells 23, 458-462 (2005)
http://dx.doi.org/10.1634/stemcells.2004-0245

Klimanskaya, I. et al., Lancet 365, 1636-1641 (2005)
http://dx.doi.org/10.1016/S0140-6736(05)66473-2

Kuroda, T. et al., Mol Cell Biol 25, 2475-2485 (2005)
http://dx.doi.org/10.1128/MCB.25.6.2475-2485.2005

Kwon, Y. D. et al., Mol Ther 12, 28-32 (2005)
http://dx.doi.org/10.1016/j.ymthe.2005.03.010

Laflamme, M. A. et al., Am J Pathol 167, 663-671 (2005)
http://ajp.amjpathol.org/cgi/content/abstract/167/3/663

2005 Lee, J. B. et al., Biol Reprod 72, 42-49 (2005)
http://dx.doi.org/10.1095/biolreprod.104.033480

Lee, J. B. et al., Mol Cells 19, 31-38 (2005)
http://molcells.inforang.com/article_pdf/Ksmcb/19/Ksmcb19-1-4.pdf

Lee, J. et al., Stem Cells 23, 738-751 (2005)
http://dx.doi.org/10.1634/stemcells.2004-0375

Lev, S. et al., Ann N Y Acad Sci 1047, 50-65 (2005)
http://dx.doi.org/10.1196/annals.1341.005

Levenberg, S. et al., Tissue Eng 11, 506-512 (2005)
http://dx.doi.org/10.1089/ten.2005.11.506

Li, T. et al., Chin Med J (Engl) 118, 116-122 (2005)
http://www.cmj.org/Periodical/abstractlist.asp?titleid=LW7858

Li, X. J. et al., Nat Biotechnol 23, 215-221 (2005)
http://dx.doi.org/10.1038/nbt1063

Li, Y. et al., Biotechnol Bioeng 91, 688-698 (2005)
http://dx.doi.org/10.1002/bit.20536

Liu, Y. P. et al., Stem Cells Dev 14, 487-492 (2005)
http://dx.doi.org/10.1089/scd.2005.14.487

Maitra, A. et al., Nat Genet 37, 1099-1103 (2005)
http://dx.doi.org/10.1038/ng1631

Martin, M. J. et al., Nat Med 11, 228-232 (2005)
http://dx.doi.org/10.1038/nm1181

McDevitt T. C. et al., J Mol Cell Cardiol 39, 865-873 (2005)
http://dx.doi.org/10.1016/j.yjmcc.2005.09.007

Mitalipova, M. M. et al., Nat Biotechnol 23, 19-20 (2005)
http://dx.doi.org/10.1038/nbt0105-19

Mossman, A. K. et al., Stem Cells Dev 14, 656-663 (2005)
http://dx.doi.org/10.1089/scd.2005.14.656

Muotri, A. R. et al., Proc Natl Acad Sci U S A 102, 18644-18648 (2005)
http://dx.doi.org/10.1073/pnas.0509315102

Ng, E. S. et al., Blood 106, 1601-1603 (2005)
http://dx.doi.org/10.1182/blood-2005-03-0987

Nistor, G. I. et al., Glia 49, 385-396 (2005)
http://dx.doi.org/10.1002/glia.20127

Noaksson, K. et al., Stem Cells 23, 1460-1467 (2005)
http://dx.doi.org/10.1634/stemcells.2005-0093

2005 Oh, S. K. et al., Stem Cells 23, 211-219 (2005)
http://dx.doi.org/10.1634/stemcells.2004-0122

Oh, S. K. et al., Stem Cells 23, 605 -609 (2005)
http://dx.doi.org/10.1634/stemcells.2004-0297

Park, C. H. et al., J Neurochem 92, 1265-1276 (2005)
http://dx.doi.org/10.1111/j.1471-4159.2004.03006.x

Passier, R. et al., Stem Cells 23, 772-780 (2005)
http://dx.doi.org/10.1634/stemcells.20040184

Pebay, A. et al., Stem Cells 23, 1541-1548 (2005)
http://dx.doi.org/10.1634/stemcells.2004-0338

Pickering, S. J. et al., Reprod Biomed Online 10, 390-397 (2005)
http://www.rbmonline.com/4DCGI/Article/Detail?38%091%09=%201620%09

Pomp, O. et al., Stem Cells 23, 923-930 (2005)
http://dx.doi.org/10.1634/stemcells.2005-0038

Prowse, A. B. et al., Proteomics 5, 978-989 (2005)
http://dx.doi.org/10.1002/pmic.200401087

Qiu, C. et al., Exp Hematol 33, 1450-1458 (2005)
http://dx.doi.org/10.1016/j.exphem.2005.09.003

Ren, C. P. et al., Acta Biochim Biophys Sin (Shanghai) 37, 68 -73 (2005)
http://dx.doi.org/10.1111/j.1745-7270.2005.00001.x

Reppel, M. et al., J Electrocardiol 38, 166-170 (2005)
http://dx.doi.org/10.1016/j.jelectrocard.2005.06.029

Rodda, D. J. et al., J Biol Chem 280, 24731-24737 (2005)
http://dx.doi.org/10.1074/jbc.M502573200

Rugg-Gunn, P. J.et al., Nat Genet 37, 585-587 (2005)
http://dx.doi.org/10.1038/ng1556

Schwartz, C. M. et al., Stem Cells Dev 14, 517-534 (2005)
http://dx.doi.org/10.1089/scd.2005.14.517

Schwartz, R. E. et al., Stem Cells Dev 14, 643-655 (2005)
http://dx.doi.org/10.1089/scd.2005.14.643

Segev, H. et al., Dev Growth Differ 47, 295-306 (2005)
http://dx.doi.org/10.1111/j.1440-169X.2005.00803.x

Shin, S. et al., Stem Cells Dev 14, 266-269 (2005)
http://dx.doi.org/10.1089/scd.2005.14.266

Siemen, H. et al., Stem Cells Dev 14, 378-383 (2005)
http://dx.doi.org/10.1089/scd.2005.14.378

2005 Simon, C. et al., Fertil Steril 83, 246-249 (2005)
http://dx.doi.org/10.1016/j.fertnstert.2004.09.004

Singh Roy, N. et al., Exp Neurol 196, 224-234 (2005)
http://dx.doi.org/10.1016/j.expneurol.2005.06.021

Sjögren-Jansson, E. et al., Dev Dyn 233, 1304-1314 (2005)
http://dx.doi.org/10.1002/dvdy.20459

Skottman, H. et al., Stem Cells 23, 1343-1356 (2005)
http://dx.doi.org/10.1634/stemcells.2004-0341

Son, Y. S. et al., Stem Cells 23, 1502-1513 (2005)
http://dx.doi.org/10.1634/stemcells.2004-0307

St John, J. C. et al., Cloning Stem Cells 7, 141-153 (2005)
http://dx.doi.org/10.1089/clo.2005.7.141

Stamp, L. et al., Stem Cells 23, 103-112 (2005)
http://dx.doi.org/10.1634/stemcells.2004-0147

Steele, W. et al., Reprod Biomed Online 10, 755-766 (2005)
http://www.rbmonline.com/4DCGI/Article/Detail?38%091%09=%201665%09

Stojkovic, P. et al., Stem Cells 23, 306-314 (2005)
http://dx.doi.org/10.1634/stemcells.2004-0137

Stojkovic, P. et al., Stem Cells 23, 895-902 (2005)
http://dx.doi.org/10.1634/stemcells.2004-0326

Tabar, V. et al., Nat Biotechnol 23, 601-616 (2005)
http://dx.doi.org/10.1038/nbt1088

Tai, G., et al., Biochem Biophys Res Commun 333, 1116-1122 (2005)
http://dx.doi.org/10.1016/j.bbrc.2005.05.195

Tan, S. M. & Droge, P., Stem Cells 23, 868-873 (2005)
http://dx.doi.org/10.1634/stemcells.2005-0044

Vallier, L. et al., J Cell Sci 118, 4495-4509 (2005)
http://dx.doi.org/10.1242/jcs.02553

van de Stolpe, A. et al., Reprod Biomed Online 11, 476-485 (2005)
http://www.rbmonline.com/4DCGI/Article/Detail?38%091%09=%201849%09

Venable, A. et al., BMC Dev Biol 5, 15 (2005)
http://dx.doi.org/10.1186/1471-213X-5-15

Verlinsky, Y. et al., Reprod Biomed Online 10, 105-110 (2005)
http://www.rbmonline.com/4DCGI/Article/Detail?38%091%09=%201590%09

Vodyanik, M. A. et al., Blood 105, 617-626 (2005)
http://dx.doi.org/10.1182/blood-2004-04-1649

2005 Wang, G. et al., Biochem Biophys Res Commun 330, 934-942 (2005)
http://dx.doi.org/10.1016/j.bbrc.2005.03.058

Wang, J. et al., Cell Biol Int 29, 654-661 (2005)
http://dx.doi.org/10.1016/j.cellbi.2005.03.019

Wang, K. et al., Stem Cells 23, 1526-1534 (2005)
http://dx.doi.org/10.1634/stemcells.2004-0299

Wang, L. et al., Blood 105, 4598-4603 (2005)
http://dx.doi.org/10.1182/blood-2004-10-4065

Wang, L. et al., J Exp Med 201, 1603-1614 (2005)
http://dx.doi.org/10.1084/jem.20041888

Wang, Q. et al., Stem Cells 23, 1221-1227 (2005)
http://dx.doi.org/10.1634/stemcells.2004-0347

Ware, C. B. et al., Biotechniques 38, 879-983 (2005)
http://dx.doi.org/10.1007/978-1-59745-536-7_4

Wei, C. L. et al., Stem Cells 23, 166-185 (2005)
http://dx.doi.org/10.1634/stemcells.2004-0162

Wiblin, A. E., et al., J Cell Sci 118, 3861-3868 (2005)
http://dx.doi.org/10.1242/jcs.02500

Woll, P. S. et al., J Immunol 175, 5095-5103 (2005)
http://www.jimmunol.org/cgi/content/abstract/175/8/5095

Wu, C. F. et al., Reprod Biomed Online 11, 733-739 (2005)
http://www.rbmonline.com/4DCGI/Article/Detail?38%091%09=%201944%09

Xie, C. Q. et al., Cell Biol Int 29, 623-628 (2005)
http://dx.doi.org/10.1016/j.cellbi.2005.03.019

Xiong, C. et al., Stem Cells Dev 14, 671-675 (2005)
http://dx.doi.org/10.1089/scd.2005.14.671

Xiong, C. et al., Stem Cells Dev,14, 367-377 (2005)
http://dx.doi.org/10.1089/scd.2005.14.367

Xu, C. et al., Stem Cells 23, 315-323 (2005)
http://dx.doi.org/10.1634/stemcells.2004-0211

Xu, R. H. et al., Nat Methods 2, 185-190 (2005)
http://dx.doi.org/10.1038/NMETH744

Xue, T. et al., Circulation 111, 11-20 (2005)
http://dx.doi.org/10.1161/01.CIR.0000151313.18547.A2

Yan, Y. et al., Stem Cells 23, 781-790 (2005)
http://dx.doi.org/10.1634/stemcells.2004-0365

2005 Yang, A. X. et al., Stem Cells Dev 14, 270-284 (2005)
http://dx.doi.org/10.1089/scd.2005.14.270

Yoo, S. J. et al., Exp Mol Med 37, 399-407 (2005)
http://www.e-emm.org/current_abstract_view.htm?sid1=517&volume=37&no=5&articletype

Zaehres, H. et al., Stem Cells 23, 299-305 (2005)
http://dx.doi.org/10.1634/stemcells.2004-0252

Zambidis, E. T. et al., Blood 106, 860-870 (2005)
http://dx.doi.org/10.1182/blood-2004-11-4522

Zhan, M., et al., Cell Biochem Biophys 43, 379-405 (2005)
http://dx.doi.org/10.1385/CBB:43:3:379

Zwaka, T. P.& Thomson, J. A., Stem Cells 23, 146-149 (2005)
http://dx.doi.org/10.1634/stemcells.2004-0248

2006 Aghajanova, L. et al. Fertil Steril 86 Suppl 4, 1193-1209 (2006)
http://dx.doi.org/10.1016/j.fertnstert.2005.12.081

Ahn, S. E. et al., Biochem Biophys Res Commun, 340, 403-408 (2006)
http://dx.doi.org/10.1016/j.bbrc.2005.12.020

Anderson, J. S. et al., Retrovirology 3, 24 (2006)
http://dx.doi.org/10.1186/1742-4690-3-24

Androutsellis-Theotokis, A. et al. Nature 442, 823-826 (2006)
http://dx.doi.org/10.1038/nature04940

Armstrong, L. et al. Hum Mol Genet 15, 1894-1913 (2006)
http://dx.doi.org/10.1093/hmg/ddl112

Baharvand, H. et al. Dev Growth Differ 48, 117-128 (2006)
http://dx.doi.org/10.1111/j.1440-169X.2006.00851.x

Baharvand, H., et al., Dev Growth Differ 48, 323-332 (2006)
http://dx.doi.org/10.1111/j.1440-169X.2006.00867.x

Baharvand, H., et al., Int J Dev Biol 50, 645-652 (2006)
http://dx.doi.org/10.1387/ijdb.052072hb

Baharvand, H., et al., Proteomics 6, 3544-3549 (2006)
http://dx.doi.org/10.1002/pmic.200500844

Banin, E. et al., Stem Cells 24, 246-257 (2006)
http://dx.doi.org/10.1634/stemcells.2005-0009

Becker, K. A. et al. J Cell Physiol 209, 883-893 (2006)
http://dx.doi.org/10.1002/jcp.20776

Ben-Dor, I. et al. Mol Ther 14, 255-267 (2006)
http://dx.doi.org/10.1016/j.ymthe.2006.02.010

2006 Benzing, C. et al., Neuroreport 17, 1675-1681 (2006)
http://dx.doi.org/10.1097/01.wnr.0000236861.01210.72

Beqqali, A., et al., Stem Cells 24, 1956-1967 (2006)
http://dx.doi.org/10.1387/ijdb.052072hb

Bibikova, M. et al., Genome Res 16, 1075-1083 (2006)
http://dx.doi.org/10.1111/j.1440-169X.2006.00867.x

Bowles, K. M. et al., Stem Cells 24, 1359-1369 (2006)
http://dx.doi.org/10.1634/stemcells.2005-0210

Brederlau, A. et al., Stem Cells 24, 1433-1440 (2006)
http://dx.doi.org/10.1634/stemcells.2005-0393

Cai, J. et al., Stem Cells 24, 516-530 (2006)
http://dx.doi.org/10.1634/stemcells.2005-0143

Caisander, G. et al., Chromosome Res 14, 131-137 (2006)
http://dx.doi.org/10.1007/s10577-006-1019-8

Cameron, C. M. & Kaufman, D. S., Biotechnol Bioeng 94, 938-948 (2006)
http://dx.doi.org/10.1002/bit.20919

Cauffman, G. et al., Stem Cells 24, 2685-91 (2006)
http://dx.doi.org/10.1634/stemcells.2005-0611

Cerdan, C. et al. Nat Med 12, 1113-1114; author reply 1115 (2006)
http://dx.doi.org/10.1038/nm1006-1113

Chang, K. H. et al., Blood 108, 1515-1523 (2006)
http://dx.doi.org/10.1182/blood-2005-11-011874

Chen, S.& Oh, S. K., J Biotechnol 122, 341-361 (2006)
http://dx.doi.org/10.1016/j.jbiotec.2005.11.022

Chen, Y. et al., Cell Transplant 15, 865-871 (2006)
http://www.ingentaconnect.com/content/cog/ct/2006/00000015/00000010/art00004

Cho, Y. M. et al., Biochem Biophys Res Commun 348, 1472-1478 (2006)
http://dx.doi.org/10.1016/j.bbrc.2006.08.020

Choo, A. et al., J Biotechnol 122, 130-141 (2006)
http://dx.doi.org/10.1016/j.jbiotec.2005.09.008

Clements, M. O. et al., Tissue Eng 12, 1741-1751 (2006)
http://dx.doi.org/10.1089/ten.2006.12.1741

Cloutier, F. et al., Reg Med 1, 469-479 (2006)
http://dx.doi.org/10.2217/17460751.1.4.469

Constantinescu, D., et al., Stem Cells 24, 177-185 (2006)
http://dx.doi.org/10.1634/stemcells.2004-0159

2006 Cooke, M.J.et al., Stem Cells Dev 15, 254-259 (2006)
http://dx.doi.org/10.1089/scd.2006.15.254

D'Amour, K. A. et al., Nat Biotechnol 24, 1392-1401 (2006)
http://dx.doi.org/10.1038/nbt1259

Darr, H. et al., Development 133, 1193-1201 (2006)
http://dx.doi.org/10.1242/dev.02286

Dean, S. K. et al., Transplantation 82, 1175-1184 (2006)
http://dx.doi.org/10.1097/01.tp.0000239518.23354.64

Denning, C. et al., Int J Dev Biol 50, 27-37 (2006)
http://dx.doi.org/10.1387/ijdb.052107cd

Ding, V. et al., Biotechnol Lett 28, 491-495 (2006)
http://dx.doi.org/10.1007/s10529-006-0005-8

Dolnikov, K. et al., Stem Cells 24, 236-245 (2006)
http://dx.doi.org/10.1634/stemcells.2005-0036

Drukker, M. et al., Stem Cells 24, 221-229 (2006)
http://dx.doi.org/10.1634/stemcells.2005-0188

Ellerström, C. et al., Stem Cells 24, 2170-2176 (2006)
http://dx.doi.org/10.1634/stemcells.2006-0130

Fang, D. et al., Stem Cells 24, 1668-1677 (2006)
http://dx.doi.org/10.1634/stemcells.2005-0414

Fletcher, J.M. et al., Cloning Stem Cells 8, 319-334 (2006)
http://dx.doi.org/10.1089/clo.2006.8.319

Forsyth, N. R. et al., Cloning Stem Cells 8, 16-23 (2006)
http://dx.doi.org/10.1089/clo.2006.8.16

Galic, Z. et al. Proc Natl Acad Sci U S A 103, 11742-11747 (2006)
http://dx.doi.org/10.1073/pnas.0604244103

Gaur, M. et al. J Thromb Haemost 4, 436-442 (2006)
http://dx.doi.org/10.1111/j.1538-7836.2006.01744.x

Grinnemo, K. H. et al., Reprod Biomed Online 13, 712-724 (2006)
http://www.rbmonline.com/4DCGI/Article/Detail?38%091%09=%202365%09

Guillaume, D. J. et al., J Neurosci Res 84, 1165-1176 (2006)
http://dx.doi.org/10.1002/jnr.21022

Harun, R. et al. Hum Reprod 21, 1349-1358 (2006)
http://dx.doi.org/10.1093/humrep/del017

Hasegawa, K. et al., Stem Cells 24, 2649-2660 (2006)
http://dx.doi.org/10.1634/stemcells.2005-0657

2006 Heins, N. et al., J Biotechnol 122, 511-520 (2006)
http://dx.doi.org/10.1016/j.jbiotec.2005.10.010

Heng, B. C. et al., Int J Med Sci 13, 124-129 (2006)
http://www.medsci.org/v03p0124.htm

Heng, B. C. et al., J Biomed Sci 13, 433-445 (2006)
http://dx.doi.org/10.1007/s11373-005-9051-9

Heng, B. C. et al., Zygote 14, 361-348 (2006)
http://dx.doi.org/10.1017/S0967199406003893

Heng, B.C. et al., In Vitro Cell Dev Biol Anim 42, 54-57 (2006)
http://dx.doi.org/10.1290/0510071.1

Herszfeld, D. et al. Nat Biotechnol 24, 351-357 (2006)
http://dx.doi.org/10.1038/nbt1197

Hewitt, Z. et al., Cloning Stem Cells 8, 225-234 (2006)
http://dx.doi.org/10.1089/clo.2006.8.225

Hirst, C. E. et al. Dev Biol 293, 90-103 (2006)
http://dx.doi.org/10.1016/j.ydbio.2006.01.023

Huang, H. et al. Biochem Biophys Res Commun 351, 321-327 (2006)
http://dx.doi.org/10.1016/j.bbrc.2006.09.171

Huettner, J. E. et al., Stem Cells 24, 1654-1667 (2006)
http://dx.doi.org/10.1634/stemcells.2005-0003

Hwang, N. S. et al., Tissue Eng 12, 2695-2706 (2006)
http://dx.doi.org/10.1089/ten.2006.12.2695

Imreh, M. P. et al., J Cell Biochem 99, 508-516 (2006)
http://dx.doi.org/10.1002/jcb.20897

Inniss, K. & Moore, H., Stem Cells Dev 15, 789-796 (2006)
http://dx.doi.org/10.1089/scd.2006.15.789

Iuchi, S. et al., Differentiation 74, 160-166 (2006)
http://dx.doi.org/10.1111/j.1432-0436.2006.00067.x

Iuchi, S. et al., Proc Natl Acad Sci U S A 103, 1792-1797 (2006)
http://dx.doi.org/10.1073/pnas.0510953103

James, D. et al., Dev Biol 295, 90-102 (2006)
http://dx.doi.org/10.1016/j.ydbio.2006.03.026

Jang, J. E. et al., Stem Cells Dev 15, 109-117 (2006)
http://dx.doi.org/10.1089/scd.2006.15.109

Ji, L. et al., Tissue Eng 12, 665-679 (2006)
http://dx.doi.org/10.1089/ten.2006.12.665

2006 Joannides, A. et al., Stem Cells 24, 230-235 (2006)
http://dx.doi.org/10.1634/stemcells.2005-0243

Josephson, R. et al., BMC Biol 4, 28 (2006)
http://dx.doi.org/10.1186/1741-7007-4-28

Kameda, T. et al., Biochem Biophys Res Commun 349, 1269-1277 (2006)
http://dx.doi.org/10.1016/j.bbrc.2006.08.175

Karp, J. M. et al. Stem Cells 24, 835-843 (2006)
http://dx.doi.org/10.1634/stemcells.2005-0383

Katkov, I. I. et al., Cryobiology 53, 194-205 (2006)
http://dx.doi.org/10.1016/j.cryobiol.2006.05.005

Kee, K. et al., Stem Cells Dev 15, 831-837 (2006)
http://dx.doi.org/10.1089/scd.2006.15.831

Khademhosseini, A. et al. Biomaterials 27, 5968-5977 (2006)
http://dx.doi.org/10.1016/j.biomaterials.2006.06.035

Kim, B. K. et al., FEBS Lett 580, 5869-5874 (2006)
http://dx.doi.org/10.1016/j.febslet.2006.09.053

Kim, C. G. et al., Mol Cells 21, 343-355 (2006)
http://molcells.inforang.com/article_pdf/Ksmcb/21/Ksmcb21-3-4.pdf

Kim, S. J. et al., Acta Haematol 116, 219-222 (2006)
http://dx.doi.org/10.1159/000094687

Kim, S. K. et al., Toxicol Sci 94, 310-321 (2006)
http://dx.doi.org/10.1093/toxsci/kfl114

Klimanskaya, I. et al., Nature 444, 481-485 (2006)
http://dx.doi.org/10.1038/nature05142

Koch, P et al., O. Nucleic Acids Res 34, e120 (2006)
http://dx.doi.org/10.1093/nar/gkl674

Kofidis, T. et al. Eur J Cardiothorac Surg 29, 50-55 (2006)
http://dx.doi.org/10.1016/j.ejcts.2005.10.015

Lagarkova, M. A. et al., Cell Cycle 5, 416-420 (2006)
http://www.landesbioscience.com/journals/6/article/2440/

Lamba, D. A. et al., Proc Natl Acad Sci U S A 103, 12769-12774 (2006)
http://dx.doi.org/10.1073/pnas.0601990103

Lavon, N. et al., Stem Cells 24, 1923-1930 (2006)
http://dx.doi.org/10.1634/stemcells.2005-0397

Lee, D. H. et al., J Neurosurg 105, 127-133 (2006)
http://dx.doi.org/10.3171/ped.2006.105.2.127

2006 Lee, D. S. et al., Life Sci 80, 154-159 (2006)
http://dx.doi.org/10.1016/j.lfs.2006.08.039

Lee, J. et al., J Biol Chem 281, 33554-33565 (2006)
http://dx.doi.org/10.1074/jbc.M603937200

Lee, S. J. et al., Biomaterials 27, 3466-3472 (2006)
http://dx.doi.org/10.1016/j.biomaterials.2006.01.059

Lee, T. I. et al. Cell 125, 301-313 (2006)
http://dx.doi.org/10.1016/j.cell.2006.02.043

Levenstein, M. E. et al. Stem Cells 24, 568-574 (2006)
http://dx.doi.org/10.1634/stemcells.2005-0247

Levine, A. J. & Brivanlou, A. H., Development 133, 209-216 (2006)
http://dx.doi.org/10.1242/dev.02192

Li, H. et al. BMC Genomics 7, 103 (2006)
http://dx.doi.org/10.1186/1471-2164-7-103

Li, O. et al., Genesis 44, 523-529 (2006)
http://dx.doi.org/10.1002/dvg.20242

Li, S. S. et al. Stem Cells Dev 15, 532-555 (2006)
http://dx.doi.org/10.1089/scd.2006.15.532

Li, Y.J., et al., J Biomed Mater Res A 79, 1-5 (2006)
http://dx.doi.org/10.1002/jbm.a.30732

Lim, U. M. et al., Curr Neurovasc Res 3, 281-288 (2006)
http://dx.doi.org/10.2174/156720206778792902

Liu, Y. et al., Biochem Biophys Res Commun 346, 131-139 (2006)
http://dx.doi.org/10.1016/j.bbrc.2006.05.086

Liu, Y. et al., BMC Dev Biol 6, 20 (2006)
http://dx.doi.org/10.1186/1471-213X-6-20

Lowell, S. et al., PLoS Biol 4, e121 (2006)
http://dx.doi.org/10.1371/journal.pbio.0040121

Lu, J., et al., Proc Natl Acad Sci U S A 103, 5688-5693 (2006)
http://dx.doi.org/10.1073/pnas.0601383103

Ludwig, T. E. et al., Nat Biotechnol 24, 185-187 (2006)
http://dx.doi.org/10.1038/nbt1177

Ludwig, T. E. et al., Nat Methods 3, 637-646 (2006)
http://dx.doi.org/10.1038/nmeth902

Lund, R. D. et al., Cloning Stem Cells 8, 189-199 (2006)
http://dx.doi.org/10.1089/clo.2006.8.189

2006 Luo, Y. et al., Stem Cells 24, 865-875 (2006)
http://dx.doi.org/10.1634/stemcells.2005-0392

Lysdahl, H. et al. Reprod Biomed Online 12, 119-126 (2006)
http://www.rbmonline.com/4DCGI/Article/Detail?38%091%09=%201969%09

Mallon, B. S. et al., Int J Biochem Cell Biol 38, 1063-1075 (2006)
http://dx.doi.org/10.1016/j.biocel.2005.12.014

Mandal, A. et al., Differentiation 74, 81-90 (2006)
http://dx.doi.org/10.1111/j.1432-0436.2006.00051.x

Martinat, C. et al. Proc Natl Acad Sci U S A 103, 2874-2879 (2006)
http://dx.doi.org/10.1073/pnas.0511153103

Mateizel, I. et al. Hum Reprod 21, 503-511 (2006)
http://dx.doi.org/10.1093/humrep/dei345

Mikkola, M. et al. BMC Dev Biol 6, 40 (2006)
http://dx.doi.org/10.1186/1471-213X-6-40

Mohr, J. C. et al., Biomaterials 27, 6032-6042 (2006)
http://dx.doi.org/10.1016/j.biomaterials.2006.07.012

Mohr, J. C. et al., Biotechnol Prog 22, 825-834 (2006)
http://dx.doi.org/10.1021/bp0600334

Morris, G. J. et al. Reprod Biomed Online 13, 421-426 (2006)
http://www.rbmonline.com/4DCGI/Article/Detail?38%091%09=%202331%09

Narayan, A. D. et al. Blood 107, 2180-2183 (2006)
http://dx.doi.org/10.1182/blood-2005-05-1922

Nasonkin, I. O. & Koliatsos, V. E., Exp Neurol 201, 525-529 (2006)
http://dx.doi.org/10.1016/j.expneurol.2006.05.002

Noggle, S. A. et al., Stem Cells 24, 1646-1653 (2006)
http://dx.doi.org/10.1634/stemcells.2005-0314

Nolden, L. et al., Nat Methods 3, 461-467 (2006)
http://dx.doi.org/10.1038/nmeth884

Norstrom, A. et al., Exp Biol Med (Maywood) 231, 1753-1762 (2006)
http://www.ebmonline.org/cgi/content/abstract/231/11/1753

Olivier, E. N. et al., Exp Hematol 34, 1635-1642 (2006)
http://dx.doi.org/10.1016/j.exphem.2006.07.003

Olivier, E. N. et al., Stem Cells 24, 1914-1922 (2006)
http://dx.doi.org/10.1634/stemcells.2005-0648

Peng, H. M. & Chen, G., Hum Reprod 21, 217-22 (2006)
http://dx.doi.org/10.1093/humrep/dei275

2006 Plaia, T. W. et al., Stem Cells 24, 531-546 (2006)
http://dx.doi.org/10.1634/stemcells.2005-0315

Player, A. et al., Stem Cells Dev 15, 315-323 (2006)
http://dx.doi.org/10.1089/scd.2006.15.315

Poon, E. et al., J Cell Sci 119, 759-768 (2006)
http://dx.doi.org/10.1242/jcs.02788

Postovit, L. M. et al., Stem Cells 24, 501-505 (2006)
http://dx.doi.org/10.1634/stemcells.2005-0459

Pyle, A. D. et al., Nat Biotechnol 24, 344-350 (2006)
http://dx.doi.org/10.1038/nbt1189

Ren, C. et al. Stem Cells 24, 1338-1347 (2006)
http://dx.doi.org/10.1634/stemcells.2005-0338

Rho, J. Y. et al., Hum Reprod 21, 405-412 (2006)
http://dx.doi.org/10.1093/humrep/dei328

Richards, M. et al., Stem Cells 24, 1162-1173 (2006)
http://dx.doi.org/10.1634/stemcells.2005-0304

Roy, N. S. et al. Nat Med 12, 1259-1268 (2006)
http://dx.doi.org/10.1038/nm1495

Saha, S. et al., J Cell Physiol 206, 126-137 (2006)
http://dx.doi.org/10.1002/jcp.20441

Samadikuchaksaraei, A. et al., Tissue Eng 12, 867-875 (2006)
http://dx.doi.org/10.1089/ten.2006.12.867

Shen, Y. et al., Hum Mol Genet 15, 2623-2635 (2006)
http://dx.doi.org/10.1093/hmg/ddl188

Shin, S. et al., Stem Cells 24, 125-138 (2006)
http://dx.doi.org/10.1634/stemcells.2004-0150

Sidhu, K. S. & Tuch, B. E. Stem Cells Dev 15, 61-69 (2006)
http://dx.doi.org/10.1089/scd.2006.15.61

Sidhu, K. S. et al., Stem Cells Dev 15, 741-747 (2006)
http://dx.doi.org/10.1089/scd.2006.15.741

Skottman, H. et al., Stem Cells 24, 151-167 (2006)
http://dx.doi.org/10.1634/stemcells.2004-0189

Slukvin, I. I. et al., J Immunol 176, 2924-2932 (2006)
http://www.jimmunol.org/cgi/content/abstract/176/5/2924

Soto-Gutierrez, A. et al., Cell Transplant 15, 335-341 (2006)
http://dx.doi.org/10.3727/000000006783981945

2006 Stewart, M.H. et al., Nat Methods 3, 807-815 (2006)
http://dx.doi.org/10.1038/nmeth939

Strelchenko, N. et al., Reprod Biomed Online 12, 107-111 (2006)
http://www.rbmonline.com/4DCGI/Article/Detail?38%091%09=%202071%09

Suemori, H. et al., Biochem Biophys Res Commun 345, 926-932 (2006)
http://dx.doi.org/10.1016/j.bbrc.2006.04.135

Sun, B. W. et al. Hum Mol Genet 15, 65-75 (2006)
http://dx.doi.org/10.1093/hmg/ddi427

Suter, D. M. et al. Stem Cells 24, 615-623 (2006)
http://dx.doi.org/10.1634/stemcells.2005-0226

Tallheden, T. et al., Life Sci 79, 999-1006 (2006)
http://dx.doi.org/10.1016/j.lfs.2006.05.021

Taylor, R. A. et al., Nat Methods 3, 179-181 (2006)
http://dx.doi.org/10.1038/nmeth855

Tian, X. et al., Stem Cells 24, 1370-1380 (2006)
http://dx.doi.org/10.1634/stemcells.2005-0340

Trivedi, H.L. et al. Transplant Proc 38, 3103-3108 (2006)
http://dx.doi.org/10.1016/j.transproceed.2006.08.173

Tuve, S. et al., J Virol 80, 12109-12120 (2006)
http://dx.doi.org/10.1128/JVI.01370-06

Tzukerman, M. et al. Cancer Res 66, 3792-3801 (2006)
http://dx.doi.org/10.1158/0008-5472.CAN-05-3467

Ueno, M. et al. Proc Natl Acad Sci U S A 103, 9554-9559 (2006)
http://dx.doi.org/10.1073/pnas.0600104103

Valbuena, D. et al. Reprod Biomed Online 13, 875-886 (2006)
http://www.rbmonline.com/4DCGI/Article/Detail?38%091%09=%202516%09

Van Hoof, D. et al. Mol Cell Proteomics 5, 1261-1273 (2006)
http://dx.doi.org/10.1074/mcp.M500405-MCP200

Vats, A. et al., Tissue Eng 12, 1687-1697 (2006)
http://dx.doi.org/10.1089/ten.2006.12.1687

Verlinsky, Y. et al. Reprod Biomed Online 13, 547-550 (2006)
http://www.rbmonline.com/4DCGI/Article/Detail?38%091%09=%202437%09

Vodyanik, M. A. et al., Blood 108, 2095-2105 (2006)
http://dx.doi.org/10.1182/blood-2006-02-003327

Wang, T. W. et al. J Comp Neurol 497, 88-100 (2006)
http://dx.doi.org/10.1002/cne.20984

2006 Ward, C. M. et al., Exp Cell Res 312, 1713-1726 (2006)
http://dx.doi.org/10.1016/j.yexcr.2006.02.006

Ware, C. B. et al, Stem Cells 24, 2677-2684 (2006)
http://dx.doi.org/10.1634/stemcells.2005-0452

Wearne, K. A. et al., Glycobiology 16, 981-990 (2006)
http://dx.doi.org/10.1093/glycob/cwl019

Willems, E. et al., Int J Dev Biol 50, 627-635 (2006)
http://dx.doi.org/10.1387/ijdb.052130ew

Wong, R. C. B. et al., Biochem Biophys Res Commun 344, 181-188 (2006)
http://dx.doi.org/10.1016/j.bbrc.2006.03.127

Xiao, L. et al., Stem Cells 24, 1476-1486 (2006)
http://dx.doi.org/10.1634/stemcells.2005-0299

Xu, C. et al., Stem Cells Dev 15, 631-639 (2006)
http://dx.doi.org/10.1089/scd.2006.15.631

Xu, C. et al., Stem Cells Dev 15, 931-941 (2006)
http://dx.doi.org/10.1089/scd.2006.15.931

Xu, X. et al., Cloning Stem Cells 8, 96-107 (2006)
http://dx.doi.org/10.1089/clo.2006.8.96

Yao, S. et al., Proc Natl Acad Sci U S A 103, 6907-6912 (2006)
http://dx.doi.org/10.1073/pnas.0602280103

Yoon, B. S. et al., Differentiation 74, 149-159 (2006)
http://dx.doi.org/10.1111/j.1432-0436.2006.00063.x

Yu, J. et al., Stem Cells 24, 168-176 (2006)
http://dx.doi.org/10.1634/stemcells.2005-0292

Zeng, X. et al., Neuropsychopharmacology 31, 2708-2715 (2006)
http://dx.doi.org/10.1038/sj.npp.1301125

Zhang, J. et al., Nat Cell Biol 8, 1114-1123 (2006)
http://dx.doi.org/10.1038/ncb1481

Zhang, X. et al., Stem Cells, 24, 2669-2676 (2006)
http://dx.doi.org/10.1634/stemcells.2006-0377

Zhang, Y. W. et al., Stem Cells Dev 15, 943-952 (2006)
http://dx.doi.org/10.1089/scd.2006.15.943

Zheng, J. K. et al. Cell Res 16, 713-722 (2006)
http://dx.doi.org/10.1038/sj.cr.7310080

2007 Abhyankar, V. V. & Beebe D. J., Anal Chem 79, 4066-4073 (2007)
http://dx.doi.org/10.1021/ac062371p

2007 Adewumi, O. et al., Nat Biotechnol 25, 803-816 (2007)
http://dx.doi.org/10.1038/nbt1318

Ahmad, S. et al., Stem Cells 25, 1145-1155 (2007)
http://dx.doi.org/10.1634/stemcells.2006-0516

Allegrucci, C. et al., Hum Mol Genet 16, 1253-68 (2007)
http://dx.doi.org/10.1093/hmg/ddm074

Anderson, D. et al., Mol Ther, 15, 2027-2036 (2007)
http://dx.doi.org/10.1038/sj.mt.6300303

Anisimov, S. V. et al., BMC Genomics, 8, 46 (2007)
http://dx.doi.org/10.1186/1471-2164-8-46

Assou ,S. et al., Stem Cells, 25, 961-973 (2007)
http://dx.doi.org/10.1634/stemcells.2006-0352

Babaie, Y. et al., Stem Cells 25, 500-510 (2007)
http://dx.doi.org/10.1634/stemcells.2006-0426

Baharvand, H. et al., Int J Dev Biol, 51 (2007)
http://dx.doi.org/10.1387/ijdb.72280hb

Baker, D. E. et al., Nat Biotechnol 25, 207-215 (2007)
http://dx.doi.org/10.1038/nbt1285

Bakre, M. M. et al., J Biol Chem 282, 31703-31712 (2007)
http://dx.doi.org/10.1074/jbc.M704287200

Barberi, T. et al., Nat Med, 13, 642-648 (2007)
http://dx.doi.org/10.1038/nm1533

Becker, K. A. et al., J Cell Physiol, 210, 517-526 (2007)
http://dx.doi.org/10.1002/jcp.20903

Bendall, S. C. et al., Nature 448, 1015-1021 (2007)
http://dx.doi.org/10.1038/nature06027

Ben-Hur, T. et al., Magn Reson Med 57, 164-171 (2007)
http://dx.doi.org/10.1002/mrm.21116

Bettiol, E. et al., Differentiation 75, 669-681 (2007)
http://dx.doi.org/10.1111/j.1432-0436.2007.00174.x

Binah, O. et al., J Electrocardiol 40, 192-196 (2007)
http://dx.doi.org/10.1016/j.jelectrocard.2007.05.035

Biton, S. et al., DNA Repair (Amst) 6, 128-134 (2007)
http://dx.doi.org/10.1016/j.dnarep.2006.10.019

Blum, B. & Benvenisty N., Stem Cells 25, 1924-1930 (2007)
http://dx.doi.org/10.1634/stemcells.2007-0073

2007 Boyd, N. L. et al., Exp Biol Med (Maywood) 232, 833-843 (2007)
http://www.ebmonline.org/cgi/content/abstract/232/6/833

Bradbury, M. S. et al., J Neurochem 102, 2029-2039 (2007)
http://dx.doi.org/10.1111/j.1471-4159.2007.04681.x

Brimble, S. N. et al., Stem Cells 25, 54-62 (2007)
http://dx.doi.org/10.1634/stemcells.2006-0232

Brons, I. G. et al., Nature 448, 191-195 (2007)
http://dx.doi.org/10.1038/nature05950

Brown, B. D. et al., Nat Biotechnol 25, 1457-1467 (2007)
http://dx.doi.org/10.1038/nbt1372

Burridge, P. W. et al., Stem Cells, 25, 929-938 (2007)
http://dx.doi.org/10.1634/stemcells.2006-0598

Cabrera, C. M. et al., Cell Biol Int 31, 1072-1078 (2007)
http://dx.doi.org/10.1016/j.cellbi.2007.03.015

Cai J. et al., Hepatology 45, 1229-1239 (2007)
http://dx.doi.org/10.1002/hep.21582

Cai L. et al., Cell Res 17, 62-72 (2007)
http://dx.doi.org/10.1038/sj.cr.7310138

Carlson, M. E.& Conboy I. M., Aging Cell 6, 371-382 (2007)
http://dx.doi.org/10.1111/j.1474-9726.2007.00286.x

Caspi O. et al., Circ Res 100, 263-272 (2007)
http://dx.doi.org/10.1161/01.RES.0000257776.05673.ff

Caspi O. et al., J Am Coll Cardiol 50, 1884-1893 (2007)
http://dx.doi.org/10.1016/j.jacc.2007.07.054

Cedervall J. et al., Laryngoscope 117, 2075-2081 (2007)
http://dx.doi.org/10.1097/MLG.0b013e3181379c7c

Cezar, G. G. et al.,Stem Cells Dev 16, 869-882 (2007)
http://dx.doi.org/10.1089/scd.2007.0022

Chen D. et al., Exp Hematol 35, 1344-1357 (2007)
http://dx.doi.org/10.1016/j.exphem.2007.06.004

Chen, H. F. et al., Hum Reprod, 22, 567-577 (2007)
http://dx.doi.org/10.1093/humrep/del412

Chen, T. et al., Stem Cells 25, 392-401 (2007)
http://dx.doi.org/10.1634/stemcells.2006-0145

Chin, A. C. et al., J Biotechnol 130, 320-328 (2007)
http://dx.doi.org/10.1016/j.jbiotec.2007.04.013

2007 Cho, S. W. et al., Circulation 116, 2409-2419 (2007)
http://dx.doi.org/10.1161/CIRCULATIONAHA.106.687038

Choudhary, M. et al., Stem Cells 25, 3045-3057 (2007)
http://dx.doi.org/10.1634/stemcells.2007-0296

Conley, B. J. et al., Biochem Cell Biol 85, 121-132(2007)
http://dx.doi.org/10.1139/o06-145

Crook, V. V. et al., Cell Stem Cell 1, 490-494 (2007)
http://dx.doi.org/10.1016/j.stem.2007.10.004

Cui, L. et al., J Struct Bio 158, 307-317 (2007)
http://dx.doi.org/10.1016/j.jsb.2006.11.009

Dai, W. et al., J Mol Cell Cardiol 43, 504-516 (2007)
http://dx.doi.org/10.1016/j.yjmcc.2007.07.001

Das, P. et al., Stem Cell Res 1, 61-74 (2007)
http://dx.doi.org/10.1016/j.scr.2007.09.004

Davidson, K. C. et al., Mol Cell Neurosci 36, 408-415 (2007)
http://dx.doi.org/10.1016/j.mcn.2007.07.013

Denham, M. et al., Am J Physiol Lung Cell Mol Physiol 292, L1241-1247 (2007)
http://dx.doi.org/10.1152/ajplung.00440.2006

Derda, R. et al., ACS Chem Biol 2, 347-355 (2007)
http://dx.doi.org/10.1021/cb700032u

Duan, Y. et al., Stem Cells 25, 3058-3068 (2007)
http://dx.doi.org/10.1634/stemcells.2007-0291

Dvash, T. et al., Stem Cells 25, 465-472 (2007)
http://dx.doi.org/10.1634/stemcells.2006-0179

Eastham, A. M. et al., Cancer Res 67, 11254-11562 (2007)
http://dx.doi.org/10.1158/0008-5472.CAN-07-2253

Egozi, D. et al., Faseb J 21, 2807-2817 (2007)
http://dx.doi.org/10.1096/fj.06-7758com

Eiges, R. et al., Cell Stem Cell 1, 568-577 (2007)
http://dx.doi.org/10.1016/j.stem.2007.09.001

Ek, M. et al., Biochem Pharmacol 74, 496-503 (2007)
http://dx.doi.org/10.1016/j.bcp.2007.05.009

Ellerström, C. et al., Stem Cells 25, 1690-1696 (2007)
http://dx.doi.org/10.1634/stemcells.2006-0607

Ferreira, L. S. et al., Biomaterials 28, 2706-2717 (2007)
http://dx.doi.org/10.1016/j.biomaterials.2007.01.021

2007 Ferreira, L. S. et al., Circ Res 101, 286-294 (2007)
http://dx.doi.org/10.1161/CIRCRESAHA.107.150201

Figallo, E. et al., Lab Chip 7, 710-719 (2007)
http://dx.doi.org/10.1039/b700063d

Filipczyk, A. A. et al., Cell Mol Life Sci 64, 704-718 (2007)
http://dx.doi.org/10.1007/s00018-007-6523-2

Follows, G. A. et al., Nucleic Acids Res 35, e56 (2007)
http://dx.doi.org/10.1093/nar/gkm108

Fox, M. S. et al., Dev Biol 301, 417-431 (2007)
http://dx.doi.org/10.1016/j.ydbio.2006.08.047

Frandsen, U. et al., Biochem Biophys Res Commun, 362, 568-574 (2007)
http://dx.doi.org/10.1016/j.bbrc.2007.07.200

Garcia-Perez, J. L. et al., Hum Mol Genet, 16, 1569-1577 (2007)
http://dx.doi.org/10.1093/hmg/ddm105

Gauthaman, K. et al., Reprod Biomed Online 15, 566-581 (2007)
http://www.rbmonline.com/4DCGI/Article/Detail?38%091%09=%202962%09

Gerecht S. et al., Proc Natl Acad Sci U S A 104, 11298-11303 (2007)
http://dx.doi.org/10.1073/pnas.0703723104

Gerecht, S. et al., Biomaterials 28, 4068-4077 (2007)
http://dx.doi.org/10.1016/j.biomaterials.2007.05.027

Gerecht, S. et al., Biomaterials 28, 4826-4835 (2007)
http://dx.doi.org/10.1016/j.biomaterials.2007.07.039

Gertow, K. et al., J Cell Biochem 100, 1518-1525 (2007)
http://dx.doi.org/10.1002/jcb.21144

Gharwan, H. et al., Mol Ther 15, 1827-1833 (2007)
http://dx.doi.org/10.1038/sj.mt.6300244

Ghule, P. N. et al., J Cell Physiol 213, 9-17 (2007)
http://dx.doi.org/10.1002/jcp.21119

Greber, B. et al., BMC Dev Biol 7, 46 (2007)
http://dx.doi.org/10.1186/1471-213X-7-46

Greber, B. et al., Stem Cells 25, 455-464 (2007)
http://dx.doi.org/10.1634/stemcells.2006-0476

Greco, S. J. et al., Stem Cells 25, 3143-3154 (2007)
http://dx.doi.org/10.1634/stemcells.2007-0351

Gropp, M. & Reubinoff E., Cloning Stem Cells 9, 339-345 (2007)
http://dx.doi.org/10.1089/clo.2006.0077

2007 Grskovic, M. et al., PLoS Genet 3, e145 (2007)
http://dx.doi.org/10.1371/journal.pgen.0030145

Guenther, M. G. et al., Cell 130, 77-88 (2007)
http://dx.doi.org/10.1016/j.cell.2007.05.042

Hasegawa, K. et al., Stem Cells 25, 1707-1712 (2007)
http://dx.doi.org/10.1634/stemcells.2006-0813

Hay, D. C. et al., Cloning Stem Cells 9, 51-62 (2007)
http://dx.doi.org/10.1089/clo.2006.0045

Heiskanen, A. et al., Stem Cells 25, 197-202 (2007)
http://dx.doi.org/10.1634/stemcells.2006-0444

Hellman, A. et al., Science 315, 1141-1143 (2007)
http://dx.doi.org/10.1126/science.1136352

Heng, B. C. et al., Biosci Rep 27, 257-264 (2007)
http://dx.doi.org/10.1007/s10540-007-9051-2

Heng, B. C. et al., Biotechnol Appl Biochem 47, 33-37 (2007)
http://dx.doi.org/10.1042/BA20060151

Hewitt, Z. et al., Stem Cells 25, 10-18 (2007)
http://dx.doi.org/10.1634/stemcells.2005-0481

Hirst, M. et al., Genome Biol 8, R113 (2007)
http://dx.doi.org/10.1186/gb-2007-8-6-r113

Huang, G. et al., Chin Med J (Engl), 120, 589-594 (2007)
http://www.cmj.org/periodical/PaperList.asp?id=LW2007455546876044O5

Huang, Y. et al., Hybridoma (Larchmt) 26, 387-391 (2007)
http://dx.doi.org/10.1089/hyb.2007.0517

Huang, Z. et al., J Cell Physiol 211, 816-825 (2007)
http://dx.doi.org/10.1002/jcp.20985

Huber, I. et al., Faseb J 21, 2551-2563 (2007)
http://dx.doi.org/10.1096/fj.05-5711com

Iacovitti, L. et al., Brain Res 1127, 19-25 (2007)
http://dx.doi.org/10.1016/j.brainres.2006.10.022

Inanc, B. et al., Artif Organs 31, 792-800 (2007)
http://dx.doi.org/10.1111/j.1525-1594.2007.00470.x

Irion, S. et al., Nat Biotechnol 25, 1477-1482 (2007)
http://dx.doi.org/10.1038/nbt1362

Izrael, M. et al., Mol Cell Neurosci 34, 310-323 (2007)
http://dx.doi.org/10.1016/j.mcn.2006.11.008

2007 Jiang, J. et al., Stem Cells 25, 1940-1953 (2007)
http://dx.doi.org/10.1634/stemcells.2006-0761

Jiang, W. et al., Cell Res 17, 333-344 (2007)
http://dx.doi.org/10.1038/cr.2007.28

Joannides, A. J. et al., Brain 130, 1263-1275 (2007)
http://dx.doi.org/10.1093/brain/awm070

Joannides, A. J. et al., Stem Cells 25, 731-737 (2007)
http://dx.doi.org/10.1634/stemcells.2006-0562

Johnson, M. A. et al., J Neurosci 27, 3069-3077 (2007)
http://dx.doi.org/10.1523/JNEUROSCI.4562-06.2007

Josephson, R. et al., Stem Cells 25, 437-446 (2007)
http://dx.doi.org/10.1634/stemcells.2006-0236

Kang, H. B. et al., Stem Cells Dev 16, 615-623 (2007)
http://dx.doi.org/10.1089/scd.2007.0014

Kang, S. M. et al., Stem Cells 25, 419-424 (2007)
http://dx.doi.org/10.1634/stemcells.2005-0482

Kärner, E. et al., Stem Cells Dev 16, 39-52 (2007)
http://dx.doi.org/10.1089/scd.2007.16.ft-1

Kennedy, M. et al., Blood 109, 2679-2687 (2007)
http://dx.doi.org/10.1182/blood-2006-09-047704

Kim, D. Y. et al., Neurosci Res 58, 164-175 (2007)
http://dx.doi.org/10.1016/j.neures.2007.02.016

Kim, J. et al., Stem Cells Dev 16, 269-280 (2007)
http://dx.doi.org/10.1089/scd.2006.0108

Kim, K. et al., Cell Stem Cell 1, 346-352 (2007)
http://dx.doi.org/10.1016/j.stem.2007.07.001

Kim, K. P. et al., Genome Res 17, 1731-1742 (2007)
http://dx.doi.org/10.1101/gr.6609207

Kim, M. S. et al., Lab Chip 7, 513-515 (2007)
http://dx.doi.org/10.1039/b617760n

Kim, S. E. et al., Mol Cells 23, 49-56 (2007)
http://molcells.inforang.com/article_pdf/Ksmcb/23/Ksmcb23-1-7.pdf

Kim, S. et al., Stem Cells 25, 2601-2609 (2007)
http://dx.doi.org/10.1634/stemcells.2006-0814

Kim, S. et al., Stem Cells Dev 16, 537-545 (2007)
http://dx.doi.org/10.1089/scd.2006.0088

2007 Kim, S. J. et al., Stem Cells Dev 16, 421-428 (2007)
http://dx.doi.org/10.1089/scd.2006.0098

Ko, J. Y. et al., J Neurochem 103, 1417-1429 (2007)
http://dx.doi.org/10.1111/j.1471-4159.2007.04898.x

Koay, E. J. et al., Stem Cells 25, 2183-2190 (2007)
http://dx.doi.org/10.1634/stemcells.2007-0105

Krtolica, A. et al., Stem Cells 25, 2215-2223 (2007)
http://dx.doi.org/10.1634/stemcells.2007-0230

Kumar, M. et al., Stem Cells Dev 16, 667-681 (2007)
http://dx.doi.org/10.1089/scd.2006.0115

Laflamme, M. A. et al., Nat Biotechnol 25, 1015-1024 (2007)
http://dx.doi.org/10.1038/nbt1327

Lakshmipathy, U. et al., Stem Cells Dev 16, 1003-1016 (2007)
http://dx.doi.org/10.1089/scd.2007.0026

Laslett, A. L. et al., BMC Dev Biol 7, 12 (2007)
http://dx.doi.org/10.1186/1471-213X-7-12

Laursen, S. B. et al., Reprod Biomed Online,15, 89-98 (2007)
http://www.rbmonline.com/4DCGI/Article/Detail?38%091%09=%202790%09

Lee, G. et al., Nat Biotechnol 25, 1468-1475 (2007)
http://dx.doi.org/10.1038/nbt1365

Lee, H. et al., Stem Cells 25, 1931-1939 (2007)
http://dx.doi.org/10.1634/stemcells.2007-0097

Lee, J. P. et al., Nat Med 13, 439-447 (2007)
http://dx.doi.org/10.1038/nm1548

Lee, M. H. et al., PLoS Genet 3, e233 (2007)
http://dx.doi.org/10.1371/journal.pgen.0030233

Lees, J. G. et al., Regen Med 2, 289-300 (2007)
http://dx.doi.org/10.2217/17460751.2.3.289

Leor, J. et al., Heart 93, 1278-1284 (2007)
http://dx.doi.org/10.1136/hrt.2006.093161

Li, J. et al., Differentiation 75, 299-307 (2007)
http://dx.doi.org/10.1111/j.1432-0436.2006.00143.x

Li, O. et al., FEBS Lett 581, 3533-3537 (2007)
http://dx.doi.org/10.1016/j.febslet.2007.06.072

Lian, Q. et al., Stem Cells 25, 425-436 (2007)
http://dx.doi.org/10.1634/stemcells.2006-0420

2007 Liew, C. G. et al., Stem Cells 25, 1521-1528 (2007)
http://dx.doi.org/10.1634/stemcells.2006-0634

Lim, L. S. et al., Mol Biol Cell 18, 1348-58 (2007)
http://dx.doi.org/10.1091/mbc.E06-07-0624

Liu, J. et al., Stem Cells 25, 3038-3044 (2007)
http://dx.doi.org/10.1634/stemcells.2007-0549

Lombardo, A. et al., Nat Biotechnol 25, 1298-1306 (2007)
http://dx.doi.org/10.1038/nbt1353

Lu, S. J. et al., Genome Biol 8, R240 (2007)
http://dx.doi.org/10.1186/gb-2007-8-11-r240

Lu, S. J. et al., Nat Methods 4, 501-509 (2007)
http://dx.doi.org/10.1038/nmeth1041

Lu, S. J. et al., Stem Cells Dev 16, 547-559 (2007)
http://dx.doi.org/10.1089/scd.2007.0002

Ma, F. et al., Int J Hematol 85, 371-379 (2007)
http://dx.doi.org/10.1532/IJH97.06203

Mantel, C. et al., Blood 109, 4518-4527 (2007)
http://dx.doi.org/10.1182/blood-2006-10-054247

McLean, A. B. et al., Stem Cells 25, 29-38 (2007)
http://dx.doi.org/10.1634/stemcells.2006-0219

Narkilahti, S. et al., Biomed Eng Online 6, 11 (2007)
http://dx.doi.org/10.1186/1475-925X-6-11

Nat, R. et al., Glia 55, 385-99 (2007)
http://dx.doi.org/10.1002/glia.20463

Nicholas, C. R. et al., Stem Cells Dev 16,109-117 (2007)
http://dx.doi.org/10.1089/scd.2006.0059

Nieto, A. et al., Cell Biol Int 31, 269-278 (2007)
http://dx.doi.org/10.1016/j.cellbi.2006.11.006

Ohm, J. E. et al, Nat Genet 39, 237-242 (2007)
http://dx.doi.org/10.1038/ng1972

Okamura, R. M. et al., J Neuroimmunol 192, 1-2 (2007)
http://dx.doi.org/10.1016/j.jneuroim.2007.09.030

Ozolek, J. A. et al., Stem Cells Dev 16, 134-144 (2007)
http://dx.doi.org/10.1089/scd.2006.0109

Pajerowski J. D. et al., Proc Natl Acad Sci U S A 104, 15619-15624 (2007)
http://dx.doi.org/10.1073/pnas.0702576104

2007 Pal, R. & Khanna A., Differentiation 75, 112-122 (2007)
http://dx.doi.org/10.1111/j.1432-0436.2006.00123.x

Pal, R. et al., Regen Med 2, 179-192 (2007)
http://dx.doi.org/10.2217/17460751.2.2.179

Pan, G. et al., Cell Stem Cell 1, 299-312 (2007)
http://dx.doi.org/10.1016/j.stem.2007.08.003

Pankratz, M. T. et al., Stem Cells 25, 1511-1520 (2007)
http://dx.doi.org/10.1634/stemcells.2006-0707

Peerani, R. et al., Embo J 26, 4744-4755 (2007)
http://dx.doi.org/10.1038/sj.emboj.7601896

Peiffer, I. et al., Stem Cells Dev 16, 393-402 (2007)
http://dx.doi.org/10.1089/scd.2006.0013

Peura, T. T. et al., Theriogenology 67, 32-42 (2007)
http://dx.doi.org/10.1016/j.theriogenology.2006.09.031

Phillips, B. W. et al., Stem Cells Dev 16, 561-578 (2007)
http://dx.doi.org/10.1089/scd.2007.0029

Pick, M. et al., Stem Cells 25, 2206-2214 (2007)
http://dx.doi.org/10.1634/stemcells.2006-0713

Pillekamp, F. et al., Stem Cells 25, 174-180 (2007)
http://dx.doi.org/10.1634/stemcells.2006-0094

Porayette, P. et al., Biochem Biophys Res Commun 364, 522-527 (2007)
http://dx.doi.org/10.1016/j.bbrc.2007.10.021

Prokhorovich, M. A. et al., Bull Exp Biol Med 144, 126-129 (2007)
http://dx.doi.org/10.1007/s10517-007-0271-z

Pruszak, J. et al., Stem Cells 25, 2257-2268 (2007)
http://dx.doi.org/10.1634/stemcells.2006-0744

Qin, H. et al., J Biol Chem 282, 5842-5852 (2007)
http://dx.doi.org/10.1074/jbc.M610464200

Rajala, K. et al., Hum Reprod 22, 1231-1238 (2007)
http://dx.doi.org/10.1093/humrep/del523

Rajesh, D. et al., Stem Cells 25, 490-499 (2007)
http://dx.doi.org/10.1634/stemcells.2006-0277

Rodriguez, R. T. et al., Exp Biol Med (Maywood) 232, 1368-1380 (2007)
http://dx.doi.org/10.3181/0703-RM-63

Rufaihah, A. J. et al., J Gene Med 9, 452-461 (2007)
http://dx.doi.org/10.1002/jgm.1034

2007 Sartiani, L. et al., Stem Cells 25, 1136-1144 (2007)
http://dx.doi.org/10.1634/stemcells.2006-0466

Saxe, J. P. et al., Chem Biol 14, 1019-1030 (2007)
http://dx.doi.org/10.1016/j.chembiol.2007.07.016

Schneider, B. L. et al., Hum Mol Genet 16, 651-666 (2007)
http://dx.doi.org/10.1093/hmg/ddm008

Schrattenholz, A. & Klemm M., Altex 24, 9-15 (2007)
http://www.altex.ch/de/index.html?id=49&iid=82&aid=4

Schulz, T. C. et al., BMC Genomics 8, 478 (2007)
http://dx.doi.org/10.1186/1471-2164-8-478

Senju, S. et al., Stem Cells 25, 2720-2729 (2007)
http://dx.doi.org/10.1634/stemcells.2007-0321

Serobyan, N. et al., Life Sc ,80, 2352-6230 (2007)
http://dx.doi.org/10.1016/j.lfs.2007.04.017

Shi, F. et al., Eur J Neurosci 26, 3016-3023 (2007)
http://dx.doi.org/10.1111/j.1460-9568.2007.05909.x

Shih, C. C. et al., Stem Cells Dev 16, 893-902 (2007)
http://dx.doi.org/10.1089/scd.2007.0070

Shim, J. H. et al., Diabetologia 50, 1228-1238 (2007)
http://dx.doi.org/10.1007/s00125-007-0634-z

Shin, S. et al., Stem Cells 25, 1298-1306 (2007)
http://dx.doi.org/10.1634/stemcells.2006-0660

Shin, S. et al., Stem Cells Dev 16, 131-141 (2007)
http://dx.doi.org/10.1089/scd.2006.0023

Siva, K. et al., PLoS ONE 2, e1202 (2007)
http://dx.doi.org/10.1371/journal.pone.0001202

Soderdahl, T. et al., Toxicol In Vitro 21, 929-937 (2007)
http://dx.doi.org/10.1016/j.tiv.2007.01.021

Soh, B. S. et al., Stem Cells 25, 3029-3037 (2007)
http://dx.doi.org/10.1634/stemcells.2007-0372

Son, Y. S. & Hong J., J Microbiol 45, 547-552 (2007)
http://www.msk.or.kr/jsp/recent_paper.jsp?paperSeq=2610

Sone, M. et al., Arterioscler Thromb Vasc Biol 27, 2127-2134 (2007)
http://dx.doi.org/10.1161/ATVBAHA.107.143149

Song, J. et al., Neurosci Lett 423, 58-61 (2007)
http://dx.doi.org/10.1016/j.neulet.2007.05.066

2007 Sonntag, K. C. et al., Stem Cells 25, 411-418 (2007)
http://dx.doi.org/10.1634/stemcells.2006-0380

Srivastava, A. S. et al., Stem Cells 25, 1456-1461 (2007)
http://dx.doi.org/10.1634/stemcells.2006-0701

Ström, S. et al., Hum Reprod 22, 3051-3058 (2007)
http://dx.doi.org/10.1093/humrep/dem335

Sullivan, K. E. et al., Mol Cell Biol 27, 5147-5160 (2007)
http://dx.doi.org/10.1128/MCB.02429-06

Sumi, T. et al., Oncogene 26, 5564-5576 (2007)
http://dx.doi.org/10.1038/sj.onc.1210353

Sun, Y. et al., Genomics 89, 22-35 (2007)
http://dx.doi.org/10.1016/j.ygeno.2006.09.010

Synnergren, J. et al., Stem Cells 25, 473-480 (2007)
http://dx.doi.org/10.1634/stemcells.2006-0247

Sze, S. K. et al., Mol Cell Proteomics 6, 1680-1689 (2007)
http://dx.doi.org/10.1074/mcp.M600393-MCP200

Takahashi, K. et al., Cell 131, 861-872 (2007)
http://dx.doi.org/10.1016/j.cell.2007.11.019

Tan, S. M. et al., Nucleic Acids Res 35, e118 (2007)
http://dx.doi.org/10.1093/nar/gkm704

Terraciano, V. et al., Stem Cells 25, 2730-2738 (2007)
http://dx.doi.org/10.1634/stemcells.2007-0228

Terstegge, S. et al., Biotechnol Bioeng 96, 195-201 (2007)
http://dx.doi.org/10.1002/bit.21061

Tesar, P. J. et al., Nature 448, 196-199 (2007)
http://dx.doi.org/10.1038/nature05972

Toh, W. S. et al., Stem Cells 25, 950-960 (2007)
http://dx.doi.org/10.1634/stemcells.2006-0326

Tomescot, A. et al., Stem Cells 25, 2200-2205 (2007)
http://dx.doi.org/10.1634/stemcells.2007-0133

Trigona, W. L. et al., Antioxid Redox Signal 9, 751-756 (2007)
http://dx.doi.org/10.1089/ars.2007.1602

Trivedi, P. et al., Exp Hematol 35, 146-154 (2007)
http://dx.doi.org/10.1016/j.exphem.2006.09.003

Ullmann, U. et al., Mol Hum Reprod 13, 21-32 (2007)
http://dx.doi.org/10.1093/molehr/gal091

2007 Vallier, L. et al., Stem Cell ,25, 1490-1497 (2007)
http://dx.doi.org/10.1634/stemcells.2006-0825

van Harmelen, V. et al., Obesity (Silver Spring) 15, 846-852 (2007)
http://dx.doi.org/10.1038/oby.2007.595

Vanikar, A. V. et al., Transplant Proc 39, 658-661 (2007)
http://dx.doi.org/10.1016/j.transproceed.2007.01.048

Vieyra, D. S. et al., Stem Cells 25, 2559-2566 (2007)
http://dx.doi.org/10.1634/stemcells.2007-0248

Wang, D. et al., Proc Natl Acad Sci U S A 104, 4449-4454 (2007)
http://dx.doi.org/10.1073/pnas.0700052104

Wang, L. et al., Blood 110, 4111-4119 (2007)
http://dx.doi.org/10.1182/blood-2007-03-082586

Wang, Z. X. et al., Stem Cells 25, 2173-2182 (2007)
http://dx.doi.org/10.1634/stemcells.2007-0085

Wang, Z. Z. et al., Nat Biotechnol 25, 317-318 (2007)
http://dx.doi.org/10.1038/nbt1287

Watanabe, K. et al., Nat Biotechnol 25, 681-686 (2007)
http://dx.doi.org/10.1038/nbt1310

Wilber, A. et al., Stem Cells 25, 2919-2927 (2007)
http://dx.doi.org/10.1634/stemcells.2007-0026

Wilson, P. G. et al., Stem Cells Dev 16, 1027-1041 (2007)
http://dx.doi.org/10.1089/scd.2007.0061

Wolvetang, E. J. et al., Biochem Biophys Res Commun 363, 610-615 (2007)
http://dx.doi.org/10.1016/j.bbrc.2007.09.035

Wong, R. C. et al., Stem Cells Dev 16, 989-1001 (2007)
http://dx.doi.org/10.1089/scd.2007.0057

Wu, H. et al., Proc Natl Acad Sci U S A 104, 13821-13826 (2007)
http://dx.doi.org/10.1073/pnas.0706199104

Xia, X. et al., Stem Cells Dev 16, 167-176 (2007)
http://dx.doi.org/10.1089/scd. 2006.0057

Xie, C. Q. et al., Arterioscler Thromb Vasc Biol 27, e311-312 (2007)
http://dx.doi.org/10.1161/ATVBAHA.107.154260

Xie, C. Q. et al., Stem Cells Dev 16, 25-29 (2007)
http://dx.doi.org/10.1089/scd.2006.110206

Yang, M. J. et al., Biomacromolecules 8, 2746-2752 (2007)
http://dx.doi.org/10.1021/bm0704166

2007 Yeo, G. W. et al., PLoS Comput Biol 3, 1951-1967 (2007)
http://dx.doi.org/10.1371/journal.pcbi.0030196

Yeo, S. et al., Biochem Biophys Res Commun 359, 536-542 (2007)
http://dx.doi.org/10.1016/j.bbrc.2007.05.120

Yu, J. et al., Science 318, 1917-1920 (2007)
http://dx.doi.org/10.1126/science.1151526

Zangrossi, S. et al., Stem Cells 25, 1675-1680 (2007)
http://dx.doi.org/10.1634/stemcells.2006-0611

Zeng, J. et al., Stem Cells 25, 1055-61 (2007)
http://dx.doi.org/10.1634/stemcells.2006-0616

Zhang, P. et al., Fertil Steril 87, 677-690 (2007)
http://dx.doi.org/10.1016/j.fertnstert.2006.07.1509

Zhao, M. et al., Biochem Biophys Res Commun 362, 916-922 (2007)
http://dx.doi.org/10.1016/j.bbrc.2007.08.081

Zhao, X. D. et al., Cell Stem Cell 1, 286-298 (2007)
http://dx.doi.org/10.1016/j.stem.2007.08.004

Zhong, J. F. et al., Gene Expr 14, 23-34 (2007)
http://www.ingentaconnect.com/content/cog/ge/2007/00000014/00000001/art00003

Zhou, B. Y. et al., Stem Cells 25, 779-789 (2007)
http://dx.doi.org/10.1634/stemcells.2006-0128

2008 Aasen, T. et al., Nat Biotechnol 26, 1276-1284 (2008)
http://dx.doi.org/10.1038/nbt.1503

Aberdam, E. et al., Stem Cells 26, 440-444 (2008)
http://dx.doi.org/10.1634/stemcells.2007-0588

Adler, S. et al., Altern Lab Anim 36, 129-140 (2008)
http://www.labmeeting.com/paper/28143887/adler-2008-testing-potential-developmental-to

Adler, S. et al., Toxicol In Vitro 22, 200-211 (2008)
http://dx.doi.org/10.1016/j.tiv.2007.07.013

Agarwal, S. et al., Stem Cells 26, 1117-1127 (2008)
http://dx.doi.org/10.1634/stemcells.2007-1102

Aharonowiz, M. et al., PLoS ONE 3, e3145 (2008)
http://dx.doi.org/10.1371/journal.pone.0003145

Ananiev, G. E. et al., BMC Mol Biol 9, 68 (2008)
http://dx.doi.org/10.1186/1471-2199-9-68

Andersson, M. K. et al., BMC Cell Biol 9, 37 (2008)
http://dx.doi.org/10.1186/1471-2121-9-37

2008 Apati, A. et al., Biochim Biophys Acta 1778, 2700-2709 (2008)
http://dx.doi.org/10.1016/j.bbamem.2008.08.010

Atkinson, S. P. et al., Stem Cells 26, 1174-1185 (2008)
http://dx.doi.org/10.1634/stemcells.2007-0497

Atlasi, Y. et al., Stem Cells 26, 3068-3074 (2008)
http://dx.doi.org/10.1634/stemcells.2008-0530

Aubry, L. et al., Proc Natl Acad Sci U S A 105, 16707-16712 (2008)
http://dx.doi.org/10.1073/pnas.0808488105

Avery, K. et al., Stem Cells Dev 17, 1195-1205 (2008)
http://dx.doi.org/10.1089/scd.2008.0063

Baharvand, H. et al., Differentiation 76, 465-477 (2008)
http://dx.doi.org/10.1111/j.1432-0436.2007.00252.x

Bajpai, R. et al., Mol Reprod Dev 75, 818-827 (2008)
http://dx.doi.org/10.1002/mrd.20809

Bandi, S. et al., AIDS Res Ther 5, 1 (2008)
http://dx.doi.org/10.1186/1742-6405-5-1

Banuelos, C. A. et al., DNA Repair (Amst) 7, 1471-1483 (2008)
http://dx.doi.org/10.1016/j.dnarep.2008.05.005

Bar, M. et al., Stem Cells 26, 2496-2505 (2008)
http://dx.doi.org/10.1634/stemcells.2008-0356

Barroso-delJesus, A. et al., Mol Cell Biol 28, 6609-6619 (2008)
http://dx.doi.org/10.1128/MCB.00398-08

Bartova, E. et al., Dev Dyn 237, 3690-3702 (2008)
http://dx.doi.org/10.1002/dvdy.21773

Bartova, E. et al., Differentiation 76, 24-32 (2008)
http://dx.doi.org/10.1111/j.1432-0436.2007.00192.x

Bauwens, C. L. et al., Stem Cells 26, 2300-2310 (2008)
http://dx.doi.org/10.1634/stemcells.2008-0183

Bendall, S. C. et al., Mol Cell Proteomics 7, 1587-1597 (2008)
http://dx.doi.org/10.1074/mcp.M800113-MCP200

Bera, T. K. et al., Stem Cells Dev 17, 325-332 (2008)
http://dx.doi.org/10.1089/scd.2007.0079

Bigdeli, N. et al., J Biotechnol 133, 146-153 (2008)
http://dx.doi.org/10.1016/j.jbiotec.2007.08.045

Bloushtain-Qimron, N. et al., Proc Natl Acad Sci U S A 105, 14076-14081 (2008)
http://dx.doi.org/10.1073/pnas.0805206105

2008 Bonig, H. et al., Transfusion 48, 1039-1040 (2008)
http://dx.doi.org/10.1111/j.1537-2995.2008.01706.x

Bonnefont, J. et al., Am J Hum Genet 83, 208-218 (2008)
http://dx.doi.org/10.1016/j.ajhg.2008.07.007

Braam, S. R. et al., Nat Methods 5, 389-392 (2008)
http://dx.doi.org/10.1038/nmeth.1200

Braam, S. R. et al., Stem Cells 26, 2257-2265 (2008)
http://dx.doi.org/10.1634/stemcells.2008-0291

Brink, T. C. et al., Cells Tissues Organs 188, 9-22 (2008)
http://dx.doi.org/10.1159/000112843

Brito-Martins, M. et al., Br J Pharmacol 153, 751-759 (2008)
http://dx.doi.org/10.1038/sj.bjp.0707619

Brokhman, I. et al., Differentiation 76, 145-155 (2008)
http://dx.doi.org/10.1111/j.1432-0436.2007.00196.x

Calvanese, V. et al., PLoS ONE 3, e3294 (2008)
http://dx.doi.org/10.1371/journal.pone.0003294

Cameron, C. M. et al., Exp Biol Med (Maywood) 233, 1044-1057 (2008)
http://dx.doi.org/10.3181/0709-RM-263

Cao, F. et al., PLoS ONE 3, e3474 (2008)
http://dx.doi.org/10.1371/journal.pone.0003474

Cao, H. et al., PLoS ONE 3, e2820 (2008)
http://dx.doi.org/10.1371/journal.pone.0002820

Cao, T. et al., Cloning Stem Cells 10, 1-10 (2008)
http://dx.doi.org/10.1089/clo.2007.0049

Card, D. A. et al., Mol Cell Biol 28, 6426-6438 (2008)
http://dx.doi.org/10.1128/MCB.00359-08

Catalina, P. et al., Mol Cancer 7, 76 (2008)
http://dx.doi.org/10.1186/1476-4598-7-76

Chan, E. M. et al., Cloning Stem Cells 10, 107-118 (2008)
http://dx.doi.org/10.1089/clo.2007.0064

Chan, K. K. et al., Stem Cells Dev 17, 825-836 (2008)
http://dx.doi.org/10.1089/scd.2008.0233

Chang, K. H. et al., Exp Cell Res 314, 2930-2940 (2008)
http://dx.doi.org/10.1016/j.yexcr.2008.07.019

Chavez, S. L. et al., Stem Cells Dev 17, 535-546 (2008)
http://dx.doi.org/10.1089/scd.2007.0216

2008 Chen, G. et al., Cell Stem Cell 2, 345-355 (2008)
http://dx.doi.org/10.1016/j.stem.2008.02.004

Chen, X. et al., Stem Cells 26, 2759-2767 (2008)
http://dx.doi.org/10.1634/stemcells.2008-0398

Chen, Y. T. et al., Stem Cells Dev 17, 853-855 (2008)
http://dx.doi.org/10.1089/scd.2007.0226

Cheng, E. H. et al., Reprod Biomed Online 17, 436-444 (2008)
http://www.rbmonline.com/4DCGI/Article/Detail?38%091%09=%203303%09

Chiao, E. et al., Stem Cells 26, 2032-2041 (2008)
http://dx.doi.org/10.1634/stemcells.2007-0964

Chiba, S. et al., Stem Cells 26, 2810-2820 (2008)
http://dx.doi.org/10.1634/stemcells.2008-0085

Cho, M. S. et al., Proc Natl Acad Sci U S A 105, 3392-3397 (2008)
http://dx.doi.org/10.1073/pnas.0712359105

Cho, Y. M. et al., Biochem Biophys Res Commun 366, 129-134 (2008)
http://dx.doi.org/10.1016/j.bbrc.2007.11.112

Choi, H. S. et al., Cell Tissue Res 333, 197-206 (2008)
http://dx.doi.org/10.1007/s00441-008-0632-6

Choo, A. B. et al., Stem Cells 26, 1454-1463 (2008)
http://dx.doi.org/10.1634/stemcells.2007-0576

Chung, Y. et al., Cell Stem Cell 2, 113-117 (2008)
http://dx.doi.org/10.1016/j.stem.2007.12.013

Cobo, F. et al., Cloning Stem Cells 10, 65-74 (2008)
http://dx.doi.org/10.1089/clo.2007.0020

Come, J. et al., Tissue Eng Part C Methods 14, 289-298 (2008)
http://dx.doi.org/10.1089/ten.tec.2008.0029

Conrad, S. et al., Nature 456, 344-349 (2008)
http://dx.doi.org/10.1038/nature07404

Correia, A. S. et al., Front Neurosci 2, 26 (2008)
http://dx.doi.org/10.3389/neuro.01.011.2008

Daadi, M. M. et al., PLoS ONE 3, e1644 (2008)
http://dx.doi.org/10.1371/journal.pone.0001644

Davis, R. P. et al., Blood 111, 1876-1884 (2008)
http://dx.doi.org/10.1182/blood-2007-06-093609

De Temmerman, N. et al., Mol Hum Reprod 14, 405-412 (2008)
http://dx.doi.org/10.1093/molehr/gan034

2008 Desbordes, S. C. et al., Cell Stem Cell 2, 602-612 (2008)
http://dx.doi.org/10.1016/j.stem.2008.05.010

Dhara, S. K. et al., Differentiation 76, 454-464 (2008)
http://dx.doi.org/10.1111/j.1432-0436.2007.00256.x

Di Domenico, A. I. et al., Cloning Stem Cells 10, 217-230 (2008)
http://dx.doi.org/10.1089/clo.2008.0016

Di Giorgio, F. P. et al., Cell Stem Cell 3, 637-648 (2008)
http://dx.doi.org/10.1016/j.stem.2008.09.017

Diecke, S. et al., Cells Tissues Organs 188, 52-61 (2008)
http://dx.doi.org/10.1159/000121282

Dimos, J. T. et al., Science 321, 1218-1221 (2008)
http://dx.doi.org/10.1126/science.1158799

Dormeyer, W. et al., J Proteome Res 7, 2936-2951 (2008)
http://dx.doi.org/10.1021/pr800056j

Dottori, M. et al., Stem Cells 26, 1146-1154 (2008)
http://dx.doi.org/10.1634/stemcells.2007-1118

Eiselleova, L. et al., Int J Dev Biol 52, 353-563 (2008)
http://dx.doi.org/10.1387/ijdb.082590le

Elkabetz, Y. et al., Genes Dev 22, 152-165 (2008)
http://dx.doi.org/10.1101/gad.1616208

Erceg, S. et al., PLoS ONE 3, e2122 (2008)
http://dx.doi.org/10.1371/journal.pone.0002122

Eshpeter, A. et al., Cell Prolif 41, 843-858 (2008)
http://dx.doi.org/10.1111/j.1365-2184.2008.00564.x

Feki, A. et al., Swiss Med Wkly 138, 540-550 (2008)
http://dx.doi.org/2008/37/smw-12385

Fletcher, J. et al., Cloning Stem Cells 10, 331-339 (2008)
http://dx.doi.org/10.1089/clo.2007.0094

Fong, H. et al., Stem Cells 26, 1931-1938 (2008)
http://dx.doi.org/10.1634/stemcells.2007-1002

Forsyth, N. R. et al., Regen Med 3, 817-833 (2008)
http://dx.doi.org/10.2217/17460751.3.6.817

Forsyth, N. R. et al., Rejuvenation Res 11, 5-17 (2008)
http://dx.doi.org/10.1089/rej.2007.0567

Fox, V. et al., Stem Cells 26, 715-723 (2008)
http://dx.doi.org/10.1634/stemcells.2007-0368

2008 Freed, W. J. et al., PLoS ONE 3, e1422 (2008)
http://dx.doi.org/10.1371/journal.pone.0001422

Freund, C. et al., Stem Cells 26, 724-733 (2008)
http://dx.doi.org/10.1634/stemcells.2007-0617

Fu, J. D. et al., Stem Cells Dev 17, 315-324 (2008)
http://dx.doi.org/10.1089/scd.2007.0114

Fuentealba, L. C. et al., Proc Natl Acad Sci U S A 105, 7732-7737 (2008)
http://dx.doi.org/10.1073/pnas.0803027105

Furue, M. K. et al., Proc Natl Acad Sci U S A 105, 1340913414 (2008)
http://dx.doi.org/10.1073/pnas.0806136105

Gallo, P. et al., Gene Ther 15, 161-170 (2008)
http://dx.doi.org/10.1038/sj.gt.3303017

Garcia-Gonzalo, F. R. et al., PLoS ONE 3, e1384 (2008)
http://dx.doi.org/10.1371/journal.pone.0001384

Ghule, P. N. et al., Proc Natl Acad Sci U S A 105, 16964-16964 (2008)
http://dx.doi.org/10.1073/pnas.0809273105

Gjerstorff, M. F. et al., Hum Reprod 23, 2194-2201 (2008)
http://dx.doi.org/10.1093/humrep/den262

Golob, J. L. et al., Dev Dyn 237, 1389-1398 (2008)
http://dx.doi.org/10.1002/dvdy.21545

Gong, J. et al., Exp Eye Res 86, 957-965 (2008)
http://dx.doi.org/10.1016/j.exer.2008.03.014

Graichen, R. et al., Differentiation 76, 357-370 (2008)
http://dx.doi.org/10.1111/j.1432-0436.2007.00236.x

Grandela, C. et al., Stem Cell Res. 1(2), 116–128 (2008)
http://dx.doi.org/10.1016/j.scr.2007.10.003

Greber, B. et al., Stem Cells Dev 17, 1065-1078 (2008)
http://dx.doi.org/10.1089/scd.2008.0035

Green, J. J. et al., Nano Lett 8, 3126-3130 (2008)
http://dx.doi.org/10.1021/nl8012665

Grinnemo, K. H. et al., Stem Cells 26, 1850-1857 (2008)
http://dx.doi.org/10.1634/stemcells.2008.0111

Hall, L. L. et al., J Cell Physiol 216, 445-452 (2008)
http://dx.doi.org/10.1002/jcp.21411

Harb, N. et al., PLoS ONE 3, e3001 (2008)
http://dx.doi.org/10.1371/journal.pone.0003001

2008 Harkness, L. et al., Stem Cell Res 1, 219-227 (2008)
http://dx.doi.org/10.1016/j.scr.2008.06.001

Hay, D. C. et al., Proc Natl Acad Sci U S A 105, 12301-12306 (2008)
http://dx.doi.org/10.1073/pnas.0806522105

Hay, D. C. et al., Stem Cells 26, 894-902 (2008)
http://dx.doi.org/10.1634/stemcells.2007-0718

Hayashi, H. et al., Eur J Neurosci 27, 261-268 (2008)
http://dx.doi.org/10.1111/j.1460-9568.2008.06027.x

Hayes, B. et al., Stem Cells 26, 465-473 (2008)
http://dx.doi.org/10.1634/stemcells.2007-0640

Heng, B. C. et al., Tissue Cell 40, 219-228 (2008)
http://dx.doi.org/10.1016/j.tice.2007.12.003

Hikita, S. T. et al., PLoS ONE 3, e3312 (2008)
http://dx.doi.org/10.1371/journal.pone.0003312

Hoben, G. M. et al., Stem Cells 26, 422-430 (2008)
http://dx.doi.org/10.1634/stemcells.2007-0641

Hockemeyer, D. et al., Cell Stem Cell 3, 346-353 (2008)
http://dx.doi.org/10.1016/j.stem.2008.08.014

Hohenstein, K. A. et al., Stem Cells 26, 1436-1443 (2008)
http://dx.doi.org/10.1634/stemcells.2007-0857

Hong, S. et al., J Neurochem 104, 316-324 (2008)
http://dx.doi.org/10.1111/j.1471-4159.2007.04952.x

Huangfu, D. et al., Nat Biotechnol 26, 1269-1275 (2008)
http://dx.doi.org/10.1038/nbt.1502

Hurst, J. H. et al., BMC Neurosci 9, 118 (2008)
http://dx.doi.org/10.1186/1471-2202-9-118

Hwang, N. S. et al., PLoS ONE 3, e2498 (2008)
http://dx.doi.org/10.1371/journal.pone.0002498

Hwang, N. S. et al., Proc Natl Acad Sci U S A 105, 20641-20646 (2008)
http://dx.doi.org/10.1073/pnas.0809680106

Inanc, B. et al., Artif Organs 32, 100-109 (2008)
http://dx.doi.org/10.1111/j.1525-1594.2007.00499.x

Inanc, B. et al., Tissue Eng Part A 14, 955-964 (2008)
http://dx.doi.org/10.1089/ten.tea.2007.0213

Inniss, K. et al., Stem Cells Dev 17, 1195 (2008)
http://dx.doi.org/10.1089/scd.2008.0063

2008 Ishii, T. et al., Am J Physiol Gastrointest Liver Physiol 295, G313 (2008)
http://dx.doi.org/10.1152/ajpgi.00072.2008

Ivey, K. N. et al., Cell Stem Cell 2, 219-229 (2008)
http://dx.doi.org/10.1016/j.stem.2008.01.016

Jaksch, M. et al., Cancer Res 68, 7882-7886 (2008)
http://dx.doi.org/10.1158/0008-5472.CAN-08-0723

Jang, J. et al., Stem Cells 26, 2782-2790 (2008)
http://dx.doi.org/10.1634/stemcells.2007-1053

Ji, J. et al., Stem Cells 26, 2485-2495 (2008)
http://dx.doi.org/10.1634/stemcells.2008-0642

Karlsson, K. R. et al., Exp Hematol 36, 1167-1175 (2008)
http://dx.doi.org/10.1016/j.exphem.2008.04.009

Kim, S. et al., Biomaterials 29, 1043-1053 (2008)
http://dx.doi.org/10.1016/j.biomaterials.2007.11.005

Kim, Y. Y. et al., Yonsei Med J 49, 819-827 (2008)
http://dx.doi.org/10.3349/ymj.2008.49.5.819

King, C. C. et al., Regen Med 3, 175-180 (2008)
http://dx.doi.org/10.2217/17460751.3.2.175

Kiprilov, E. N. et al., J Cell Biol 180, 897-904 (2008)
http://dx.doi.org/10.1083/jcb.200706028

Koay, E. J. et al., Osteoarthritis Cartilage 16, 1450-1456 (2008)
http://dx.doi.org/10.1016/j.joca.2008.04.007

Korneev, S. A. et al., RNA 14, 2030-2037 (2008)
http://dx.doi.org/10.1261/rna.1084308

Kroon, E. et al., Nat Biotechnol 26, 443-452 (2008)
http://dx.doi.org/10.1038/nbt1393

Ku, M. et al., PLoS Genet 4, e1000242 (2008)
http://dx.doi.org/10.1371/journal.pgen.1000242

Kuntz, S. et al., Stem Cells 26, 734-744 (2008)
http://dx.doi.org/10.1634/stemcells.2007-0772

Ladewig, J. et al., Stem Cells 26, 1705-1712 (2008)
http://dx.doi.org/10.1634/stemcells.2008-0007

Lagarkova, M. A. et al., Cell Cycle 7, 2929-2935 (2008)
http://www.landesbioscience.com/journals/cc/article/6700/

Lagarkova, M. A. et al., Cell Cycle 7, 3610-3612 (2008)
http://www.landesbioscience.com/journals/cc/article/6981/

2008 Lai, B. et al., Stem Cells Dev 17, 565-572 (2008)
http://dx.doi.org/10.1089/scd.2007.0124

Lam, H. et al., Biochem Biophys Res Commun 372, 601-616 (2008)
http://dx.doi.org/10.1016/j.bbrc.2008.05.116

Lappalainen, R. S. et al., Neurosci Lett 440, 246-250 (2008)
http://dx.doi.org/10.1016/j.neulet.2008.05.090

Laurent, L. C. et al., Stem Cells 26, 1506-1516 (2008)
http://dx.doi.org/10.1634/stemcells.2007-1081

Lavon, N. et al., Stem Cells 26, 1874-1882 (2008)
http://dx.doi.org/10.1634/stemcells.2008-0156

Ledran, M. H. et al., Cell Stem Cell 3, 85-93 (2008)
http://dx.doi.org/10.1016/j.stem.2008.06.001

Lee, G. S. et al., Mol Cells 25, 487-493 (2008)
http://molcells.inforang.com/article_pdf/Ksmcb/25/Ksmcb25-4-5.pdf

Lee, Y. J. et al., Int J Dev Biol 52, 43-45 (2008)
http://dx.doi.org/10.1387/ijdb.072274yl

Lefort, N. et al., Nat Biotechnol 26, 1364-1366 (2008)
http://dx.doi.org/10.1038/nbt.1509

Lerou, P. H. et al., Nat Biotechnol 26, 212-214 (2008)
http://dx.doi.org/10.1038/nbt1378

Levenstein, M. E. et al., Stem Cells 26, 3099-3107 (2008)
http://dx.doi.org/10.1634/stemcells.2007-1056

Li, T. et al., Hum Reprod 23, 358-364 (2008)
http://dx.doi.org/10.1093/humrep/dem386

Li, X. et al., J Genet Genomics 35, 723-728 (2008)
http://dx.doi.org/10.1016/S1673-8527(08)60227-1

Li, X. et al., Stem Cells Dev 17, 1079-1085 (2008)
http://dx.doi.org/10.1089/scd.2007.0247

Li, X. J. et al., Stem Cells 26, 886-893 (2008)
http://dx.doi.org/10.1634/stemcells.2007-0620

Li, Z. et al., Stem Cells 26, 864-873 (2008)
http://dx.doi.org/10.1634/stemcells.2007-0843

Liao, J. et al., Cell Res 18, 600-603 (2008)
http://dx.doi.org/10.1038/cr.2008.51

Liew, C. G. et al., PLoS ONE 3, e1783 (2008)
http://dx.doi.org/10.1371/journal.pone.0001783

2008 Lock, L. T. et al., Med J Malaysia 63 Suppl A, 5-6 (2008)
http://www.ncbi.nlm.nih.gov/pubmed/19024957

Lowry, W. E. et al., Proc Natl Acad Sci U S A 105, 2883-2888 (2008)
http://dx.doi.org/10.1073/pnas.0711983105

Lu, S. J. et al., Blood 112, 4475-4484 (2008)
http://dx.doi.org/10.1182/blood-2008-05-157198

Lu, S. J. et al., Regen Med 3, 693-704 (2008)
http://dx.doi.org/10.2217/17460751.3.5.693

Ma, F. et al., Proc Natl Acad Sci U S A 105, 13087-13092 (2008)
http://dx.doi.org/10.1073/pnas.0802220105

Ma, W. et al., BMC Dev Biol 8, 90 (2008)
http://dx.doi.org/10.1186/1471-213X-8-90

Maherali, N. et al., Cell Stem Cell 3, 340-345 (2008)
http://dx.doi.org/10.1016/j.stem.2008.08.003

Maimets, T. et al., Oncogene 27, 5277-5287 (2008)
http://dx.doi.org/10.1038/onc.2008.166

Mali, P. et al., Stem Cells 26, 1998-2005 (2008)
http://dx.doi.org/10.1634/stemcells.2008-0346

Mantel, C. et al., Cell Cycle 7, 484-492 (2008)
http://www.landesbioscience.com/journals/cc/article/5316/

Marchetto, M. C. et al., Cell Stem Cell 3, 649-657 (2008)
http://dx.doi.org/10.1016/j.stem.2008.10.001

Martin, C. H. et al., Blood 112, 2730-2737 (2008)
http://dx.doi.org/10.1182/blood-2008-01-133801

Martin-Ibanez, R. et al., Hum Reprod 23, 2744-2755 (2008)
http://dx.doi.org/10.1093/humrep/den316

Mateizel, I. et al., Reprod Biomed Online 16, 741-753 (2008)
http://www.rbmonline.com/4DCGI/Article/Detail?38%091%09=%203063%09

Maurer, J. et al., PLoS ONE 3, e3451 (2008)
http://dx.doi.org/10.1371/journal.pone.0003451

Maynard, S. et al., Stem Cells 26, 2266-2674 (2008)
http://dx.doi.org/10.1634/stemcells.2007-1041

Mayshar, Y. et al., Stem Cells 26, 767-747 (2008)
http://dx.doi.org/10.1634/stemcells.2007-1037

McElroy, S. L. et al., Reprod Biomed Online 16, 684-693 (2008)
http://www.rbmonline.com/4DCGI/Article/Detail?38%091%09=%203148%09

2008 Mehta, A. et al., Cell Biol Int 32, 1412-1424 (2008)
http://dx.doi.org/10.1016/j.cellbi.2008.08.012

Melchior, K. et al., Biol Chem 389, 897-903 (2008)
http://dx.doi.org/10.1515/BC.2008.108

Meng, G. et al., Stem Cells Dev 17, 413-422 (2008)
http://dx.doi.org/10.1089/scd.2007.0236

Metallo, C. M. et al., Biotechnol Bioeng 100, 830-837 (2008)
http://dx.doi.org/10.1002/bit.21809

Metallo, C. M. et al., Stem Cells 26, 372-380 (2008)
http://dx.doi.org/10.1634/stemcells.2007-0501

Miyazaki, T. et al., Biochem Biophys Res Commun 375, 27-32 (2008)
http://dx.doi.org/10.1016/j.bbrc.2008.07.111

Molne, J. et al., Transplantation 86, 1407-1413 (2008)
http://dx.doi.org/10.1097/TP.0b013e31818a6805

Monk, M. et al., Mol Hum Reprod 14, 347-355 (2008)
http://dx.doi.org/10.1093/molehr/gan025

Moore, J. C. et al., Biochem Biophys Res Commun 372, 553-558 (2008)
http://dx.doi.org/10.1016/j.bbrc.2008.05.076

Moore, J. C. et al., Biochem Biophys Res Commun 377, 4651 (2008)
http://dx.doi.org/10.1016/j.bbrc.2008.09.076

Morin, R. D. et al., Genome Res 18, 610-621 (2008)
http://dx.doi.org/10.1101/gr.7179508

Mouffouk, F. et al., Anal Biochem 372, 140-147 (2008)
http://dx.doi.org/10.1016/j.ab.2007.09.024

Mujoo, K. et al., Proc Natl Acad Sci U S A 105, 18924-18929 (2008)
http://dx.doi.org/10.1073/pnas.0810230105

Muller, F. J. et al., Nature 455, 401-405 (2008)
http://dx.doi.org/10.1038/nature07213

Nakagawa, M. et al., Nat Biotechnol 26, 101-106 (2008)
http://dx.doi.org/10.1038/nbt1374

Navarro-Alvarez, N. et al., Cell Transplant 17, 27-33 (2008)
http://www.ingentaconnect.com/content/cog/ct/2008/00000017/F0020001/art00005

O'Connor, M. D. et al., Stem Cells 26, 1109-1116 (2008)
http://dx.doi.org/10.1634/stemcells.2007-0801

Okoye, U. C. et al., J Mol Signal 3, 16 (2008)
http://dx.doi.org/10.1186/1750-2187-3-16

2008 Osafune, K. et al., Nat Biotechnol 26, 313-315 (2008)
http://dx.doi.org/10.1038/nbt1383

Osakada, F. et al., Nat Biotechnol 26, 215-224 (2008)
http://dx.doi.org/10.1038/nbt1384

Oyamada, N. et al., J Transl Med 6, 54 (2008)
http://dx.doi.org/10.1186/1479-5876-6-54

Paatero, A. O. et al., Nucleic Acids Res 36, e148 (2008)
http://dx.doi.org/10.1093/nar/gkn801

Park, I. H. et al., Cell 134, 877-886 (2008)
http://dx.doi.org/10.1016/j.cell.2008.07.041

Park, I. H. et al., Nature 451, 141-146 (2008)
http://dx.doi.org/10.1038/nature06534

Park, Y. B. et al., Exp Mol Med 40, 98-108 (2008)
http://www.e-emm.org/search_read.htm?page=98&year=2008&vol=40

Peiffer, I. et al., Stem Cells Dev 17, 519-533 (2008)
http://dx.doi.org/10.1089/scd.2007.0279

Pereira, C. F. et al., PLoS Genet 4, e1000170 (2008)
http://dx.doi.org/10.1371/journal.pgen.1000170

Peura, T. et al., Cloning Stem Cells 10, 203-216 (2008)
http://dx.doi.org/10.1089/clo.2007.0062

Phanstiel, D. et al., Proc Natl Acad Sci U S A 105, 4093-4098 (2008)
http://dx.doi.org/10.1073/pnas.0710515105

Phillips, B. W. et al., J Biotechnol 134, 79-87 (2008)
http://dx.doi.org/10.1016/j.jbiotec.2007.12.007

Phillips, B. W. et al., J Biotechnol 138, 24-32 (2008)
http://dx.doi.org/10.1016/j.jbiotec.2008.07.1997

Pomp, O. et al., Brain Res 1230, 50-60 (2008)
http://dx.doi.org/10.1016/j.brainres.2008.07.029

Postovit, L. M. et al., Proc Natl Acad Sci U S A 105, 4329-4334 (2008)
http://dx.doi.org/10.1073/pnas.0800467105

Qiu, C. et al., Blood 111, 2400-2408 (2008)
http://dx.doi.org/10.1182/blood-2007-07-102087

Qiu, D. et al., Biochem Biophys Res Commun 369, 735-740 (2008)
http://dx.doi.org/10.1016/j.bbrc.2008.02.102

Ravindran, G. et al., Biochem Biophys Res Commun 373, 258-264 (2008)
http://dx.doi.org/10.1016/j.bbrc.2008.06.022

2008 Raya, A. et al., Cold Spring Harb Symp Quant Biol 73, 127-135 (2008)
http://dx.doi.org/10.1101/sqb.2008.73.038

Richards, S. et al., Tissue Eng Part C Methods 14, 221-232 (2008)
http://dx.doi.org/10.1089/ten.tec.2007.0428

Robey, T. E. et al., J Mol Cell Cardiol 45, 567-581 (2008)
http://dx.doi.org/10.1016/j.yjmcc.2008.03.009

Sachlos, E. et al., Biomaterials 29, 4471-4480 (2008)
http://dx.doi.org/10.1016/j.biomaterials.2008.08.012

Saha, S. et al., Biophys J 94, 4123-4133 (2008)
http://dx.doi.org/10.1529/biophysj.107.119891

Salvagiotto, G. et al., Exp Hematol 36, 1377-1389 (2008)
http://dx.doi.org/10.1016/j.exphem.2008.06.015

Santoni de Sio, F. R. et al., Stem Cells 26, 2142-2152 (2008)
http://dx.doi.org/10.1634/stemcells.2007-0705

Saretzki, G. et al., Stem Cells 26, 455-464 (2008)
http://dx.doi.org/10.1634/stemcells.2007-0628

Satin, J. et al., Stem Cells 26, 1961-1972 (2008)
http://dx.doi.org/10.1634/stemcells.2007-0591

Saxena, S. et al., Mol Reprod Dev 75, 1523-1532 (2008)
http://dx.doi.org/10.1002/mrd.20895

Schaumburg, C. et al., J Virol 82, 8896-8899 (2008)
http://dx.doi.org/10.1128/JVI.00406-08

Sedan, O. et al., Stem Cells 26, 3130-3138 (2008)
http://dx.doi.org/10.1634/stemcells.2008-0777

Seguin, C. A. et al., Cell Stem Cell 3, 182-195 (2008)
http://dx.doi.org/10.1016/j.stem.2008.06.018

Seol, H. W. et al., Chromosome Res 16, 1075-1084 (2008)
http://dx.doi.org/10.1007/s10577-008-1258-y

Shen, Y. et al., Proc Natl Acad Sci U S A 105, 4709-4714 (2008)
http://dx.doi.org/10.1073/pnas.0712018105

Shiraki, N. et al., Genes Cells 13, 731-746 (2008)
http://dx.doi.org/10.1111/j.1365-2443.2008.01201.x

Shojaei, F. et al., Exp Hematol 36, 1442-1454 (2008)
http://dx.doi.org/10.1016/j.exphem.2008.06.001

Sidhu, K. S. et al., Stem Cells Dev 17, 41-51 (2008)
http://dx.doi.org/10.1089/scd.2007.0055

2008 Silva, S. S. et al., Proc Natl Acad Sci U S A 105, 4820-4825 (2008)
http://dx.doi.org/10.1073/pnas.0712136105

Siti-Ismail, N. et al., Biomaterials 29, 3946-3952 (2008)
http://dx.doi.org/10.1016/j.biomaterials.2008.04.027

Sivasubramaniyan, K. et al., Regen Med 3, 23-31 (2008)
http://dx.doi.org/10.2217/17460751.3.1.23

Smith, J. R. et al., Dev Biol 313, 107-117 (2008)
http://dx.doi.org/10.1016/j.ydbio.2007.10.003

Smith, J. R. et al., Stem Cells 26, 496-504 (2008)
http://dx.doi.org/10.1634/stemcells.2007-0039

Son, M. Y. et al., Reproduction 136, 423-432 (2008)
http://dx.doi.org/10.1530/REP-08-0080

Song, T. et al., Mol Hum Reprod 14, 619-625 (2008)
http://dx.doi.org/10.1093/molehr/gan058

Spits, C. et al., Nat Biotechnol 26, 1361-1363 (2008)
http://dx.doi.org/10.1038/nbt.1510

Stewart, R. et al., Regen Med 3, 505-522 (2008)
http://dx.doi.org/10.2217/17460751.3.4.505

Su, Z. et al., Clin Cancer Res 14, 6207-6217 (2008)
http://dx.doi.org/10.1158/1078-0432.CCR-08-0309

Sumi, T. et al., Development 135, 2969-2679 (2008)
http://dx.doi.org/10.1242/dev.021121

Sun, X. et al., Hum Reprod 23, 2185-2193 (2008)
http://dx.doi.org/10.1093/humrep/den137

Suzuki, K. et al., Proc Natl Acad Sci U S A 105, 13781-13786 (2008)
http://dx.doi.org/10.1073/pnas.0806976105

Swijnenburg, R. J. et al., Proc Natl Acad Sci U S A 105, 12991-12996 (2008)
http://dx.doi.org/10.1073/pnas.0805802105

Synnergren, J. et al., J Biotechnol 134, 162-170 (2008)
http://dx.doi.org/10.1016/j.jbiotec.2007.11.011

Synnergren, J. et al., Stem Cells 26, 1831-1840 (2008)
http://dx.doi.org/10.1634/stemcells.2007-1033

Takayama, N. et al., Blood 111, 5298-5306 (2008)
http://dx.doi.org/10.1182/blood-2007-10-117622

Tan, J. C. et al., Neuroreport 19, 1451-1455 (2008)
http://dx.doi.org/10.1097/WNR.0b013e32830e4c35

2008 Tateishi, K. et al., J Biol Chem 283, 31601-31697 (2008)
http://dx.doi.org/10.1074/jbc.M806597200

Thomson, A. et al., Cloning Stem Cells 10, 89-106 (2008)
http://dx.doi.org/10.1089/clo.2007.0072

Thyagarajan, B. et al., Stem Cells 26, 119-126 (2008)
http://dx.doi.org/10.1634/stemcells.2007-0283

Tian, X. F. et al., Scand J Clin Lab Invest 68, 58-67 (2008)
http://dx.doi.org/10.1080/00365510701466416

Tilgner, K. et al., Stem Cells 26, 3075-3085 (2008)
http://dx.doi.org/10.1634/stemcells.2008-0289

Tremoleda, J. L. et al., Cloning Stem Cells 10, 119-132 (2008)
http://dx.doi.org/10.1089/clo.2007.0R36

Trivedi, P. et al., Exp Hematol 36, 350-359 (2008)
http://dx.doi.org/10.1016/j.exphem.2007.10.007

Tsuneyoshi, N. et al., Biochem Biophys Res Commun 367, 899-905 (2008)
http://dx.doi.org/10.1016/j.bbrc.2007.12.189

Turetsky, T. et al., Hum Reprod 23, 46-53 (2008)
http://dx.doi.org/10.1093/humrep/dem351

Tzezana, R. et al., Tissue Eng Part C Methods 14, 281-288 (2008)
http://dx.doi.org/10.1089/ten.tec.2008.0201

Tzur, G. et al., PLoS ONE 3, e3726 (2008)
http://dx.doi.org/10.1371/journal.pone.0003726

Ullmann, U. et al., Mol Hum Reprod 14, 169-179 (2008)
http://dx.doi.org/10.1093/molehr/gan001

Unger, C. et al., Stem Cells 26, 2455-2466 (2008)
http://dx.doi.org/10.1634/stemcells.2007-0876

Ungrin, M. D. et al., PLoS ONE 3, e1565 (2008)
http://dx.doi.org/10.1371/journal.pone.0001565

Valamehr, B. et al., Proc Natl Acad Sci U S A 105, 14459-14464 (2008)
http://dx.doi.org/10.1073/pnas.0807235105

Valbuena, D. et al., Reprod Biomed Online 17, 127-135 (2008)
http://www.rbmonline.com/4DCGI/Article/Detail?38%091%09=%203217%09

Van Hoof, D. et al., Stem Cells 26, 2777-2781 (2008)
http://dx.doi.org/10.1634/stemcells.2008-0365

van Laake, L. W. et al., Circ Res 102, 1008-1010 (2008)
http://dx.doi.org/10.1161/CIRCRESAHA.108.175505

2008 Varelas, X. et al., Nat Cell Biol 10, 837-848 (2008)
http://dx.doi.org/10.1038/ncb1748

Vazin, T. et al., Restor Neurol Neurosci 26, 447-458 (2008)
http://iospress.metapress.com/content/e56529g2uvj54188/

Vazin, T. et al., Stem Cells 26, 1517-1525 (2008)
http://dx.doi.org/10.1634/stemcells.2008-0039

Vinoth, K. J. et al., Stem Cells Dev 17, 599-607 (2008)
http://dx.doi.org/10.1089/scd.2007.0088

Vugler, A. et al., Exp Neurol 214, 347-361 (2008)
http://dx.doi.org/10.1016/j.expneurol.2008.09.007

Wang, Z. X. et al., Stem Cells 26, 2791-2799 (2008)
http://dx.doi.org/10.1634/stemcells.2008-0443

Wearne, K. A. et al., Glycoconj J 25, 121-136 (2008)
http://dx.doi.org/10.1007/s10719-007-9064-x

West, F. D. et al., Stem Cells 26, 2768-2776 (2008)
http://dx.doi.org/10.1634/stemcells.2008-0124

West, M. D. et al., Regen Med 3, 287-308 (2008)
http://dx.doi.org/10.2217/17460751.3.3.287

Westfall, S. D. et al., Stem Cells Dev 17, 869-881 (2008)
http://dx.doi.org/10.1089/scd.2007.0240

Woll, P. S. et al., Blood 111, 122-131 (2008)
http://dx.doi.org/10.1182/blood-2007-04-084186

Wu, H. et al., Stem Cells 26, 1484-1489 (2008)
http://dx.doi.org/10.1634/stemcells.2007-0993

Wu, Z. et al., J Biol Chem 283, 24991-25002 (2008)
http://dx.doi.org/10.1074/jbc.M803893200

Xia, X. et al., Stem Cells 26, 525-533 (2008)
http://dx.doi.org/10.1634/stemcells.2007-0710

Xie, D. et al., Genome Res 18, 1325-1335 (2008)
http://dx.doi.org/10.1101/gr.072769.107

Xu, R. H. et al., Cell Stem Cell 3, 196-206 (2008)
http://dx.doi.org/10.1016/j.stem.2008.07.001

Xu, X. Q. et al., Cytotherapy 10, 376-389 (2008)
http://dx.doi.org/10.1080/14653240802105307

Xu, X. Q. et al., Differentiation 76, 958-970 (2008)
http://dx.doi.org/10.1111/j.1432-0436.2008.00284.x

2008 Yamahara, K. et al., PLoS ONE 3, e1666 (2008)
http://dx.doi.org/10.1371/journal.pone.0001666

Yang, C. et al., Stem Cells 26, 850-863 (2008)
http://dx.doi.org/10.1634/stemcells.2007-0677

Yang, D. et al., Stem Cells 26, 55-63 (2008)
http://dx.doi.org/10.1634/stemcells.2007-0494

Yang, L. et al., Nature 453, 524-528 (2008)
http://dx.doi.org/10.1038/nature06894

Yang, S. et al., Genes Chromosomes Cancer 47, 665-679 (2008)
http://dx.doi.org/10.1002/gcc.20574

Yirme, G. et al., Stem Cells Dev 17, 1227-1241 (2008)
http://dx.doi.org/10.1089/scd.2007.0272

Yocum, A. K. et al., Mol Cell Proteomics 7, 750-767 (2008)
http://dx.doi.org/10.1074/mcp.M700399-MCP200

Yu, X. et al., Cell Stem Cell 2, 461-471 (2008)
http://dx.doi.org/10.1016/j.stem.2008.03.001

Zambidis, E. T. et al., Blood 112, 3601-3614 (2008)
http://dx.doi.org/10.1182/blood-2008-03-144766

Zdravkovic, T. et al., Reprod Toxicol 26, 86-93 (2008)
http://dx.doi.org/10.1016/j.reprotox.2008.07.004

Zhan, X. et al., Cloning Stem Cells 10, 513-522 (2008)
http://dx.doi.org/10.1089/clo.2007.0087

Zhang, P. et al., Blood 111, 1933-1941 (2008)
http://dx.doi.org/10.1182/blood-2007-02-074120

Zhao, M. et al., Mol Biotechnol 40, 19-26 (2008)
http://dx.doi.org/10.1007/s12033-008-9043-x

Zhao, Y. et al., Cell Stem Cell 3, 475-479 (2008)
http://dx.doi.org/10.1016/j.stem.2008.10.002

Zhong, J. F. et al., Lab Chip 8, 68-74 (2008)
http://dx.doi.org/10.1039/b712116d

Zhou, J. et al., Stem Cells Dev 17, 737-749 (2008)
http://dx.doi.org/10.1089/scd.2007.0186

Zhou, J. M. et al., Cell Biol Int 32, 80-85 (2008)
http://dx.doi.org/10.1016/j.cellbi.2007.08.015

Zhou, Y. et al., Biochem Biophys Res Commun 376, 542-547 (2008)
http://dx.doi.org/10.1016/j.bbrc.2008.09.032
