# Supplementary material for: Human Embryonic Stem Cell Lines and Their Use in International Research
Source: Stem Cells. 2010 Feb;28(2):240–6. doi: 10.1002/stem.286 (PMC2952289; doi:10.1002/stem.286)
Supplement: Supplementary file 6 [file stem0028-0240-SD6.pdf]

Use of hESC Lines in Published Experimental Research

as determined by analysis of English language peer-reviewed literature

---

alpha-EC-C

Provider: Sun Yat-sen University, Guangzhou, China

Li, T. et al., Hum Reprod 23, 358-364 (2008)

AMC-hES1

Provider: Asan Medical Center -University of Ulsan, Seoul, Korea

Lee, Y. J. et al., Int J Dev Biol 52, 43-45 (2008)

AMC-hES2

Provider: Asan Medical Center -University of Ulsan, Seoul, Korea

Lee, Y. J. et al., Int J Dev Biol 52, 43-45 (2008)

AS034

Provider: Cellartis AB, Göteborg, Sweden

Heins, N. et al., Stem Cells 22, 367-376 (2004)

Brolen, G. K. C. et al., Diabetes 54, 2867-2874 (2005)

Darnfors, C. et al., Stem Cells 23, 483-488 (2005)

Noaksson, K. et al., Stem Cells 23, 1460-1467 (2005)

Soderdahl, T. et al., Toxicol In Vitro 21, 929-937 (2007)

Molne, J. et al., Transplantation 86, 1407-1413 (2008)

AS034.1 (subline of AS034)

Provider: Cellartis AB, Göteborg, Sweden

Heins, N. et al., Stem Cells 22, 367-376 (2004)

Darnfors, C. et al., Stem Cells 23, 483-488 (2005)

Noaksson, K. et al., Stem Cells 23, 1460-1467 (2005)

Heins, N. et al., J Biotechnol 122, 511-520 (2006)

Bigdeli, N. et al., J Biotechnol 133, 146-153 (2008)

AS034.1.1 (subline of AS034)

Provider: Cellartis AB, Göteborg, Sweden

Caisander, G. et al., Chromosome Res 14, 131-137 (2006)

Heins, N. et al., J Biotechnol 122, 511-520 (2006)

AS038

Provider: Cellartis AB, Göteborg, Sweden

Heins, N. et al., Stem Cells 22, 367-376 (2004)

Darnfors, C. et al., Stem Cells 23, 483-488 (2005)

Sjögren-Jansson, E. et al., Dev Dyn 233, 1304-1314 (2005)

Molne, J. et al., Transplantation 86, 1407-1413 (2008)

AS094 / SA094

Provider: Cellartis AB, Göteborg, Sweden

Hansson, M. et al., Diabetes 53, 2603-2609 (2004)

BG01 (hESBGN-01)

Provider: BresaGen, Inc., Athens, Georgia

Mitalipova, M. et al., Stem Cells 21, 521-526 (2003)

Schulz, T. C. et al., BMC Neurosci 4, 27 (2003)

Bhattacharya, B. et al., Blood 103, 2956-2964 (2004)

Bieberich, E. et al., J Cell Biol 167, 723-734 (2004)

Brimble, S. N. et al., Stem Cells Dev 13, 585-597 (2004)

Buytaert-Hoefen, K. A. et al., Stem Cells 22, 669-674 (2004)

Calhoun, J. D. et al., Biochem Biophys Res Commun 323, 453-464 (2004)

Rao, R. R. et al., Biotechnol Bioeng 88, 273-286 (2004)

Sato, N. et al., Nat Med 10, 55-63 (2004)

Schulz, T. C. et al., Stem Cells 22, 1218-1238 (2004)

Zeng, X. et al., Restor Neurol Neurosci 22, 421-428 (2004)

Zeng, X. et al., Stem Cells 22, 292-312 (2004)

Zeng, X. et al., Stem Cells 22, 925-940 (2004)

Goh, G. et al., Thromb Haemost 94, 728-737 (2005)

James, D. et al., Development 132, 1273-1282 (2005)

Maitra, A. et al., Nat Genet 37, 1099-1103 (2005)

Mitalipova, M. M. et al., Nat Biotechnol 23, 19-20 (2005)

Noaksson, K. et al., Stem Cells 23, 1460-1467 (2005)

Schwartz, C. M. et al., Stem Cells Dev 14, 517-534 (2005)

Shin, S. et al., Stem Cells Dev 14, 266-269 (2005)

Steele, W. et al., Reprod Biomed Online 10, 755-766 (2005)

Venable, A. et al., BMC Dev Biol 5, 15 (2005)

Yang, A. X. et al., Stem Cells Dev 14, 270-284 (2005)

Bibikova, M. et al., Genome Res 16, 1075-1083 (2006)

Cai, J. et al., Stem Cells 24, 516-530 (2006)

Chang, K. H. et al., Blood 108, 1515-1523 (2006)

D'Amour, K. A. et al., Nat Biotechnol 24, 1392-1401 (2006)

Denning, C. et al., Int J Dev Biol 50, 27-37 (2006)

Huettner, J. E. et al., Stem Cells 24, 1654-1667 (2006)

Josephson, R. et al., BMC Biol 4, 28 (2006)

Levine, A. J. & Brivanlou, A. H., Development 133, 209-216 (2006)

Li, H. et al., BMC Genomics 7, 103 (2006)

Liu, Y. et al., BMC Dev Biol 6, 20 (2006)

Lu, J., et al., Proc Natl Acad Sci U S A 103, 5688-5693 (2006)

Luo, Y. et al., Stem Cells 24, 865-875 (2006)

Mandal, A. et al., Differentiation 74, 81-90 (2006)

Nasonkin, I. O. & Koliatsos, V. E., Exp Neurol 201, 525-529 (2006)

Noggle, S. A. et al., Stem Cells 24, 1646-1653 (2006)
Plaia, T. W. et al., Stem Cells 24, 531-546 (2006)
Player, A. et al., Stem Cells Dev 15, 315-323 (2006)
Shin, S. et al., Stem Cells 24, 125-138 (2006)
Ware, C. B. et al, Stem Cells 24, 2677-2684 (2006)
Wearne, K. A. et al., Glycobiology 16, 981-990 (2006)
Zeng, X. et al., Neuropsychopharmacology 31, 2708-2715 (2006)
Adewumi, O. et al., Nat Biotechnol 25, 803-816 (2007)
Allegrucci, C. et al., Hum Mol Genet, 16, 1253-1268 (2007)
Brimble, S. N. et al., Stem Cells 25, 54-62 (2007)
Burridge, P. W. et al., Stem Cells, 25, 929-938 (2007)
Garcia-Perez, J. L. et al., Hum Mol Genet,16, 1569-1577 (2007)
Hirst, M. et al., Genome Biol 8, R113 (2007)
Iacovitti, L. et al., Brain Res 1127,19-25 (2007)
Josephson, R. et al., Stem Cells 25, 437-446 (2007)
Kim, K. et al., Cell Stem Cell 1, 346-352 (2007)
Kim, K. P. et al., Genome Res 17, 1731-1742 (2007)
McLean, A. B. et al., Stem Cells 25, 29-38 (2007)
Schulz, T. C. et al., BMC Genomics 8, 478 (2007)
Shin, S. et al., Stem Cells 25, 1298-1306 (2007)
Sun, Y. et al., Genomics 89, 22-35 (2007)
Terraciano, V. et al., Stem Cells 25, 2730-2738 (2007)
Wang, L. et al., Blood 110, 4111-4119 (2007)
Wilson, P. G. et al., Stem Cells Dev 16, 1027-1041 (2007)
Bar, M. et al., Stem Cells 26, 2496-2505 (2008)
Bonig, H. et al., Transfusion 48, 1039-1040 (2008)
Card, D. A. et al., Mol Cell Biol 28, 6426-6438 (2008)
Chan, E. M. et al., Cloning Stem Cells 10, 107-118 (2008)
Chang, K. H. et al., Exp Cell Res 314, 2930-2940 (2008)
Harb, N. et al., PLoS ONE 3, e3001 (2008)
Hockemeyer, D. et al., Cell Stem Cell 3, 346-353 (2008)
Maynard, S. et al., Stem Cells 26, 2266-2674 (2008)
Muller, F. J. et al., Nature 455, 401-405 (2008)
Vazin, T. et al., Restor Neurol Neurosci 26, 447-458 (2008)
Vazin, T. et al., Stem Cells 26, 1517-1525 (2008)
Wearne, K. A. et al., Glycoconj J 25, 121-136 (2008)
West, F. D. et al., Stem Cells 26, 2768-2776 (2008)
Yocum, A. K. et al., Mol Cell Proteomics 7, 750-767 (2008)

BG01V (subline of BG01)

Provider: BresaGen, Inc., Athens, Georgia

Zeng, X. et al., Restor Neurol Neurosci 22, 421-428 (2004)
Bibikova, M. et al., Genome Res 16, 1075-1083 (2006)

Herszfeld, D. et al., Nat Biotechnol 24, 351-357 (2006)
Josephson, R. et al., BMC Biol 4, 28 (2006)
Li, H. et al., BMC Genomics 7, 103 (2006)
Liu, Y. et al., BMC Dev Biol 6, 20 (2006)
Luo, Y. et al., Stem Cells 24, 865-875 (2006)
Plaia, T. W. et al., Stem Cells 24, 531-546 (2006)
Allegrucci, C. et al., Hum Mol Genet 16, 1253-68 (2007)
Brimble, S. N. et al., Stem Cells 25, 54-62 (2007)
Gauthaman, K. et al., Reprod Biomed Online 15, 566-581 (2007)
Greco, S. J. et al., Stem Cells 25, 3143-3154 (2007)
Josephson, R. et al., Stem Cells 25, 437-446 (2007)
Koay, E. J. et al., Stem Cells 25, 2183-2190 (2007)
Pal, R. & Khanna A., Differentiation 75, 112-122 (2007)
Pal, R. et al., Regen Med 2, 179-192 (2007)
Sun, Y. et al., Genomics 89, 22-35 (2007)
Barroso-delJesus, A. et al., Mol Cell Biol 28, 6609-6619 (2008)
Chiba, S. et al., Stem Cells 26, 2810-2820 (2008)
Gong, J. et al., Exp Eye Res 86, 957-965 (2008)
Hoben, G. M. et al., Stem Cells 26, 422-430 (2008)
Muller, F. J. et al., Nature 455, 401-405 (2008)
Thyagarajan, B. et al., Stem Cells 26, 119-126 (2008)

BG02 (hESBGN-02)

Provider: BresaGen, Inc., Athens, Georgia

Mitalipova, M. et al., Stem Cells 21, 521-526 (2003)
Schulz, T. C. et al., BMC Neurosci 4, 27 (2003)
Besser, D., J Biol Chem 279, 45076-45084 (2004)
Bhattacharya, B. et al., Blood 103, 2956-2964 (2004)
Brimble, S. N. et al., Stem Cells Dev 13, 585-597 (2004)
Dhara, S. K. & Benvenisty, N., Nucleic Acids Res 32, 3995-4002 (2004)
Sato, N. et al., Nat Med 10, 55-63 (2004)
Zeng, X. et al., Stem Cells 22, 292-312 (2004)
Bhattacharya, B. et al., BMC Dev Biol, 5, 22 (2005)
Hoffman, L. M. et al., Stem Cells 23, 1468-1478 (2005)
James, D. et al., Development 132, 1273-1282 (2005)
Maitra, A. et al., Nat Genet 37, 1099-1103 (2005)
Mitalipova, M. M. et al., Nat Biotechnol 23, 19-20 (2005)
Noaksson, K. et al., Stem Cells 23, 1460-1467 (2005)
Schwartz, C. M. et al., Stem Cells Dev 14, 517-534 (2005)
Shin, S. et al., Stem Cells Dev 14, 266-269 (2005)
Venable, A. et al., BMC Dev Biol 5, 15 (2005)
Yang, A. X. et al., Stem Cells Dev 14, 270-284 (2005)
Bibikova, M. et al., Genome Res 16, 1075-1083 (2006)

Cai, J. et al., Stem Cells 24, 516-530 (2006)

Chang, K. H. et al., Blood 108, 1515-1523 (2006)

D'Amour, K. A. et al., Nat Biotechnol 24, 1392-1401 (2006)

Hwang, N. S. et al., Tissue Eng 12, 2695-2706 (2006)

Josephson, R. et al., BMC Biol 4, 28 (2006)

Li, H. et al., BMC Genomics 7, 103 (2006)

Liu, Y. et al., BMC Dev Biol 6, 20 (2006)

Noggle, S. A. et al., Stem Cells 24, 1646-1653 (2006)

Player, A. et al., Stem Cells Dev 15, 315-323 (2006)

Shin, S. et al., Stem Cells 24, 125-138 (2006)

Ware, C. B. et al, Stem Cells 24, 2677-2684 (2006)

Adewumi, O. et al., Nat Biotechnol 25, 803-816 (2007)

Boyd, N. L. et al., Exp Biol Med (Maywood) 232, 833-843 (2007)

Brimble, S. N. et al., Stem Cells 25, 54-62 (2007)

McLean, A. B. et al., Stem Cells 25, 29-38 (2007)

Shin, S. et al., Stem Cells Dev 16, 131-141 (2007)

Sun, Y. et al., Genomics 89, 22-35 (2007)

Terraciano, V. et al., Stem Cells 25, 2730-2738 (2007)

Wang, L. et al., Blood 110, 4111-4119 (2007)

Wilson, P. G. et al., Stem Cells Dev 16, 1027-1041 (2007)

Bonig, H. et al., Transfusion 48, 1039-1040 (2008)

Braam, S. R. et al., Nat Methods 5, 389-392 (2008)

Chang, K. H. et al., Exp Cell Res 314, 2930-2940 (2008)

Green, J. J. et al., Nano Lett 8, 3126-3130 (2008)

Hockemeyer, D. et al., Cell Stem Cell 3, 346-353 (2008)

Hwang, N. S. et al., PLoS ONE 3, e2498 (2008)

Muller, F. J. et al., Nature 455, 401-405 (2008)

BG03 (hESBGN-03)

Provider: BresaGen, Inc., Athens, Georgia

Mitalipova, M. et al., Stem Cells 21, 521-526 (2003)

Brimble, S. N. et al., Stem Cells Dev 13, 585-597 (2004)

Schulz, T. C. et al., Stem Cells 22, 1218-1238 (2004)

Maitra, A. et al., Nat Genet 37, 1099-1103 (2005)

Noaksson, K. et al., Stem Cells 23, 1460-1467 (2005)

Schwartz, C. M. et al., Stem Cells Dev 14, 517-534 (2005)

Bibikova, M. et al., Genome Res 16, 1075-1083 (2006)

Cai, J. et al., Stem Cells 24, 516-530 (2006)

Chang, K. H. et al., Blood 108, 1515-1523 (2006)

D'Amour, K. A. et al., Nat Biotechnol 24, 1392-1401 (2006)

Josephson, R. et al., BMC Biol 4, 28 (2006)

Li, H. et al., BMC Genomics 7, 103 (2006)

Liu, Y. et al., BMC Dev Biol 6, 20 (2006)

Luo, Y. et al., Stem Cells 24, 865-875 (2006)

Ware, C. B. et al, Stem Cells 24, 2677-2684 (2006)

Adewumi, O. et al., Nat Biotechnol 25, 803-816 (2007)

Brimble, S. N. et al., Stem Cells 25, 54-62 (2007)

Gharwan, H. et al., Mol Ther 15, 1827-1833 (2007)

Josephson, R. et al., Stem Cells 25, 437-446 (2007)

Kim, K. et al., Cell Stem Cell 1, 346-352 (2007)

Pal, R. et al., Regen Med 2, 179-192 (2007)

Schulz, T. C. et al., BMC Genomics 8, 478 (2007)

Sun, Y. et al., Genomics 89, 22-35 (2007)

Tesar, P. J. et al., Nature 448, 196-199 (2007)

Wang, L. et al., Blood 110, 4111-4119 (2007)

Bonig, H. et al., Transfusion 48, 1039-1040 (2008)

Chang, K. H. et al., Exp Cell Res 314, 2930-2940 (2008)

Freed, W. J. et al., PLoS ONE 3, e1422 (2008)

Hall, L. L. et al., J Cell Physiol 216, 445-452 (2008)

Muller, F. J. et al., Nature 455, 401-405 (2008)

BG04 (hESBGN-04)

Provider: BresaGen, Inc., Athens, Georgia

Mitalipova, M. et al., Stem Cells 21, 521-526 (2003)

CA1

Provider: Mount Sinai Hospital, Toronto

Adewumi, O. et al., Nat Biotechnol 25, 803-816 (2007)

Bendall, S. C. et al., Nature 448, 1015-1021 (2007)

Peerani, R. et al., Embo J 26, 4744-4755 (2007)

Banuelos, C. A. et al., DNA Repair (Amst) 7, 1471-1483 (2008)

Li, X. et al., Stem Cells Dev 17, 1079-1085 (2008)

Meng, G. et al., Stem Cells Dev 17, 413-422 (2008)

O'Connor, M. D. et al., Stem Cells 26, 1109-1116 (2008)

Seguin, C. A. et al., Cell Stem Cell 3, 182-195 (2008)

Ungrin, M. D. et al., PLoS ONE 3, e1565 (2008)

Varelas, X. et al., Nat Cell Biol 10, 837-848 (2008)

CA2

Provider: Mount Sinai Hospital, Toronto

Adewumi, O. et al., Nat Biotechnol 25, 803-816 (2007)

Li, X. et al., Stem Cells Dev 17, 1079-1085 (2008)

Seguin, C. A. et al., Cell Stem Cell 3, 182-195 (2008)

Ungrin, M. D. et al., PLoS ONE 3, e1565 (2008)

CCTL10

Provider: Mendel University Brno

Dvorak, P. et al., Stem Cells 23, 1200-1211 (2005)

CCTL12

Provider: Mendel University Brno

Dvorak, P. et al., Stem Cells 23, 1200-1211 (2005)

Adewumi, O. et al., Nat Biotechnol 25, 803-816 (2007)

CCTL14

Provider: Mendel University Brno

Dvorak, P. et al., Stem Cells 23, 1200-1211 (2005)

Adewumi, O. et al., Nat Biotechnol 25, 803-816 (2007)

Eiselleova, L. et al., Int J Dev Biol 52, 353-563 (2008)

CCTL9

Provider: Mendel University Brno

Dvorak, P. et al., Stem Cells 23, 1200-1211 (2005)

Adewumi, O. et al., Nat Biotechnol 25, 803-816 (2007)

CHAhES-3

Provider: Pochon CHA University College of Medicine, Seoul, Korea

Ahn, S. E. et al., Biochem Biophys Res Commun, 340, 403-408 (2006)

Lee, J. et al., J Biol Chem 281, 33554-33565 (2006)

Cho, S. W. et al., Circulation 116, 2409-2419 (2007)

Kim, J. et al., Stem Cells Dev 16, 269-280 (2007)

Kim, S. et al., Stem Cells 25, 2601-2609 (2007)

Kim, S. et al., Stem Cells Dev 16, 537-545 (2007)

Kim, S. et al., Biomaterials 29, 1043-1053 (2008)

Son, M. Y. et al., Reproduction 136, 423-432 (2008)

CHAhES-4

Provider: Pochon CHA University College of Medicine, Seoul, Korea

Kim, M. S. et al., Lab Chip 7, 513-515 (2007)

Yeo, S. et al., Biochem Biophys Res Commun 359, 536-542 (2007)

CHB-1

Provider: Harvard University, Cambridge, MA, USA

Lerou, P. H. et al., Nat Biotechnol 26, 212-214 (2008)

CHB-10

Provider: Harvard University, Cambridge, MA, USA

Lerou, P. H. et al., Nat Biotechnol 26, 212-214 (2008)

CHB-11

Provider: Harvard University, Cambridge, MA, USA

Lerou, P. H. et al., Nat Biotechnol 26, 212-214 (2008)

CHB-12

Provider: Harvard University, Cambridge, MA, USA

Lerou, P. H. et al., Nat Biotechnol 26, 212-214 (2008)

CHB-2

Provider: Harvard University, Cambridge, MA, USA

Lerou, P. H. et al., Nat Biotechnol 26, 212-214 (2008)

CHB-3

Provider: Harvard University, Cambridge, MA, USA

Lerou, P. H. et al., Nat Biotechnol 26, 212-214 (2008)

CHB-4

Provider: Harvard University, Cambridge, MA, USA

Lerou, P. H. et al., Nat Biotechnol 26, 212-214 (2008)

CHB-5

Provider: Harvard University, Cambridge, MA, USA

Lerou, P. H. et al., Nat Biotechnol 26, 212-214 (2008)

CHB-6

Provider: Harvard University, Cambridge, MA, USA

Lerou, P. H. et al., Nat Biotechnol 26, 212-214 (2008)

CHB-8

Provider: Harvard University, Cambridge, MA, USA

Lerou, P. H. et al., Nat Biotechnol 26, 212-214 (2008)

CHB-9

Provider: Harvard University, Cambridge, MA, USA

Lerou, P. H. et al., Nat Biotechnol 26, 212-214 (2008)

CH-ES1

Provider: Geneva University, Geneva, Switzerland

Feki, A. et al., Swiss Med Wkly 138, 540-550 (2008)

---

cHES-1

Provider: Sun Yat-sen University, Guangzhou, China

Li, T. et al., Chin Med J (Engl) 118, 116-122 (2005)

---

chHES20

Provider: Central South University, Changsha, China

Yang, S. et al., Genes Chromosomes Cancer 47, 665-679 (2008)

Zhou, J. et al., Stem Cells Dev 17, 737-749 (2008)

---

chHES22

Provider: Central South University, Changsha, China

Zhou, J. et al., Stem Cells Dev 17, 737-749 (2008)

---

chHES3 (formerly chESC-3, H3)

Provider: Central South University, Changsha, China

Wang, J. et al., Cell Biol Int 29, 654-661 (2005)

Yang, S. et al., Genes Chromosomes Cancer 47, 665-679 (2008)

---

chHES8

Provider: Central South University, Changsha, China

Zhou, J. et al., Stem Cells Dev 17, 737-749 (2008)

CLS1

Provider: Laboratory for Stem Cell Research, Aalborg University, Denmark

Lysdahl, H. et al., Reprod Biomed Online 12, 119-126 (2006)

CLS2

Provider: Laboratory for Stem Cell Research, Aalborg University, Denmark

Lysdahl, H. et al., Reprod Biomed Online 12, 119-126 (2006)

CLS3

Provider: Laboratory for Stem Cell Research, Aalborg University, Denmark

Lysdahl, H. et al., Reprod Biomed Online 12, 119-126 (2006)

CLS4

Provider: Laboratory for Stem Cell Research, Aalborg University, Denmark

Lysdahl, H. et al., Reprod Biomed Online 12, 119-126 (2006)

CSES1

Provider: International Stem Cell Research Institute, Los Angeles, CA. USA, CA. USA

Lavon, N. et al., Stem Cells 26, 1874-1882 (2008)

CSES2

Provider: International Stem Cell Research Institute, Los Angeles, CA. USA

Lavon, N. et al., Stem Cells 26, 1874-1882 (2008)

CSES3

Provider: International Stem Cell Research Institute, Los Angeles, CA. USA

Lavon, N. et al., Stem Cells 26, 1874-1882 (2008)

CSES4

Provider: International Stem Cell Research Institute, Los Angeles, CA. USA

Lavon, N. et al., Stem Cells 26, 1874-1882 (2008)

CSES5

Provider: International Stem Cell Research Institute, Los Angeles, CA. USA

Lavon, N. et al., Stem Cells 26, 1874-1882 (2008)

CSES6

Provider: International Stem Cell Research Institute, Los Angeles, CA. USA

Lavon, N. et al., Stem Cells 26, 1874-1882 (2008)

CSES7

Provider: International Stem Cell Research Institute, Los Angeles, CA. USA

Lavon, N. et al., Stem Cells 26, 1874-1882 (2008)

CyT203

Provider: NovoCell Inc., San Diego, CA, USA

D'Amour, K. A. et al., Nat Biotechnol 24, 1392-1401 (2006)

Cezar, G. G. et al., Stem Cells Dev 16, 869-882 (2007)

Lakshmipathy, U. et al., Stem Cells Dev 16, 1003-1016 (2007)

Yeo, G. W. et al., PLoS Comput Biol 3, 1951-1967 (2007)

Kroon, E. et al., Nat Biotechnol 26, 443-452 (2008)

Marchetto, M. C. et al., Cell Stem Cell 3, 649-657 (2008)

CyT25

Provider: Cythera Inc., San Diego, CA, USA

Hoffman, L. M. et al., Stem Cells 23, 1468-1478 (2005)

Muotri, A. R. et al., Proc Natl Acad Sci U S A 102, 18644-18648 (2005)

D'Amour, K. A. et al., Nat Biotechnol 24, 1392-1401 (2006)

Garcia-Perez, J. L. et al., Hum Mol Genet, 16, 1569-1577 (2007)

Lakshmipathy, U. et al., Stem Cells Dev 16, 1003-1016 (2007)

McLean, A. B. et al., Stem Cells 25, 29-38 (2007)

Muller, F. J. et al., Nature 455, 401-405 (2008)

CyT49

Provider: NovoCell Inc., San Diego, CA, USA

D'Amour, K. A. et al., Nat Biotechnol 24, 1392-1401 (2006)

Wang, L. et al., Blood 110, 4111-4119 (2007)

Kroon, E. et al., Nat Biotechnol 26, 443-452 (2008)

Endeavour-1 (E1)

Provider: Prince of Wales Hospital, Sidney, & Illawarra Area Health Service, NSW, Australia

Sidhu, K. S. et al., Stem Cells Dev 17, 41-51 (2008)

ES[2]

Provider: Center for Regenerative Medicine in Barcedlona, Spain

Raya, A. et al., Cold Spring Harb Symp Quant Biol 73, 127-135 (2008)

ES[3]

Provider: Center for Regenerative Medicine in Barcedlona, Spain

Raya, A. et al., Cold Spring Harb Symp Quant Biol 73, 127-135 (2008)

ES[4]

Provider: Center for Regenerative Medicine in Barcedlona, Spain

Aasen, T. et al., Nat Biotechnol 26, 1276-1284 (2008)

Raya, A. et al., Cold Spring Harb Symp Quant Biol 73, 127-135 (2008)

ES[5]

Provider: Center for Regenerative Medicine in Barcedlona, Spain

Raya, A. et al., Cold Spring Harb Symp Quant Biol 73, 127-135 (2008)

ES[6]

Provider: Center for Regenerative Medicine in Barcedlona, Spain

Raya, A. et al., Cold Spring Harb Symp Quant Biol 73, 127-135 (2008)

ES-76

Provider: Jones Institute for Reproductive Medicine, Eastern Virginia Medical School, VA, USA

Lanzendorf, S. E. et al., Fertil Steril 76, 132-137 (2001)

Huntriss, J. et al., Mol Reprod Dev 67, 323-336 (2004)

ES-78-1

Provider: Jones Institute for Reproductive Medicine, Eastern Virginia Medical School, VA, USA

Lanzendorf, S. E. et al., Fertil Steril 76, 132-137 (2001)

ES-78-2

Provider: Jones Institute for Reproductive Medicine, Eastern Virginia Medical School, VA, USA

Lanzendorf, S. E. et al., Fertil Steril 76, 132-137 (2001)

ESI-013

Provider: ES Cell International Pte Ltd, Singapore

Crook, V. V. et al., Cell Stem Cell 1, 490-494 (2007)

ESI-014

Provider: ES Cell International Pte Ltd, Singapore

Crook, V. V. et al., Cell Stem Cell 1, 490-494 (2007)

ESI-017

Provider: ES Cell International Pte Ltd, Singapore

Crook, V. V. et al., Cell Stem Cell 1, 490-494 (2007)

Phillips, B. W. et al., J Biotechnol 138, 24-32 (2008)

Phillips, B. W. et al., J Biotechnol 134, 79-87 (2008)

ESI-027

Provider: ES Cell International Pte Ltd, Singapore

Crook, V. V. et al., Cell Stem Cell 1, 490-494 (2007)

ESI-035

Provider: ES Cell International Pte Ltd, Singapore

Crook, V. V. et al., Cell Stem Cell 1, 490-494 (2007)

ESI-049

Provider: ES Cell International Pte Ltd, Singapore

Crook, V. V. et al., Cell Stem Cell 1, 490-494 (2007)

ESI-051

Provider: ES Cell International Pte Ltd, Singapore

Crook, V. V. et al., Cell Stem Cell 1, 490-494 (2007)

ESI-053

Provider: ES Cell International Pte Ltd, Singapore

Crook, V. V. et al., Cell Stem Cell 1, 490-494 (2007)

ESM01

Provider: Russian Academy of Sciences, Moscow, Russia

Lagarkova, M. A. et al., Cell Cycle 5, 416-420 (2006)

Prokhorovich, M. A. et al., Bull Exp Biol Med 144, 126-129 (2007)

Korneev, S. A. et al., Rna 14, 2030-2037 (2008)

Lagarkova, M. A. et al., Cell Cycle 7, 2929-2935 (2008)

ESM02

Provider: Russian Academy of Sciences, Moscow, Russia

Lagarkova, M. A. et al., Cell Cycle 5, 416-420 (2006)

Prokhorovich, M. A. et al., Bull Exp Biol Med 144, 126-129 (2007)

Korneev, S. A. et al., RNA 14, 2030-2037 (2008)

Lagarkova, M. A. et al., Cell Cycle 7, 2929-2935 (2008)

ESM03

Provider: Russian Academy of Sciences, Moscow, Russia

Lagarkova, M. A. et al., Cell Cycle 5, 416-420 (2006)

Prokhorovich, M. A. et al., Bull Exp Biol Med 144, 126-129 (2007)

Lagarkova, M. A. et al., Cell Cycle 7, 2929-2935 (2008)

ESM04

Provider: Russian Academy of Sciences, Moscow, Russia

Prokhorovich, M. A. et al., Bull Exp Biol Med 144, 126-129 (2007)

FC018

Provider: Cellartis AB, Göteborg, Sweden

Heins, N. et al., Stem Cells 22, 367-376 (2004)

FES21

Provider: University of Helsinki, Finland

Skottman, H. et al., Stem Cells 23, 1343-1356 (2005)

Mikkola, M. et al., BMC Dev Biol 6, 40 (2006)

Adewumi, O. et al., Nat Biotechnol 25, 803-816 (2007)

Heiskanen, A. et al., Stem Cells 25, 197-202 (2007)

FES22

Provider: University of Helsinki, Finland

Skottman, H. et al., Stem Cells 23, 1343-1356 (2005)

Mikkola, M. et al., BMC Dev Biol 6, 40 (2006)

Adewumi, O. et al., Nat Biotechnol 25, 803-816 (2007)

Heiskanen, A. et al., Stem Cells 25, 197-202 (2007)

FES29

Provider: University of Helsinki, Finland

Skottman, H. et al., Stem Cells 23, 1343-1356 (2005)

Mikkola, M. et al., BMC Dev Biol 6, 40 (2006)

Adewumi, O. et al., Nat Biotechnol 25, 803-816 (2007)

Heiskanen, A. et al., Stem Cells 25, 197-202 (2007)

Paatero, A. O. et al., Nucleic Acids Res 36, e148 (2008)

FES30

Provider: University of Helsinki, Finland

Skottman, H. et al., Stem Cells 23, 1343-1356 (2005)

Mikkola, M. et al., BMC Dev Biol 6, 40 (2006)

Adewumi, O. et al., Nat Biotechnol 25, 803-816 (2007)

Heiskanen, A. et al., Stem Cells 25, 197-202 (2007)

FY-3PN

Provider: The Third Affiliated Hospital of Guangzhou Medical Collge, Guangzhou, China

Sun, X. et al., Hum Reprod 23, 2185-2193 (2008)

FY-hES-1

Provider: The Third Affiliated Hospital of Guangzhou Medical Collge, Guangzhou, China

Sun, X. et al., Hum Reprod 23, 2185-2193 (2008)

FY-hES-3

Provider: The Third Affiliated Hospital of Guangzhou Medical Collge, Guangzhou, China

Sun, X. et al., Hum Reprod 23, 2185-2193 (2008)

FY-hES-4

Provider: The Third Affiliated Hospital of Guangzhou Medical Collge, Guangzhou, China

Sun, X. et al., Hum Reprod 23, 2185-2193 (2008)

FY-hES-5

Provider: The Third Affiliated Hospital of Guangzhou Medical Collge, Guangzhou, China

Sun, X. et al., Hum Reprod 23, 2185-2193 (2008)

FY-hES-7

Provider: The Third Affiliated Hospital of Guangzhou Medical Collge, Guangzhou, China

Sun, X. et al., Hum Reprod 23, 2185-2193 (2008)

FY-hES-8

Provider: The Third Affiliated Hosptial of Guangzhou Medical Collge, Guangzhou, China

Sun, X. et al., Hum Reprod 23, 2185-2193 (2008)

H1 (WA01)

Provider: WiCell Research Institute, Madison, WI, USA

Thomson, J. A. et al., Science 282, 1145-1147 (1998)

Carpenter, M. K. et al., Exp Neurol 172, 383-397 (2001)

Kaufman, D. S. et al., Proc Natl Acad Sci U S A 98, 10716-10721 (2001)
Xu, C. H. et al., Nat Biotechnol 19, 971-974 (2001)
Zhang, S. C. et al., Nat Biotechnol 19, 1129-1133 (2001)
Pfeifer, A. et al., Proc Natl Acad Sci U S A 99, 2140-2145 (2002)
Xu, C. et al., Circ Res 91, 501-508 (2002)
Xu, R. H. et al., Nat Biotechnol 20, 1261-1264 (2002)
Carpenter, M. K. et al., Cloning Stem Cells 5, 79-88 (2003)
Chadwick, K. et al., Blood 102, 906-915 (2003)
Cheng, L. Z. et al., Stem Cells 21, 131-142 (2003)
He, J. Q. et al., Circ Res 93, 32-39 (2003)
Rambhatla, L. et al., Cell Transplant 12, 1-11 (2003)
Sato, N. et al., Dev Biol 260, 404-412 (2003)
Sottile, V. et al., Cloning Stem Cells 5, 149-155 (2003)
Besser, D., J Biol Chem 279, 45076-45084 (2004)
Bhattacharya, B. et al., Blood 103, 2956-2964 (2004)
Bielby, R. C. et al., Tissue Eng 10, 1518-1525 (2004)
Brandenberger, R. et al., BMC Dev Biol 4, 10 (2004)
Brandenberger, R. et al., Nat Biotechnol 22, 707-716 (2004)
Carpenter, M. K. et al., Dev Dyn 229, 243-258 (2004)
Cerdan, C.et al., Blood 103, 2504-2512 (2004)
Gerami-Naini, B. et al., Endocrinology 145, 1517-1524 (2004)
Ginis, I. et al., Dev Biol 269, 360-380 (2004)
Hay, D. C. et al., Stem Cells 22, 225-235 (2004)
Ji, L. et a ., Biotechnol Bioeng 88, 299-312 (2004)
Johkura, K. et al., J Anat 205, 247-255 (2004)
Klimanskaya, I. et al., Cloning Stem Cells 6, 217-245 (2004)
Lakshmipathy, U. et al., Stem Cells 22, 531-543 (2004)
Li, L. et al., Stem Cells 22, 448-456 (2004)
Liu, Y. P. et al., Stem Cells Dev 13, 636-645 (2004)
Lu, S. J. et al., Blood 103, 4134-4141 (2004)
Miura, T. et al., Stem Cells Dev 13, 694-715 (2004)
Orner, B. P. et al., J Am Chem Soc 126, 10808-10809 (2004)
Perrier, A. L. et al., Proc Natl Acad Sci U S A 101, 12543-12548 (2004)
Rao, R. R. et al., Biotechnol Bioeng 88, 273-286 (2004)
Reppel, M., et al., Cell Physiol Biochem 14, 187-196 (2004)
Rosler, E. S. et al., Dev Dyn 229, 259-274 (2004)
Sato, N. et al., Nat Med 10, 55-63 (2004)
Shirahashi, H. et al., Cell Transplant 13, 197-211 (2004)
Tian, X. et al., Exp Hematol 32, 1000-1009 (2004)
Wang, L. et al., Immunity 21, 31-41 (2004)
Xu, C. et al., Stem Cells 22, 972-980 (2004)
Zeng, X. et al., Stem Cells 22, 925-940 (2004)
Zhan, X. et al., Lancet 364, 163-171 (2004)

Barberi, T. et al., PLoS Med 2, e161 (2005)
Bhattacharya, B. et al., BMC Dev Biol, 5, 22 (2005)
Dravid, G. et al., Stem Cells 23, 1489-1501 (2005)
Enver, T. et al., Hum Mol Genet 14, 3129-1340 (2005)
Ezashi, T. et al., Proc Natl Acad Sci U S A 102, 4783-4788 (2005)
Fang, Z. F. et al., Cell Res 15, 394-400 (2005)
Genbacev, O. et al., Fertil Steril 83, 1517-1529 (2005)
Gerrard, L. et al., Stem Cells 23, 1234-1241 (2005)
Gerrard, L. et al., Stem Cells 23, 124-133 (2005)
Hoffman, L. M. et al., Stem Cells 23, 1468-1478 (2005)
Hyslop, L. et al., Stem Cells 23, 1035-1043 (2005)
James, D. et al., Development 132, 1273-1282 (2005)
Kameda, T. & Thomson, J. A., Stem Cells 23, 1535-1540 (2005)
Klimanskaya, I. et al., Lancet 365, 1636-1641 (2005)
Laflamme, M. A. et al., Am J Pathol 167, 663-671 (2005)
Li, X. J. et al., Nat Biotechnol 23, 215-221 (2005)
Li, Y. et al., Biotechnol Bioeng 91, 688-698 (2005)
Maitra, A. et al., Nat Genet 37, 1099-1103 (2005)
Martin, M. J. et al., Nat Med 11, 228-232 (2005)
Qiu, C. et al., Exp Hematol 33, 1450-1458 (2005)
Reppel, M. et al., J Electrocardiol 38, 166-170 (2005)
Schwartz, R. E. et al., Stem Cells Dev 14, 643-655 (2005)
Singh Roy, N. et al., Exp Neurol 196, 224-234 (2005)
Stojkovic, P. et al., Stem Cells 23, 306-314 (2005)
Stojkovic, P. et al., Stem Cells 23, 895-902 (2005)
Tabar, V. et al., Nat Biotechnol 23, 601-616 (2005)
Tai, G., et al., Biochem Biophys Res Commun 333, 1116-1122 (2005)
Vodyanik, M. A. et al., Blood 105, 617-626 (2005)
Wang, G. et al., Biochem Biophys Res Commun 330, 934-942 (2005)
Wang, K. et al., Stem Cells 23, 1526-1534 (2005)
Wang, L. et al., Blood 105, 4598-4603 (2005)
Wang, L. et al., J Exp Med 201, 1603-1614 (2005)
Wang, Q. et al., Stem Cells 23, 1221-1127 (2005)
Ware, C. B. et al., Biotechniques 38, 879-983 (2005)
Wei, C. L. et al., Stem Cells 23, 166-185 (2005)
Wiblin, A. E., et al., J Cell Sci 118, 3861-3868 (2005)
Xiong, C. et al., Stem Cells Dev 14, 671-675 (2005)
Xiong, C. et al., Stem Cells Dev,14, 367-377 (2005)
Xu, R. H. et al., Nat Methods 2, 185 (2005)
Xue, T. et al., Circulation 111, 11-20 (2005)
Yan, Y. et al., Stem Cells 23, 781-790 (2005)
Zambidis, E. T. et al., Blood 106, 860-870 (2005)
Zhan, M., et al., Cell Biochem Biophys 43, 379-405 (2005)

Anderson, J. S. et al., Retrovirology 3, 24 (2006)
Androutsellis-Theotokis, A. et al., Nature 442, 823-826 (2006)
Armstrong, L. et al., Hum Mol Genet 15, 1894-1913 (2006)
Becker, K. A. et al., J Cell Physiol 209, 883-893 (2006)
Bibikova, M. et al., Genome Res 16, 1075-1083 (2006)
Cameron, C. M. & Kaufman, D. S., Biotechnol Bioeng 94, 938-948 (2006)
Cerdan, C. et al., Nat Med 12, 1113-1114; author reply 1115 (2006)
Chang, K. H. et al., Blood 108, 1515-1523 (2006)
Fang, D. et al., Stem Cells 24, 1668-1677 (2006)
Forsyth, N. R. et al., Cloning Stem Cells 8, 16-23 (2006)
Galic, Z. et al., Proc Natl Acad Sci U S A 103, 11742-11747 (2006)
Heng, B. C. et al., Int J Med Sci 13, 124-129 (2006)
Heng, B. C. et al., J Biomed Sci 13, 433-445 (2006)
Heng, B. C. et al., Zygote 14, 361-348 (2006)
Heng, B.C. et al., In Vitro Cell Dev Biol Anim 42, 54-57 (2006)
Huettner, J. E. et al., Stem Cells 24, 1654-1667 (2006)
Jang, J. E. et al., Stem Cells Dev 15, 109-117 (2006)
Ji, L. et al., Tissue Eng 12, 665-679 (2006)
Josephson, R. et al., BMC Biol 4, 28 (2006)
Lamba, D. A. et al., Proc Natl Acad Sci U S A 103, 12769-12774 (2006)
Levenstein, M. E. et al., Stem Cells 24, 568-574 (2006)
Levine, A. J. & Brivanlou, A. H., Development 133, 209-216 (2006)
Liu, Y. et al., Biochem Biophys Res Commun 346, 131-139 (2006)
Liu, Y. et al., BMC Dev Biol 6, 20 (2006)
Ludwig, T. E. et al., Nat Biotechnol 24, 185-187 (2006)
Lund, R. D. et al., Cloning Stem Cells 8, 189-199 (2006)
Mohr, J. C. et al., Biomaterials 27, 6032-6042 (2006)
Mohr, J. C. et al., Biotechnol Prog 22, 825-834 (2006)
Narayan, A. D. et al., Blood 107, 2180-2183 (2006)
Noggle, S. A. et al., Stem Cells 24, 1646-1653 (2006)
Olivier, E. N. et al., Exp Hematol 34, 1635-1642 (2006)
Olivier, E. N. et al., Stem Cells 24, 1914-1922 (2006)
Plaia, T. W. et al., Stem Cells 24, 531-546 (2006)
Postovit, L. M. et al., Stem Cells 24, 501-505 (2006)
Pyle, A. D. et al., Nat Biotechnol 24, 344-350 (2006)
Ren, C. et al., Stem Cells 24, 1338-1347 (2006)
Roy, N. S. et al., Nat Med 12, 1259-1268 (2006)
Saha, S. et al., J Cell Physiol 206, 126-137 (2006)
Samadikuchaksaraei, A. et al., Tissue Eng 12, 867-875 (2006)
Slukvin, I. I. et al., J Immunol 176, 2924-2932 (2006)
Stewart, M.H. et al., Nat Methods 3, 807-815 (2006)
Suter, D. M. et al., Stem Cells 24, 615-623 (2006)
Tian, X. et al., Stem Cells 24, 1370-1380 (2006)

Vats, A. et al., Tissue Eng 12, 1687-1697 (2006)
Vodyanik, M. A. et al., Blood 108, 2095-2105 (2006)
Wang, T. W. et al., J Comp Neurol 497, 88-100 (2006)
Ware, C. B. et al, Stem Cells 24, 2677-2684 (2006)
Xiao, L. et al., Stem Cells 24, 1476-1486 (2006)
Xu, C. et al., Stem Cells Dev 15, 931-941 (2006)
Xu, X. et al., Cloning Stem Cells 8, 96-107 (2006)
Yao, S. et al., Proc Natl Acad Sci U S A 103, 6907-6912 (2006)
Yu, J. et al., Stem Cells 24, 168-176 (2006)
Zhang, X. et al., Stem Cells, 24, 2669-2676 (2006)
Zhang, Y. W. et al., Stem Cells Dev 15, 943-952 (2006)
Zheng, J. K. et al., Cell Res 16, 713-722 (2006)
Adewumi, O. et al., Nat Biotechnol 25, 803-816 (2007)
Ahmad, S. et al., Stem Cells 25, 1145-1155 (2007)
Babaie, Y. et al., Stem Cells 25, 500-510 (2007)
Baker, D. E. et al., Nat Biotechnol 25, 207-215 (2007)
Bakre, M. M. et al., J Biol Chem 282, 31703-31712 (2007)
Barberi, T. et al., Nat Med, 13, 642-648 (2007)
Becker, K. A. et al., J Cell Physiol, 210, 517-526 (2007)
Bendall, S. C. et al., Nature 448, 1015-1021 (2007)
Bettiol, E. et al., Differentiation 75, 669-681 (2007)
Brons, I. G. et al., Nature 448, 191-195 (2007)
Cai J. et al., Hepatology 45, 1229-1239 (2007)
Cai L. et al., Cell Res 17, 62-72 (2007)
Cezar, G. G. et al.,Stem Cells Dev 16, 869-882 (2007)
Chen D. et al., Exp Hematol 35, 1344-1357 (2007)
Chen, T. et al., Stem Cells 25, 392-401 (2007)
Choudhary, M. et al., Stem Cells 25, 3045-3057 (2007)
Cui, L. et al., J Struct Bio 158, 307-317 (2007)
Das, P. et al., Stem Cell Res 1, 61-74 (2007)
Derda, R. et al., ACS Chem Biol 2, 347-355 (2007)
Garcia-Perez, J. L. et al.,Hum Mol Genet,16, 1569-1577 (2007)
Gerecht S. et al., Proc Natl Acad Sci U S A 104, 11298-11303 (2007)
Gharwan, H. et al., Mol Ther 15, 1827-1833 (2007)
Greber, B. et al., BMC Dev Biol 7, 46 (2007)
Greber, B. et al., Stem Cells 25, 455-464 (2007)
Hay, D. C. et al., Cloning Stem Cells 9, 51-62 (2007)
Heng, B. C. et al., Biosci Rep 27, 257-264 (2007)
Hirst, M. et al., Genome Biol 8, R113 (2007)
Irion, S. et al., Nat Biotechnol 25, 1477-1482 (2007)
Jiang, J. et al., Stem Cells 25, 1940-1953 (2007)
Jiang, W. et al., Cell Res 17, 333-344 (2007)
Josephson, R. et al., Stem Cells 25, 437-446 (2007)

Kennedy, M. et al., Blood 109, 2679-2687 (2007)
Kim, K. P. et al., Genome Res 17, 1731-1742 (2007)
Krtolica, A. et al., Stem Cells 25, 2215-2223 (2007)
Lee, H. et al., Stem Cells 25, 1931-1939 (2007)
Li, J. et al., Differentiation 75, 299-307 (2007)
Lian, Q. et al., Stem Cells 25, 425-436 (2007)
Liew, C. G. et al., Stem Cells 25, 1521-1528 (2007)
Liu, J. et al., Stem Cells 25, 3038-3044 (2007)
Lu, S. J. et al., Genome Biol 8, R240 (2007)
Lu, S. J. et al., Nat Methods 4, 501-509 (2007)
Lu, S. J. et al., Stem Cells Dev 16, 547-559 (2007)
Ma, F. et al., Int J Hematol 85, 371-379 (2007)
McLean, A. B. et al., Stem Cells 25, 29-38 (2007)
Ohm, J. E. et al, Nat Genet 39, 237-242 (2007)
Okamura, R. M. et al., J Neuroimmunol 192, 1-2 (2007)
Pan, G. et al., Cell Stem Cell 1, 299-312 (2007)
Pankratz, M. T. et al., Stem Cells 25, 1511-1520 (2007)
Pillekamp, F. et al., Stem Cells 25, 174-180 (2007)
Pruszak, J. et al., Stem Cells 25, 2257-2268 (2007)
Qin, H. et al., J Biol Chem 282, 5842-5852 (2007)
Rufaihah, A. J. et al., J Gene Med 9, 452-461 (2007)
Sartiani, L. et al., Stem Cells 25, 1136-1144 (2007)
Shih, C. C. et al., Stem Cells Dev 16, 893-902 (2007)
Shin, S. et al., Stem Cells 25, 1298-1306 (2007)
Soh, B. S. et al., Stem Cells 25, 3029-3037 (2007)
Srivastava, A. S. et al., Stem Cells 25, 1456-1461 (2007)
Sullivan, K. E. et al., Mol Cell Biol 27, 5147-5160 (2007)
Toh, W. S. et al., Stem Cells 25, 950-960 (2007)
Trivedi, P. et al., Exp Hematol 35, 146-154 (2007)
Wang, L. et al., Blood 110, 4111-4119 (2007)
Wang, Z. X. et al., Stem Cells 25, 2173-2182 (2007)
Wang, Z. Z. et al., Nat Biotechnol 25, 317-318 (2007)
Xia, X. et al., Stem Cells Dev 16, 167-176 (2007)
Xie, C. Q. et al., Arterioscler Thromb Vasc Biol 27, e311-312 (2007)
Xie, C. Q. et al., Stem Cells Dev 16, 25-29 (2007)
Yu, J. et al., Science 318, 1917-1920 (2007)
Zhao, M. et al., Biochem Biophys Res Commun 362, 916-922 (2007)
Zhong, J. F. et al., Gene Expr 14, 23-34 (2007)
Zhou, B. Y. et al., Stem Cells 25, 779-789 (2007)
Adler, S. et al., Toxicol In Vitro 22, 200-211 (2008)
Agarwal, S. et al., Stem Cells 26, 1117-1127 (2008)
Ananiev, G. E. et al., BMC Mol Biol 9, 68 (2008)
Bandi, S. et al., AIDS Res Ther 5, 1 (2008)

Banuelos, C. A. et al., DNA Repair (Amst) 7, 1471-1483 (2008)

Bar, M. et al., Stem Cells 26, 2496-2505 (2008)

Bendall, S. C. et al., Mol Cell Proteomics 7, 1587-1597 (2008)

Bera, T. K. et al., Stem Cells Dev 17, 325-332 (2008)

Bonig, H. et al., Transfusion 48, 1039-1040 (2008)

Cao, H. et al., PLoS ONE 3, e2820 (2008)

Card, D. A. et al., Mol Cell Biol 28, 6426-6438 (2008)

Chang, K. H. et al., Exp Cell Res 314, 2930-2940 (2008)

Chen, G. et al., Cell Stem Cell 2, 345-355 (2008)

Chen, X. et al., Stem Cells 26, 2759-2767 (2008)

Chen, Y. T. et al., Stem Cells Dev 17, 853-855 (2008)

Choi, H. S. et al., Cell Tissue Res 333, 197-206 (2008)

Conrad, S. et al., Nature 456, 344-349 (2008)

Desbordes, S. C. et al., Cell Stem Cell 2, 602-612 (2008)

Di Domenico, A. I. et al., Cloning Stem Cells 10, 217-230 (2008)

Elkabetz, Y. et al., Genes Dev 22, 152-165 (2008)

Erceg, S. et al., PLoS ONE 3, e2122 (2008)

Eshpeter, A. et al., Cell Prolif 41, 843-858 (2008)

Fletcher, J. et al., Cloning Stem Cells 10, 331-339 (2008)

Fong, H. et al., Stem Cells 26, 1931-1938 (2008)

Forsyth, N. R. et al., Regen Med 3, 817-833 (2008)

Forsyth, N. R. et al., Rejuvenation Res 11, 5-17 (2008)

Fox, V. et al., Stem Cells 26, 715-723 (2008)

Fu, J. D. et al., Stem Cells Dev 17, 315-324 (2008)

Greber, B. et al., Stem Cells Dev 17, 1065-1078 (2008)

Harb, N. et al., PLoS ONE 3, e3001 (2008)

Hay, D. C. et al., Proc Natl Acad Sci U S A 105, 12301-12306 (2008)

Hay, D. C. et al., Stem Cells 26, 894-902 (2008)

Hayes, B. et al., Stem Cells 26, 465-473 (2008)

Heng, B. C. et al., Tissue Cell 40, 219-228 (2008)

Hohenstein, K. A. et al., Stem Cells 26, 1436-1443 (2008)

Hong, S. et al., J Neurochem 104, 316-324 (2008)

Ji, J. et al., Stem Cells 26, 2485-2495 (2008)

Kiprilov, E. N. et al., J Cell Biol 180, 897-904 (2008)

Ledran, M. H. et al., Cell Stem Cell 3, 85-93 (2008)

Lee, G. S. et al., Mol Cells 25, 487-493 (2008)

Lefort, N. et al., Nat Biotechnol 26, 1364-1366 (2008)

Levenstein, M. E. et al., Stem Cells 26, 3099-3107 (2008)

Li, X. et al., Stem Cells Dev 17, 1079-1085 (2008)

Li, X. J. et al., Stem Cells 26, 886-893 (2008)

Lu, S. J. et al., Blood 112, 4475-4484 (2008)

Lu, S. J. et al., Regen Med 3, 693-704 (2008)

Ma, F. et al., Proc Natl Acad Sci U S A 105, 13087-13092 (2008)

Maimets, T. et al., Oncogene 27, 5277-5287 (2008)
Melchior, K. et al., Biol Chem 389, 897-903 (2008)
Metallo, C. M. et al., Stem Cells 26, 372-380 (2008)
Moore, J. C. et al., Biochem Biophys Res Commun 372, 553-558 (2008)
Moore, J. C. et al., Biochem Biophys Res Commun 377, 46-51 (2008)
Muller, F. J. et al., Nature 455, 401-405 (2008)
O'Connor, M. D. et al., Stem Cells 26, 1109-1116 (2008)
Pereira, C. F. et al., PLoS Genet 4, e1000170 (2008)
Phanstiel, D. et al., Proc Natl Acad Sci U S A 105, 4093-4098 (2008)
Postovit, L. M. et al., Proc Natl Acad Sci U S A 105, 4329-4334 (2008)
Qiu, C. et al., Blood 111, 2400-2408 (2008)
Richards, S. et al., Tissue Eng Part C Methods 14, 221-232 (2008)
Saha, S. et al., Biophys J 94, 4123-4133 (2008)
Salvagiotto, G. et al., Exp Hematol 36, 1377-1389 (2008)
Saretzki, G. et al., Stem Cells 26, 455-464 (2008)
Siti-Ismail, N. et al., Biomaterials 29, 3946-3952 (2008)
Smith, J. R. et al., Stem Cells 26, 496-504 (2008)
Stewart, R. et al., Regen Med 3, 505-522 (2008)
Swijnenburg, R. J. et al., Proc Natl Acad Sci U S A 105, 12991-12996 (2008)
Thomson, A. et al., Cloning Stem Cells 10, 89-106 (2008)
Tian, X. F. et al., Scand J Clin Lab Invest 68, 58-67 (2008)
Trivedi, P. et al., Exp Hematol 36, 350-359 (2008)
Vinoth, K. J. et al., Stem Cells Dev 17, 599-607 (2008)
Wang, Z. X. et al., Stem Cells 26, 2791-2799 (2008)
Westfall, S. D. et al., Stem Cells Dev 17, 869-881 (2008)
Woll, P. S. et al., Blood 111, 122-131 (2008)
Wu, Z. et al., J Biol Chem 283, 24991-25002 (2008)
Xie, D. et al., Genome Res 18, 1325-1335 (2008)
Xu, R. H. et al., Cell Stem Cell 3, 196-206 (2008)
Yang, C. et al., Stem Cells 26, 850-863 (2008)
Yang, L. et al., Nature 453, 524-528 (2008)
Yu, X. et al., Cell Stem Cell 2, 461-471 (2008)
Zambidis, E. T. et al., Blood 112, 3601-3614 (2008)
Zhan, X. et al., Cloning Stem Cells 10, 513-522 (2008)
Zhang, P. et al., Blood 111, 1933-1941 (2008)
Zhao, Y. et al., Cell Stem Cell 3, 475-479 (2008)

H1 (Derivatives)

Liu, Y. P. et al., Stem Cells Dev 14, 487-92 (2005)
Klimanskaya, I. et al., Nature 444, 481-85 (2006)
Chung, Y. et al., Cell Stem Cell 2, 113-117 (2008)

H1.1 (subline of H1)

Kaufman, D. S. et al., Proc Natl Acad Sci U S A 98, 10716-10721 (2001)

Sperger, J. M. et al., Proc Natl Acad Sci U S A 100, 13350-13355 (2003)

Zwaka, T. P.& Thomson, J. A., Nat Biotechnol 21, 319-321 (2003)

Draper, J. S. et al., Nat Biotechnol 22, 53-54 (2004)

Zwaka, T. P.& Thomson, J. A., Stem Cells 23, 146-149 (2005)

Heng, B. C. et al., Biotechnol Appl Biochem 47, 33-37 (2007)

H13 (WA13)

Provider: WiCell Research Institute, Madison, Wisconsin, USA

Thomson, J. A. et al., Science 282, 1145-1147 (1998)

Drukker, M. et al., Proc Natl Acad Sci U S A 99, 9864-9869 (2002)

Gerecht-Nir, S. et al., Lab Invest 83, 1811-1820 (2003)

Sperger, J. M. et al., Proc Natl Acad Sci U S A 100, 13350-13355 (2003)

Dvash, T. et al., Hum Reprod 19, 2875-2883 (2004)

Gerecht-Nir, S. et al., Biol Reprod 71, 2029-2036 (2004)

Gerecht-Nir, S. et al., Biotechnol Bioeng 88, 313-320 (2004)

Segev, H. et al., Stem Cells 22, 265-274 (2004)

Urbach, A.et al., Stem Cells 22, 635-641 (2004)

Zhan, M., et al., Cell Biochem Biophys 43, 379-405 (2005)

Darr, H. et al., Development 133, 1193-1201 (2006)

Drukker, M. et al., Stem Cells 24, 221-229 (2006)

Ware, C. B. et al, Stem Cells 24, 2677-2684 (2006)

Adewumi, O. et al., Nat Biotechnol 25, 803-816 (2007)

Dvash, T. et al., Stem Cells 25, 465-472 (2007)

Ferreira, L. S. et al., Circ Res 101, 286-294 (2007)

Figallo, E. et al., Lab Chip 7, 710-719 (2007)

Gerecht S. et al., Proc Natl Acad Sci U S A 104, 11298-11303 (2007)

Gerecht, S. et al., Biomaterials 28, 4068-4077 (2007)

Gerecht, S. et al., Biomaterials 28, 4826-4835 (2007)

Hirst, M. et al., Genome Biol 8, R113 (2007)

H14 (WA14)

Provider: WiCell Research Institute, Madison, Wisconsin, USA

Thomson, J. A. et al., Science 282, 1145-1147 (1998)

Xu, C. H. et al., Nat Biotechnol 19, 971-974 (2001)

Henderson, J. K. et al., Stem Cells 20, 329-337 (2002)

Xu, R. H. et al., Nat Biotechnol 20, 1261-1264 (2002)

He, J. Q. et al., Circ Res 93, 32-39 (2003)

Sperger, J. M. et al., Proc Natl Acad Sci U S A 100, 13350-13355 (2003)

Carpenter, M. K. et al., Dev Dyn 229, 243-258 (2004)

Draper, J. S. et al., Nat Biotechnol 22, 53-54 (2004)

Matin, M. M. et al., Stem Cells 22, 659-668 (2004)

Enver, T. et al., Hum Mol Genet 14, 3129-1340 (2005)

Xu, R. H. et al., Nat Methods 2, 185-190 (2005)

Harun, R. et al., Hum Reprod 21, 1349-1358 (2006)

Levenstein, M. E. et al., Stem Cells 24, 568-574 (2006)

Ludwig, T. E. et al., Nat Biotechnol 24, 185-187 (2006)

Ludwig, T. E. et al., Nat Methods 3, 637-646 (2006)

Ware, C. B. et al, Stem Cells 24, 2677-2684 (2006)

Adewumi, O. et al., Nat Biotechnol 25, 803-816 (2007)

Baker, D. E. et al., Nat Biotechnol 25, 207-215 (2007)

Gharwan, H. et al., Mol Ther 15, 1827-1833 (2007)

Hirst, M. et al., Genome Biol 8, R113 (2007)

Liew, C. G. et al., Stem Cells 25, 1521-1528 (2007)

Calvanese, V. et al., PLoS ONE 3, e3294 (2008)

Hikita, S. T. et al., PLoS ONE 3, e3312 (2008)

Levenstein, M. E. et al., Stem Cells 26, 3099-3107 (2008)

Maurer, J. et al., PLoS ONE 3, e3451 (2008)

Xu, R. H. et al., Cell Stem Cell 3, 196-206 (2008)

H1-OGN (subline of H1)

Park, I. H. et al., Nature 451, 141-146 (2008)

H2B

Provider: Technion-Israel Institute of Technology, Rambam Medical Center, Haifa

Bauwens, C. L. et al., Stem Cells 26, 2300-2310 (2008)

H7 (WA07)

Provider: WiCell Research Institute, Madison, Wisconsin, USA

Thomson, J. A. et al., Science 282, 1145-1147 (1998)

Carpenter, M. K. et al., Exp Neurol 172, 383-397 (2001)

Xu, C. H. et al., Nat Biotechnol 19, 971-974 (2001)

Andrews, P. W., Philos Trans R Soc Lond B Biol Sci 357, 405-417 (2002)

Draper, J. S. et al., J Anat 200, 249-258 (2002)

Henderson, J. K. et al., Stem Cells 20, 329-337 (2002)

Xu, C. et al., Circ Res 91, 501-508 (2002)

Xu, R. H. et al., Nat Biotechnol 20, 1261-1264 (2002)

Carpenter, M. K. et al., Cloning Stem Cells 5, 79-88 (2003)

He, J. Q. et al., Circ Res 93, 32-39 (2003)

Sperger, J. M. et al., Proc Natl Acad Sci U S A 100, 13350-13055 (2003)

Walsh, J. & Andrews, P. W., Apmis 111, 197-210 (2003)

Brandenberger, R. et al., BMC Dev Biol 4, 10 (2004)

Brandenberger, R. et al., Nat Biotechnol 22, 707-716 (2004)

Carpenter, M. K. et al., Dev Dyn 229, 243-258 (2004)
Draper, J. S. et al., Nat Biotechnol 22, 53-54 (2004)
Draper, J. S. et al., Stem Cells Dev 13, 325-336 (2004)
Ginis, I. et al., Dev Biol 269, 360-380 (2004)
Klimanskaya, I. et al., Cloning Stem Cells 6, 217-245 (2004)
Li, L. et al., Stem Cells 22, 448-456 (2004)
Matin, M. M. et al., Stem Cells 22, 659-668 (2004)
Miura, T. et al., Stem Cells Dev 13, 694-715 (2004)
Rosler, E. S. et al., Dev Dyn 229, 259-274 (2004)
Xu, C. et al., Stem Cells 22, 972-980 (2004)
Andrews, P. W. et al., Biochem Soc Trans 33, 1526-1530 (2005)
Bhattacharya, B. et al., BMC Dev Biol, 5, 22 (2005)
D'Amour, K. A. et al., Nat Biotechnol 23, 1534-1541(2005)
Enver, T. et al., Hum Mol Genet 14, 3129-1340 (2005)
Faulkner, J.& Keirstead, H. S., Transpl Immunol 15, 131-142 (2005)
Genbacev, O. et al., Fertil Steril 83, 1517-1529 (2005)
Gerrard, L. et al., Stem Cells 23, 1234-1241 (2005)
Hoffman, L. M. et al., Stem Cells 23, 1468-1478 (2005)
Keirstead, H. S. et al., J Neurosci 25, 4694-4705 (2005)
Klimanskaya, I. et al., Lancet 365, 1636-1641 (2005)
Laflamme, M. A. et al., Am J Pathol 167, 663-671 (2005)
Maitra, A. et al., Nat Genet 37, 1099-1103 (2005)
McDevitt T. C. et al., J Mol Cell Cardiol 39, 865-873 (2005)
Nistor, G. I. et al., Glia 49, 385-396 (2005)
Rugg-Gunn, P. J.et al., Nat Genet 37, 585-587 (2005)
Wang, L. et al., J Exp Med 201, 1603-1614 (2005)
Wei, C. L. et al., Stem Cells 23, 166-185 (2005)
Wiblin, A. E., et al., J Cell Sci 118, 3861-3868 (2005)
Xu, C. et al., Stem Cells 23, 315-323 (2005)
Bibikova, M. et al., Genome Res 16, 1075-1083 (2006)
Cloutier, F. et al., Reg Med 1, 469-479 (2006)
Harun, R. et al., Hum Reprod 21, 1349-1358 (2006)
Josephson, R. et al., BMC Biol 4, 28 (2006)
Kofidis, T. et al., Eur J Cardiothorac Surg 29, 50-55 (2006)
Lee, S. J. et al., Biomaterials 27, 3466-3472 (2006)
Levenstein, M. E. et al., Stem Cells 24, 568-574 (2006)
Liu, Y. et al., BMC Dev Biol 6, 20 (2006)
Ludwig, T. E. et al., Nat Biotechnol 24, 185-187 (2006)
Lund, R. D. et al., Cloning Stem Cells 8, 189-199 (2006)
Plaia, T. W. et al., Stem Cells 24, 531-546 (2006)
Ware, C. B. et al, Stem Cells 24, 2677-2684 (2006)
Xu, C. et al., Stem Cells Dev 15, 631-639 (2006)
Xu, C. et al., Stem Cells Dev 15, 931-941 (2006)

Zhang, Y. W. et al., Stem Cells Dev 15, 943-952 (2006)
Adewumi, O. et al., Nat Biotechnol 25, 803-816 (2007)
Allegrucci, C. et al., Hum Mol Genet, 16, 1253-1268 (2007)
Baker, D. E. et al., Nat Biotechnol 25, 207-215 (2007)
Carlson, M. E.& Conboy I. M., Aging Cell 6, 371-382 (2007)
Garcia-Perez, J. L. et al.,Hum Mol Genet,16, 1569-1577 (2007)
Hellman, A. et al., Science 315, 1141-1143 (2007)
Hirst, M. et al., Genome Biol 8, R113 (2007)
Jiang, J. et al., Stem Cells 25, 1940-1953 (2007)
Josephson, R. et al., Stem Cells 25, 437-446 (2007)
Krtolica, A. et al., Stem Cells 25, 2215-2223 (2007)
Laflamme, M. A. et al., Nat Biotechnol 25, 1015-1024 (2007)
Liew, C. G. et al., Stem Cells 25, 1521-1528 (2007)
Lu, S. J. et al., Nat Methods 4, 501-509 (2007)
Pajerowski J. D. et al., Proc Natl Acad Sci U S A 104, 15619-15624 (2007)
Pruszak, J. et al., Stem Cells 25, 2257-2268 (2007)
Shin, S. et al., Stem Cells 25, 1298-1306 (2007)
Sonntag, K. C. et al., Stem Cells 25, 411-418 (2007)
Trigona, W. L. et al., Antioxid Redox Signal 9, 751-756 (2007)
Atlasi, Y. et al., Stem Cells 26, 3068-3074 (2008)
Brito-Martins, M. et al., Br J Pharmacol 153, 751-759 (2008)
Calvanese, V. et al., PLoS ONE 3, e3294 (2008)
Fox, V. et al., Stem Cells 26, 715-723 (2008)
Furue, M. K. et al., Proc Natl Acad Sci U S A 105, 1340913414 (2008)
Golob, J. L. et al., Dev Dyn 237, 1389-1398 (2008)
Hall, L. L. et al., J Cell Physiol 216, 445-452 (2008)
Hay, D. C. et al., Stem Cells 26, 894-902 (2008)
Liew, C. G. et al., PLoS ONE 3, e1783 (2008)
Lu, S. J. et al., Blood 112, 4475 (2008)
Muller, F. J. et al., Nature 455, 401-405 (2008)
Okoye, U. C. et al., J Mol Signal 3, 16 (2008)
Peiffer, I. et al., Stem Cells Dev 17, 519-533 (2008)
Pereira, C. F. et al., PLoS Genet 4, e1000170 (2008)
Phanstiel, D. et al., Proc Natl Acad Sci U S A 105, 4093-4098 (2008)
Robey, T. E. et al., J Mol Cell Cardiol 45, 567-581 (2008)
Schaumburg, C. et al., J Virol 82, 8896-8899 (2008)
Shen, Y. et al., Proc Natl Acad Sci U S A 105, 4709-4714 (2008)
Silva, S. S. et al., Proc Natl Acad Sci U S A 105, 4820-4825 (2008)
Thomson, A. et al., Cloning Stem Cells 10, 89-106 (2008)
Tremoleda, J. L. et al., Cloning Stem Cells 10, 119-132 (2008)
Trivedi, P. et al., Exp Hematol 36, 350-359 (2008)
Zdravkovic, T. et al., Reprod Toxicol 26, 86-93 (2008)
Zhang, P. et al., Blood 111, 1933-1941 (2008)

Zhao, Y. et al., Cell Stem Cell 3, 475-479 (2008)

H9

Provider: WiCell Research Institute, Madison, Wisconsin, USA

Thomson, J. A. et al., Science 282, 1145-1147 (1998)

Amit, M. et al., Dev Biol 227, 271-278 (2000)

Itskovitz-Eldor, J. et al., Mol Med 6, 88-95 (2000)

Tzukerman, M. et al., Mol Biol Cell 11, 4381-4391 (2000)

Assady, S. et al., Diabetes 50, 1691-1697 (2001)

Carpenter, M. K. et al., Exp Neurol 172, 383-397 (2001)

Eiges, R. et al., Curr Biol 11, 514-518 (2001)

Schuldiner, M. et al., Brain Res 913, 201-205 (2001)

Xu, C. H. et al., Nat Biotechnol 19, 971-974 (2001)

Zhang, S. C. et al., Nat Biotechnol 19, 1129-1133 (2001)

Drukker, M. et al., Proc Natl Acad Sci U S A 99, 9864-9869 (2002)

Levenberg, S. et al., Proc Natl Acad Sci U S A 99, 4391-4396 (2002)

Xu, C. et al., Circ Res 91, 501-508 (2002)

Xu, R. H. et al., Nat Biotechnol 20, 1261-1264 (2002)

Amit, M. et al., Biol Reprod 68, 2150-2156 (2003)

Carpenter, M. K. et al., Cloning Stem Cells 5, 79-88 (2003)

Chadwick, K. et al., Blood 102, 906-915 (2003)

Green, H. et al., Proc Natl Acad Sci U S A 100, 15625-15630 (2003)

He, J. Q. et al., Circ Res 93, 32-39 (2003)

Levenberg, S. et al., Proc Natl Acad Sci U S A 100, 12741-12746 (2003)

Ma, Y. et al., Stem Cells 21, 111-117 (2003)

Rajagopal, J. et al., Science 299, 363 (2003)

Rambhatla, L. et al., Cell Transplant 12, 1-11 (2003)

Schuldiner, M. et al., Stem Cells 21, 257-265 (2003)

Smith-Arica, J. R. et al., Cloning Stem Cells 5, 51-62 (2003)

Sottile, V. et al., Cloning Stem Cells 5, 149-155 (2003)

Sperger, J. M. et al., Proc Natl Acad Sci U S A 100, 13350-13055 (2003)

Abeyta, M. J. et al., Hum Mol Genet 13, 601-608 (2004)

Amit, M. et al., Biol Reprod 70, 837-845 (2004)

Anderson, D. G. et al., Nat Biotechnol 22, 863-866 (2004)

Anneren, C.& Cowan, C. A., J Biol Chem 279, 31590-31598 (2004)

Bhattacharya, B. et al., Blood 103, 2956-2964 (2004)

Brandenberger, R. et al., BMC Dev Biol 4, 10 (2004)

Brandenberger, R. et al., Nat Biotechnol 22, 707-716 (2004)

Carpenter, M. K. et al., Dev Dyn 229, 243-258 (2004)

Cerdan, C., Rouleau, A., Bhatia, M., Blood 103, 2504-2512 (2004)

Clark, A. T. et al., Hum Mol Genet 13, 727-739 (2004)

Clark, A. T. et al., Stem Cells 22, 169-179 (2004)

Daheron, L. et al., Stem Cells 22, 770-778 (2004)

Dhara, S. K. & Benvenisty,K., N., Nucleic Acids Res 32, 3995-4002 (2004)
Dvash, T. et al., Hum Reprod 19, 2875-2883 (2004)
Ginis, I. et al., Dev Biol 269, 360-380 (2004)
Hay, D. C. et al., Stem Cells 22, 225-235 (2004)
Heng, B. C. et al., In Vitro Cell Dev Biol Anim 40, 255-257 (2004)
Ji, L. et a ., Biotechnol Bioeng 88, 299-312 (2004)
Klimanskaya, I. et al., Cloning Stem Cells 6, 217-245 (2004)
Li, L. et al., Stem Cells 22, 448-456 (2004)
Liu, Y. P. et al., Stem Cells Dev 13, 636-645 (2004)
Menendez, P. et al., Mol Ther 10, 1109-1120 (2004)
Miura, T. et al., Stem Cells Dev 13, 694-715 (2004)
Perrier, A. L. et al., Proc Natl Acad Sci U S A 101, 12543-12548 (2004)
Ponsaerts, P. et al., Cloning Stem Cells 6, 211-216 (2004)
Rosler, E. S. et al., Dev Dyn 229, 259-274 (2004)
Vallier, L. et al., Dev Biol 275, 403 (2004)
Vallier, L. et al., Stem Cells 22, 2-11 (2004)
Wang, L. et al., Immunity 21, 31-41 (2004)
Xu, C. et al., Stem Cells 22, 972-980 (2004)
Amit, M. et al., Stem Cells 23, 761-771 (2005)
Barberi, T. et al., PLoS Med 2, e161 (2005)
Bhattacharya, B. et al., BMC Dev Biol, 5, 22 (2005)
Boyer, L. A. et al., Cell 122, 947-956 (2005)
Cao, T. et al., Tissue Cell 37, 325-334 (2005)
Cowan, C. A. et al., Science 309, 369-373 (2005)
D'Amour, K. A. et al., Nat Biotechnol 23, 1534-1541(2005)
Dravid, G. et al., Stem Cells 23, 1489-1501 (2005)
Genbacev, O. et al., Fertil Steril 83, 1517-1529 (2005)
Gerrard, L. et al., Stem Cells 23, 1234-1241 (2005)
Heng, B. C. et al., Ann Clin Lab Sci 35, 459-462 (2005)
Heng, B. C. et al., Cell Biochem Funct 23, 141-146 (2005)
Hoffman, L. M. et al., Stem Cells 23, 1468-1478 (2005)
Kameda, T. & Thomson, J. A., Stem Cells 23, 1535-1540 (2005)
Klimanskaya, I. et al., Lancet 365, 1636-1641 (2005)
Levenberg, S. et al., Tissue Eng 11, 506-512 (2005)
Li, X. J. et al., Nat Biotechnol 23, 215-221 (2005)
Maitra, A. et al., Nat Genet 37, 1099-1103 (2005)
Ren, C. P. et al., Acta Biochim Biophys Sin (Shanghai) 37, 68-73 (2005)
Rugg-Gunn, P. J.et al., Nat Genet 37, 585-587 (2005)
Schwartz, R. E. et al., Stem Cells Dev 14, 643-655 (2005)
Vallier, L. et al., J Cell Sci 118, 4495-4509 (2005)
Vodyanik, M. A. et al., Blood 105, 617-626 (2005)
Wang, L. et al., Blood 105, 4598-4603 (2005)
Wang, L. et al., J Exp Med 201, 1603-1614 (2005)

Wei, C. L. et al., Stem Cells 23, 166-185 (2005)
Wiblin, A. E., et al., J Cell Sci 118, 3861-3868 (2005)
Woll, P. S. et al., J Immunol 175, 5095-5103 (2005)
Xu, C. et al., Stem Cells 23, 315-323 (2005)
Xu, R. H. et al., Nat Methods 2, 185-190 (2005)
Yan, Y. et al., Stem Cells 23, 781-790 (2005)
Zaehres, H. et al., Stem Cells 23, 299-305 (2005)
Zhan, M., et al., Cell Biochem Biophys 43, 379-405 (2005)
Androutsellis-Theotokis, A. et al., Nature 442, 823-826 (2006)
Becker, K. A. et al., J Cell Physiol 209, 883-893 (2006)
Bibikova, M. et al., Genome Res 16, 1075-1083 (2006)
Bowles, K. M. et al., Stem Cells 24, 1359-1369 (2006)
Cameron, C. M. & Kaufman, D. S., Biotechnol Bioeng 94, 938-948 (2006)
Cerdan, C. et al., Nat Med 12, 1113-1114; author reply 1115 (2006)
Clements, M. O. et al., Tissue Eng 12, 1741-1751 (2006)
Darr, H. et al., Development 133, 1193-1201 (2006)
Drukker, M. et al., Stem Cells 24, 221-229 (2006)
Fang, D. et al., Stem Cells 24, 1668-1677 (2006)
Forsyth, N. R. et al., Cloning Stem Cells 8, 16-23 (2006)
Gaur, M. et al., J Thromb Haemost 4, 436-442 (2006)
Guillaume, D. J. et al., J Neurosci Res 84, 1165-1176 (2006)
Hewitt, Z. et al., Cloning Stem Cells 8, 225-234 (2006)
Iuchi, S. et al., Differentiation 74, 160-166 (2006)
Iuchi, S. et al., Proc Natl Acad Sci U S A 103, 1792-1797 (2006)
Ji, L. et al., Tissue Eng 12, 665-679 (2006)
Joannides, A. et al., Stem Cells 24, 230-235 (2006)
Josephson, R. et al., BMC Biol 4, 28 (2006)
Kameda, T. et al., Biochem Biophys Res Commun 349, 1269-1277 (2006)
Karp, J. M. et al., Stem Cells 24, 835-843 (2006)
Katkov, I. I. et al., Cryobiology 53, 194-205 (2006)
Kee, K. et al., Stem Cells Dev 15, 831-837 (2006)
Khademhosseini, A. et al., Biomaterials 27, 5968-5977 (2006)
Klimanskaya, I. et al., Nature 444, 481-485 (2006)
Lee, T. I. et al., Cell 125, 301-313 (2006)
Levenstein, M. E. et al., Stem Cells 24, 568-574 (2006)
Liu, Y. et al., Biochem Biophys Res Commun 346, 131-139 (2006)
Liu, Y. et al., BMC Dev Biol 6, 20 (2006)
Lu, J., et al., Proc Natl Acad Sci U S A 103, 5688-5693 (2006)
Ludwig, T. E. et al., Nat Biotechnol 24, 185-187 (2006)
Lund, R. D. et al., Cloning Stem Cells 8, 189-199 (2006)
Martinat, C. et al., Proc Natl Acad Sci U S A 103, 2874-2879 (2006)
Mohr, J. C. et al., Biomaterials 27, 6032-6042 (2006)
Mohr, J. C. et al., Biotechnol Prog 22, 825-834 (2006)

Plaia, T. W. et al., Stem Cells 24, 531-546 (2006)
Pyle, A. D. et al., Nat Biotechnol 24, 344-350 (2006)
Ren, C. et al., Stem Cells 24, 1338-1347 (2006)
Roy, N. S. et al., Nat Med 12, 1259-1268 (2006)
Saha, S. et al., J Cell Physiol 206, 126-137 (2006)
Slukvin, I. I. et al., J Immunol 176, 2924-2932 (2006)
Stewart, M.H. et al., Nat Methods 3, 807-815 (2006)
Sun, B. W. et al., Hum Mol Genet 15, 65-75 (2006)
Tian, X. et al., Stem Cells 24, 1370-1380 (2006)
Vodyanik, M. A. et al., Blood 108, 2095-2105 (2006)
Ware, C. B. et al, Stem Cells 24, 2677-2684 (2006)
Xu, X. et al., Cloning Stem Cells 8, 96-107 (2006)
Adewumi, O. et al., Nat Biotechnol 25, 803-816 (2007)
Barberi, T. et al., Nat Med, 13, 642-648 (2007)
Becker, K. A. et al., J Cell Physiol, 210, 517-526 (2007)
Bendall, S. C. et al., Nature 448, 1015-1021 (2007)
Blum, B. & Benvenisty N., Stem Cells 25, 1924-1930 (2007)
Boyd, N. L. et al.,Exp Biol Med (Maywood), 232, 833-843 (2007)
Bradbury, M. S. et al., J Neurochem 102, 2029-2039 (2007)
Brons, I. G. et al., Nature 448, 191-195 (2007)
Cai J. et al., Hepatology 45, 1229-1239 (2007)
Cai L. et al., Cell Res 17, 62-72 (2007)
Cezar, G. G. et al.,Stem Cells Dev 16, 869-882 (2007)
Chen, H. F. et al., Hum Reprod, 22, 567-577 (2007)
Chen, T. et al., Stem Cells 25, 392-401 (2007)
Choudhary, M. et al., Stem Cells 25, 3045-3057 (2007)
Das, P. et al., Stem Cell Res 1, 61-74 (2007)
Derda, R. et al., ACS Chem Biol 2, 347-355 (2007)
Dvash, T. et al., Stem Cells 25, 465-472 (2007)
Egozi, D. et al., Faseb J 21, 2807-2817 (2007)
Ferreira, L. S. et al., Biomaterials 28, 2706-2717 (2007)
Ferreira, L. S. et al., Circ Res 101, 286-294 (2007)
Figallo, E. et al., Lab Chip 7, 710-719 (2007)
Fox, M. S. et al., Dev Biol 301, 417-431 (2007)
Garcia-Perez, J. L. et al.,Hum Mol Genet,16, 1569-1577 (2007)
Gerecht S. et al., Proc Natl Acad Sci U S A 104, 11298-11303 (2007)
Gerecht, S. et al., Biomaterials 28, 4068-4077 (2007)
Gerecht, S. et al., Biomaterials 28, 4826-4835 (2007)
Gharwan, H. et al., Mol Ther 15, 1827-1833 (2007)
Ghule, P. N. et al., J Cell Physiol 213, 9-17 (2007)
Greber, B. et al., BMC Dev Biol 7, 46 (2007)
Greber, B. et al., Stem Cells 25, 455-464 (2007)
Grskovic, M. et al., PLoS Genet 3, e145 (2007)

Guenther, M. G. et al., Cell 130, 77-88 (2007)
Hewitt, Z. et al., Stem Cells 25, 10-18 (2007)
Hirst, M. et al., Genome Biol 8, R113 (2007)
Huang, Z. et al., J Cell Physiol 211, 816-825 (2007)
Iacovitti, L. et al., Brain Res 1127,19-25 (2007)
Jiang, J. et al., Stem Cells 25, 1940-1953 (2007)
Jiang, W. et al., Cell Res 17, 333-344 (2007)
Joannides, A. J. et al., Brain 130, 1263-1275 (2007)
Joannides, A. J. et al., Stem Cells 25, 731-737 (2007)
Johnson, M. A. et al., J Neurosci 27, 3069-3077 (2007)
Josephson, R. et al., Stem Cells 25, 437-446 (2007)
Kärner, E. et al., Stem Cells Dev 16, 39-52 (2007)
Kim, K. et al., Cell Stem Cell 1, 346-352 (2007)
Ko, J. Y. et al., J Neurochem 103, 1417-1429 (2007)
Krtolica, A. et al., Stem Cells 25, 2215-2223 (2007)
Lee, G. et al., Nat Biotechnol 25, 1468-1475 (2007)
Lee, H. et al., Stem Cells 25, 1931-1939 (2007)
Lee, J. P. et al., Nat Med 13, 439-447 (2007)
Li, J. et al., Differentiation 75, 299-307 (2007)
Lu, S. J. et al., Genome Biol 8, R240 (2007)
Lu, S. J. et al., Nat Methods 4, 501-509 (2007)
Lu, S. J. et al., Stem Cells Dev 16, 547-559 (2007)
Nicholas, C. R. et al., Stem Cells Dev 16,109-117 (2007)
Ohm, J. E. et al, Nat Genet 39, 237-242 (2007)
Pankratz, M. T. et al., Stem Cells 25, 1511-1520 (2007)
Peerani, R. et al., Embo J 26, 4744-4755 (2007)
Porayette, P. et al., Biochem Biophys Res Commun 364, 522-527 (2007)
Pruszak, J. et al., Stem Cells 25, 2257-2268 (2007)
Qin, H. et al., J Biol Chem 282, 5842-5852 (2007)
Rajesh, D. et al., Stem Cells 25, 490-499 (2007)
Schneider, B. L. et al., Hum Mol Genet 16, 651-666 (2007)
Schrattenholz, A. & Klemm M., Altex 24, 9-15 (2007)
Serobyan, N. et al., Life Sc ,80, 2352-6230 (2007)
Shi, F. et al., Eur J Neurosci 26, 3016-3023 (2007)
Shin, S. et al., Stem Cells 25, 1298-1306 (2007)
Soh, B. S. et al., Stem Cells 25, 3029-3037 (2007)
Sonntag, K. C. et al., Stem Cells 25, 411-418 (2007)
Sullivan, K. E. et al., Mol Cell Biol 27, 5147-5160 (2007)
Takahashi, K. et al., Cell 131, 861-872 (2007)
Tesar, P. J. et al., Nature 448, 196-199 (2007)
Toh, W. S. et al., Stem Cells 25, 950-960 (2007)
Trivedi, P. et al., Exp Hematol 35, 146-154 (2007)
Vallier, L. et al., Stem Cell ,25, 1490-1497 (2007)

Vieyra, D. S. et al., Stem Cells 25, 2559-2566 (2007)
Wang, Z. Z. et al., Nat Biotechnol 25, 317-318 (2007)
Wilber, A. et al., Stem Cells 25, 2919-2927 (2007)
Wilson, P. G. et al., Stem Cells Dev 16, 1027-1041 (2007)
Xia, X. et al., Stem Cells Dev 16, 167-176 (2007)
Xie, C. Q. et al., Arterioscler Thromb Vasc Biol 27, e311-e312 (2007)
Yang, M. J. et al., Biomacromolecules 8, 2746-2752 (2007)
Zhao, M. et al., Biochem Biophys Res Commun 362, 916-922 (2007)
Zhou, B. Y. et al., Stem Cells 25, 779-789 (2007)
Aberdam, E. et al., Stem Cells 26, 440-444 (2008)
Agarwal, S. et al., Stem Cells 26, 1117-1127 (2008)
Atkinson, S. P. et al., Stem Cells 26, 1174-1185 (2008)
Aubry, L. et al., Proc Natl Acad Sci U S A 105, 16707-16712 (2008)
Bajpai, R. et al., Mol Reprod Dev 75, 818-827 (2008)
Banuelos, C. A. et al., DNA Repair (Amst) 7, 1471-1483 (2008)
Bauwens, C. L. et al., Stem Cells 26, 2300-2310 (2008)
Bendall, S. C. et al., Mol Cell Proteomics 7, 1587-1597 (2008)
Bera, T. K. et al., Stem Cells Dev 17, 325-332 (2008)
Bonig, H. et al., Transfusion 48, 1039-1040 (2008)
Brink, T. C. et al., Cells Tissues Organs 188, 9-22 (2008)
Cameron, C. M. et al., Exp Biol Med (Maywood) 233, 1044-1057 (2008)
Cao, F. et al., PLoS ONE 3, e3474 (2008)
Cao, T. et al., Cloning Stem Cells 10, 1-10 (2008)
Chan, E. M. et al., Cloning Stem Cells 10, 107-118 (2008)
Chen, G. et al., Cell Stem Cell 2, 345-355 (2008)
Chen, Y. T. et al., Stem Cells Dev 17, 853-855 (2008)
Chiao, E. et al., Stem Cells 26, 2032-2041 (2008)
Choi, H. S. et al., Cell Tissue Res 333, 197-206 (2008)
Come, J. et al., Tissue Eng Part C Methods 14, 289-298 (2008)
Daadi, M. M. et al., PLoS ONE 3, e1644 (2008)
Desbordes, S. C. et al., Cell Stem Cell 2, 602-612 (2008)
Dhara, S. K. et al., Differentiation 76, 454-464 (2008)
Diecke, S. et al., Cells Tissues Organs 188, 52-61 (2008)
Elkabetz, Y. et al., Genes Dev 22, 152-165 (2008)
Erceg, S. et al., PLoS ONE 3, e2122 (2008)
Fletcher, J. et al., Cloning Stem Cells 10, 331-339 (2008)
Fong, H. et al., Stem Cells 26, 1931-1938 (2008)
Forsyth, N. R. et al., Regen Med 3, 817-833 (2008)
Forsyth, N. R. et al., Rejuvenation Res 11, 5-17 (2008)
Ghule, P. N. et al., Proc Natl Acad Sci U S A 105, 16964-16964 (2008)
Greber, B. et al., Stem Cells Dev 17, 1065-1078 (2008)
Hall, L. L. et al., J Cell Physiol 216, 445-452 (2008)
Harb, N. et al., PLoS ONE 3, e3001 (2008)

Hay, D. C. et al., Proc Natl Acad Sci U S A 105, 12301-12306 (2008)
Hikita, S. T. et al., PLoS ONE 3, e3312 (2008)
Hoben, G. M. et al., Stem Cells 26, 422-430 (2008)
Hohenstein, K. A. et al., Stem Cells 26, 1436-1443 (2008)
Hurst, J. H. et al., BMC Neurosci 9, 118 (2008)
Ivey, K. N. et al., Cell Stem Cell 2, 219-229 (2008)
Jaksch, M. et al., Cancer Res 68, 7882-7886 (2008)
Ji, J. et al., Stem Cells 26, 2485-2495 (2008)
Kiprilov, E. N. et al., J Cell Biol 180, 897-904 (2008)
Koay, E. J. et al., Osteoarthritis Cartilage 16, 1450-1456 (2008)
Ku, M. et al., PLoS Genet 4, e1000242 (2008)
Lai, B. et al., Stem Cells Dev 17, 565-572 (2008)
Lam, H. et al., Biochem Biophys Res Commun 372, 601-616 (2008)
Laurent, L. C. et al., Stem Cells 26, 1506-1516 (2008)
Ledran, M. H. et al., Cell Stem Cell 3, 85-93 (2008)
Lefort, N. et al., Nat Biotechnol 26, 1364-1366 (2008)
Levenstein, M. E. et al., Stem Cells 26, 3099-3107 (2008)
Li, X. et al., Stem Cells Dev 17, 1079-1085 (2008)
Li, X. J. et al., Stem Cells 26, 886-893 (2008)
Li, Z. et al., Stem Cells 26, 864-873 (2008)
Lowry, W. E. et al., Proc Natl Acad Sci U S A 105, 2883-2888 (2008)
Lu, S. J. et al., Blood 112, 4475-4484 (2008)
Maimets, T. et al., Oncogene 27, 5277-5287 (2008)
Mali, P. et al., Stem Cells 26, 1998-2005 (2008)
Martin, C. H. et al., Blood 112, 2730-2737 (2008)
Maurer, J. et al., PLoS ONE 3, e3451 (2008)
Mayshar, Y. et al., Stem Cells 26, 767-747 (2008)
McElroy, S. L. et al., Reprod Biomed Online 16, 684-693 (2008)
Metallo, C. M. et al., Biotechnol Bioeng 100, 830-837 (2008)
Metallo, C. M. et al., Stem Cells 26, 372-380 (2008)
Morin, R. D. et al., Genome Res 18, 610-621 (2008)
Mouffouk, F. et al., Anal Biochem 372, 140-147 (2008)
Mujoo, K. et al., Proc Natl Acad Sci U S A 105, 18924-18929 (2008)
Muller, F. J. et al., Nature 455, 401-405 (2008)
Nakagawa, M. et al., Nat Biotechnol 26, 101-106 (2008)
O'Connor, M. D. et al., Stem Cells 26, 1109-1116 (2008)
Pereira, C. F. et al., PLoS Genet 4, e1000170 (2008)
Phanstiel, D. et al., Proc Natl Acad Sci U S A 105, 4093-4098 (2008)
Postovit, L. M. et al., Proc Natl Acad Sci U S A 105, 4329-4334 (2008)
Qiu, D. et al., Biochem Biophys Res Commun 369, 735-740 (2008)
Sachlos, E. et al., Biomaterials 29, 4471-4480 (2008)
Saha, S. et al., Biophys J 94, 4123-4133 (2008)
Shen, Y. et al., Proc Natl Acad Sci U S A 105, 4709-4714 (2008)

Shojaei, F. et al., Exp Hematol 36, 1442-1454 (2008)
Silva, S. S. et al., Proc Natl Acad Sci U S A 105, 4820-4825 (2008)
Smith, J. R. et al., Dev Biol 313, 107-117 (2008)
Smith, J. R. et al., Stem Cells 26, 496-504 (2008)
Stewart, R. et al., Regen Med 3, 505-522 (2008)
Su, Z. et al., Clin Cancer Res 14, 6207-6217 (2008)
Swijnenburg, R. J. et al., Proc Natl Acad Sci U S A 105, 12991-12996 (2008)
Tateishi, K. et al., J Biol Chem 283, 31601-31697 (2008)
Thomson, A. et al., Cloning Stem Cells 10, 89-106 (2008)
Tilgner, K. et al., Stem Cells 26, 3075-3085 (2008)
Tremoleda, J. L. et al., Cloning Stem Cells 10, 119-132 (2008)
Trivedi, P. et al., Exp Hematol 36, 350-359 (2008)
Unger, C. et al., Stem Cells 26, 2455-2466 (2008)
Ungrin, M. D. et al., PLoS ONE 3, e1565 (2008)
Valbuena, D. et al., Reprod Biomed Online 17, 127-135 (2008)
Varelas, X. et al., Nat Cell Biol 10, 837-848 (2008)
West, M. D. et al., Regen Med 3, 287-308 (2008)
Westfall, S. D. et al., Stem Cells Dev 17, 869-881 (2008)
Woll, P. S. et al., Blood 111, 122-131 (2008)
Xia, X. et al., Stem Cells 26, 525-533 (2008)
Xu, R. H. et al., Cell Stem Cell 3, 196-206 (2008)
Yang, D. et al., Stem Cells 26, 55-63 (2008)
Yu, X. et al., Cell Stem Cell 2, 461-471 (2008)
Zambidis, E. T. et al., Blood 112, 3601-3614 (2008)
Zdravkovic, T. et al., Reprod Toxicol 26, 86-93 (2008)
Zhan, X. et al., Cloning Stem Cells 10, 513-522 (2008)
Zhang, P. et al., Blood 111, 1933-1941 (2008)
Zhao, M. et al., Mol Biotechnol 40, 19-26 (2008)
Zhong, J. F. et al., Lab Chip 8, 68-74 (2008)
Zhou, Y. et al., Biochem Biophys Res Commun 376, 542-547 (2008)

H9 EBNA1 (EBNA1-H9, derivative of H9)

Ren, C. P. et al., Acta Biochim Biophys Sin (Shanghai) 37, 68-73 (2005)
Lee, M. H. et al., PLoS Genet 3, e233 (2007)

H9.1 (GE91, subline of H9)

Amit, M. et al., Dev Biol 227, 271-278 (2000)
Schuldiner, M. et al., Proc Natl Acad Sci U S A 97, 11307-11312 (2000)
Xu, C. et al., Circ Res 91, 501-508 (2002)
Tzukerman, M. et al., Proc Natl Acad Sci U S A 100, 13507-13512 (2003)
Tzukerman, M. et al., Cancer Res 66, 3792-3801 (2006)

H9.2 (GE92, subline of H9)

Amit, M. et al., Dev Biol 227, 271-278 (2000)

Kaufman, D. S. et al., Proc Natl Acad Sci U S A 98, 10716-10721 (2001)

Kehat, I. et al., J Clin Invest 108, 407-414 (2001)

Zhang, S. C. et al., Nat Biotechnol 19, 1129-1133 (2001)

Amit, M. & Itskovitz-Eldor, J., J Anat 200, 225-232 (2002)

Kehat, I. et al., Circ Res 91, 659-661 (2002)

Xu, C. et al., Circ Res 91, 501-508 (2002)

Gerecht-Nir, S. et al., Lab Invest 83, 1811-1820 (2003)

Snir, M. et al., Am J Physiol Heart Circ Physiol 285, H2355 (2003)

Dang, S. M. et al., Stem Cells 22, 275-282 (2004)

Gerecht-Nir, S. et al., Biol Reprod 71, 2029-2036 (2004)

Gerecht-Nir, S. et al., Biotechnol Bioeng 86, 493-502 (2004)

Gerecht-Nir, S. et al., Biotechnol Bioeng 88, 313-320 (2004)

Ginis, I. et al., Dev Biol 269, 360-380 (2004)

Kehat, I. et al., Nat Biotechnol 22, 1282-1289 (2004)

Magyar, J. et al., Cardiovasc Res 64, 477-487 (2004)

Satin, J. et al., J Physiol-London 559, 479-496 (2004)

Segev, H. et al., Stem Cells 22, 265-274 (2004)

Dolnikov, K. et al., Ann N Y Acad Sci 1047, 66-75 (2005)

Gerecht-Nir, S. et al., Dev Dyn 232, 487-497 (2005)

Lev, S. et al., Ann N Y Acad Sci 1047, 50-65 (2005)

Segev, H. et al., Dev Growth Differ 47, 295-306 (2005)

Siemen, H. et al., Stem Cells Dev 14, 378-383 (2005)

Benzing, C. et al., Neuroreport 17, 1675-1681 (2006)

Dolnikov, K. et al., Stem Cells 24, 236-245 (2006)

Koch, P et al., O. Nucleic Acids Res 34, e120 (2006)

Nolden, L. et al., Nat Methods 3, 461-467 (2006)

Caspi O. et al., Circ Res 100, 263-272 (2007)

Caspi O. et al., J Am Coll Cardiol 50, 1884-1893 (2007)

Huber, I. et al., Faseb J 21, 2551-2563 (2007)

Leor, J. et al., Heart 93, 1278-1284 (2007)

Terstegge, S. et al., Biotechnol Bioeng 96, 195-201 (2007)

Wang, D. et al., Proc Natl Acad Sci U S A 104, 4449-4454 (2007)

Ladewig, J. et al., Stem Cells 26, 1705-1712 (2008)

Satin, J. et al., Stem Cells 26, 1961-1972 (2008)

Sedan, O. et al., Stem Cells 26, 3130-3138 (2008)

Yirme, G. et al., Stem Cells Dev 17, 1227-1241 (2008)

HAD 1

Provider: Hadassah University Medical Center, Jerusalem, Israel

Turetsky, T. et al., Hum Reprod 23, 46-53 (2008)

HAD 2

Provider: Hadassah University Medical Center, Jerusalem, Israel

Turetsky, T. et al., Hum Reprod 23, 46-53 (2008)

HAD 3

Provider: Hadassah University Medical Center, Jerusalem, Israel

Turetsky, T. et al., Hum Reprod 23, 46-53 (2008)

HAD 5

Provider: Hadassah University Medical Center, Jerusalem, Israel

Turetsky, T. et al., Hum Reprod 23, 46-53 (2008)

HEFX

Provider: Hadassah University Medical Center, Jerusalem, Israel

Eiges, R. et al., Cell Stem Cell 1, 568-577 (2007)

HES-1 (ES01)

Provider: ES Cell International Pte Ltd, Singapore Pte Ltd, Singapore

Reubinoff, B. E. et al., Nat Biotechnol 18, 399-404 (2000)

Reubinoff, B. E. et al., Hum Reprod 16, 2187-2194 (2001)

Reubinoff, B. E. et al., Nat Biotechnol 19, 1134 (2001)

Cooper, S. et al., J Anat 200, 259-265 (2002)

Drukker, M. et al., Proc Natl Acad Sci U S A 99, 9864-9869 (2002)

Goldstein, R. S. et al., Dev Dynam 225, 80-86 (2002)

Gropp, M. et al., Mol Ther 7, 281-287 (2003)

Ben-Hur, T. et al., Stem Cells 22, 1246-1255 (2004)

Buzzard, J. J. et al., Nat Biotechnol 22, 381-282 (2004)

Zhou, C. Q. et al., Chin Med J (Engl) 117, 1050-1055 (2004)

Itsykson, P. et al., Mol Cell Neurosci 30, 24-36 (2005)

Pomp, O. et al., Stem Cells 23, 923-930 (2005)

Wu, C.F. et al., Reprod Biomed Online 11, 733-739 (2005)

Banin, E. et al., Stem Cells 24, 246-257 (2006)

Ben-Dor, I. et al., Mol Ther 14, 255-267 (2006)

Ware, C. B. et al, Stem Cells 24, 2677-2684 (2006)

Adewumi, O. et al., Nat Biotechnol 25, 803-816 (2007)

Ben-Hur, T. et al., Magn Reson Med 57, 164-171 (2007)

Biton, S. et al., DNA Repair (Amst) 6, 128-134 (2007)

Gropp, M. & Reubinoff E., Cloning Stem Cells 9, 339-345 (2007)

Phillips, B. W. et al., Stem Cells Dev 16, 561-578 (2007)

Shin, S. et al., Stem Cells 25, 1298-1306 (2007)

Tesar, P. J. et al., Nature 448, 196-199 (2007)

Zangrossi, S. et al., Stem Cells 25, 1675-1680 (2007)

Zeng, J. et al., Stem Cells 25, 1055-61 (2007)

Aharonowiz, M. et al., PLoS ONE 3, e3145 (2008)

Brokhman, I. et al., Differentiation 76, 145-155 (2008)

Hall, L. L. et al., J Cell Physiol 216, 445-452 (2008)

Muller, F. J. et al., Nature 455, 401-405 (2008)

Tzur, G. et al., PLoS ONE 3, e3726 (2008)

---

hES-18

Provider: Huazhong University of Science and Technology, Wuhan, China

Chen, H. et al., Hum Reprod 20, 2201 (2005)

HES-2 (ES02)

Provider: ES Cell International Pte Ltd, Singapore Pte Ltd, Singapore

Reubinoff, B. E. et al., Nat Biotechnol 18, 399-404 (2000)

Reubinoff, B. E. et al., Hum Reprod 16, 2187-2194 (2001)

Cooper, S. et al., J Anat 200, 259-265 (2002)

Mummery, C. et al., J Anat 200, 233-242 (2002)

Sathananthan, H. et al., Reprod Biomed Online 4, 56-61 (2002)

Mummery, C. et al., Circulation 107, 2733-2740 (2003)

Buzzard, J. J. et al., Nat Biotechnol 22, 381-282 (2004)

Choo, A. B. et al., Biotechnol Bioeng 88, 321-331 (2004)

Conley, B. J. et al., Fetal Diagn Ther 19, 218-223 (2004)

Pera, M. F. et al., J Cell Sci 117, 1269-1280 (2004)

Richards, M. et al., Stem Cells 22, 779-789 (2004)

Itsykson, P. et al., Mol Cell Neurosci 30, 24-36 (2005)

Maitra, A. et al., Nat Genet 37, 1099-1103 (2005)

Ng, E. S. et al., Blood 106, 1601-1603 (2005)

Passier, R. et al., Stem Cells 23, 772-780 (2005)

Pebay, A. et al., Stem Cells 23, 1541-1548 (2005)

Stamp, L. et al., Stem Cells 23, 103-112 (2005)

Tan, S. M. & Droge, P., Stem Cells 23, 868-873 (2005)

van de Stolpe, A. et al., Reprod Biomed Online 11, 476-485 (2005)

Wei, C. L. et al., Stem Cells 23, 166-185 (2005)

Beqqali, A., et al., Stem Cells 24, 1956-1967 (2006)

Bibikova, M. et al., Genome Res 16, 1075-1083 (2006)

Choo, A. et al., J Biotechnol 122, 130-141 (2006)

Herszfeld, D. et al., Nat Biotechnol 24, 351-357 (2006)

Hirst, C. E. et al., Dev Biol 293, 90-103 (2006)

Josephson, R. et al., BMC Biol 4, 28 (2006)

Li, O. et al., Genesis 44, 523-529 (2006)

Taylor, R. A. et al., Nat Methods 3, 179-181 (2006)

Van Hoof, D. et al., Mol Cell Proteomics 5, 1261-1273 (2006)

Ware, C. B. et al, Stem Cells 24, 2677-2684 (2006)

Adewumi, O. et al., Nat Biotechnol 25, 803-816 (2007)
Allegrucci, C. et al., Hum Mol Genet, 16, 1253-1268 (2007)
Chin, A. C. et al., J Biotechnol 130, 320-328 (2007)
Conley, B. J. et al., Biochem Cell Biol 85, 121-132(2007)
Denham, M. et al., Am J Physiol Lung Cell Mol Physiol 292, L1241-1247 (2007)
Filipczyk, A. A. et al., Cell Mol Life Sci 64, 704-718 (2007)
Irion, S. et al., Nat Biotechnol 25, 1477-1482 (2007)
Kennedy, M. et al., Blood 109, 2679-2687 (2007)
Kim, K. P. et al., Genome Res 17, 1731-1742 (2007)
Lakshmipathy, U. et al., Stem Cells Dev 16, 1003-1016 (2007)
Laslett, A. L. et al., BMC Dev Biol 7, 12 (2007)
Liu, J. et al., Stem Cells 25, 3038-3044 (2007)
Phillips, B. W. et al., Stem Cells Dev 16, 561-578 (2007)
Tan, S. M. et al., Nucleic Acids Res 35, e118 (2007)
Wolvetang, E. J. et al., Biochem Biophys Res Commun 363, 610-615 (2007)
Bera, T. K. et al., Stem Cells Dev 17, 325-332 (2008)
Braam, S. R. et al., Nat Methods 5, 389-392 (2008)
Braam, S. R. et al., Stem Cells 26, 2257-2265 (2008)
Chan, K. K. et al., Stem Cells Dev 17, 825-836 (2008)
Choo, A. B. et al., Stem Cells 26, 1454-1463 (2008)
Dottori, M. et al., Stem Cells 26, 1146-1154 (2008)
Freund, C. et al., Stem Cells 26, 724-733 (2008)
Graichen, R. et al., Differentiation 76, 357-370 (2008)
Hall, L. L. et al., J Cell Physiol 216, 445-452 (2008)
Monk, M. et al., Mol Hum Reprod 14, 347-355 (2008)
Moore, J. C. et al., Biochem Biophys Res Commun 372, 553-558 (2008)
Muller, F. J. et al., Nature 455, 401-405 (2008)
Peiffer, I. et al., Stem Cells Dev 17, 519-533 (2008)
Silva, S. S. et al., Proc Natl Acad Sci U S A 105, 4820-4825 (2008)
Tzur, G. et al., PLoS ONE 3, e3726 (2008)
Van Hoof, D. et al., Stem Cells 26, 2777-2781 (2008)
Xu, X. Q. et al., Differentiation 76, 958-970 (2008)
Yang, L. et al., Nature 453, 524-528 (2008)

HES-3 (ES03)

Provider: ES Cell International Pte Ltd, Singapore Pte Ltd, Singapore

Cooper, S. et al., J Anat 200, 259-265 (2002)
Richards, M. et al., Nat Biotechnol 20, 933-936 (2002)
Pickering, S. J. et al., Reprod Biomed Online 7, 353-364 (2003)
Richards, M. et al., Stem Cells 21, 546-556 (2003)
Buzzard, J. J. et al., Nat Biotechnol 22, 381-282 (2004)
Choo, A. B. et al., Biotechnol Bioeng 88, 321-331 (2004)
Pera, M. F. et al., J Cell Sci 117, 1269-1280 (2004)

Perrier, A. L. et al., Proc Natl Acad Sci U S A 101, 12543-12548 (2004)
Richards, M. et al., Stem Cells 22, 51-64 (2004)
Richards, M. et al., Stem Cells 22, 779-789 (2004)
Wong, R. C. B. et al., Stem Cells 22, 883-889 (2004)
Costa, M. et al., Nat Methods 2, 259-260 (2005)
Fong, W. J. et al., Bioprocess Biosyst Eng 27, 381-387 (2005)
Khoo, M. L., et al., Biol Reprod 73, 1147-1156 (2005)
Maitra, A. et al., Nat Genet 37, 1099-1103 (2005)
Mossman, A. K. et al., Stem Cells Dev 14, 656-663 (2005)
Ng, E. S. et al., Blood 106, 1601-1603 (2005)
Passier, R. et al., Stem Cells 23, 772-780 (2005)
Pebay, A. et al., Stem Cells 23, 1541-1548 (2005)
Prowse, A. B. et al., Proteomics 5, 978-989 (2005)
Rugg-Gunn, P. J.et al., Nat Genet 37, 585-587 (2005)
Stamp, L. et al., Stem Cells 23, 103-112 (2005)
Tabar, V. et al., Nat Biotechnol 23, 601-616 (2005)
van de Stolpe, A. et al., Reprod Biomed Online 11, 476-485 (2005)
Bibikova, M. et al., Genome Res 16, 1075-1083 (2006)
Chen, S.& Oh, S. K., J Biotechnol 122, 341-361 (2006)
Choo, A. et al., J Biotechnol 122, 130-141 (2006)
Dean, S. K. et al., Transplantation 82, 1175-1184 (2006)
Ding, V. et al., Biotechnol Lett 28, 491-495 (2006)
Herszfeld, D. et al., Nat Biotechnol 24, 351-357 (2006)
Josephson, R. et al., BMC Biol 4, 28 (2006)
Richards, M. et al., Stem Cells 24, 1162-1173 (2006)
Sidhu, K. S. & Tuch, B. E. Stem Cells Dev 15, 61-69 (2006)
Ware, C. B. et al, Stem Cells 24, 2677-2684 (2006)
Wong, R. C. B. et al., Biochem Biophys Res Commun 344, 181-188 (2006)
Adewumi, O. et al., Nat Biotechnol 25, 803-816 (2007)
Bendall, S. C. et al., Nature 448, 1015-1021 (2007)
Chin, A. C. et al., J Biotechnol 130, 320-328 (2007)
Davidson, K. C. et al., Mol Cell Neurosci 36, 408-415 (2007)
Filipczyk, A. A. et al., Cell Mol Life Sci 64, 704-718 (2007)
Gauthaman, K. et al., Reprod Biomed Online 15, 566-581 (2007)
Hirst, M. et al., Genome Biol 8, R113 (2007)
Irion, S. et al., Nat Biotechnol 25, 1477-1482 (2007)
Lakshmipathy, U. et al., Stem Cells Dev 16, 1003-1016 (2007)
Laslett, A. L. et al., BMC Dev Biol 7, 12 (2007)
Lees, J. G. et al., Regen Med 2, 289-300 (2007)
Peiffer, I. et al., Stem Cells Dev 16, 393-402 (2007)
Phillips, B. W. et al., Stem Cells Dev 16, 561-578 (2007)
Pick, M. et al., Stem Cells 25, 2206-2214 (2007)
Shin, S. et al., Stem Cells 25, 1298-1306 (2007)

Sone, M. et al., Arterioscler Thromb Vasc Biol 27, 2127-2134 (2007)
Wolvetang, E. J. et al., Biochem Biophys Res Commun 363, 610-615 (2007)
Wong, R. C. et al., Stem Cells Dev 16, 989-1001 (2007)
Zhao, X. D. et al., Cell Stem Cell 1, 286-298 (2007)
Bera, T. K. et al., Stem Cells Dev 17, 325-332 (2008)
Chan, K. K. et al., Stem Cells Dev 17, 825-836 (2008)
Choo, A. B. et al., Stem Cells 26, 1454-1463 (2008)
Davis, R. P. et al., Blood 111, 1876-1884 (2008)
Dottori, M. et al., Stem Cells 26, 1146-1154 (2008)
Graichen, R. et al., Differentiation 76, 357-370 (2008)
Hall, L. L. et al., J Cell Physiol 216, 445-452 (2008)
Muller, F. J. et al., Nature 455, 401-405 (2008)
Oyamada, N. et al., J Transl Med 6, 54 (2008)
Peiffer, I. et al., Stem Cells Dev 17, 519-533 (2008)
Silva, S. S. et al., Proc Natl Acad Sci U S A 105, 4820-4825 (2008)
Sumi, T. et al., Development 135, 2969-2679 (2008)
Xu, X. Q. et al., Cytotherapy 10, 376-389 (2008)
Yamahara, K. et al., PLoS ONE 3, e1666 (2008)
Zambidis, E. T. et al., Blood 112, 3601-3614 (2008)

HES3.1 (derivative of HES3)

Provider: Prince of Wales Hospital, Sidney, & Illawarra Area Health Service, NSW, Australia

Lim, U. M. et al., Curr Neurovasc Res 3, 281-288 (2006)
Sidhu, K. S. & Tuch, B. E. Stem Cells Dev 15, 61-69 (2006)

HES3.2 (derivative of HES3)

Provider: Prince of Wales Hospital, Sidney, & Illawarra Area Health Service, NSW, Australia

Lim, U. M. et al., Curr Neurovasc Res 3, 281-288 (2006)
Sidhu, K. S. & Tuch, B. E. Stem Cells Dev 15, 61-69 (2006)

HES3.3 (derivative of HES3)

Provider: Prince of Wales Hospital, Sidney, & Illawarra Area Health Service, NSW, Australia

Lim, U. M. et al., Curr Neurovasc Res 3, 281-288 (2006)
Sidhu, K. S. & Tuch, B. E. Stem Cells Dev 15, 61-69 (2006)

HES-3.gfp (ENVY, derivative of HES3)

Provider: Monash University, Clayton, Victoria, Australia

Costa, M. et al., Nat Methods 2, 259-260 (2005)
Ng, E. S. et al., Blood 106, 1601-1603 (2005)
Sidhu, K. S. & Tuch, B. E. Stem Cells Dev 15, 61-69 (2006)
Sidhu, K. S. et al., Stem Cells Dev 15, 741-747 (2006)
Dai, W. et al., J Mol Cell Cardiol 43, 504-516 (2007)
Lees, J. G. et al., Regen Med 2, 289-300 (2007)

Wolvetang, E. J. et al., Biochem Biophys Res Commun 363, 610-615 (2007)
Braam, S. R. et al., Nat Methods 5, 389-392 (2008)
Freund, C. et al., Stem Cells 26, 724-733 (2008)
Graichen, R. et al., Differentiation 76, 357-370 (2008)
van Laake, L. W. et al., Circ Res 102, 1008-1010 (2008)
Xu, X. Q. et al., Differentiation 76, 958-970 (2008)

HES-4 (ES04)

Provider: ES Cell International Pte Ltd, Singapore Pte Ltd, Singapore

Cooper, S. et al., J Anat 200, 259-265 (2002)
Richards, M. et al., Nat Biotechnol 20, 933-936 (2002)
Richards, M. et al., Stem Cells 21, 546-556 (2003)
Buzzard, J. J. et al., Nat Biotechnol 22, 381-282 (2004)
Choo, A. B. et al., Biotechnol Bioeng 88, 321-331 (2004)
Richards, M. et al., Stem Cells 22, 51-64 (2004)
Richards, M. et al., Stem Cells 22, 779-789 (2004)
Wong, R. C. B. et al., Stem Cells 22, 883-889 (2004)
Ng, E. S. et al., Blood 106, 1601-1603 (2005)
Passier, R. et al., Stem Cells 23, 772 (2005)
Pebay, A. et al., Stem Cells 23, 1541-8 (2005)
Choo, A. et al., J Biotechnol 122, 130-141 (2006)
Herszfeld, D. et al., Nat Biotechnol 24, 351-357 (2006)
Taylor, R. A. et al., Nat Methods 3, 179-181 (2006)
Ward, C. M. et al., Exp Cell Res 312, 1713-1726 (2006)
Ware, C. B. et al, Stem Cells 24, 2677-2684 (2006)
Adewumi, O. et al., Nat Biotechnol 25, 803-816 (2007)
Eastham, A. M. et al., Cancer Res 67, 11254-11562 (2007)
Hirst, M. et al., Genome Biol 8, R113 (2007)
Lakshmipathy, U. et al., Stem Cells Dev 16, 1003-1016 (2007)
Laslett, A. L. et al., BMC Dev Biol 7, 12 (2007)
Phillips, B. W. et al., Stem Cells Dev 16, 561-578 (2007)
Bera, T. K. et al., Stem Cells Dev 17, 325-332 (2008)
Choo, A. B. et al., Stem Cells 26, 1454-1463 (2008)
Dottori, M. et al., Stem Cells 26, 1146-1154 (2008)
Graichen, R. et al., Differentiation 76, 357-370 (2008)
Monk, M. et al., Mol Hum Reprod 14, 347-355 (2008)
Muller, F. J. et al., Nature 455, 401-405 (2008)

HES-5 (ES05)

Provider: ES Cell International Pte Ltd, Singapore Pte Ltd., Singapore

Buzzard, J. J. et al., Nat Biotechnol 22, 381-282 (2004)

Herszfeld, D. et al., Nat Biotechnol 24, 351-357 (2006)

Ware, C. B. et al, Stem Cells 24, 2677-2684 (2006)

Adewumi, O. et al., Nat Biotechnol 25, 803-816 (2007)

Zangrossi, S. et al., Stem Cells 25, 1675-1680 (2007)

HES-6 (ES06)

Provider: ES Cell International Pte Ltd, Singapore Pte Ltd., Singapore

Buzzard, J. J. et al., Nat Biotechnol 22, 381-282 (2004)

Herszfeld, D. et al., Nat Biotechnol 24, 351-357 (2006)

Ware, C. B. et al, Stem Cells 24, 2677-2684 (2006)

hES-8

Provider: Huazhong University of Science and Technology, Wuhan, China

Chen, H. et al., Hum Reprod 20, 2201-2206 (2005)

hESC-NL1 (formerly NL-hESC1)

Provider: Hubrecht Laboratory, Utrecht, The Netherlands

van de Stolpe, A. et al., Reprod Biomed Online 11, 476-485 (2005)

Van Hoof, D. et al., Mol Cell Proteomics 5, 1261-1273 (2006)

Adewumi, O. et al., Nat Biotechnol 25, 803-816 (2007)

Braam, S. R. et al., Nat Methods 5, 389-392 (2008)

Xu, X. Q. et al., Differentiation 76, 958-970 (2008)

HESC-NL2 (formerly NL-hESC2)

Provider: Hubrecht Laboratory, Utrecht, The Netherlands

Braam, S. R. et al., Nat Methods 5, 389-392 (2008)

Xu, X. Q. et al., Differentiation 76, 958-970 (2008)

HESC-NL3

Provider: Hubrecht Laboratory, Utrecht, The Netherlands

Braam, S. R. et al., Nat Methods 5, 389-392 (2008)

Braam, S. R. et al., Stem Cells 26, 2257-2265 (2008)

Freund, C. et al., Stem Cells 26, 724-733 (2008)

HESC-NL4

Provider: Hubrecht Laboratory, Utrecht, The Netherlands

Braam, S. R. et al., Nat Methods 5, 389-392 (2008)

Freund, C. et al., Stem Cells 26, 724-733 (2008)

HS181

Provider: Karolinska Institut, Stockholm, Sweden

Hovatta, O. et al., Hum Reprod 18, 1404-1409 (2003)
Gertow, K. et al., Stem Cells Dev 13, 421-435 (2004)
Imreh, M. P. et al., Stem Cells Dev 13, 337-343 (2004)
Inzunza, J. et al., Mol Hum Reprod 10, 461-466 (2004)
Inzunza, J. et al., Stem Cells 23, 544-549 (2005)
Skottman, H. et al., Stem Cells 23, 1343-1356 (2005)
Aghajanova, L. et al., Fertil Steril 86 Suppl 4, 1193-1209 (2006)
Imreh, M. P. et al., J Cell Biochem 99, 508-516 (2006)
Lowell, S. et al., PLoS Biol 4, e121 (2006)
Skottman, H. et al., Stem Cells 24, 151-167 (2006)
Adewumi, O. et al., Nat Biotechnol 25, 803-816 (2007)
Cedervall J. et al., Laryngoscope 117, 2075-2081 (2007)
Gertow, K. et al., J Cell Biochem 100, 1518-1525 (2007)
Kärner, E. et al., Stem Cells Dev 16, 39-52 (2007)
Nat, R. et al., Glia 55, 385-399 (2007)
Nieto, A. et al., Cell Biol Int 31, 269-278 (2007)
Rajala, K. et al., Hum Reprod 22, 1231-1238 (2007)
Zhang, P. et al., Fertil Steril 87, 677-690 (2007)
Barroso-delJesus, A. et al., Mol Cell Biol 28, 6609-6619 (2008)
Calvanese, V. et al., PLoS ONE 3, e3294 (2008)
Catalina, P. et al., Mol Cancer 7, 76- (2008)
Cobo, F. et al., Cloning Stem Cells 10, 65-74 (2008)
Lappalainen, R. S. et al., Neurosci Lett 440, 246-250 (2008)
Unger, C. et al., Stem Cells 26, 2455-2466 (2008)

HS207

Provider: Karolinska Institut, Stockholm, Sweden

Hovatta, O. et al., Hum Reprod 18, 1404-1409 (2003)
Martin-Ibanez, R. et al., Hum Reprod 23, 2744-2755 (2008)

HS235

Provider: Karolinska Institut, Stockholm, Sweden

Inzunza, J. et al., Mol Hum Reprod 10, 461-466 (2004)
Inzunza, J. et al., Stem Cells 23, 544-549 (2005)
Skottman, H. et al., Stem Cells 23, 1343-1356 (2005)
Aghajanova, L. et al., Fertil Steril 86 Suppl 4, 1193-1209 (2006)
Skottman, H. et al., Stem Cells 24, 151-167 (2006)
Assou ,S. et al., Stem Cells, 25, 961-973 (2007)

HS237

Provider: Karolinska Institut, Stockholm, Sweden

Inzunza, J. et al., Mol Hum Reprod 10, 461-466 (2004)

Koivisto, H. et al., Reprod Biomed Online 9, 330-337 (2004)

Nat, R., Hovatta, O., J Cell Mol Med 8, 570-571 (2004)

Inzunza, J. et al., Stem Cells 23, 544-549 (2005)

Skottman, H. et al., Stem Cells 23, 1343-1356 (2005)

Aghajanova, L. et al., Fertil Steril 86 Suppl 4, 1193-1209 (2006)

Grinnemo, K. H. et al., Reprod Biomed Online 13, 712-724 (2006)

Skottman, H. et al., Stem Cells 24, 151-167 (2006)

Kärner, E. et al., Stem Cells Dev 16, 39-52 (2007)

Narkilahti, S. et al., Biomed Eng Online 6, 11 (2007)

Nat, R. et al., Glia 55, 385-399 (2007)

Rajala, K. et al., Hum Reprod 22, 1231-1238 (2007)

Zhang, P. et al., Fertil Steril 87, 677-690 (2007)

HS293

Provider: Karolinska Institut, Stockholm, Sweden

Inzunza, J. et al., Stem Cells 23, 544-549 (2005)

Aghajanova, L. et al., Fertil Steril 86 Suppl 4, 1193-1209 (2006)

Grinnemo, K. H. et al., Reprod Biomed Online 13, 712-724 (2006)

Assou, S. et al., Stem Cells, 25, 961-973 (2007)

Cabrera, C. M. et al., Cell Biol Int 31, 1072-1078 (2007)

Narkilahti, S. et al., Biomed Eng Online 6, 11 (2007)

Nat, R. et al., Glia 55, 385-399 (2007)

Rajala, K. et al., Hum Reprod 22, 1231-1238 (2007)

Barroso-delJesus, A. et al., Mol Cell Biol 28, 6609-6619 (2008)

Cobo, F. et al., Cloning Stem Cells 10, 65-74 (2008)

HS306

Provider: Karolinska Institut, Stockholm, Sweden

Inzunza, J. et al., Stem Cells 23, 544-549 (2005)

Grinnemo, K. H. et al., Reprod Biomed Online 13, 712-724 (2006)

Kärner, E. et al., Stem Cells Dev 16, 39-52 (2007)

Nat, R. et al., Glia 55, 385-399 (2007)

Rajala, K. et al., Hum Reprod 22, 1231-1238 (2007)

van Harmelen, V. et al., Obesity (Silver Spring) 15, 846-852 (2007)

HS346

Provider: Karolinska Institut, Stockholm, Sweden

Inzunza, J. et al., Stem Cells 23, 544-549 (2005)

Grinnemo, K. H. et al., Reprod Biomed Online 13, 712-724 (2006)

Nat, R. et al., Glia 55, 385-399 (2007)

HS351

Provider: Karolinska Institut, Stockholm, Sweden

Inzunza, J. et al., Stem Cells 23, 544-549 (2005)

HS356

Provider: Karolinska Institut, Stockholm, Sweden

Inzunza, J. et al., Stem Cells 23, 544-549 (2005)

HS360

Provider: Karolinska Institut, Stockholm, Sweden

Inzunza, J. et al., Stem Cells 23, 544-549 (2005)

HS361

Provider: Karolinska Institut, Stockholm, Sweden

Inzunza, J. et al., Stem Cells 23, 544-549 (2005)

HS362

Provider: Karolinska Institut, Stockholm, Sweden

Inzunza, J. et al., Stem Cells 23, 544-549 (2005)

Grinnemo, K. H. et al., Reprod Biomed Online 13, 712-724 (2006)

HS363

Provider: Karolinska Institut, Stockholm, Sweden

Inzunza, J. et al., Stem Cells 23, 544-549 (2005)

Grinnemo, K. H. et al., Reprod Biomed Online 13, 712-724 (2006)

HS364

Provider: Karolinska Institut, Stockholm, Sweden

Inzunza, J. et al., Stem Cells 23, 544-549 (2005)

HS366

Provider: Karolinska Institut, Stockholm, Sweden

Inzunza, J. et al., Stem Cells 23, 544-549 (2005)

HS368

Provider: Karolinska Institut, Stockholm, Sweden

Inzunza, J. et al., Stem Cells 23, 544-549 (2005)

Grinnemo, K. H. et al., Reprod Biomed Online 13, 712-724 (2006)

HS382

Provider: Karolinska Institut, Stockholm, Sweden

Nat, R. et al., Glia 55, 385-399 (2007)

HS401

Provider: Karolinska Institut, Stockholm, Sweden

Grinnemo, K. H. et al., Stem Cells 26, 1850-1857 (2008)

Martin-Ibanez, R. et al., Hum Reprod 23, 2744-2755 (2008)

HS415

Provider: Karolinska Institut, Stockholm, Sweden

Ström, S. et al., Hum Reprod 22, 3051-3058 (2007)

HS420

Provider: Karolinska Institut, Stockholm, Sweden

Ström, S. et al., Hum Reprod 22, 3051-3058 (2007)

HS422

Provider: Karolinska Institut, Stockholm, Sweden

Ström, S. et al., Hum Reprod 22, 3051-3058 (2007)

HS426

Provider: Karolinska Institut, Stockholm, Sweden

Ström, S. et al., Hum Reprod 22, 3051-3058 (2007)

HS429

Provider: Karolinska Institut, Stockholm, Sweden

Ström, S. et al., Hum Reprod 22, 3051-3058 (2007)

HSF-1 (UC01)

Provider: University of California, San Francisco, CA, USA

Abeyta, M. J. et al., Hum Mol Genet 13, 601-608 (2004)

Clark, A. T. et al., Hum Mol Genet 13, 727-739 (2004)

Clark, A. T. et al., Stem Cells 22, 169-179 (2004)

Sun, B. W. et al., Hum Mol Genet 15, 65-75 (2006)

Adewumi, O. et al., Nat Biotechnol 25, 803-816 (2007)

Saxe, J. P. et al., Chem Biol 14, 1019-1030 (2007)

Wu, H. et al., Proc Natl Acad Sci U S A 104, 13821-13826 (2007)

Fuentealba, L. C. et al., Proc Natl Acad Sci U S A 105, 7732-7737 (2008)

Lowry, W. E. et al., Proc Natl Acad Sci U S A 105, 2883-2888 (2008)

McElroy, S. L. et al., Reprod Biomed Online 16, 684-693 (2008)

Valamehr, B. et al., Proc Natl Acad Sci U S A 105, 14459-14464 (2008)

Wu, H. et al., Stem Cells 26, 1484-1489 (2008)

HSF-10

Provider: University of California, San Francisco, CA, USA

Chavez, S. L. et al., Stem Cells Dev 17, 535-546 (2008)

HSF-12

Provider: University of California, San Francisco, CA, USA

Chavez, S. L. et al., Stem Cells Dev 17, 535-546 (2008)

HSF-13

Provider: University of California, San Francisco, CA, USA

Chavez, S. L. et al., Stem Cells Dev 17, 535-546 (2008)

HSF-6 (UC06)

Provider: University of California, San Francisco, CA, USA

Abeyta, M. J. et al., Hum Mol Genet 13, 601-608 (2004)

Clark, A. T. et al., Hum Mol Genet 13, 727-739 (2004)

Clark, A. T. et al., Stem Cells 22, 169-179 (2004)

Daheron, L. et al., Stem Cells 22, 770-778 (2004)

Humphrey, R. K. et al., Stem Cells 22, 522-530 (2004)

Moore, F. L. et al., Genomics 83, 834-843 (2004)

Vallier, L. et al., Dev Biol 275, 403-421 (2004)

Beattie, G. M. et al., Stem Cells 23, 489-495 (2005)

Cai, J. et al., BMC Dev Biol 5, 26 (2005)

Ezeh, U. I., et al., Cancer,104, 2255-2265 (2005)

Park, C. H. et al., J Neurochem 92, 1265-1276 (2005)

Rugg-Gunn, P. J.et al., Nat Genet 37, 585-587 (2005)

Son, Y. S. et al., Stem Cells 23, 1502-1513 (2005)

St John, J. C. et al., Cloning Stem Cells 7, 141-153 (2005)

Yoo, S. J. et al., Exp Mol Med 37, 399-407 (2005)

Androutsellis-Theotokis, A. et al., Nature 442, 823-826 (2006)

Chang, K. H. et al., Blood 108, 1515-1523 (2006)

Clements, M. O. et al., Tissue Eng 12, 1741-1751 (2006)

Constantinescu, D., et al., Stem Cells 24, 177-185 (2006)

Gaur, M. et al., J Thromb Haemost 4, 436-442 (2006)

Lamba, D. A. et al., Proc Natl Acad Sci U S A 103, 12769-12774 (2006)

Li, Y.J., et al., J Biomed Mater Res A 79, 1-5 (2006)

Poon, E. et al., J Cell Sci 119, 759-768 (2006)

Postovit, L. M. et al., Stem Cells 24, 501-505 (2006)

Shen, Y. et al., Hum Mol Genet 15, 2623-2635 (2006)

Sun, B. W. et al., Hum Mol Genet 15, 65-75 (2006)

Tuve, S. et al., J Virol 80, 12109-12120 (2006)

Ware, C. B. et al, Stem Cells 24, 2677-2684 (2006)
Yao, S. et al., Proc Natl Acad Sci U S A 103, 6907-6912 (2006)
Yoon, B. S. et al., Differentiation 74, 149-159 (2006)
Adewumi, O. et al., Nat Biotechnol 25, 803-816 (2007)
Brons, I. G. et al., Nature 448, 191-195 (2007)
Duan, Y. et al., Stem Cells 25, 3058-3068 (2007)
Garcia-Perez, J. L. et al.,Hum Mol Genet,16, 1569-1577 (2007)
Gharwan, H. et al., Mol Ther 15, 1827-1833 (2007)
Hirst, M. et al., Genome Biol 8, R113 (2007)
Kim, S. E. et al., Mol Cells 23, 49-56 (2007)
Ko, J. Y. et al., J Neurochem 103, 1417-1429 (2007)
Ozolek, J. A. et al., Stem Cells Dev 16, 134-144 (2007)
Rodriguez, R. T. et al., Exp Biol Med (Maywood) 232, 1368-1380 (2007)
Saxe, J. P. et al., Chem Biol 14, 1019-1030 (2007)
Shih, C. C. et al., Stem Cells Dev 16, 893-902 (2007)
Son, Y. S. & Hong J., J Microbiol 45, 547-552 (2007)
Vallier, L. et al., Stem Cell ,25, 1490-1497 (2007)
Wu, H. et al., Proc Natl Acad Sci U S A 104, 13821-13826 (2007)
Bera, T. K. et al., Stem Cells Dev 17, 325-332 (2008)
Bonig, H. et al., Transfusion 48, 1039-1040 (2008)
Chan, E. M. et al., Cloning Stem Cells 10, 107-118 (2008)
Chang, K. H. et al., Exp Cell Res 314, 2930-2940 (2008)
Hall, L. L. et al., J Cell Physiol 216, 445-452 (2008)
Hong, S. et al., J Neurochem 104, 316-324 (2008)
King, C. C. et al., Regen Med 3, 175-180 (2008)
Muller, F. J. et al., Nature 455, 401-405 (2008)
Shen, Y. et al., Proc Natl Acad Sci U S A 105, 4709-4714 (2008)
Smith, J. R. et al., Dev Biol 313, 107-117 (2008)
Smith, J. R. et al., Stem Cells 26, 496-504 (2008)
Wu, H. et al., Stem Cells 26, 1484-1489 (2008)

HSF-7

Provider: University of California, San Francisco, CA, USA

Chavez, S. L. et al., Stem Cells Dev 17, 535-546 (2008)

HSF-8

Provider: University of California, San Francisco, CA, USA

Chavez, S. L. et al., Stem Cells Dev 17, 535-546 (2008)

HSF-9

Provider: University of California, San Francisco, CA, USA

Chavez, S. L. et al., Stem Cells Dev 17, 535-546 (2008)

HUES1

Provider: Harvard University, Cambridge, MA, USA

Cowan, C. A. et al., N Engl J Med 350, 1353-1356 (2004)

Pomp, O. et al., Stem Cells 23, 923-930 (2005)

Lund, R. D. et al., Cloning Stem Cells 8, 189-199 (2006)

Van Hoof, D. et al., Mol Cell Proteomics 5, 1261-1273 (2006)

Adewumi, O. et al., Nat Biotechnol 25, 803-816 (2007)

Assou ,S. et al., Stem Cells, 25, 961-973 (2007)

Brown, B. D. et al., Nat Biotechnol 25, 1457-1467 (2007)

Kim, K. P. et al., Genome Res 17, 1731-1742 (2007)

Lombardo, A. et al., Nat Biotechnol 25, 1298-1306 (2007)

Shi, F. et al., Eur J Neurosci 26, 3016-3023 (2007)

Tomescot, A. et al., Stem Cells 25, 2200-2205 (2007)

Andersson, M. K. et al., BMC Cell Biol 9, 37 (2008)

Apati, A. et al., Biochim Biophys Acta 1778, 2700-2709 (2008)

Atlasi, Y. et al., Stem Cells 26, 3068-3074 (2008)

Bartova, E. et al., Dev Dyn 237, 3690-3702 (2008)

Braam, S. R. et al., Nat Methods 5, 389-392 (2008)

Braam, S. R. et al., Stem Cells 26, 2257-2265 (2008)

Di Giorgio, F. P. et al., Cell Stem Cell 3, 637-648 (2008)

Furue, M. K. et al., Proc Natl Acad Sci U S A 105, 13409-13414 (2008)

Osafune, K. et al., Nat Biotechnol 26, 313-315 (2008)

Saxena, S. et al., Mol Reprod Dev 75, 1523-1532 (2008)

Silva, S. S. et al., Proc Natl Acad Sci U S A 105, 4820-4825 (2008)

Zhou, Y. et al., Biochem Biophys Res Commun 376, 542-547 (2008)

HUES10

Provider: Harvard University, Cambridge, MA, USA

Cowan, C. A. et al., N Engl J Med 350, 1353-1356 (2004)

Lund, R. D. et al., Cloning Stem Cells 8, 189-199 (2006)

Adewumi, O. et al., Nat Biotechnol 25, 803-816 (2007)

Baker, D. E. et al., Nat Biotechnol 25, 207-215 (2007)

Kim, K. P. et al., Genome Res 17, 1731-1742 (2007)

Dimos, J. T. et al., Science 321, 1218-1221 (2008)

Osafune, K. et al., Nat Biotechnol 26, 313-315 (2008)

HUES11

Provider: Harvard University, Cambridge, MA, USA

Cowan, C. A. et al., N Engl J Med 350, 1353-1356 (2004)

Adewumi, O. et al., Nat Biotechnol 25, 803-816 (2007)

Osafune, K. et al., Nat Biotechnol 26, 313-315 (2008)

HUES12

Provider: Harvard University, Cambridge, MA, USA

Cowan, C. A. et al., N Engl J Med 350, 1353-1356 (2004)

Adewumi, O. et al., Nat Biotechnol 25, 803-816 (2007)

Blum, B. & Benvenisty N., Stem Cells 25, 1924-1930 (2007)

Kim, K. P. et al., Genome Res 17, 1731-1742 (2007)

Di Giorgio, F. P. et al., Cell Stem Cell 3, 637-648 (2008)

Osafune, K. et al., Nat Biotechnol 26, 313-315 (2008)

Silva, S. S. et al., Proc Natl Acad Sci U S A 105, 4820-4825 (2008)

HUES13

Provider: Harvard University, Cambridge, MA, USA

Cowan, C. A. et al., N Engl J Med 350, 1353-1356 (2004)

Adewumi, O. et al., Nat Biotechnol 25, 803-816 (2007)

Baker, D. E. et al., Nat Biotechnol 25, 207-215 (2007)

Blum, B. & Benvenisty N., Stem Cells 25, 1924-1930 (2007)

Kim, K. P. et al., Genome Res 17, 1731-1742 (2007)

Di Giorgio, F. P. et al., Cell Stem Cell 3, 637-648 (2008)

Maurer, J. et al., PLoS ONE 3, e3451 (2008)

Muller, F. J. et al., Nature 455, 401-405 (2008)

Osafune, K. et al., Nat Biotechnol 26, 313-315 (2008)

HUES14

Provider: Harvard University, Cambridge, MA, USA

Cowan, C. A. et al., N Engl J Med 350, 1353-1356 (2004)

Adewumi, O. et al., Nat Biotechnol 25, 803-816 (2007)

Baker, D. E. et al., Nat Biotechnol 25, 207-215 (2007)

Kim, K. P. et al., Genome Res 17, 1731-1742 (2007)

Osafune, K. et al., Nat Biotechnol 26, 313-315 (2008)

Silva, S. S. et al., Proc Natl Acad Sci U S A 105, 4820-4825 (2008)

HUES15

Provider: Harvard University, Cambridge, MA, USA

Cowan, C. A. et al., N Engl J Med 350, 1353-1356 (2004)

Adewumi, O. et al., Nat Biotechnol 25, 803-816 (2007)

Kim, K. P. et al., Genome Res 17, 1731-1742 (2007)

Braam, S. R. et al., Nat Methods 5, 389-392 (2008)

Osafune, K. et al., Nat Biotechnol 26, 313-315 (2008)

Silva, S. S. et al., Proc Natl Acad Sci U S A 105, 4820-4825 (2008)

HUES16

Provider: Harvard University, Cambridge, MA, USA

Cowan, C. A. et al., N Engl J Med 350, 1353-1356 (2004)

Adewumi, O. et al., Nat Biotechnol 25, 803-816 (2007)

Kim, K. P. et al., Genome Res 17, 1731-1742 (2007)

Osafune, K. et al., Nat Biotechnol 26, 313-315 (2008)

HUES17

Provider: Harvard University, Cambridge, MA, USA

Cowan, C. A. et al., N Engl J Med 350, 1353-1356 (2004)

Adewumi, O. et al., Nat Biotechnol 25, 803-816 (2007)

Baker, D. E. et al., Nat Biotechnol 25, 207-215 (2007)

Kim, K. P. et al., Genome Res 17, 1731-1742 (2007)

Shi, F. et al., Eur J Neurosci 26, 3016-3023 (2007)

Liao, J. et al., Cell Res 18, 600-603 (2008)

Osafune, K. et al., Nat Biotechnol 26, 313-315 (2008)

Wu, Z. et al., J Biol Chem 283, 24991-25002 (2008)

HUES2

Provider: Harvard University, Cambridge, MA, USA

Cowan, C. A. et al., N Engl J Med 350, 1353-1356 (2004)

Lund, R. D. et al., Cloning Stem Cells 8, 189-199 (2006)

Adewumi, O. et al., Nat Biotechnol 25, 803-816 (2007)

Kim, K. P. et al., Genome Res 17, 1731-1742 (2007)

Huangfu, D. et al., Nat Biotechnol 26, 1269-1275 (2008)

Karlsson, K. R. et al., Exp Hematol 36, 1167-1175 (2008)

Osafune, K. et al., Nat Biotechnol 26, 313-315 (2008)

HUES20

Provider: Harvard University, Cambridge, MA, USA

Josephson, R. et al., Stem Cells 25, 437-446 (2007)

Muller, F. J. et al., Nature 455, 401-405 (2008)

HUES21

Provider: Harvard University, Cambridge, MA, USA

Josephson, R. et al., Stem Cells 25, 437-446 (2007)

Muller, F. J. et al., Nature 455, 401-405 (2008)

HUES22

Provider: Harvard University, Cambridge, MA, USA

Josephson, R. et al., Stem Cells 25, 437-446 (2007)

Muller, F. J. et al., Nature 455, 401-405 (2008)

HUES3

Provider: Harvard University, Cambridge, MA, USA

Cowan, C. A. et al., N Engl J Med 350, 1353-1356 (2004)

Huang, H. et al., Biochem Biophys Res Commun 351, 321-327 (2006)

Lund, R. D. et al., Cloning Stem Cells 8, 189-199 (2006)

Adewumi, O. et al., Nat Biotechnol 25, 803-816 (2007)

Assou, S. et al., Stem Cells, 25, 961-973 (2007)

Brown, B. D. et al., Nat Biotechnol 25, 1457-1467 (2007)

Huang, Y. et al., Hybridoma (Larchmt) 26, 387-391 (2007)

Kim, K. P. et al., Genome Res 17, 1731-1742 (2007)

Lombardo, A. et al., Nat Biotechnol 25, 1298-1306 (2007)

Andersson, M. K. et al., BMC Cell Biol 9, 37 (2008)

Di Giorgio, F. P. et al., Cell Stem Cell 3, 637-648 (2008)

Dimos, J. T. et al., Science 321, 1218-1221 (2008)

Lu, S. J. et al., Blood 112, 4475 (2008)

Lu, S. J. et al., Regen Med 3, 693-704 (2008)

Osafune, K. et al., Nat Biotechnol 26, 313-315 (2008)

Santoni de Sio, F. R. et al., Stem Cells 26, 2142-2152 (2008)

Saxena, S. et al., Mol Reprod Dev 75, 1523-1532 (2008)

Zhou, Y. et al., Biochem Biophys Res Commun 376, 542-547 (2008)

HUES3-HB9::GFP (derivative of HUES3)

Provider: Harvard University, Cambridge, MA, USA

Di Giorgio, F. P. et al., Cell Stem Cell 3, 637-648 (2008)

HUES4

Provider: Harvard University, Cambridge, MA, USA

Cowan, C. A. et al., N Engl J Med 350, 1353-1356 (2004)

Adewumi, O. et al., Nat Biotechnol 25, 803-816 (2007)

Kim, K. P. et al., Genome Res 17, 1731-1742 (2007)

Osafune, K. et al., Nat Biotechnol 26, 313-315 (2008)

HUES5

Provider: Harvard University, Cambridge, MA, USA

Cowan, C. A. et al., N Engl J Med 350, 1353-1356 (2004)

Lund, R. D. et al., Cloning Stem Cells 8, 189-199 (2006)

Adewumi, O. et al., Nat Biotechnol 25, 803-816 (2007)

Baker, D. E. et al., Nat Biotechnol 25, 207-215 (2007)

Kim, K. P. et al., Genome Res 17, 1731-1742 (2007)

Braam, S. R. et al., Nat Methods 5, 389-392 (2008)

Di Giorgio, F. P. et al., Cell Stem Cell 3, 637-648 (2008)

Osafune, K. et al., Nat Biotechnol 26, 313-315 (2008)

Silva, S. S. et al., Proc Natl Acad Sci U S A 105, 4820-4825 (2008)

HUES6

Provider: Harvard University, Cambridge, MA, USA

Cowan, C. A. et al., N Engl J Med 350, 1353-1356 (2004)

Chew, J. L. et al., Mol Cell Biol 25, 6031-6046 (2005)

Cowan, C. A. et al., Science 309, 369-373 (2005)

Rodda, D. J. et al., J Biol Chem 280, 24731-24737 (2005)

James, D. et al., Dev Biol 295, 90-102 (2006)

Lund, R. D. et al., Cloning Stem Cells 8, 189-199 (2006)

Zhang, J. et al., Nat Cell Biol 8, 1114-1123 (2006)

Adewumi, O. et al., Nat Biotechnol 25, 803-816 (2007)

Baker, D. E. et al., Nat Biotechnol 25, 207-215 (2007)

Bakre, M. M. et al., J Biol Chem 282, 31703-31712 (2007)

Kim, K. P. et al., Genome Res 17, 1731-1742 (2007)

Huangfu, D. et al., Nat Biotechnol 26, 1269-1275 (2008)

Osafune, K. et al., Nat Biotechnol 26, 313-315 (2008)

Silva, S. S. et al., Proc Natl Acad Sci U S A 105, 4820-4825 (2008)

HUES7

Provider: Harvard University, Cambridge, MA, USA

Cowan, C. A. et al., N Engl J Med 350, 1353-1356 (2004)

D'Amour, K. A. et al., Nat Biotechnol 23, 1534-1541(2005)

Goh, G. et al., Thromb Haemost 94, 728-737 (2005)

Pomp, O. et al., Stem Cells 23, 923-930 (2005)

Bibikova, M. et al., Genome Res 16, 1075-1083 (2006)

Denning, C. et al., Int J Dev Biol 50, 27-37 (2006)

Josephson, R. et al., BMC Biol 4, 28 (2006)

Lund, R. D. et al., Cloning Stem Cells 8, 189-199 (2006)

Sun, B. W. et al., Hum Mol Genet 15, 65-75 (2006)

Adewumi, O. et al., Nat Biotechnol 25, 803-816 (2007)

Allegrucci, C. et al., Hum Mol Genet, 16, 1253-1268 (2007)

Anderson, D. et al., Mol Ther, 15, 2027-2036 (2007)

Baker, D. E. et al., Nat Biotechnol 25, 207-215 (2007)

Bakre, M. M. et al., J Biol Chem 282, 31703-31712 (2007)

Burridge, P. W. et al., Stem Cells, 25, 929-938 (2007)

Iacovitti, L. et al., Brain Res 1127,19-25 (2007)

Kim, K. P. et al., Genome Res 17, 1731-1742 (2007)

Kumar, M. et al., Stem Cells Dev 16, 667-681 (2007)

Li, O. et al., FEBS Lett 581, 3533-3537 (2007)

Phillips, B. W. et al., Stem Cells Dev 16, 561-578 (2007)

Shi, F. et al., Eur J Neurosci 26, 3016-3023 (2007)

Braam, S. R. et al., Nat Methods 5, 389-392 (2008)

Brokhman, I. et al., Differentiation 76, 145-155 (2008)

Dormeyer, W. et al., J Proteome Res 7, 2936-2951 (2008)

Gallo, P. et al., Gene Ther 15, 161-170 (2008)

Garcia-Gonzalo, F. R. et al., PLoS ONE 3, e1384 (2008)

Li, X. et al., J Genet Genomics 35, 723-728 (2008)

Maurer, J. et al., PLoS ONE 3, e3451 (2008)

Mayshar, Y. et al., Stem Cells 26, 767-747 (2008)

Muller, F. J. et al., Nature 455, 401-405 (2008)

Osafune, K. et al., Nat Biotechnol 26, 313-315 (2008)

Pomp, O. et al., Brain Res 1230, 50-60 (2008)

Saxena, S. et al., Mol Reprod Dev 75, 1523-1532 (2008)

Van Hoof, D. et al., Stem Cells 26, 2777-2781 (2008)

HUES8

Provider: Harvard University, Cambridge, MA, USA

Cowan, C. A. et al., N Engl J Med 350, 1353-1356 (2004)

Lund, R. D. et al., Cloning Stem Cells 8, 189-199 (2006)

Adewumi, O. et al., Nat Biotechnol 25, 803-816 (2007)

Bakre, M. M. et al., J Biol Chem 282, 31703-31712 (2007)

Iacovitti, L. et al., Brain Res 1127, 19-25 (2007)

Kim, K. P. et al., Genome Res 17, 1731-1742 (2007)

Li, O. et al., FEBS Lett 581, 3533-3537 (2007)

Tan, S. M. et al., Nucleic Acids Res 35, e118 (2007)

Gallo, P. et al., Gene Ther 15, 161-170 (2008)

Huangfu, D. et al., Nat Biotechnol 26, 1269-1275 (2008)

Maherali, N. et al., Cell Stem Cell 3, 340-345 (2008)

Osafune, K. et al., Nat Biotechnol 26, 313-315 (2008)

HUES9

Provider: Harvard University, Cambridge, MA, USA

Cowan, C. A. et al., N Engl J Med 350, 1353-1356 (2004)

Joannides, A. et al., Stem Cells 24, 230-235 (2006)

Adewumi, O. et al., Nat Biotechnol 25, 803-816 (2007)

Bakre, M. M. et al., J Biol Chem 282, 31703-31712 (2007)

Frandsen, U. et al., Biochem Biophys Res Commun, 362, 568-574 (2007)

Inanc, B. et al., Artif Organs 31, 792-800 (2007)

Joannides, A. J. et al., Brain 130, 1263-1275 (2007)

Joannides, A. J. et al., Stem Cells 25, 731-737 (2007)

Josephson, R. et al., Stem Cells 25, 437-446 (2007)

Kim, K. P. et al., Genome Res 17, 1731-1742 (2007)

Lian, Q. et al., Stem Cells 25, 425-436 (2007)

Lim, L. S. et al., Mol Biol Cell 18, 1348-58 (2007)

Phillips, B. W. et al., Stem Cells Dev 16, 561-578 (2007)

Siva, K. et al., PLoS ONE 2, e1202 (2007)

Sze, S. K. et al., Mol Cell Proteomics 6, 1680-1689 (2007)

Tan, S. M. et al., Nucleic Acids Res 35, e118 (2007)

Apati, A. et al., Biochim Biophys Acta 1778, 2700-2709 (2008)

Bartova, E. et al., Dev Dyn 237, 3690-3702 (2008)

Bartova, E. et al., Differentiation 76, 24-32 (2008)

Di Giorgio, F. P. et al., Cell Stem Cell 3, 637-648 (2008)

Dimos, J. T. et al., Science 321, 1218-1221 (2008)

Garcia-Gonzalo, F. R. et al., PLoS ONE 3, e1384 (2008)

Gjerstorff, M. F. et al., Hum Reprod 23, 2194-2201 (2008)

Harkness, L. et al., Stem Cell Res 1, 219-227 (2008)

Hwang, N. S. et al., Proc Natl Acad Sci U S A 105, 20641-20646 (2008)

Inanc, B. et al., Artif Organs 32, 100-109 (2008)

Inanc, B. et al., Tissue Eng Part A 14, 955-964 (2008)

Marchetto, M. C. et al., Cell Stem Cell 3, 649-657 (2008)

Mayshar, Y. et al., Stem Cells 26, 767-747 (2008)

Muller, F. J. et al., Nature 455, 401-405 (2008)

Osafune, K. et al., Nat Biotechnol 26, 313-315 (2008)

Pomp, O. et al., Brain Res 1230, 50-60 (Sep 16, 2008)

Richards, S. et al., Tissue Eng Part C Methods 14, 221 (2008)

Saxena, S. et al., Mol Reprod Dev 75, 1523-1532 (2008)

Silva, S. S. et al., Proc Natl Acad Sci U S A 105, 4820-4825 (2008)

Sivasubramaniyan, K. et al., Regen Med 3, 23-31 (2008)

HUES9 (Derivatives)

Come, J. et al., Tissue Eng Part C Methods 14, 289-298 (2008)

I3 (TE03)

Provider: Technion-Israel Institute of Technology, Rambam Medical Center, Haifa, Israel

Amit, M. & Itskovitz-Eldor, J., J Anat 200, 225-232 (2002)

Amit, M. et al., Biol Reprod 68, 2150-2156 (2003)

Amit, M. et al., Biol Reprod 70, 837-845 (2004)

Amit, M. et al., Stem Cells 23, 761-771 (2005)

Dolnikov, K. et al., Stem Cells 24, 236-245 (2006)

Josephson, R. et al., BMC Biol 4, 28 (2006)

Mallon, B. S. et al., Int J Biochem Cell Biol 38, 1063-1075 (2006)

Adewumi, O. et al., Nat Biotechnol 25, 803-816 (2007)

Izrael, M. et al., Mol Cell Neurosci 34, 310-323 (2007)

Terstegge, S. et al., Biotechnol Bioeng 96, 195-201 (2007)

Aberdam, E. et al., Stem Cells 26, 440-444 (2008)
Calvanese, V. et al., PLoS ONE 3, e3294 (2008)
Hall, L. L. et al., J Cell Physiol 216, 445-452 (2008)
Ma, W. et al., BMC Dev Biol 8, 90 (2008)
Mayshar, Y. et al., Stem Cells 26, 767-747 (2008)

I4 (TE04)

Provider: Technion-Israel Institute of Technology, Rambam Medical Center, Haifa, Israel

Amit, M. & Itskovitz-Eldor, J., J Anat 200, 225-232 (2002)
Amit, M. et al., Stem Cells 23, 761-771 (2005)
Bibikova, M. et al., Genome Res 16, 1075-1083 (2006)
Josephson, R. et al., BMC Biol 4, 28 (2006)
Hall, L. L. et al., J Cell Physiol 216, 445-452 (2008)

I6 (TE06)

Provider: Technion-Israel Institute of Technology, Rambam Medical Center, Haifa, Israel

Amit, M. & Itskovitz-Eldor, J., J Anat 200, 225-232 (2002)
Amit, M. et al., Biol Reprod 68, 2150-2156 (2003)
Gerecht-Nir, S. et al., Lab Invest 83, 1811-1820 (2003)
Amit, M. et al., Biol Reprod 70, 837-845 (2004)
Bhattacharya, B. et al., Blood 103, 2956-2964 (2004)
Dang, S. M. et al., Stem Cells 22, 275-282 (2004)
Gerecht-Nir, S. et al., Biol Reprod 71, 2029-2036 (2004)
Ginis, I. et al., Dev Biol 269, 360-380 (2004)
Segev, H. et al., Stem Cells 22, 265-274 (2004)
Amit, M. et al., Stem Cells 23, 761-771 (2005)
Bibikova, M. et al., Genome Res 16, 1075-1083 (2006)
Cai, J. et al., Stem Cells 24, 516-530 (2006)
Josephson, R. et al., BMC Biol 4, 28 (2006)
Li, H. et al., BMC Genomics 7, 103 (2006)
Liu, Y. et al., BMC Dev Biol 6, 20 (2006)
Player, A. et al., Stem Cells Dev 15, 315-323 (2006)
Xiao, L. et al., Stem Cells 24, 1476-1486 (2006)
Zeng, X. et al., Neuropsychopharmacology 31, 2708-2715 (2006)
Adewumi, O. et al., Nat Biotechnol 25, 803-816 (2007)
Cai L. et al., Cell Res 17, 62-72 (2007)
Izrael, M. et al., Mol Cell Neurosci 34, 310-323 (2007)
Leor, J. et al., Heart 93, 1278-1284 (2007)
Peerani, R. et al., Embo J 26, 4744-4755 (2007)
Shin, S. et al., Stem Cells 25, 1298-1306 (2007)
Tomescot, A. et al., Stem Cells 25, 2200-2205 (2007)
Zhou, B. Y. et al., Stem Cells 25, 779-789 (2007)
Aberdam, E. et al., Stem Cells 26, 440-444 (2008)

Ma, W. et al., BMC Dev Biol 8, 90 (2008)

Maynard, S. et al., Stem Cells 26, 2266-2674 (2008)

Mayshar, Y. et al., Stem Cells 26, 767-747 (2008)

Muller, F. J. et al., Nature 455, 401-405 (2008)

Ungrin, M. D. et al., PLoS ONE 3, e1565 (2008)

I8

Provider: Technion-Israel Institute of Technology, Rambam Medical Center, Haifa, Israel

Amit, M. et al., Stem Cells 23, 761-771 (2005)

Lee, G. et al., Nat Biotechnol 25, 1468-1475 (2007)

I9

Provider: Technion-Israel Institute of Technology, Rambam Medical Center, Haifa, Israel

Gerecht-Nir, S. et al., Lab Invest 83, 1811-1820 (2003)

Suss-Toby, E. et al., Hum Reprod 19, 670-675 (2004)

J3

Provider: Technion-Israel Institute of Technology, Rambam Medical Center, Haifa. Israel

Muller, F. J. et al., Nature 455, 401-405 (2008)

KCL-001 (formerly WT3)

Provider: King's College London, UK

Pickering, S. J. et al., Reprod Biomed Online 7, 353-364 (2003)

Karlsson, K. R. et al., Exp Hematol 36, 1167-1175 (2008)

KCL-002 (formerly WT4)

Provider: King's College London, UK

Karlsson, K. R. et al., Exp Hematol 36, 1167-1175 (2008)

KCL-003-CF1 (formerly CF-1)

Provider: King's College London, UK

Pickering, S. J. et al., Reprod Biomed Online 10, 390-397 (2005)

Adewumi, O. et al., Nat Biotechnol 25, 803-816 (2007)

KhES-1

Provider: Institute for Frontier Medical Sciences, Kyoto University, Japan

Fujioka, T. et al., Int J Dev Biol 48, 1149-1154 (2004)

Hatano, S. Y. et al., Mech Dev 122, 67-79 (2005)

Kuroda, T. et al., Mol Cell Biol 25, 2475-2485 (2005)

Chen, Y. et al., Cell Transplant 15, 865-871 (2006)

Hasegawa, K. et al., Stem Cells 24, 2649-2660 (2006)

Soto-Gutierrez, A. et al., Cell Transplant 15, 335-341 (2006)

Suemori, H. et al., Biochem Biophys Res Commun 345, 926-932 (2006)

Ueno, M. et al., Proc Natl Acad Sci U S A 103, 9554-9559 (2006)

Adewumi, O. et al., Nat Biotechnol 25, 803-816 (2007)

Hasegawa, K. et al., Stem Cells 25, 1707-1712 (2007)

Senju, S. et al., Stem Cells 25, 2720-2729 (2007)

Sone, M. et al., Arterioscler Thromb Vasc Biol 27, 2127-2134 (2007)

Watanabe, K. et al., Nat Biotechnol 25, 681-686 (2007)

Ishii, T. et al., Am J Physiol Gastrointest Liver Physiol 295, G313 (2008)

Miyazaki, T. et al., Biochem Biophys Res Commun 375, 27-32 (2008)

Navarro-Alvarez, N. et al., Cell Transplant 17, 27-33 (2008)

Osakada, F. et al., Nat Biotechnol 26, 215-224 (2008)

Shiraki, N. et al., Genes Cells 13, 731-746 (2008)

Sumi, T. et al., Development 135, 2969-2679 (2008)

Takayama, N. et al., Blood 111, 5298-5306 (2008)

Tsuneyoshi, N. et al., Biochem Biophys Res Commun 367, 899-905 (2008)

KhES-1 (subline 1)

Provider: Institute for Frontier Medical Sciences, Kyoto University, Japan

Suzuki, K. et al., Proc Natl Acad Sci U S A 105, 13781-13786 (2008)

KhES-2

Provider: Institute for Frontier Medical Sciences, Kyoto University, Japan

Hasegawa, K. et al., Stem Cells 24, 2649-2660 (2006)

Suemori, H. et al., Biochem Biophys Res Commun 345, 926-932 (2006)

Adewumi, O. et al., Nat Biotechnol 25, 803-816 (2007)

Hasegawa, K. et al., Stem Cells 25, 1707-1712 (2007)

Sumi, T. et al., Oncogene 26, 5564-5576 (2007)

Watanabe, K. et al., Nat Biotechnol 25, 681-686 (2007)

Hayashi, H. et al., Eur J Neurosci 27, 261-268 (2008)

Ishii, T. et al., Am J Physiol Gastrointest Liver Physiol 295, G313 (2008)

Miyazaki, T. et al., Biochem Biophys Res Commun 375, 27-32 (2008)

Takayama, N. et al., Blood 111, 5298-5306 (2008)

Tsuneyoshi, N. et al., Biochem Biophys Res Commun 367, 899-905 (2008)

KhES-3

Provider: Institute for Frontier Medical Sciences, Kyoto University, Japan

Hasegawa, K. et al., Stem Cells 24, 2649-2660 (2006)

Suemori, H. et al., Biochem Biophys Res Commun 345, 926-932 (2006)

Adewumi, O. et al., Nat Biotechnol 25, 803-816 (2007)

Hasegawa, K. et al., Stem Cells 25, 1707-1712 (2007)

Senju, S. et al., Stem Cells 25, 2720-2729 (2007)

Sumi, T. et al., Oncogene 26, 5564-5576 (2007)

Watanabe, K. et al., Nat Biotechnol 25, 681-686 (2007)

Ishii, T. et al., Am J Physiol Gastrointest Liver Physiol 295, G313 (2008)

Miyazaki, T. et al., Biochem Biophys Res Commun 375, 27-32 (2008)

Osakada, F. et al., Nat Biotechnol 26, 215-224 (2008)

Sumi, T. et al., Development 135, 2969-2679 (2008)

Suzuki, K. et al., Proc Natl Acad Sci U S A 105, 13781-13786 (2008)

Takayama, N. et al., Blood 111, 5298-5306 (2008)

Tsuneyoshi, N. et al., Biochem Biophys Res Commun 367, 899-905 (2008)

KMEB1

Provider: University of Southern Denmark, Odense, Denmark

Gjerstorff, M. F. et al., Hum Reprod 23, 2194-2201 (2008)

KMEB2

Provider: University of Southern Denmark, Odense, Denmark

Gjerstorff, M. F. et al., Hum Reprod 23, 2194-2201 (2008)

KMEB3

Provider: University of Southern Denmark, Odense, Denmark

Gjerstorff, M. F. et al., Hum Reprod 23, 2194-2201 (2008)

KMEB4

Provider: University of Southern Denmark, Odense, Denmark

Gjerstorff, M. F. et al., Hum Reprod 23, 2194-2201 (2008)

KMEB5

Provider: University of Southern Denmark, Odense, Denmark

Gjerstorff, M. F. et al., Hum Reprod 23, 2194-2201 (2008)

LRB01

Provider: University of Copenhagen, Denmark

Laursen, S. B. et al., Reprod Biomed Online,15, 89-98 (2007)

LRB02

Provider: University of Copenhagen, Denmark

Laursen, S. B. et al., Reprod Biomed Online,15, 89-98 (2007)

LRB03

Provider: University of Copenhagen, Denmark

Laursen, S. B. et al., Reprod Biomed Online,15, 89-98 (2007)

Kiprilov, E. N. et al., J Cell Biol 180, 897-904 (2008)

LRB04

Provider: University of Copenhagen, Denmark

Laursen, S. B. et al., Reprod Biomed Online 15, 89-98 (2007)

MA01

Provider: Advanced Cell Technology Inc., Santa Monica, CA, USA

Klimanskaya, I. et al., Nature 444, 481-85 (2006)

Lund, R. D. et al., Cloning Stem Cells 8, 189-199 (2006)

Lu, S. J. et al., Nat Methods 4, 501-509 (2007)

Lu, S. J. et al., Blood 112, 4475 (2008)

Lu, S. J. et al., Regen Med 3, 693-704 (2008)

MA03

Provider: Advanced Cell Technology Inc., Santa Monica, CA, USA

Lund, R. D. et al., Cloning Stem Cells 8, 189-199 (2006)

Lu, S. J. et al., Nat Methods 4, 501-509 (2007)

Lu, S. J. et al., Stem Cells Dev 16, 547-559 (2007)

West, M. D. et al., Regen Med 3, 287-308 (2008)

MA04

Provider: Advanced Cell Technology Inc., Santa Monica, CA, USA

Lund, R. D. et al., Cloning Stem Cells 8, 189-199 (2006)

MA09

Provider: Advanced Cell Technology Inc., Santa Monica, CA, USA

Klimanskaya, I. et al., Nature 444, 481-485 (2006)

Lund, R. D. et al., Cloning Stem Cells 8, 189-199 (2006)

Lu, S. J. et al., Nat Methods 4, 501-509 (2007)

Lu, S. J. et al., Blood 112, 4475-4484 (2008)

MA133

Provider: Advanced Cell Technology Inc., Santa Monica, CA, USA

Lu, S. J. et al., Blood 112, 4475-4484 (2008)

MA14 (formerly ACT-14)

Provider: Advanced Cell Technology Inc., Santa Monica, CA, USA

Klimanskaya, I. et al., Lancet 365, 1636-1641 (2005)

Lund, R. D. et al., Cloning Stem Cells 8, 189-199 (2006)

MA40

Provider: Advanced Cell Technology Inc., Santa Monica, CA, USA

Lund, R. D. et al., Cloning Stem Cells 8, 189-199 (2006)

Lu, S. J. et al., Nat Methods 4, 501-509 (2007)

MA99

Provider: Advanced Cell Technology Inc., Santa Monica, CA, USA

Lu, S. J. et al., Blood 112, 4475-4484 (2008)

MAJ1

Provider: Advanced Cell Technology Inc., Santa Monica, CA, USA

Lund, R. D. et al., Cloning Stem Cells 8, 189-199 (2006)

MB01

Provider: Maria Biotech Co. Ltd., Maria Infertility Hospital Medical Institute, Seoul, Korea

Park, S. P. et al., Hum Reprod 19, 676-684 (2004)

Kim, D. Y. et al., Neurosci Res 58, 164-175 (2007)

MB02

Provider: Maria Biotech Co. Ltd., Maria Infertility Hospital Medical Institute, Seoul, Korea

Park, S. P. et al., Hum Reprod 19, 676-684 (2004)

MB03

Provider: Maria Biotech Co. Ltd., Maria Infertility Hospital Medical Institute, Seoul, Korea

Park, S. et al., Neurosci Lett 353, 91-94 (2003)

Park, S. P. et al., Hum Reprod 19, 676-684 (2004)

Park, S. et al., Neurosci Lett 359, 99-103 (2004)

Lee, D. H. et al., J Neurosurg 105, 127-133 (2006)

MB04

Provider: Maria Biotech Co. Ltd., Maria Infertility Hospital Medical Institute, Seoul, Korea

Park, S. P. et al., Hum Reprod 19, 676-684 (2004)

MB05

Provider: Maria Biotech Co. Ltd., Maria Infertility Hospital Medical Institute, Seoul, Korea

Park, S. P. et al., Hum Reprod 19, 676-684 (2004)

MB06

Provider: Maria Biotech Co. Ltd., Maria Infertility Hospital Medical Institute, Seoul, Korea

Park, S. P. et al., Hum Reprod 19, 676-684 (2004)

MB07

Provider: Maria Biotech Co. Ltd., Maria Infertility Hospital Medical Institute, Seoul, Korea

Park, S. P. et al., Hum Reprod 19, 676-684 (2004)

MB08

Provider: Maria Biotech Co. Ltd., Maria Infertility Hospital Medical Institute, Seoul, Korea

Park, S. P. et al., Hum Reprod 19, 676-684 (2004)

MB09

Provider: Maria Biotech Co. Ltd., Maria Infertility Hospital Medical Institute, Seoul, Korea

Park, S. P. et al., Hum Reprod 19, 676-684 (2004)

MEL-1

Provider: Stem Cell Sciences Ltd and Australian Stem Cell Centre Clayton, Victoria Australia

Adewumi, O. et al., Nat Biotechnol 25, 803-816 (2007)

MEL-2

Provider: Stem Cell Sciences Ltd and Australian Stem Cell Centre Clayton, Victoria Australia

Adewumi, O. et al., Nat Biotechnol 25, 803-816 (2007)

Postovit, L. M. et al., Proc Natl Acad Sci U S A 105, 4329-4334 (2008)

MINE

Provider: Istanbul Memorial Hospital, Istanbul, Turkey, Istanbul, Turkey, Istanbul, Turkey

Findikli, N. et al., Reprod Biomed Online 10, 617-627 (2005)

Miz-hES1

Provider: MizMedi Hospital, Seoul National University, Seoul, Korea, Seoul, Korea

Park, J. H. et al., Biol Reprod 69, 2007-2014 (2003)

Hong, S. H. et al., Mol Cells 18, 320-325 (2004)

Lee, J. B. et al., Reproduction 128, 727-735 (2004)

Park, J. H. et al., Mol Cells 17, 309-315 (2004)

Suh, M. R. et al., Dev Biol 270, 488-498 (2004)

Kang, H. B., et al., Stem Cells Dev 14, 395-401 (2005)

Kim, S. J. et al., Mol Cells 19, 46-53 (2005)

Kim, S. K. et al., Stem Cells 23, 458-462 (2005)

Lee, J. B. et al., Biol Reprod 72, 42-49 (2005)

Lee, J. B. et al., Mol Cells 19, 31-38 (2005)

Park, C. H. et al., J Neurochem 92, 1265-1276 (2005)

Son, Y. S. et al., Stem Cells 23, 1502-1513 (2005)

Yoo, S. J. et al., Exp Mol Med 37, 399-407 (2005)

Lee, D. S. et al., Life Sci 80, 154-159 (2006)

Lee, J. et al., J Biol Chem 281, 33554-33565 (2006)

Rho, J. Y. et al., Hum Reprod 21, 405-412 (2006)

Ware, C. B. et al, Stem Cells 24, 2677-2684 (2006)

Mantel, C. et al., Blood 109, 4518-4527 (2007)

Shim, J. H. et al., Diabetologia 50, 1228-1238 (2007)

Son, Y. S. & Hong J., J Microbiol 45, 547-552 (2007)

Yeo, S. et al., Biochem Biophys Res Commun 359, 536-542 (2007)

Mantel, C. et al., Cell Cycle 7, 484-492 (2008)

Miz-hES10

Provider: MizMedi Hospital, Seoul National University, Seoul, Korea

Kim, S. J. et al., Mol Cells 19, 46-53 (2005)

Shim, J. H. et al., Diabetologia 50, 1228-1238 (2007)

Miz-hES11

Provider: MizMedi Hospital, Seoul National University, Seoul, Korea

Kim, S. J. et al., Mol Cells 19, 46-53 (2005)

Miz-hES12

Provider: MizMedi Hospital, Seoul National University, Seoul, Korea

Kim, S. J. et al., Mol Cells 19, 46-53 (2005)

Miz-hES13

Provider: MizMedi Hospital, Seoul National University, Seoul, Korea

Kim, S. J. et al., Mol Cells 19, 46-53 (2005)

Miz-hES14

Provider: MizMedi Hospital, Seoul National University, Seoul, Korea

Lee, J. B. et al., Biol Reprod 72, 42-49 (2005)

Miz-hES15

Provider: MizMedi Hospital, Seoul National University, Seoul, Korea

Lee, J. B. et al., Biol Reprod 72, 42-49 (2005)

Miz-hES2

Provider: MizMedi Hospital, Seoul National University, Seoul, Korea

Park, J. H. et al., Biol Reprod 69, 2007-2014 (2003)

Park, J. H. et al., Mol Cells 17, 309-315 (2004)

Yoon, B. S. et al., Differentiation 74, 149-159 (2006)

Miz-hES3

Provider: MizMedi Hospital, Seoul National University, Seoul, Korea

Park, J. H. et al., Biol Reprod 69, 2007-2014 (2003)

Park, J. H. et al., Mol Cells 17, 309-315 (2004)

Kim, S. K. et al., Stem Cells 23, 458-462 (2005)

Lee, J. B. et al., Mol Cells 19, 31-38 (2005)

Lee, J. B. et al., Biol Reprod 72, 42-49 (2005)

Kim, C. G. et al., Mol Cells 21, 343-355 (2006)

Miz-hES4

Provider: MizMedi Hospital, Seoul National University, Seoul, Korea

Kim, S. J. et al., Mol Cells 19, 46-53 (2005)

Kim, S. K. et al., Stem Cells 23, 458-462 (2005)

Lee, J. B. et al., Mol Cells 19, 31-38 (2005)

Son, Y. S. et al., Stem Cells 23, 1502-1513 (2005)

Yoo, S. J. et al., Exp Mol Med 37, 399-407 (2005)

Kim, B. K. et al., FEBS Lett 580, 5869-5874 (2006)

Kim, S. K. et al., Toxicol Sci 94, 310-321 (2006)

Kang, S. M. et al., Stem Cells 25, 419-424 (2007)

Kim, S. E. et al., Mol Cells 23, 49-56 (2007)

Shim, J. H. et al., Diabetologia 50, 1228-1238 (2007)

Muller, F. J. et al., Nature 455, 401-405 (2008)

Miz-hES5

Provider: MizMedi Hospital, Seoul National University, Seoul, Korea

Kim, S. J. et al., Mol Cells 19, 46-53 (2005)

Morris, G. J. et al., Reprod Biomed Online 13, 421-426 (2006)

Song, J. et al., Neurosci Lett 423, 58-61 (2007)

Muller, F. J. et al., Nature 455, 401-405 (2008)

Miz-hES6

Provider: MizMedi Hospital, Seoul National University, Seoul, Korea

Kim, S. J. et al., Mol Cells 19, 46-53 (2005)

Son, Y. S. et al., Stem Cells 23, 1502-1513 (2005)

Yoo, S. J. et al., Exp Mol Med 37, 399-407 (2005)

Kim, B. K. et al., FEBS Lett 580, 5869-5874 (2006)

Kim, S. K. et al., Toxicol Sci 94, 310-321 (2006)

Kim, S. E. et al., Mol Cells 23, 49-56 (2007)

Muller, F. J. et al., Nature 455, 401-405 (2008)

Miz-hES7

Provider: MizMedi Hospital, Seoul National University, Seoul, Korea

Kim, S. J. et al., Mol Cells 19, 46-53 (2005)

Miz-hES8

Provider: MizMedi Hospital, Seoul National University, Seoul, Korea

Kim, S. J. et al., Mol Cells 19, 46-53 (2005)

Miz-hES9

Provider: MizMedi Hospital, Seoul National University, Seoul, Korea

Lee, J. B. et al., Biol Reprod 72, 42-49 (2005)

---

n.n.

Provider: China Medical University, Shenyang, China

Tan, J. C. et al., Neuroreport 19, 1451-1455 (2008)

---

n.n.

Provider: National University of Singapore, Kent Ridge, Singapore

Richards, M. et al., Nat Biotechnol 20, 933 (2002)

NCL1 (formerly hES-NCL-1)

Provider: University of Newcastle and Newcastle Fertility Centre at LIFE, Newcastle. UK

Stojkovic, M. et al., Stem Cells 22, 790-797 (2004)
Hyslop, L. et al., Stem Cells 23, 1035-1043 (2005)
Stojkovic, P. et al., Stem Cells 23, 306-314 (2005)
Stojkovic, P. et al., Stem Cells 23, 895-902 (2005)
Armstrong, L. et al., Hum Mol Genet 15, 1894-1913 (2006)
Cooke, M.J.et al., Stem Cells Dev 15, 254-259 (2006)
Zhang, X. et al., Stem Cells, 24, 2669-2676 (2006)
Adewumi, O. et al., Nat Biotechnol 25, 803-816 (2007)
Ahmad, S. et al., Stem Cells 25, 1145-1155 (2007)
Allegrucci, C. et al., Hum Mol Genet, 16, 1253-1268 (2007)
Choudhary, M. et al., Stem Cells 25, 3045-3057 (2007)
Kim, K. P. et al., Genome Res 17, 1731-1742 (2007)
Ledran, M. H. et al., Cell Stem Cell 3, 85-93 (2008)
Maimets, T. et al., Oncogene 27, 5277-5287 (2008)
Saretzki, G. et al., Stem Cells 26, 455-464 (2008)
Stewart, R. et al., Regen Med 3, 505-522 (2008)
Tilgner, K. et al., Stem Cells 26, 3075-3085 (2008)
Yang, C. et al., Stem Cells 26, 850-863 (2008)

NCL2

Provider: University of Newcastle, UK

Zhang, X. et al., Stem Cells, 24, 2669-2676 (2006)

NCL3

Provider: University of Newcastle, UK

Zhang, X. et al., Stem Cells, 24, 2669-2676 (2006)

NCL4

Provider: University of Newcastle, UK

Zhang, X. et al., Stem Cells, 24, 2669-2676 (2006)

NCL5

Provider: University of Newcastle, UK

Zhang, X. et al., Stem Cells, 24, 2669-2676 (2006)

NCL6

Provider: University of Newcastle, UK

Zhang, X. et al., Stem Cells, 24, 2669-2676 (2006)

NCL7

Provider: University of Newcastle, UK

Zhang, X. et al., Stem Cells, 24, 2669-2676 (2006)

NCL8

Provider: University of Newcastle, UK

Zhang, X. et al., Stem Cells, 24, 2669-2676 (2006)

NED1 (MA126)

Provider: Advanced Cell Technology Inc., Santa Monica, CA, USA

Chung, Y. et al., Cell Stem Cell 2, 113-117 (2008)

NED2 (MA127)

Provider: Advanced Cell Technology Inc., Santa Monica, CA, USA

Chung, Y. et al., Cell Stem Cell 2, 113-117 (2008)

NED3 (MA128)

Provider: Advanced Cell Technology Inc., Santa Monica, CA, USA

Chung, Y. et al., Cell Stem Cell 2, 113-117 (2008)

NED4 (MA129)

Provider: Advanced Cell Technology Inc., Santa Monica, CA, USA

Chung, Y. et al., Cell Stem Cell 2, 113-117 (2008)

NED5

Provider: Advanced Cell Technology Inc., Santa Monica, CA, USA

Chung, Y. et al., Cell Stem Cell 2, 113-117 (2008)

NOTT1

Provider: University of Nottingham, UK

Allegrucci, C. et al., Hum Mol Genet, 16, 1253-1268 (2007)

Burridge, P. W. et al., Stem Cells, 25, 929-938 (2007)

Kim, K. P. et al., Genome Res 17, 1731-1742 (2007)

Braam, S. R. et al., Nat Methods 5, 389-392 (2008)

NOTT2

Provider: University of Nottingham, UK

Burridge, P. W. et al., Stem Cells, 25, 929-938 (2007)

Kim, K. P. et al., Genome Res 17, 1731-1742 (2007)

Braam, S. R. et al., Nat Methods 5, 389-392 (2008)

NS-3

Provider: Istanbul Memorial Hospital, Istanbul, Turkey

Findikli, N. et al., Reprod Biomed Online 10, 617-627 (2005)

NS-4

Provider: Istanbul Memorial Hospital, Istanbul, Turkey

Findikli, N. et al., Reprod Biomed Online 10, 617-627 (2005)

NS-5

Provider: Istanbul Memorial Hospital, Istanbul, Turkey

Findikli, N. et al., Reprod Biomed Online 10, 617-627 (2005)

NS-6

Provider: Istanbul Memorial Hospital, Istanbul, Turkey

Findikli, N. et al., Reprod Biomed Online 10, 617-627 (2005)

NS-7

Provider: Istanbul Memorial Hospital, Istanbul, Turkey

Findikli, N. et al., Reprod Biomed Online 10, 617-627 (2005)

NS-8

Provider: Istanbul Memorial Hospital, Istanbul, Turkey

Findikli, N. et al., Reprod Biomed Online 10, 617-627 (2005)

NTU1

Provider: Taiwan National University, Taipei, Taiwan

Chen, H. F. et al., Hum Reprod, 22, 567-577 (2007)

NTU2

Provider: Taiwan National University, Taipei, Taiwan

Chen, H. F. et al., Hum Reprod, 22, 567-577 (2007)

NTU3

Provider: Taiwan National University, Taipei, Taiwan

Chen, H. F. et al., Hum Reprod, 22, 567-577 (2007)

Odense-3 (hESC-OD3)

Provider: University of Southern Denmark, Odense, Denmark

Frandsen, U. et al., Biochem Biophys Res Commun, 362, 568-574 (2007)

Harkness, L. et al., Stem Cell Res 1, 219-227 (2008)

PKU1

Provider: Peking University Stem Cell Research Center, Beijing, China

Peng, H. & Chen, G., Hum Reprod 21, 217-22 (2006)

Song, T. et al., Mol Hum Reprod 14, 619-625 (2008)

PKU2

Provider: Peking University Stem Cell Research Center, Beijing, China

Peng, H. & Chen, G., Hum Reprod 21, 217-22 (2006)

ReliCellhES1

Provider: Reliance Life Sciences, Rabale, Navi Mumbai, India

Bibikova, M. et al., Genome Res 16, 1075-1083 (2006)

Josephson, R. et al., BMC Biol 4, 28 (2006)

Mandal, A. et al., Differentiation 74, 81-90 (2006)

Pal, R. & Khanna A., Differentiation 75, 112-122 (2007)

Pal, R. et al., Regen Med 2, 179-192 (2007)

Mehta, A. et al., Cell Biol Int 32, 1412-1424 (2008)

Ravindran, G. et al., Biochem Biophys Res Commun 373, 258-264 (2008)

RH1

Provider: Roslin Institute, UK

Fletcher, J.M. et al., Cloning Stem Cells 8, 319-334 (2006)

Forsyth, N. R. et al., Cloning Stem Cells 8, 16-23 (2006)

Forsyth, N. R. et al., Regen Med 3, 817-833 (2008)

Forsyth, N. R. et al., Rejuvenation Res 11, 5-17 (2008)

RH3

Provider: Roslin Institute, UK

Fletcher, J.M. et al., Cloning Stem Cells 8, 319-334 (2006)

RH4

Provider: Roslin Institute, UK

Fletcher, J.M. et al., Cloning Stem Cells 8, 319-334 (2006)

RH5

Provider: Roslin Institute, UK

Fletcher, J.M. et al., Cloning Stem Cells 8, 319-334 (2006)

RH6

Provider: Roslin Institute, UK

Fletcher, J.M. et al., Cloning Stem Cells 8, 319-334 (2006)

RH7

Provider: Roslin Institute, UK

Fletcher, J.M. et al., Cloning Stem Cells 8, 319-334 (2006)

Royan H1

Provider: Royan Institute, Teheran, Iran

Baharvand, H. et al., Differentiation 72, 224-229 (2004)

Baharvand, H., et al., Int J Dev Biol 50, 645-652 (2006)

Baharvand, H., et al., Dev Growth Differ 48, 323-332 (2006)

Royan H2

Provider: Royan Institute, Teheran, Iran

Baharvand, H. et al., Dev Growth Differ 48, 117-128 (2006)

Baharvand, H., et al., Proteomics 6, 3544-3549 (2006)

Royan H3

Provider: Royan Institute, Teheran, Iran

Baharvand, H. et al., Dev Growth Differ 48, 117-128 (2006)

Baharvand, H., et al., Proteomics 6, 3544-3549 (2006)

Royan H4

Provider: Royan Institute, Teheran, Iran

Baharvand, H. et al., Dev Growth Differ 48, 117-128 (2006)

Royan H5

Provider: Royan Institute, Teheran, Iran

Baharvand, H. et al., Dev Growth Differ 48, 117-128 (2006)

Baharvand, H., et al., Proteomics 6, 3544-3549 (2006)

Baharvand, H. et al., Int J Dev Biol, 51 (2007)

Baharvand, H. et al., Differentiation 76, 465-477 (2008)

Royan H6

Provider: Royan Institute, Teheran, Iran

Baharvand, H. et al., Dev Growth Differ 48, 117-128 (2006)

RUES1

Provider: Rockefeller University, New York, USA

James, D. et al., Dev Biol 295, 90-102 (2006)

RUES1-eGFP (derivative of RUES1)

Provider: Rockefeller University, New York, USA

Lee, G. et al., Nat Biotechnol 25, 1468-1475 (2007)

Lee, H. et al., Stem Cells 25, 1931-1939 (2007)

Elkabetz, Y. et al., Genes Dev 22, 152-165 (2008)

SA002.5 (subline of Sahlgrenska 002)

Provider: Cellartis AB, Göteborg, Sweden

Maitra, A. et al., Nat Genet 37, 1099-1103 (2005)

Bibikova, M. et al., Genome Res 16, 1075-1083 (2006)

Brederlau, A. et al., Stem Cells 24, 1433-1440 (2006)

Caisander, G. et al., Chromosome Res 14, 131-137 (2006)

Heins, N. et al., J Biotechnol 122, 511-520 (2006)

Josephson, R. et al., BMC Biol 4, 28 (2006)

Ek, M. et al., Biochem Pharmacol 74, 496-503 (2007)

Ellerström, C. et al., Stem Cells 25, 1690-1696 (2007)

Soderdahl, T. et al., Toxicol In Vitro 21, 929-937 (2007)

Synnergren, J. et al., Stem Cells 25, 473-480 (2007)

Adler, S. et al., Altern Lab Anim 36, 129-140 (2008)

Molne, J. et al., Transplantation 86, 1407-1413 (2008)

Synnergren, J. et al., J Biotechnol 134, 162-170 (2008)

SA046

Provider: Cellartis AB, Göteborg, Sweden

Molne, J. et al., Transplantation 86, 1407-1413 (2008)

SA121

Provider: Cellartis AB, Göteborg, Sweden

Heins, N. et al., Stem Cells 22, 367-376 (2004)

Brolen, G. K. C. et al., Diabetes 54, 2867-2874 (2005)

Darnfors, C. et al., Stem Cells 23, 483-488 (2005)

Noaksson, K. et al., Stem Cells 23, 1460-1467 (2005)

Sjögren-Jansson, E. et al., Dev Dyn 233, 1304-1314 (2005)

Caisander, G. et al., Chromosome Res 14, 131-137 (2006)

Ellerström, C. et al., Stem Cells 24, 2170-2176 (2006)

Norstrom, A. et al., Exp Biol Med (Maywood) 231, 1753-1762 (2006)

Ellerström, C. et al., Stem Cells 25, 1690-1696 (2007)

Soderdahl, T. et al., Toxicol In Vitro 21, 929-937 (2007)

Andersson, M. K. et al., BMC Cell Biol 9, 37 (2008)

SA167

Provider: Cellartis AB, Göteborg, Sweden

Sjögren-Jansson, E. et al., Dev Dyn 233, 1304-1314 (2005)

Ek, M. et al., Biochem Pharmacol 74, 496-503 (2007)

Ellerström, C. et al., Stem Cells 25, 1690-1696 (2007)

Soderdahl, T. et al., Toxicol In Vitro 21, 929-937 (2007)

Bigdeli, N. et al., J Biotechnol 133, 146-153 (2008)

Molne, J. et al., Transplantation 86, 1407-1413 (2008)

SA181

Provider: Cellartis AB, Göteborg, Sweden

Heins, N. et al., Stem Cells 22, 367-376 (2004)

Brolen, G. K. C. et al., Diabetes 54, 2867-2874 (2005)

Darnfors, C. et al., Stem Cells 23, 483-488 (2005)

Noaksson, K. et al., Stem Cells 23, 1460-1467 (2005)

SA202

Provider: Cellartis AB, Göteborg, Sweden

Noaksson, K. et al., Stem Cells 23, 1460-7 (2005)

SA240

Provider: Cellartis AB, Göteborg, Sweden

Darnfors, C. et al., Stem Cells 23, 483-488 (2005)

SA348

Provider: Cellartis AB, Göteborg, Sweden

Molne, J. et al., Transplantation 86, 1407-1413 (2008)

SA461

Provider: Cellartis AB, Göteborg, Sweden

Darnfors, C. et al., Stem Cells 23, 483-488 (2005)

Caisander, G. et al., Chromosome Res 14, 131-137 (2006)

Molne, J. et al., Transplantation 86, 1407-1413 (2008)

SA611

Provider: Cellartis AB, Göteborg, Sweden

Ellerström, C. et al., Stem Cells 24, 2170-2176 (2006)

Sahlgrenska 1 (SA001)

Provider: Cellartis AB, Göteborg, Sweden

Hansson, M. et al., Diabetes 53, 2603-2609 (2004)

Darnfors, C. et al., Stem Cells 23, 483-488 (2005)

Maitra, A. et al., Nat Genet 37, 1099-1103 (2005)

Noaksson, K. et al., Stem Cells 23, 1460-1467 (2005)

Bibikova, M. et al., Genome Res 16, 1075-1083 (2006)

Josephson, R. et al., BMC Biol 4, 28 (2006)

Adewumi, O. et al., Nat Biotechnol 25, 803-816 (2007)

Ellerström, C. et al., Stem Cells 25, 1690-1696 (2007)

Soderdahl, T. et al., Toxicol In Vitro 21, 929-937 (2007)

Synnergren, J. et al., Stem Cells 25, 473-480 (2007)

Aubry, L. et al., Proc Natl Acad Sci U S A 105, 16707-16712 (2008)

Bonnefont, J. et al., Am J Hum Genet 83, 208-218 (2008)

Come, J. et al., Tissue Eng Part C Methods 14, 289-298 (2008)

Lefort, N. et al., Nat Biotechnol 26, 1364-1366 (2008)

Molne, J. et al., Transplantation 86, 1407-1413 (2008)

Sahlgrenska 2 (SA002)

Provider: Cellartis AB, Göteborg, Sweden

Hansson, M. et al., Diabetes 53, 2603-2609 (2004)

Heins, N. et al., Stem Cells 22, 367-376 (2004)

Brolen, G. K. C. et al., Diabetes 54, 2867-2874 (2005)

Darnfors, C. et al., Stem Cells 23, 483-488 (2005)

Maitra, A. et al., Nat Genet 37, 1099-1103 (2005)

Noaksson, K. et al., Stem Cells 23, 1460-1467 (2005)

Sjögren-Jansson, E. et al., Dev Dyn 233, 1304-1314 (2005)

Bibikova, M. et al., Genome Res 16, 1075-1083 (2006)

Caisander, G. et al., Chromosome Res 14, 131-137 (2006)

Heins, N. et al., J Biotechnol 122, 511-520 (2006)

Josephson, R. et al., BMC Biol 4, 28 (2006)

Norstrom, A. et al., Exp Biol Med (Maywood) 231, 1753-1762 (2006)

Adewumi, O. et al., Nat Biotechnol 25, 803-816 (2007)

Anisimov, S. V. et al., BMC Genomics, 8, 46 (2007)

Ek, M. et al., Biochem Pharmacol 74, 496-503 (2007)

Ellerström, C. et al., Stem Cells 25, 1690-1696 (2007)

Soderdahl, T. et al., Toxicol In Vitro 21, 929-937 (2007)

Synnergren, J. et al., Stem Cells 25, 473-480 (2007)

Adler, S. et al., Altern Lab Anim 36, 129-140 (2008)

Correia, A. S. et al., Front Neurosci 2, 26 (2008)

Molne, J. et al., Transplantation 86, 1407-1413 (2008)

Synnergren, J. et al., J Biotechnol 134, 162-170 (2008)

Synnergren, J. et al., Stem Cells 26, 1831-1840 (2008)

Thyagarajan, B. et al., Stem Cells 26, 119-126 (2008)

Sahlgrenska 3

Provider: Cellartis AB, Göteborg, Sweden

Tallheden, T. et al., Life Sci 79, 999-1006 (2006)

SH1

Provider: Shanghai Second Medical University, Shanghai, China

Wang, Q. et al., Stem Cells 23, 1221-1227 (2005)

SH2

Provider: Shanghai Second Medical University, Shanghai, China

Wang, Q. et al., Stem Cells 23, 1221-1227 (2005)

SH28

Provider: Shanghai Second Medical University, Shanghai, China

Fang, Z. F. et al., Cell Res 15, 394-400 (2005)

SH35

Provider: Shanghai Second Medical University, Shanghai, China

Fang, Z. F. et al., Cell Res 15, 394-400 (2005)

SH35a

Provider: Shanghai Second Medical University, Shanghai, China

Fang, Z. F. et al., Cell Res 15, 394-400 (2005)

SH38

Provider: Shanghai Second Medical University, Shanghai, China

Fang, Z. F. et al., Cell Res 15, 394-400 (2005)

SH39

Provider: Shanghai Second Medical University, Shanghai, China

Fang, Z. F. et al., Cell Res 15, 394-400 (2005)

Zheng, J. K. et al., Cell Res 16, 713-722 (2006)

SH4

Provider: Shanghai Second Medical University, Shanghai, China

Fang, Z. F. et al., Cell Res 15, 394-400 (2005)

SH42

Provider: Shanghai Second Medical University, Shanghai, China

Fang, Z. F. et al., Cell Res 15, 394-400 (2005)

Zhou, J. M. et al., Cell Biol Int 32, 80-85 (2008)

SH7

Provider: Shanghai Second Medical University, Shanghai, China

Wang, Q. et al., Stem Cells 23, 1221 (2005)

Shef-1

Provider: University of Sheffield & Axordia Ltd., UK

Draper, J. S. et al., Stem Cells Dev 13, 325-336 (2004)

Inniss, K. & Moore, H., Stem Cells Dev 15, 789-796 (2006)

Adewumi, O. et al., Nat Biotechnol 25, 803-816 (2007)

Baker, D. E. et al., Nat Biotechnol 25, 207-215 (2007)

Barroso-delJesus, A. et al., Mol Cell Biol 28, 6609-6619 (2008)

Calvanese, V. et al., PLoS ONE 3, e3294 (2008)

Catalina, P. et al., Mol Cancer 7, 76 (2008)

Furue, M. K. et al., Proc Natl Acad Sci U S A 105, 1340913414 (2008)

Inniss, K. et al., Stem Cells Dev 17, 1195 (2008)

Vugler, A. et al., Exp Neurol 214, 347-361 (2008)

Shef-2

Provider: University of Sheffield & Axordia Ltd., UK

Inniss, K. & Moore, H., Stem Cells Dev 15, 789-796 (2006)

Adewumi, O. et al., Nat Biotechnol 25, 803-816 (2007)

Barroso-delJesus, A. et al., Mol Cell Biol 28, 6609-6619 (2008)

Shef-3

Provider: University of Sheffield & Axordia Ltd., UK

Inniss, K. & Moore, H., Stem Cells Dev 15, 789-796 (2006)

Adewumi, O. et al., Nat Biotechnol 25, 803-816 (2007)

Liew, C. G. et al., Stem Cells 25, 1521-1528 (2007)

Catalina, P. et al., Mol Cancer 7, 76 (2008)

Shef-4

Provider: University of Sheffield & Axordia Ltd., UK

Inniss, K. & Moore, H., Stem Cells Dev 15, 789-796 (2006)

Baker, D. E. et al., Nat Biotechnol 25, 207-215 (2007)

Avery, K. et al., Stem Cells Dev 17, 1195-1205 (2008)

Calvanese, V. et al., PLoS ONE 3, e3294 (2008)

Furue, M. K. et al., Proc Natl Acad Sci U S A 105, 1340913414 (2008)

Shef-5

Provider: University of Sheffield & Axordia Ltd., UK

Inniss, K. & Moore, H., Stem Cells Dev 15, 789-796 (2006)

Baker, D. E. et al., Nat Biotechnol 25, 207-215 (2007)

Atlasi, Y. et al., Stem Cells 26, 3068-3074 (2008)

Calvanese, V. et al., PLoS ONE 3, e3294 (2008)

Furue, M. K. et al., Proc Natl Acad Sci U S A 105, 1340913414 (2008)

Shef-6

Provider: University of Sheffield & Axordia Ltd., UK

Inniss, K. & Moore, H., Stem Cells Dev 15, 789-796 (2006)

Shef-7

Provider: University of Sheffield & Axordia Ltd., UK

Calvanese, V. et al., PLoS ONE 3, e3294 (2008)

Vugler, A. et al., Exp Neurol 214, 347-361 (2008)

SHhes1

Provider: Shanghai JiaoTong University School of Medicine, Shanghai, China

Sun, B. W. et al., Hum Mol Genet 15, 65-75 (2006)

SI-125

Provider: Stemride International Ltd., London, UK

Verlinsky, Y. et al., Reprod Biomed Online 10, 105-110 (2005)

Verlinsky, Y. et al., Reprod Biomed Online 13, 547-550 (2006)

SI-128

Provider: Stemride International Ltd., London, UK

Verlinsky, Y. et al., Reprod Biomed Online 10, 105-110 (2005)

Verlinsky, Y. et al., Reprod Biomed Online 13, 547-550 (2006)

SI-137

Provider: Stemride International Ltd., London, UK

Verlinsky, Y. et al., Reprod Biomed Online 10, 105-110 (2005)

Verlinsky, Y. et al., Reprod Biomed Online 13, 547-550 (2006)

SI-138

Provider: Stemride International Ltd., London, UK

Verlinsky, Y. et al., Reprod Biomed Online 10, 105-110 (2005)

Verlinsky, Y. et al., Reprod Biomed Online 13, 547-550 (2006)

SI-139

Provider: Stemride International Ltd., London, UK

Verlinsky, Y. et al., Reprod Biomed Online 10, 105-110 (2005)

Verlinsky, Y. et al., Reprod Biomed Online 13, 547-550 (2006)

SI-140

Provider: Stemride International Ltd., London, UK

Verlinsky, Y. et al., Reprod Biomed Online 10, 105-110 (2005)

Verlinsky, Y. et al., Reprod Biomed Online 13, 547-550 (2006)

SI-141

Provider: Stemride International Ltd., London, UK

Verlinsky, Y. et al., Reprod Biomed Online 10, 105-110 (2005)

Verlinsky, Y. et al., Reprod Biomed Online 13, 547-550 (2006)

SI-145 (hESC-145)

Provider: Stemride International Ltd., London, UK

Verlinsky, Y. et al., Reprod Biomed Online 13, 547-550 (2006)

SI-148

Provider: Stemride International Ltd., London, UK

Verlinsky, Y. et al., Reprod Biomed Online 10, 105-110 (2005)

Verlinsky, Y. et al., Reprod Biomed Online 13, 547-550 (2006)

SI-15

Provider: Stemride International Ltd., London, UK

Strelchenko, N. et al., Reprod Biomed Online 9, 623-629 (2004)

SI-153

Provider: Stemride International Ltd., London, UK

Verlinsky, Y. et al., Reprod Biomed Online 10, 105-110 (2005)

Verlinsky, Y. et al., Reprod Biomed Online 13, 547-550 (2006)

SI-154

Provider: Stemride International Ltd., London, UK

Verlinsky, Y. et al., Reprod Biomed Online 10, 105-110 (2005)

Verlinsky, Y. et al., Reprod Biomed Online 13, 547-550 (2006)

SI-158

Provider: Stemride International Ltd., London, UK

Verlinsky, Y. et al., Reprod Biomed Online 10, 105-110 (2005)

Verlinsky, Y. et al., Reprod Biomed Online 13, 547-550 (2006)

SI-164

Provider: Stemride International Ltd., London, UK

Verlinsky, Y. et al., Reprod Biomed Online 10, 105-110 (2005)

Verlinsky, Y. et al., Reprod Biomed Online 13, 547-550 (2006)

SI-168 (hESC-168)

Provider: Stemride International Ltd., London, UK

Verlinsky, Y. et al., Reprod Biomed Online 13, 547-550 (2006)

SI-170

Provider: Stemride International Ltd., London, UK

Verlinsky, Y. et al., Reprod Biomed Online 10, 105-110 (2005)

Verlinsky, Y. et al., Reprod Biomed Online 13, 547-550 (2006)

SI-18

Provider: Stemride International Ltd., London, UK

Strelchenko, N. et al., Reprod Biomed Online 9, 623-629 (2004)

SI-180

Provider: Stemride International Ltd., London, UK

Verlinsky, Y. et al., Reprod Biomed Online 10, 105-110 (2005)

Verlinsky, Y. et al., Reprod Biomed Online 13, 547-550 (2006)

SI-186

Provider: Stemride International Ltd., London, UK

Verlinsky, Y. et al., Reprod Biomed Online 10, 105-110 (2005)

Verlinsky, Y. et al., Reprod Biomed Online 13, 547-550 (2006)

SI-187

Provider: Stemride International Ltd., London, UK

Verlinsky, Y. et al., Reprod Biomed Online 10, 105-110 (2005)

Verlinsky, Y. et al., Reprod Biomed Online 13, 547-550 (2006)

SI-194

Provider: Stemride International Ltd., London, UK

Verlinsky, Y. et al., Reprod Biomed Online 10, 105-110 (2005)

Verlinsky, Y. et al., Reprod Biomed Online 13, 547-550 (2006)

SI-197

Provider: Stemride International Ltd., London, UK

Verlinsky, Y. et al., Reprod Biomed Online 13, 547-550 (2006)

SI-201

Provider: Stemride International Ltd., London, UK

Verlinsky, Y. et al., Reprod Biomed Online 10, 105-110 (2005)

Verlinsky, Y. et al., Reprod Biomed Online 13, 547-550 (2006)

SI-208 (hESC-208)

Provider: Stemride International Ltd., London, UK

Verlinsky, Y. et al., Reprod Biomed Online 13, 547-550 (2006)

SI-21

Provider: Stemride International Ltd., London, UK

Strelchenko, N. et al., Reprod Biomed Online 9, 623-629 (2004)

SI-213

Provider: Stemride International Ltd., London, UK

Verlinsky, Y. et al., Reprod Biomed Online 13, 547-550 (2006)

SI-214

Provider: Stemride International Ltd., London, UK

Verlinsky, Y. et al., Reprod Biomed Online 13, 547-550 (2006)

SI-218

Provider: Stemride International Ltd., London, UK

Verlinsky, Y. et al., Reprod Biomed Online 13, 547-550 (2006)

SI-233

Provider: Stemride International Ltd., London, UK

Verlinsky, Y. et al., Reprod Biomed Online 13, 547-550 (2006)

SI-235

Provider: Stemride International Ltd., London, UK

Verlinsky, Y. et al., Reprod Biomed Online 13, 547-550 (2006)

SI-24

Provider: Stemride International Ltd., London, UK

Strelchenko, N. et al., Reprod Biomed Online 9, 623-629 (2004)

SI-245 (SC-245, hESC-245)

Provider: Stemride International Ltd., London, UK

Verlinsky, Y. et al., Reprod Biomed Online 13, 547-550 (2006)

SI-246

Provider: Stemride International Ltd., London, UK

Verlinsky, Y. et al., Reprod Biomed Online 13, 547-550 (2006)

SI-252 (hESC-252)

Provider: Stemride International Ltd., London, UK

Verlinsky, Y. et al., Reprod Biomed Online 13, 547-550 (2006)

SI-257 (hESC-257)

Provider: Stemride International Ltd., London, UK

Verlinsky, Y. et al., Reprod Biomed Online 13, 547-550 (2006)

SI-27

Provider: Stemride International Ltd., London, UK

Strelchenko, N. et al., Reprod Biomed Online 9, 623-629 (2004)

SI-271

Provider: Stemride International Ltd., London, UK

Verlinsky, Y. et al., Reprod Biomed Online 13, 547-550 (2006)

SI-274

Provider: Stemride International Ltd., London, UK

Verlinsky, Y. et al., Reprod Biomed Online 13, 547-550 (2006)

SI-277

Provider: Stemride International Ltd., London, UK

Verlinsky, Y. et al., Reprod Biomed Online 13, 547-550 (2006)

SI-279

Provider: Stemride International Ltd., London, UK

Verlinsky, Y. et al., Reprod Biomed Online 13, 547-550 (2006)

SI-28

Provider: Stemride International Ltd., London, UK

Strelchenko, N. et al., Reprod Biomed Online 9, 623-629 (2004)

SI-283

Provider: Stemride International Ltd., London, UK

Verlinsky, Y. et al., Reprod Biomed Online 13, 547-550 (2006)

SI-31

Provider: Stemride International Ltd., London, UK

Strelchenko, N. et al., Reprod Biomed Online 9, 623-629 (2004)

SI-33

Provider: Stemride International Ltd., London, UK

Strelchenko, N. et al., Reprod Biomed Online 9, 623-629 (2004)

SI-53

Provider: Stemride International Ltd., London, UK

Strelchenko, N. et al., Reprod Biomed Online 9, 623-629 (2004)

SI-60

Provider: Stemride International Ltd., London, UK

Strelchenko, N. et al., Reprod Biomed Online 9, 623-629 (2004)

SI-62

Provider: Stemride International Ltd., London, UK

Strelchenko, N. et al., Reprod Biomed Online 9, 623-629 (2004)

SI-63

Provider: Stemride International Ltd., London, UK

Strelchenko, N. et al., Reprod Biomed Online 9, 623-629 (2004)

SI-79

Provider: Stemride International Ltd., London, UK

Strelchenko, N. et al., Reprod Biomed Online 9, 623-629 (2004)

SI-80

Provider: Stemride International Ltd., London, UK

Strelchenko, N. et al., Reprod Biomed Online 9, 623-629 (2004)

SI-81

Provider: Stemride International Ltd., London, UK

Strelchenko, N. et al., Reprod Biomed Online 9, 623-629 (2004)

SI-93

Provider: Stemride International Ltd., London, UK

Strelchenko, N. et al., Reprod Biomed Online 9, 623-629 (2004)

SI-94

Provider: Stemride International Ltd., London, UK

Strelchenko, N. et al., Reprod Biomed Online 9, 623-629 (2004)

SI-95

Provider: Stemride International Ltd., London, UK

Strelchenko, N. et al., Reprod Biomed Online 9, 623-629 (2004)

SI-96

Provider: Stemride International Ltd., London, UK

Strelchenko, N. et al., Reprod Biomed Online 9, 623-629 (2004)

SI-97

Provider: Stemride International Ltd., London, UK

Strelchenko, N. et al., Reprod Biomed Online 9, 623-629 (2004)

SIVF03

Provider: Sidney IVF Ltd., NSW, Australia

Peura, T. T. et al., Theriogenology 67, 32-42 (2007)

Peura, T. et al., Cloning Stem Cells 10, 203-216 (2008)

SIVF04

Provider: Sidney IVF Ltd., NSW, Australia

Peura, T. T. et al., Theriogenology 67, 32-42 (2007)

Peura, T. et al., Cloning Stem Cells 10, 203-216 (2008)

SIVF05

Provider: Sidney IVF Ltd., NSW, Australia

Peura, T. T. et al., Theriogenology 67, 32-42 (2007)

Peura, T. et al., Cloning Stem Cells 10, 203-216 (2008)

SIVF06

Provider: Sidney IVF Ltd., NSW, Australia

Peura, T. T. et al., Theriogenology 67, 32-42 (2007)

Peura, T. et al., Cloning Stem Cells 10, 203-216 (2008)

SIVF07

Provider: Sidney IVF Ltd., NSW, Australia

Peura, T. T. et al., Theriogenology 67, 32-42 (2007)

Peura, T. et al., Cloning Stem Cells 10, 203-216 (2008)

SIVF08

Provider: Sidney IVF Ltd., NSW, Australia

Peura, T. T. et al., Theriogenology 67, 32-42 (2007)

Peura, T. et al., Cloning Stem Cells 10, 203-216 (2008)

SIVF09

Provider: Sidney IVF Ltd., NSW, Australia

Peura, T. T. et al., Theriogenology 67, 32-42 (2007)

Peura, T. et al., Cloning Stem Cells 10, 203-216 (2008)

SIVF10

Provider: Sidney IVF Ltd., NSW, Australia

Peura, T. T. et al., Theriogenology 67, 32-42 (2007)

Peura, T. et al., Cloning Stem Cells 10, 203-216 (2008)

SIVF11

Provider: Sidney IVF Ltd., NSW, Australia

Peura, T. T. et al., Theriogenology 67, 32-42 (2007)

Peura, T. et al., Cloning Stem Cells 10, 203-216 (2008)

SIVF12

Provider: Sidney IVF Ltd., NSW, Australia

Peura, T. et al., Cloning Stem Cells 10, 203-216 (2008)

SIVF13

Provider: Sidney IVF Ltd., NSW, Australia

Peura, T. et al., Cloning Stem Cells 10, 203-216 (2008)

SNUhES1

Provider: Seoul National University, Seoul, Korea

Oh, S. K. et al., Stem Cells 23, 211-219 (2005)

Kang, H. B. et al., Stem Cells Dev 16, 615-623 (2007)

Cho, M. S. et al., Proc Natl Acad Sci U S A 105, 3392-3397 (2008)

SNUhES11

Provider: Seoul National University, Seoul, Korea

Seol, H. W. et al., Chromosome Res 16, 1075-1084 (2008)

SNuhES16

Provider: Seoul National University, Seoul, Korea

Cho, M. S. et al., Proc Natl Acad Sci U S A 105, 3392-3397 (2008)

Jang, J. et al., Stem Cells 26, 2782-2790 (2008)

Muller, F. J. et al., Nature 455, 401-405 (2008)

Seol, H. W. et al., Chromosome Res 16, 1075-1084 (2008)

SNUhES2

Provider: Seoul National University, Seoul, Korea

Kwon, Y. D. et al., Mol Ther 12, 28-32 (2005)

Oh, S. K. et al., Stem Cells 23, 211-219 (2005)

SNUhES3

Provider: Seoul National University, Seoul, Korea

Suh, M. R. et al., Dev Biol 270, 488-498 (2004)

Ha, S. Y. et al., Hum Reprod 20, 1779-1785 (2005)

Kim, J. H. et al., Exp Mol Med 37, 36-44 (2005)

Kim, S. J., et al.,Yonsei Med J 46, 693-699 (2005)

Kim, S. K. et al., Stem Cells 23, 458-462 (2005)

Kwon, Y. D. et al., Mol Ther 12, 28-32 (2005)

Oh, S. K. et al., Stem Cells 23, 211-219 (2005)

Park, C. H. et al., J Neurochem 92, 1265-1276 (2005)

Cho, Y. M. et al., Biochem Biophys Res Commun 348, 1472-1478 (2006)

Kim, C. G. et al., Mol Cells 21, 343-355 (2006)

Kim, S. J. et al., Acta Haematol 116, 219-222 (2006)

Lee, J. et al., J Biol Chem 281, 33554-33565 (2006)

Rho, J. Y. et al., Hum Reprod 21, 405-412 (2006)

Kang, S. M. et al., Stem Cells 25, 419-424 (2007)

Kim, S. J. et al., Stem Cells Dev 16, 421-428 (2007)

Yeo, S. et al., Biochem Biophys Res Commun 359, 536-542 (2007)

Cho, M. S. et al., Proc Natl Acad Sci U S A 105, 3392-3397 (2008)

Cho, Y. M. et al., Biochem Biophys Res Commun 366, 129-134 (2008)
Jang, J. et al., Stem Cells 26, 2782-2790 (2008)
Kim, Y. Y. et al., Yonsei Med J 49, 819-827 (2008)
Park, Y. B. et al., Exp Mol Med 40, 98-108 (2008)
Seol, H. W. et al., Chromosome Res 16, 1075-1084 (2008)
Son, M. Y. et al., Reproduction 136, 423-432 (2008)

SNUhES4

Provider: Seoul National University, Seoul, Korea

Kwon, Y. D. et al., Mol Ther 12, 28-32 (2005)
Jang, J. et al., Stem Cells 26, 2782-2790 (2008)
Kim, Y. Y. et al., Yonsei Med J 49, 819-827 (2008)
Seol, H. W. et al., Chromosome Res 16, 1075-1084 (2008)

SYSU-1

Provider: Sun Yat-sen University, Guangzhou, China

Huang, G. et al., Chin Med J (Engl), 120, 589-594 (2007)

SYSU-2

Provider: Sun Yat-sen University, Guangzhou, China

Huang, G. et al., Chin Med J (Engl), 120, 589-594 (2007)

T1

Provider: Kaohsiung Medical University, Taiwan

Li, S. S. et al., Stem Cells Dev 15, 532-555 (2006)

T2

Provider: Kaohsiung Medical University, Taiwan

Li, S. S. et al., Stem Cells Dev 15, 532-555 (2006)

T3

Provider: Kaohsiung Medical University, Taiwan

Li, S. S. et al., Stem Cells Dev 15, 532-555 (2006)

T4

Provider: Kaohsiung Medical University, Taiwan

Li, S. S. et al., Stem Cells Dev 15, 532-555 (2006)

T5

Provider: Kaohsiung Medical University, Taiwan

Li, S. S. et al., Stem Cells Dev 15, 532-555 (2006)

TW1

Provider: China Medical University, Taichung, Taiwan

Cheng, E. H. et al., Reprod Biomed Online 17, 436-444 (2008)

TW2

Provider: China Medical University, Taichung, Taiwan

Cheng, E. H. et al., Reprod Biomed Online 17, 436-444 (2008)

TW3

Provider: China Medical University, Taichung, Taiwan

Cheng, E. H. et al., Reprod Biomed Online 17, 436-444 (2008)

TW4

Provider: China Medical University, Taichung, Taiwan

Cheng, E. H. et al., Reprod Biomed Online 17, 436-444 (2008)

TW5

Provider: China Medical University, Taichung, Taiwan

Cheng, E. H. et al., Reprod Biomed Online 17, 436-444 (2008)

UCSF-1

Provider: University of California, San Francisco, CA, USA

Genbacev, O. et al., Fertil Steril 83, 1517-1529 (2005)

UCSF-2

Provider: University of California, San Francisco, CA, USA

Genbacev, O. et al., Fertil Steril 83, 1517-1529 (2005)

VAL-1

Provider: Valencia Stem Cell Bank, Spain

Simon, C. et al., Fertil Steril 83, 246-249 (2005)

Krtolica, A. et al., Stem Cells 25, 2215-2223 (2007)

VAL-2

Provider: Valencia Stem Cell Bank, Spain

Simon, C. et al., Fertil Steril 83, 246-249 (2005)

Krtolica, A. et al., Stem Cells 25, 2215-2223 (2007)

VAL-3

Provider: Valencia Stem Cell Bank, Spain

Valbuena, D. et al., Reprod Biomed Online 13, 875-886 (2006)

Valbuena, D. et al., Reprod Biomed Online 17, 127-135 (2008)

VAL-4

Provider: Valencia Stem Cell Bank, Spain

Valbuena, D. et al., Reprod Biomed Online 13, 875-886 (2006)

VAL-5

Provider: Valencia Stem Cell Bank, Spain

Valbuena, D. et al., Reprod Biomed Online 13, 875-886 (2006)

Valbuena, D. et al., Reprod Biomed Online 17, 127-135 (2008)

VUB01

Provider: Vrije Universiteit Brussel, Belgium

Mateizel, I. et al., Hum Reprod 21, 503-511 (2006)

Willems, E. et al., Int J Dev Biol 50, 627-635 (2006)

Ullmann, U. et al., Mol Hum Reprod 13, 21-32 (2007)

Come, J. et al., Tissue Eng Part C Methods 14, 289-298 (2008)

De Temmerman, N. et al., Mol Hum Reprod 14, 405-412 (2008)

Kuntz, S. et al., Stem Cells 26, 734-744 (2008)

Lefort, N. et al., Nat Biotechnol 26, 1364-1366 (2008)

Mateizel, I. et al., Reprod Biomed Online 16, 741-753 (2008)

Spits, C. et al., Nat Biotechnol 26, 1361-1363 (2008)

VUB02

Provider: Vrije Universiteit Brussel, Belgium

Mateizel, I. et al., Hum Reprod 21, 503-511 (2006)

Willems, E. et al., Int J Dev Biol 50, 627-635 (2006)

Mateizel, I. et al., Reprod Biomed Online 16, 741-753 (2008)

Spits, C. et al., Nat Biotechnol 26, 1361-1363 (2008)

VUB03_DM1

Provider: Vrije Universiteit Brussel, Belgium

Cauffman, G. et al., Stem Cells 24, 2685-91 (2006)

Mateizel, I. et al., Hum Reprod 21, 503-511 (2006)

Ullmann, U. et al., Mol Hum Reprod 13, 21-32 (2007)

De Temmerman, N. et al., Mol Hum Reprod 14, 405-412 (2008)

Mateizel, I. et al., Reprod Biomed Online 16, 741-753 (2008)

Spits, C. et al., Nat Biotechnol 26, 1361-1363 (2008)

Ullmann, U. et al., Mol Hum Reprod 14, 169-179 (2008)

VUB04_CF

Provider: Vrije Universiteit Brussel, Belgium

Mateizel, I. et al., Hum Reprod 21, 503-511 (2006)

Ullmann, U. et al., Mol Hum Reprod 13, 21-32 (2007)

De Temmerman, N. et al., Mol Hum Reprod 14, 405-412 (2008)

Spits, C. et al., Nat Biotechnol 26, 1361-1363 (2008)

Ullmann, U. et al., Mol Hum Reprod 14, 169-179 (2008)

VUB05_HD

Provider: Vrije Universiteit Brussel, Belgium

Mateizel, I. et al., Hum Reprod 21, 503-511 (2006)

Lefort, N. et al., Nat Biotechnol 26, 1364-1366 (2008)

Spits, C. et al., Nat Biotechnol 26, 1361-1363 (2008)

VUB06

Provider: Vrije Universiteit Brussel, Belgium

Spits, C. et al., Nat Biotechnol 26, 1361-1363 (2008)

VUB07

Provider: Vrije Universiteit Brussel, Belgium

Spits, C. et al., Nat Biotechnol 26, 1361-1363 (2008)

Ullmann, U. et al., Mol Hum Reprod 14, 169-179 (2008)

VUB08_MFS

Provider: Vrije Universiteit Brussel, Belgium

Spits, C. et al., Nat Biotechnol 26, 1361-1363 (2008)

VUB09_FSHD

Provider: Vrije Universiteit Brussel, Belgium

Spits, C. et al., Nat Biotechnol 26, 1361-1363 (2008)

VUB11_FXS

Provider: Vrije Universiteit Brussel, Belgium

Spits, C. et al., Nat Biotechnol 26, 1361-1363 (2008)

VUB13_FXS

Provider: Vrije Universiteit Brussel, Belgium

Spits, C. et al., Nat Biotechnol 26, 1361-1363 (2008)

VUB15

Provider: Vrije Universiteit Brussel, Belgium

Spits, C. et al., Nat Biotechnol 26, 1361-1363 (2008)

VUB17

Provider: Vrije Universiteit Brussel, Belgium

Spits, C. et al., Nat Biotechnol 26, 1361-1363 (2008)

VUB19_DM1

Provider: Vrije Universiteit Brussel, Belgium

Spits, C. et al., Nat Biotechnol 26, 1361-1363 (2008)

VUB20_CMT1A

Provider: Vrije Universiteit Brussel, Belgium

Spits, C. et al., Nat Biotechnol 26, 1361-1363 (2008)

VUB24_DM1

Provider: Vrije Universiteit Brussel, Belgium

Spits, C. et al., Nat Biotechnol 26, 1361-1363 (2008)

VUB26 (VUB26_QUATRO)

Provider: Vrije Universiteit Brussel, Belgium

Spits, C. et al., Nat Biotechnol 26, 1361-1363 (2008)

WA15

Provider: WiCell Research Institute, Mdison, WI, USA

Ludwig, T. E. et al., Nat Biotechnol 24, 185-187 (2006)

WA16

Provider: WiCell Research Institute, Mdison, WI, USA

Ludwig, T. E. et al., Nat Biotechnol 24, 185-187 (2006)
